# Di‐Iron(II) [2+2] Helicates of Bis‐(Dipyrazolylpyridine) Ligands: The Influence of the Ligand Linker Group on Spin State Properties

**DOI:** 10.1002/chem.202202578

**Published:** 2022-12-27

**Authors:** Rafal Kulmaczewski, Isaac T. Armstrong, Pip Catchpole, Emily S. J. Ratcliffe, Hari Babu Vasili, Stuart L. Warriner, Oscar Cespedes, Malcolm A. Halcrow

**Affiliations:** ^1^ School of Chemistry University of Leeds Woodhouse Lane Leeds LS2 9JT UK; ^2^ Department of Chemistry Lancaster University Lancaster LA1 4YB UK; ^3^ School of Physics and Astronomy W. H. Bragg Building, University of Leeds Leeds LS2 9JT UK

**Keywords:** helicate complexes, iron, N-ligands, self-assembly, spin-crossover

## Abstract

Four bis[2‐{pyrazol‐1‐yl}‐6‐{pyrazol‐3‐yl}pyridine] ligands have been synthesized, with butane‐1,4‐diyl (*L* ^1^), pyrid‐2,6‐diyl (*L* ^2^), benzene‐1,2‐dimethylenyl (*L* ^3^) and propane‐1,3‐diyl (*L* ^4^) linkers between the tridentate metal‐binding domains. *L* ^1^ and *L* ^2^ form [Fe_2_(μ−*L*)_2_]X_4_ (X^−^=BF_4_^−^ or ClO_4_^−^) helicate complexes when treated with the appropriate iron(II) precursor. Solvate crystals of [Fe_2_(μ−*L* ^1^)_2_][BF_4_]_4_ exhibit three different helicate conformations, which differ in the torsions of their butanediyl linker groups. The solvates exhibit gradual thermal spin‐crossover, with examples of stepwise switching and partial spin‐crossover to a low‐temperature mixed‐spin form. Salts of [Fe_2_(μ−*L* ^2^)_2_]^4+^ are high‐spin, which reflects their highly twisted iron coordination geometry. The composition and dynamics of assembly structures formed by iron(II) with *L* ^1^−*L* ^3^ vary with the ligand linker group, by mass spectrometry and ^1^H NMR spectroscopy. Gas‐phase DFT calculations imply the butanediyl linker conformation in [Fe_2_(μ−*L* ^1^)_2_]^4+^ influences its spin state properties, but show anomalies attributed to intramolecular electrostatic repulsion between the iron atoms.

## Graphical abstract

This diiron(II) complex has been crystallized in three different helicate conformations, which differ in the torsions of the butane‐1,4‐diyl ligand linker groups. The crystals exhibit a range of spin state properties, including stepwise spin‐crossover of the two iron atoms. A related ligand with a rigid pyrid‐2,6‐diyl spacer forms more a distorted, high‐spin diiron(II) helicate structure.

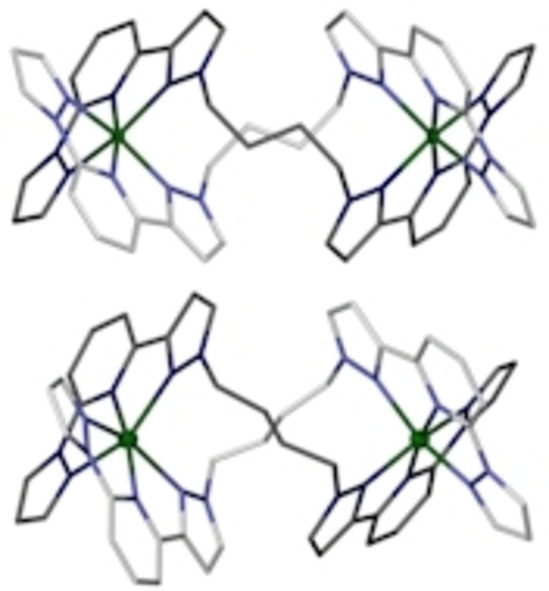

## Introduction

Spin‐crossover (SCO) compounds are versatile molecular switches, where a transition ion undergoes a change in spin state under heating/cooling, hydrostatic pressure, visible light irradiation or another physical stimulus.[^1^, ^2^, ^3^, ^4^] An SCO transition influences several bulk properties of a solid material,[^2^, ^5^] which has been harnessed in the laboratory for macroscopic^[6]^ and nanoscale^[7]^ applications including SCO compounds as switching components. Supramolecular assemblies of multiple SCO centers afford spatially defined arrays of SCO sites, which may switch independently or in concert depending on their topology and structural rigidity.[^8^, ^9^] Moreover, some assembly structures can modulate their spin states by encapsulating guest molecules,[^10^, ^11^, ^12^] or through other supramolecular interactions.^[13]^ Molecular squares or grids,[^8^, ^9^, ^14^, ^15^] tetrahedral[^8^, ^10^, ^16^, ^17^, ^18^] and cubane[^9^, ^11^, ^19^, ^20^] cage complexes with SCO‐active vertices are well‐established, while other SCO cluster and cage architectures have also been reported.[^9^, ^21^, ^22^, ^23^, ^24^, ^25^, ^26^, ^27^] However, the best‐developed class of SCO supramolecular assembly is also one of the simplest, namely helicate complexes.[^8^, ^16^]

The first dinuclear SCO helicates were reported by Piguet et al., who combined SCO iron(II) centers and emissive lanthanide ions to produce switchable, emissive molecular constructs based on [3+3]‐helicate scaffolds.^[28]^ More recently, ditopic bis‐bidentate Schiff bases[^16^, ^17^, ^29^, ^30^] or bis‐diheterocyclic ligands[^12^, ^13^, ^31^] have been versatile scaffolds for di‐iron(II) [3+3]‐helicates, which often exhibit thermal SCO. Using longer spacers between the metal‐binding domains in these ligands affords helicates with internal cavities, which can encapsulate anion guests. The guest species influence the temperature and completeness of SCO, with larger guests disfavoring the low‐spin state on steric grounds.^[12]^

All these examples are [3+3]‐helicate assemblies, with three bis‐bidentate ligands wrapped around six‐coordinate metal centers. SCO in diiron(II) [3+3]‐helicate complexes usually occurs gradually with temperature, and is often ill‐defined and incomplete. Conversely, there is just one prior example of a diiron(II) [2+2]‐helicate complex supported by a bis‐tridentate ligand scaffold, whose salts exhibit abrupt and hysteretic spin‐transitions in the solid state.^[32]^ Such cooperative switching properties are more useful for the applications listed above.

Some of the most widely studied SCO complexes are derived from [Fe(bpp)_2_]^2+^ salts (bpp=2,6‐di{pyrazolyl}pyridine). Three isomers of the bpp ligand are available: 2,6‐di{pyrazol‐1‐yl}pyridine (1‐bpp);[^33^, ^34^, ^35^, ^36^] 2,6‐di{1*H*‐pyrazol‐3‐yl}pyridine (3‐bpp);[^37^, ^38^] and the unsymmetric 2‐{pyrazol‐1‐yl}‐6‐{1*H*‐pyrazol‐3‐yl}pyridine (1,3‐bpp).[^34^, ^39^, ^40^, ^41^] Hundreds of iron(II) complex salts supported by bpp derivatives are known, many of which exhibit SCO at accessible temperatures.[^33^, ^34^, ^35^, ^36^, ^37^, ^38^, ^39^, ^40^, ^41^]

**Scheme 1:**
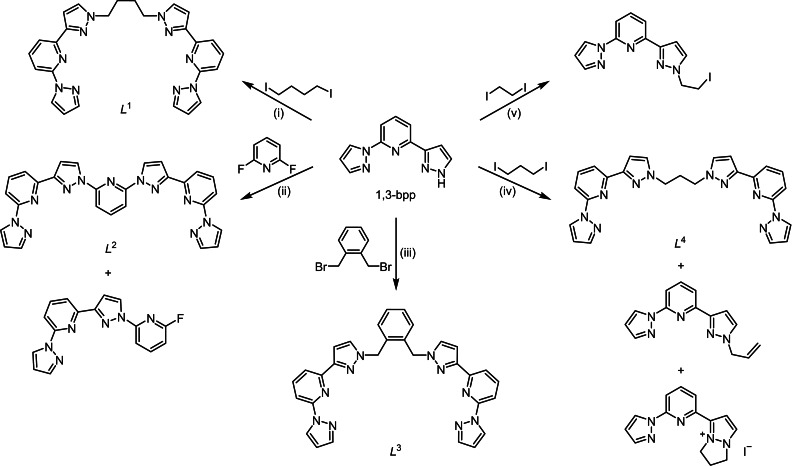
The ligand synthesis reactions undertaken in this work. Reagents and conditions: Reagents and conditions: (i) NaH, thf, 298 K then 0.5 equiv. 1,4‐diiodobutane, reflux, 72 h; (ii) NaH, dmf, 298 K then 0.5 equiv. 2,6‐difluoropyridine, reflux, 24 h; (iii) NaH, thf, 298 K then 0.5 equiv. 1,2‐bis(bromomethyl)‐benzene, reflux, 72 h; NaH, thf, 298 K then 0.5 equiv. 1,3‐diiodopropane, reflux, 24 h; (v) NaH, diglyme then 0.5 equiv. 1,2‐diiodoethane, 130 °C, 2–14 days.

As a continuation of our long‐standing interest in [Fe(bpp)_2_]^2+^ chemistry,[^33^, ^34^, ^35^] we now report an investigation of ditopic ligands containing two 1,3‐bpp domains linked by different spacers (Scheme 1).[^42^, ^43^, ^44^] Some of these cleanly yielded dinuclear iron(II) helicate complexes. Different crystals of one complex adopt one of three helical conformations, which differ in the torsions of the ligand linker and show distinct spin state behaviors. DFT calculations investigating the influence of the linker conformation on the complex spin state are also described, which highlight unexpected challenges in computing the spin states of multinuclear complexes.

## Results and Discussion

Deprotonation of 1,3‐bpp^[39]^ in dry tetrahydrofuran (thf) or *N*,*N*‐dimethylformamide (dmf), then addition of 0.5 equiv. 1,4‐diodobutane, 2,6‐difluoropyridine or 1,2‐bis(bromomethyl)‐benzene, yields 1,4‐bis(3‐{2‐[pyrazol‐1‐yl]pyrid‐6‐yl}pyrazol‐1‐yl)butane (*L* ^1^), 2,6‐bis(3‐{2‐[pyrazol‐1‐yl]pyrid‐6‐yl}pyrazol‐1‐yl)pyridine (*L* ^2^) and 1,2‐bis‐(3‐{2‐[pyrazol‐1‐yl]pyrid‐6‐yl}pyrazol‐1‐ylmethyl)benzene (*L* ^3^) after 1–3 days under heating (Scheme 1). While *L* ^1^ and *L* ^3^ were obtained in high yields in NMR purity, *L* ^2^ was always contaminated by its monosubstituted byproduct as shown in Scheme 1. Their poor solubility made it impossible to separate the two compounds. However the mixture still afforded analytically pure iron(II)/*L* ^2^ complexes when treated with iron salts, as described below.

Attempts to prepare analogues of *L* ^1^ with shorter alkyl linker groups were less successful. The desired 1,3‐bis(3‐{2‐[pyrazol‐1‐yl]pyrid‐6‐yl}pyrazol‐1‐yl)propane (*L* ^4^, Scheme 1) was obtained by this route by using 1,3‐diiodopropane as starting material, but in inconsistent lower yields. Two significant byproducts of this reaction were identified, which are both derived from monosubstituted (3‐iodopropyl)‐1,3‐bpp (Scheme 1). The same process using 1,2‐diiodoethane gave monosubstituted (2‐iodoethyl)‐1,3‐bpp as the only isolable product, even after two weeks of reaction at higher temperatures in diglyme (Scheme 1). Hence, double substitution of *α*,*ω*‐dioodoalkanes by 2 equiv. 1,3‐bpp is apparently sluggish for short chain lengths, and the butane‐diyl group in *L* ^1^ was the shortest alkyl linker that was successfully used in this study.

Treatment of *L* ^1^ or *L* ^2^ with 1 equiv. FeX_2_ ⋅ 6H_2_O (X^−^=BF_4_^−^ or ClO_4_^−^) in nitromethane afforded orange‐brown (for *L* ^1^) or bright yellow (*L* ^2^) solids after the usual workup. The complexes were identified as dinuclear helicates, [Fe_2_(μ−*L*)_2_]X_4_ (L=*L* ^1^, **1X_4_**; L=*L* ^2^, **2X_4_**) by X‐ray crystallography, microanalysis, mass spectrometry and ^1^H NMR. Analogous complexations using *L* ^3^ yielded glassy orange/yellow solids of uncertain composition, which are described in more detail below. No analytically pure complex of *L* ^4^ was obtained during this study.

The salt **1[BF_4_]_4_** was crystallized from three different solvents using diethyl ether as antisolvent. Crystals grown from acetonitrile (**1[BF_4_]_4_** ⋅ 2MeCN ⋅ Et_2_O) or acetone (**1[BF_4_]_4_** ⋅ *n*Me_2_CO, *n*≈2.5) were visually homogeneous. However, recrystallization of **1[BF_4_]_4_** from MeNO_2_/Et_2_O affords two pseudopolymorphs with needle (**1[BF_4_]_4_** ⋅ *m*MeNO_2,_ *m* ≈4.5) and prismatic (**1[BF_4_]_4_** ⋅ 2MeNO_2_) morphologies. All these solvates were crystallographically characterized, although the refinement of **1[BF_4_]_4_** ⋅ *m*MeNO_2_ is highly disordered and less precise than for the other crystals.

**Figure 1:**
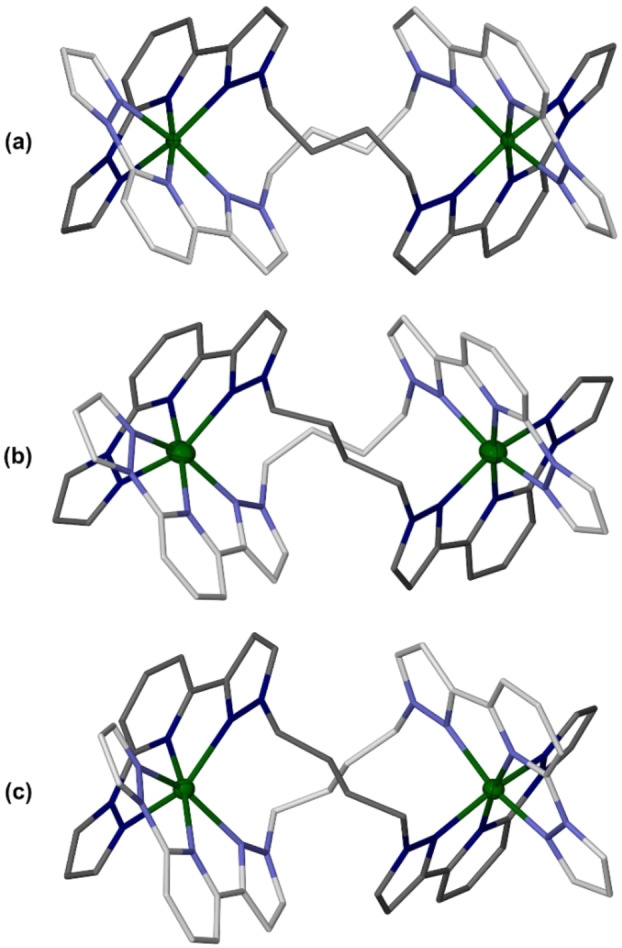
The conformations adopted by [Fe_2_(μ−*L* ^1^)_2_]^4+^ in: (a) **1[BF_4_]_4_** ⋅ 2MeNO_2_; (b) **1[BF_4_]_4_** ⋅ *n*Me_2_CO; and (c) **1[BF_4_]_4_** ⋅ 2MeCN ⋅ Et_2_O. The *L* ^1^ ligands in each molecule are distinguished with pale and dark coloration, and H atoms are omitted for clarity. The crystallographic view of molecule (a) has been inverted, to give it the same handedness as the other molecules in the Figure. Color code: C, white or gray; N, pale or dark blue; Fe, green.

Three helicate conformations are observed in the four structures, which differ in the torsions of their butanediyl linker groups. In **1[BF_4_]_4_** ⋅ 2MeNO_2_ [Figure 1, molecule (a)] both butanediyl linkers have two *gauche* torsions. Both its iron atoms are low‐spin at the temperature of measurement (125 K; Table 1). In contrast, **1[BF_4_]_4_** ⋅ 2MeCN ⋅ Et_2_O [molecule (c)] has just one *gauche* torsion at each butanediyl group. One iron atom in that crystal is high‐spin and the other is low‐spin at 125 K. The complex in **1[BF_4_]_4_** ⋅ *m*MeNO_2_ also adopts conformation (c), and is predominantly high‐spin at that temperature (Figure S17, Table 1). Lastly, the helicate in **1[BF_4_]_4_** ⋅ *n*Me_2_CO [molecule (b)] contains one of each butanediyl group conformation, and was measured at two temperatures. Both its iron atoms are high‐spin at 250 K. However, at 100 K Fe(1) adopts a mixed high/low‐spin population, while Fe(2) has become fully low‐spin (Table 1). Hence, the two iron centers in **1[BF_4_]_4_** ⋅ *n*Me_2_CO evidently undergo SCO at different temperatures on cooling.

**Table 1:** Structural parameters from the crystal structures of **1[BF_4_]_4_**.^[a]^ The conformations listed refer to those in Figure 1.

	1[BF_4_]_4_ ⋅ 2MeNO_2_	1[BF_4_]_4_ ⋅ *n*Me_2_CO		1[BF_4_]_4_ ⋅ 2MeCN ⋅ Et_2_O	1[BF_4_]_4_ ⋅ *m*MeNO_2_^[b]^
Conformation	(a)	(b)		(c)	(c)
*T* [K]	125	250	100	125	125
*V*_Oh_ {Fe(1), Fe(2)} [Å^3^]	9.649(13), 9.651(12)	12.318(10), 12.249(10)	11.231(14), 9.965(12)	12.19(3), 9.92(2)	11.32(4)/11.06(4), 11.27(4)/10.91(4)
Fe ⋅ ⋅ ⋅ Fe [Å]	8.5452(11)	8.1477(6)	8.1221(9)	7.8570(18)	7.8406(22)
*Σ* {Fe(1), Fe(2)} [°]	88.9(5), 87.7(6)	141.2(3), 139.8(4)	125.7(6), 97.1(5)	157(1), 89(1)	142.4(16)/111.7(18), 129.8(17)/106.8(19)
*Θ* {Fe(1), Fe(2)} [°]	295, 290	466, 458	483, 313	493, 295	469/357, 430/352
*ϕ* {Fe(1), Fe(2)} [°]	171.8(2), 174.1(2)	168.53(10), 173.69(11)	171.54(14), 175.52(16)	167.9(3), 173.4(3)	170.6(3), 172.8(4)
*θ* {Fe(1), Fe(2)} [°]	87.66(3), 84.28(3)	85.96(3), 83.62(3)	85.43(4), 84.30(4)	73.87(7), 84.22(7)	79.46(10)/81.08(9), 79.65(11)/80.42(12)

[a] *V*_Oh_, *Σ*, and *Θ* are indices characteristic for the spin state of a complex,^[49]^ while *φ* and *θ* are defined in the text and relate to structural distortions found in [Fe(bpp)_2_]^2+^ derivatives.[^45^, ^46^] [b] There is pyrazolyl group disorder in this crystal structure, and values for both ligand disorder sites are given for each iron atom.

The relative orientations of the two [Fe(bpp)_2_]^2+^ domains are quite similar in all these helicate conformations (Figure 1). However, each additional butanediyl *gauche* torsion pushes the two iron atoms further apart, by 0.3–0.4 Å (Table 1). The two solvates adopting conformation (c) exhibit almost identical Fe ⋅ ⋅ ⋅ Fe distances, implying this parameter may be only slightly perturbed by crystal packing effects. SCO in **1[BF_4_]_4_** ⋅ *n*Me_2_CO also has little effect on its Fe ⋅ ⋅ ⋅ Fe separation (Table 1).

**Figure 2:**
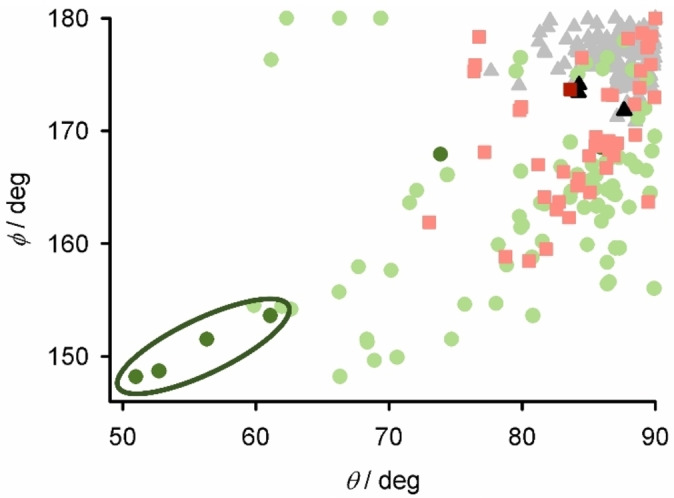
Published distortion parameters for [Fe(1‐bpp)_2_]^2+^ complexes which are low‐spin (gray triangles); high‐spin and SCO‐active (red squares); and which remain high‐spin on cooling (green circles).[^33^, ^34^, ^35^, ^36^] The iron centers in **1[BF_4_]_4_** and **2[ClO_4_]_4_** (Tables 1 and 2) are plotted using the same symbols in dark coloration, with data from the structures of **2[ClO_4_]_4_** being circled.

The metric parameters at the iron centers in the **1[BF_4_]_4_** solvates are mostly typical for SCO‐active [Fe(bpp)_2_]^2+^ derivatives (Table 1).[^33^, ^34^, ^35^, ^36^, ^37^, ^38^, ^39^, ^40^, ^41^] An exception is Fe(1) in **1[BF_4_]_4_** ⋅ 2MeCN ⋅ Et_2_O, which is high‐spin at 125 K with a more distorted coordination geometry. This is described by two parameters: the *trans*‐N{pyridyl}−Fe−N{pyridyl} angle (*ϕ*), which is 167.9(3)°; and, the least squares planes of the two bpp moieties bound to each metal atom (*θ*), which is 73.87(7)°.[^45^, ^46^] An undistorted metal center of this type would have *ϕ*=180 and *θ*=90°. Crystalline, high‐spin [Fe(bpp)_2_]^2+^ derivatives with comparable distortions to Fe(1) rarely exhibit thermal SCO,^[47]^ and are kinetically trapped in their high‐spin form upon cooling.^[48]^ Hence, the distorted geometry at Fe(1) may explain the incomplete SCO in **1[BF_4_]_4_** ⋅ 2MeCN ⋅ Et_2_O (Figure 2). The other iron atom in that structure, Fe(2), is low‐spin at the temperature of measurement and adopts a more regular coordination geometry, as expected.

**Figure 3:**
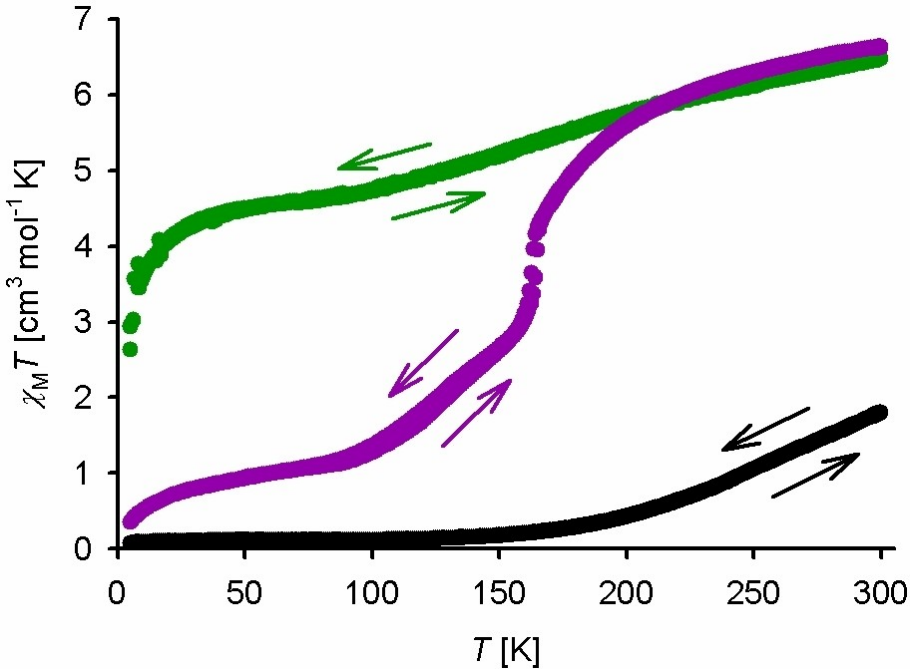
Variable temperature magnetic susceptibility data for dried polycrystalline samples of: **1[BF_4_]_4_** ⋅ *m*MeNO_2_ (black); **1[BF_4_]_4_** ⋅ *n*Me_2_CO (purple); and **1[BF_4_]_4_** ⋅ 2MeCN ⋅ Et_2_O (green). All data were measured on a 300–5‐300 K temperature ramp, at scan rate 5 K min^−1^.

The polycrystalline **1[BF_4_]_4_** solvatomorphs decompose to powders when exposed to air, leading to significant structural changes or loss of crystallinity by powder diffraction (Figures S20–S22). Elemental analysis implies some lattice solvent is retained, or replaced by atmospheric moisture, in the air‐dried solids. Magnetic susceptibility data show the materials undergo gradual thermal SCO, although each is different in form (Figure 3). All the magnetic data are reversible in cooling and warming temperature ramps, and so are not affected by in situ solvent loss. While no single crystals of **1[ClO_4_]_4_** were achieved, samples of that salt obtained from the same solvents show comparable X‐ray powder patterns and SCO profiles to their BF_4_^−^ analogues (Figures S23‐S25).

Most interestingly, air‐dried **1[BF_4_]_4_** ⋅ *n*Me_2_CO is high‐spin at 300 K and undergoes stepwise SCO on cooling, with an abrupt discontinuity near 160 K corresponding to 50 % conversion. That is consistent with the crystallographic observation that the two iron sites in that material undergo SCO at different temperatures. The transition is ca. 80 % complete at 100 K in the magnetic data, but shows a residual paramagnetism below that temperature. That implies kinetic trapping of the remaining material in its high‐spin state below 100 K,^[50]^ which is often observed in [Fe(bpp)_2_]^2+^ derivatives whose SCO extends to such low temperature.[^47^, ^51^]

A mixture of the nitromethane solvates of **1[BF_4_]_4_** ⋅ *m*MeNO_2_ undergoes significant structural changes during air‐drying by powder diffraction. The dried material is low‐spin below 100 K, and shows a very gradual continuous SCO above that temperature such that ca. 30 % of its iron atoms are high‐spin at 300 K (Figure 3). Interestingly, that is more consistent with the crystal structure of the minor solvatomorph **1[BF_4_]_4_** ⋅ 2MeNO_2_, which is low‐spin at 125 K, than with the major crystal form **1[BF_4_]_4_** ⋅ *m*MeNO_2_. Conversely, air‐dried **1[BF_4_]_4_** ⋅ 2MeCN ⋅ Et_2_O is poorly crystalline and is high‐spin at room temperature, showing a gradual SCO on cooling which is ca. 30 % complete at 100 K.

**Figure 4:**
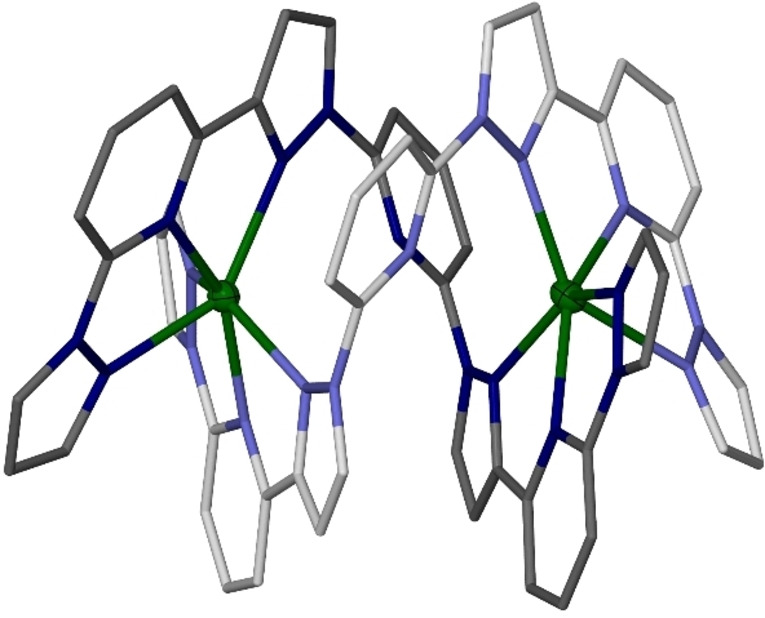
The [Fe_2_(μ−*L* ^2^)_2_]^4+^ helicate in the crystal structure of **2[ClO_4_]_4_**. Details as for Figure 1.

Full structure analyses were obtained from solvent‐free **2[ClO_4_]_4_**, and a solvate crystal **2[ClO_4_]_4_** ⋅ 3MeNO_2_ ⋅ 0.75H_2_O. The [Fe_2_(μ−*L* ^2^)_2_]^4+^ helicate has the same ligand conformation in both crystals, with the iron atoms being bound by the two *L* ^2^ ligands in the expected bis‐tridentate fashion (Figure 4). While crystals of **2[BF_4_]_4_** diffracted X‐rays more weakly, a partial refinement from a nitromethane solvate of that salt confirmed it has the same connectivity as the perchlorate crystals (Figure S28). The metal ions are high‐spin from their metric parameters, and adopt highly twisted coordination geometries with 148.18(9)≤*ϕ*≤153.60(9)° and 50.96(3)≤*θ*≤61.08(2)° (Table 2).^[45]^ These include the most severe *θ* distortions yet reported for a [Fe(bpp)_2_]^2+^ derivative,^[46]^ which probably reflects the steric constraints of the rigid *L* ^2^ ligands. Interestingly, *ϕ* and *θ* follow an almost linear relationship in these two crystals, implying the iron atoms in [Fe_2_(μ−*L* ^2^)_2_]^4+^ consistently follow the same structural distortion pathway (Figure 2).

**Table 2:** Structural parameters from the crystal structures of **2[ClO_4_]_4_**_._ Details as for Table 1.

	2[ClO_4_]_4_	2[ClO_4_]_4_ ⋅ 3MeNO_2_ ⋅ ‐0.75H_2_O
*V*_Oh_ {Fe(1), Fe(2)} [Å^3^]	11.912(9), 11.496(9)	11.642(9), 11.658(9)
Fe ⋅ ⋅ ⋅ Fe [Å]	5.1401(6)	5.1906(6)
*Σ*{Fe(1), Fe(2)} [°]	225.4(3), 213.0(3)	216.7(3), 205.6(3)
*Θ*{Fe(1), Fe(2)} [°]	466, 459	464, 584
*ϕ*{Fe(1), Fe(2)} [°]	148.68(10), 151.50(9)	148.18(9), 153.60(9)
*θ*{Fe(1), Fe(2)} [°]	52.71(5), 56.30(4)	50.96(3), 61.08(2)

The N atom of the central pyridyl ring of each ligand is oriented towards an open face of an iron atom, but at a distance of Fe ⋅ ⋅ ⋅ N=3.1–3.2 Å which is too long for a significant covalent interaction. This coordination geometry implies the **2X_4_** salts should remain high‐spin on cooling (Figure 2), which was confirmed by magnetic measurements (Figure S32).

The helicate structure in [Fe_2_(μ−*L* ^2^)_2_]^4+^ is further stabilized by one short and two longer intramolecular π ⋅ ⋅ ⋅ π interactions, between aromatic residues on each ligand (Figures S29–S30, Table S6). An additional intermolecular π ⋅ ⋅ ⋅ π interaction in both crystals associates the helicate cations into centrosymmetric dimers (the crystals are racemic, containing equal numbers of Λ and Δ helical molecules in their asymmetric unit).

Reaction of *L* ^3^ with 1 equiv. of the same iron(II) salts yielded glassy orange solids, which were perfectly amorphous by powder diffraction. These solids analyzed reasonably to the empirical formulae [Fe(*L* ^3^)]X_2_ (**3X_2_**; X^−^=BF_4_^−^ or ClO_4_^−^), with some included lattice solvent. The amorphous materials are essentially high‐spin at room temperature, and show very gradual SCO equilibria on cooling in ca. 15 % of their iron centers (Figures S33–S34).

**Figure 5:**
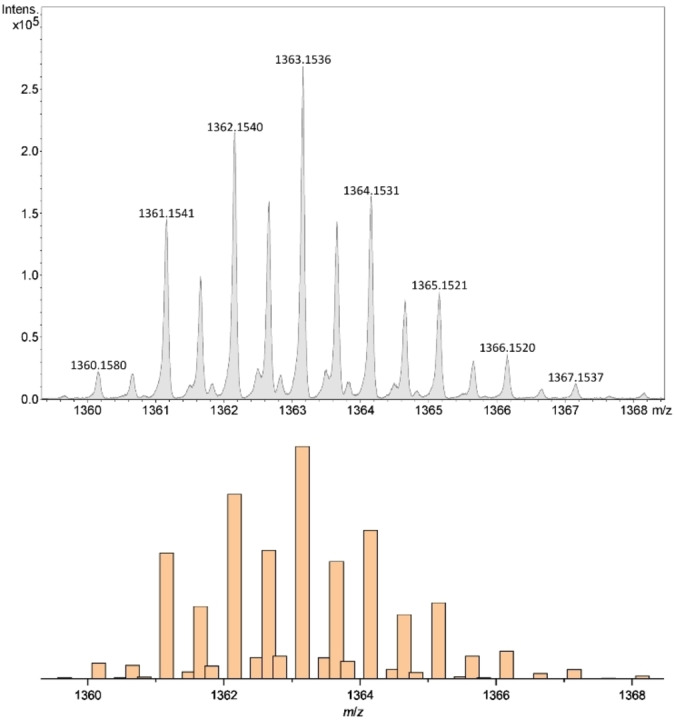
Top: expansion of the principal high mass peak in the electrospray mass spectrum of **1[ClO_4_]_4_** from MeCN solution. Bottom: simulation of the peak as a 0.3:0.6:0.1 mixture of [Fe_2_(*L* ^1^)_2_(ClO_4_)_3_]^+^, [Fe_4_(*L* ^1^)_4_(ClO_4_)_6_]^2+^ and [Fe_6_(*L* ^1^)_6_(ClO_4_)_9_]^3+^.

Electrospray mass spectra of **1[ClO_4_]_2_**, **2[ClO_4_]_2_** and **3[ClO_4_]_2_** from MeCN solution are superficially similar, with an intense peak assigned to [FeL(ClO_4_)]^+^ (L=*L* ^1^−*L* ^3^) and one principal peak at higher mass (L=*L* ^1^, *m*/*z*=1363.1526; L=*L* ^2^, *m*/*z*=1405.0681; L=*L* ^3^, *m*/*z*=1459.1513). Unexpectedly however, simulations of those dication peaks reveal they arise from a combination of [Fe_2_L_2_(ClO_4_)_3_]^+^, [Fe_4_L_4_(ClO_4_)_6_]^2+^ and, for **1[ClO_4_]_2_**, [Fe_6_(*L* ^1^)_6_(ClO_4_)_9_]^3+^ species (Figures 5, S36 and S37).[^52^, ^53^] Weak higher mass peaks from pentameric and hexameric assemblies are also visible for **3[ClO_4_]_2_**. Hence, solutions of all three complexes are a mixture of assembly structures under these conditions. The spectrum of **1[ClO_4_]_2_** shows more fragmentation than the other complexes, including metal‐free *L* ^1^ which is not present in the other spectra. That is consistent with the higher lability of **1[ClO_4_]_2_** observed by NMR (see below).

The ^1^H NMR of **1[ClO_4_]_2_** in CD_3_CN shows just one paramagnetic, *C*_2_‐symmetric *L* ^1^ environment (Figure S38). The butanediyl CH_2_ groups are diastereotopic in the spectrum, which is consistent with the chirality of the helicate structure. In contrast the ^1^H NMR of **2[ClO_4_]_2_** in CD_3_CN contains one principal *C*_2_‐symmetric *L* ^2^ environment, but with a second paramagnetic *L* ^2^‐containing species comprising 10–15 % of the sample by integration (Figure S39). Neither spectrum has peaks in the diamagnetic region from uncoordinated ligand, or dangling bpp residues from mono‐coordinated *L* ^1^ or *L* ^2^. These data imply interconversion of the assembly structures detected by mass spectrometry occurs rapidly in solution when L=*L* ^1^, giving a time‐averaged NMR spectrum, but is slower than the NMR timescale when L=*L* ^2^.

The ^1^H NMR spectrum of **3[ClO_4_]_2_** in CD_3_CN is complex, with multiple paramagnetic *L* ^3^ environments. The spectrum in (CD_3_)_2_CO is simpler, however, with three main *L* ^3^‐containing species being present (Figure S40). Hence, the composition of assembly structures in solutions of **3[ClO_4_]_2_** may be solvent‐dependent. Be that as it may, solutions of **3[ClO_4_]_2_** are a more complex mixture of assembly structures by NMR, in slow chemical exchange. That should explain the amorphous nature of the materials formed by those complexes in the solid state.

Gas phase DFT calculations were undertaken to investigate the influence of the different conformations exhibited by **1[BF_4_]_2_** on its spin state (Figure 1); and, to probe the stability of the helicate structures more generally.[^54^, ^55^] The calculations employed the B86PW91 functional and def2‐SVP basis set, which we have used successfully in comparative studies of the spin states in mononuclear iron(II) complexes of bpp derivatives or related ligands.[^56^, ^57^, ^58^, ^59^] Closely related GGA functionals have also performed well in surveys of functionals for SCO systems.^[60]^

Conformations (a)–(c) of [Fe_2_(μ−*L* ^1^)_2_]^4+^ (Figure 1), and [Fe_2_(μ−*L* ^2^)_2_]^4+^, were minimized in their low‐spin (LS, *S*=0), mixed‐spin (MS, *S*=2) and fully high‐spin (HS, *S*=4) states. The spin states of mononuclear [Fe*L* ^1^]^2+^ were also minimized, for comparison. These calculations highlighted unexpected anomalies. Firstly, [Fe_2_(μ−*L* ^1^)_2_]^4+^ was computed to be at least +100 kcal mol^−1^ higher energy than [Fe*L* ^1^]^2+^ (per mole of dimer), implying the helicate complex should not exist (Table S8). Secondly, the energy difference between the high‐spin and low‐spin states [Δ*E*{HS−*L*S}, Table 3] of [Fe_2_(μ−*L* ^1^)_2_]^4+^ is ca. 3x larger than for 2 equiv. of [Fe*L* ^1^]^2+^. That suggests the low‐spin state of the helicates is over‐stabilized by the calculation. Lastly, the Fe ⋅ ⋅ ⋅ Fe distances in minimized [Fe_2_(μ−*L* ^1^)_2_]^4+^ are consistently 0.4–1.2 Å longer than the crystallographic values; for [Fe_2_(μ−*L* ^2^)_2_]^4+^, the difference is 2.3 Å (Tables S10 and S11). All these observations can be explained, if the calculations are influenced by intramolecular electrostatic repulsion between the Fe^2+^ ions in the dinuclear complexes.^[14]^ Such electrostatic effects could be significant in the gas phase but should be reduced in condensed phases, by ion pairing and dipolar interactions to a solvent shell or crystal lattice.

**Table 3:** Computed energies of the high‐spin (HS, *S*=4), mixed‐spin (MS, *S*=2) and low‐spin (LS, *S*=0) states of the iron complexes in this work. Energies of the corresponding chromium complex minimizations are listed in Table S9.

	*E*(HS) [Ha]	*E*(MS) [Ha]	*E*(LS) [Ha]	ΔE{HS−*L*S} [kcal mol^−1^]	ΔE_rel_{HS−*L*S} [kcal mol^−1^]^[a]^	ΔE{HS−*L*S, MS} [kcal mol^−1^]^[b]^
[Fe*L* ^1^]^2+^	−2815.484288	–	−2815.490595	+4.0	–	–
[Fe_2_(μ−*L* ^1^)_2_]^4+^, conformation (a)	−5630.785068	−5630.802870	−5630.820271	+22.1	0	+0.1
[Fe_2_(μ−*L* ^1^)_2_]^4+^, conformation (b)	−5630.786168	−5630.802732	−5630.819907	+21.2	−0.9	−0.2
[Fe_2_(μ−*L* ^1^)_2_]^4+^, conformation (c)	−5630.788009	−5630.802622	−5630.818614	+19.2	−2.9	−0.4
[Fe_2_(μ−*L* ^2^)_2_]^4+^	−5810.391891	−5810.402230	−5810.409082	+10.8	−11.3	+1.1
[Fe_2_(μ−*L* ^3^)_2_]^4+^	−5935.545352	−5935.560026	−5935.572551	+17.1	−5.0	+0.7

[a] A positive Δ*E*_rel_{HS−*L*S} means the low‐spin state is more stable than for conformation (a) of [Fe_2_(μ−*L* ^1^)_2_]^4+^ (M=Fe^2+^ or Cr^0^), and *vice versa*. [b] A positive Δ*E*{HS−*L*S, MS} means the mixed‐spin state is more stable than an equimolar mixture of high‐spin and low‐spin molecules, and vice versa.

Since a solvent correction could not be included in our calculations,^[54]^ this anomaly was addressed by gas‐phase minimizations of the isoelectronic charge‐neutral molecules [Cr_2_(μ−*L*)_2_]^0^ and [CrL]^0^ (Tables S8‐S9). The computed structures of the low‐spin chromium complexes agree with expectation. However mixed‐spin or high‐spin chromium(0) minimizations yielded results that are more consistent with chromium(II) centers coordinated to [L⋅]^−^ ligand radicals. That was evidenced by their chromium coordination geometries, which are strongly Jahn‐Teller‐elongated (Tables S12–S13); and, by their *α* and *β* HOMO orbitals, which are ligand‐centered in these chromium minimizations but metal‐centered in their iron(II) counterparts (Figures S46–S47).^[55]^ Within that generalization, differences between the computed high‐spin chromium centers suggest additional subtleties, which are beyond the scope of this study.^[61]^ Because of these ambiguities, only the minimizations of the low‐spin chromium complexes are analyzed in detail.

Despite these complications, some conclusions can be drawn from the analysis. In contrast to its iron analogue, low‐spin [Cr_2_(μ−*L* ^1^)_2_]^0^ is computed to be −27 kcal mol^−1^ lower energy than mononuclear [Cr*L* ^1^]^0^, which now agrees with experiment. The Δ*E*{HS−*L*S} energies of [Cr*L* ^1^]^0^ and [Cr_2_(μ−*L* ^1^)_2_]^0^ are also more consistent with each other, than for the iron complexes (Table S8). Lastly, the metal ⋅ ⋅ ⋅ metal distances in each low‐spin [M_2_(μ−*L*)_2_]^2*z*+^ complex are 0.4‐0.6 Å shorter when M=Cr(0) than when M=Fe(II) (Tables S10‐S13). All these observations imply electrostatic repulsion between the iron atoms is indeed an important factor in our gas phase minimizations of [Fe_2_(μ−*L*)_2_]^4+^.

The three conformations of the iron complex, in a given spin state, are within 2 kcal mol^−1^ of each other by this protocol. The difference is smaller for low‐spin [Cr_2_(μ−*L* ^1^)_2_]^0^, where conformations (a)‐(c) lie within 0.7 kcal mol^−1^ (Table S9). Hence, all these conformations should be thermally accessible at room temperature, as observed. More detailed discussion of the minimized structures is not justified however, because of the ambiguities noted above.

The absolute spin state energies from a protocol like this are inaccurate, so the Δ*E*_rel_{HS−*L*S} energies in Tables 3 and S9 are scaled relative to conformation (a) of the relevant [M_2_(μ−*L* ^1^)_2_]^2*z*+^ molecule. A molecule with a positive Δ*E*_rel_{HS−*L*S} has a more stable low‐spin state than conformation (a), and so should exhibit a higher *T*_1/2_ value. Similarly, a negative Δ*E*_rel_{HS−*L*S} implies *T*_1/2_ should be lower than conformation (a). By this measure, *T*_1/2_ for the conformations of [Fe_2_(μ−*L* ^1^)_2_]^4+^ should follow the trend of (a)>(b)>(c). That is broadly consistent with the crystallographic and magnetic properties of the **1[BF_4_]_2_** solvates (Table 1, Figure 3).

The mixed‐spin forms of [Fe_2_(μ−*L* ^1^)_2_]^4+^ have almost identical energy to an equimolar mixture of high‐spin and low‐spin molecules, to within 0.5 kcal mol^−1^ (Δ*E*{HS−*L*S, MS}, Table 3). Thus, the mixed‐spin form of **1[BF_4_]_2_** is not intrinsically electronically favored. The stepwise SCO in **1[BF_4_]_4_** ⋅ *n*Me_2_CO, and the incomplete SCO in **1[BF_4_]_4_** ⋅ 2MeCN ⋅ Et_2_O, should therefore be a consequence of solid state packing effects.

**Figure 6:**
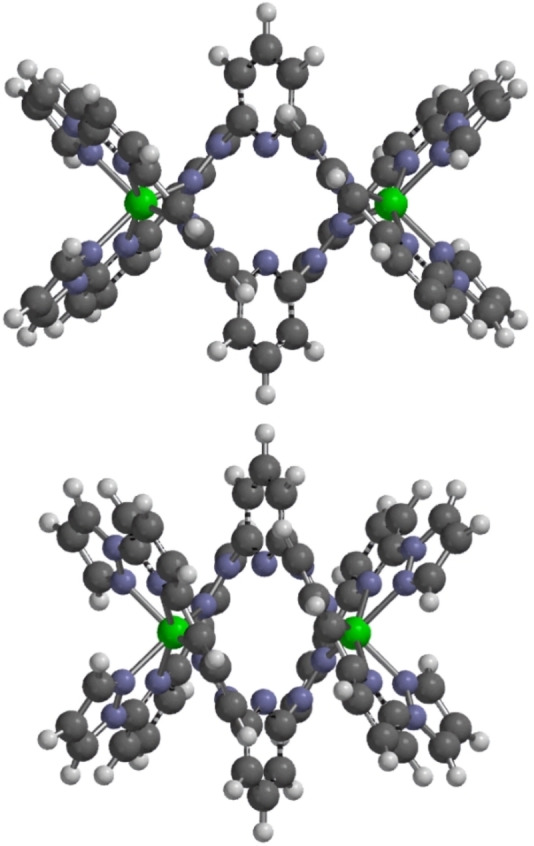
Computed structures of [Fe_2_(μ−*L* ^2^)_2_]^4+^. Top: DFT energy minimization of the high‐spin (*S*=4) iron complex (Fe ⋅ ⋅ ⋅ Fe=7.418 Å). Bottom: molecular mechanics geometry minimization (M ⋅ ⋅ ⋅ M=5.356 Å). The molecular mechanics calculation is closer to the experimental structure (Table 2, Figure 4). Color code: C, gray; H, white; N, blue; Fe or M, green.

[Fe_2_(μ−*L* ^2^)_2_]^4+^ is computed with a large, negative Δ*E*_rel_{HS−*L*S} value, showing it is strongly high‐spin as observed. However, all the spin states of [M_2_(μ−*L* ^2^)_2_]^2*z*+^ (M^*z*+^=Fe^2+^ or Cr^0^) minimized to a more symmetric *L* ^2^ ligand conformation than found crystallographically for **2[ClO_4_]_2_**. This places the metal atoms further apart, and with a less distorted coordination geometry than found experimentally.^[55]^ Since the structure should reflect both the conformational preferences of *L* ^2^ and ligand field effects on the metal geometry, our DFT protocol may over‐estimate the ligand field contribution to the structure of this molecule. Consistent with that, molecular mechanics minimizations of [M_2_(μ−*L* ^2^)_2_]^2*z*+^, which exclude ligand field considerations, reproduced the experimental conformation of **2[ClO_4_]_2_** more accurately (Figure 6).

Minimizations of the [M_2_(μ−*L* ^3^)_2_]^2*z*+^ helicate were also investigated.^[55]^ These also revealed conformational flexibility in the xylyl linker group, placing the metal atoms in the low‐spin iron complex 0.9 Å further apart than in the chromium compound (Figure S48). MM2 minimizations reproduced the chromium complex conformation, so the extended conformation of [Fe_2_(μ−*L* ^3^)_2_]^4+^ could again reflect electrostatic repulsion between its Fe^2+^ ions. The high‐spin state of [Fe_2_(μ−*L* ^3^)_2_]^4+^ is significantly stabilized compared to [Fe_2_(μ−*L* ^1^)_2_]^4+^ (Table 3), which is consistent with the high‐spin nature of **3X_2_** at room temperature. However, since no crystallographic data are available for the Fe/*L* ^3^ complex, the relevance of these results to its experimental properties is unclear.

## Conclusion

Four new ditopic ligands have been synthesized, by connecting two 1,3‐bpp metal‐binding moieties with different spacers via their distal N−H groups. Two of these, *L* ^1^ and *L* ^2^, cleanly afford [2+2] helicate complexes when complexed to iron(II). Three helical conformations of [Fe_2_(μ−*L* ^1^)_2_]^4+^ were observed in different solvate crystals of **1[BF_4_]_4_** (Figure 1), which exhibit a range of spin state properties. These include a clear, unusual stepwise SCO of the two iron centers in **1[BF_4_]_4_** ⋅ *n*Me_2_CO, via a mixed‐spin intermediate which was detected crystallographically (Figure 3).^[62]^ The DFT calculations imply conformations (a)‐(c) of [Fe_2_(μ−*L* ^1^)_2_]^4+^ should all exist in solution, while NMR showed they are in rapid chemical exchange at room temperature. Hence, the observation of different helicate conformations in crystals of **1[BF_4_]_4_** should simply reflect the crystal packing in each solvate.

In contrast, salts of [Fe_2_(μ−*L* ^2^)_2_]^4+^ remain high‐spin at all temperatures. That is explained by the molecular conformation shown by **2[ClO_4_]_4_**, which leads to the most distorted six‐coordinate geometries yet observed in the extended family of [Fe(bpp)_2_]^2+^ SCO materials (Figures 2 and 4). Analytically pure iron(II) complexes of *L* ^3^ were also obtained, which are however completely amorphous in the solid state. Hence, the molecular structures of **3[BF_4_]_2_** and **3[ClO_4_]_2_** are uncertain.

Solutions of **1[ClO_4_]_4_**, **2[ClO_4_]_4_** and **3[ClO_4_]_2_** contain both [Fe_2_(μ−*L*)_2_]^4+^ and [Fe_4_(μ−*L*)_4_]^8+^ (L=*L* ^1^−*L* ^3^) by mass spectrometry, with higher nuclearity species also being present in some cases (Figures 5 and S35–S37). Hence, the helicate complexes exist in equilibrium with other assembly structures in solution. That being the case, ^1^H NMR implies those assemblies interconvert in solution more rapidly when L=*L* ^1^ than for the more rigid L=*L* ^2^ or *L* ^3^. Hence, the identity of the spacer group strongly influences the composition and dynamics of the supramolecular assemblies formed by *L* ^1^−*L* ^3^.

Gas phase DFT calculations confirm conformations (a)–(c) of [Fe_2_(μ−*L* ^1^)_2_]^4+^ have almost identical energies, but should show detectably different spin state properties. These appear consistent with experiment, in that the computed spin state energies mirror the observed trend in solid state SCO temperatures, of conformation (a)>(b)>(c) (Figure 3, Table 3). The calculations also reproduce the high‐spin nature of [Fe_2_(μ−*L* ^2^)_2_]^4+^. However in other respects the calculations present anomalies, which are consistent with the influence of intramolecular electrostatic repulsion between the positively charged iron ions. Such effects would be compensated in condensed phases, by ion pairing and weaker intermolecular dipolar interactions with the surrounding medium. That could lead to significant discrepancies between the results of our single point calculations and experiment, as observed. Further calculations of isoelectronic [Cr_2_(μ−*L* ^1^)_2_]^0^ and [Cr_2_(μ−*L* ^2^)_2_]^0^ support that view, but were themselves only partly successful because the high‐spin chromium(0) centers undergo valence tautomeric oxidation to chromium(II) in silico.

Other gas phase DFT studies on dinuclear^[63]^ or higher nuclearity[^14^, ^53^, ^64^] SCO complexes have investigated more conformationally rigid molecules, many of which are electroneutral. The influence of intramolecular electrostatic effects on the calculations should be less apparent in those cases. However, a recent gas phase DFT study of Fe_4_ grid complexes noted that the high‐spin states of more highly charged molecules in that study were over‐stabilized computationally, compared to their uncharged analogues.^[14]^ Electrostatic interactions between the iron atoms in those molecules were proposed to contribute to that discrepancy. In contrast, the low‐spin state of the dinuclear complexes appears to be overstabilized in this work (Tables 3 and S9), although we base that observation on different criteria from those in Ref. [14]. In other respects, our results are consistent with the conclusions from that earlier study.^[65]^

## Experimental Section

**Instrumentation**: Solid state magnetic susceptibility measurements were performed with freshly isolated, unground polycrystalline samples, using a Quantum Design MPMS‐3 SQUID/VSM magnetometer in an applied field of 5000 G. Samples were protected against solvent loss by saturating the tightly sealed MPMS‐3 powder capsules with diethyl ether vapor, Unless otherwise specified, the measurements employed a temperature ramp of 5 Kmin^−1^. Diamagnetic corrections for the samples were estimated from Pascal's constants;^[66]^ a previously measured diamagnetic correction for the sample holder was also applied to the data.

Elemental microanalyses were performed by the microanalytical services at London Metropolitan University School of Human Sciences. Electrospray mass spectra were recorded on a Bruker MicroTOF‐q instrument from chloroform (organic ligands) or acetonitrile (metal complexes) solution. The peak simulations in Figures 5, S36 and S37 were plotted with *ORIGIN*,^[67]^ starting from simulations of the individual component species produced by *Bruker Compass*.^[68]^ Diamagnetic NMR spectra employed a Bruker AV3HD spectrometer operating at 400.1 (^1^H) or 100.6 MHz (^13^C), while paramagnetic ^1^H NMR spectra were obtained with a Bruker AV3 spectrometer operating at 300.1 MHz. X‐ray powder diffraction measurements were obtained at room temperature from a Bruker D2 Phaser diffractometer, using Cu‐*K*_*α*_ radiation (*λ*=1.5419 Å).

All calculations were performed by using SPARTAN’18.^[69]^ DFT calculations employed the B86PW91 functional and def2‐SVP basis set. Low‐spin systems were treated as spin restricted and high‐spin systems as spin unrestricted. The calculations were performed in the gas phase, since a solvent gradient for iron is not implemented in SPARTAN’18. Crystallographic atomic coordinates for the different conformations of **1^2+^**, and for **2^2+^**, were used as a starting point for those geometry minimizations. Otherwise, initial models were constructed de novo in the program, then subjected to a preliminary molecular mechanics minimization before the full DFT energy minimization was undertaken.

Molecular mechanics (MM) structures were calculated in SPARTAN’18^[69]^ for the chromium complexes [Cr_2_(μ−*L* ^1^)_2_]^0^ and [Cr_2_(μ−*L* ^2^)_2_]^0^, since the atomic radius of chromium in the MM minimization protocol resembles that of high‐spin iron(II) [Cr−N ca. 2.2 Å]. This was preferred over analogous MM calculations using the Fe_2_ helicate molecules, which yielded unrealistically short Fe−N distances [Fe−N ca. 1.8 Å].

**Materials and Methods**: Synthetic protocols and characterization data for *L* ^1^−*L* ^4^ and the other new compounds in Scheme 1, are given in the Supporting Information.

CAUTION We experienced no problems when using the perchlorate salts in this study. However, metal‐organic perchlorates are potentially explosive and should be handled with care in small quantities.

**Synthesis of [Fe_2_(μ**−***L*** ^**1**^ **)_2_][BF_4_]_4_ (1[BF_4_]_4_)**: A mixture of *L* ^1^ (0.20 g, 0.42 mmol) and Fe[BF_4_]_2_ ⋅ 6H_2_O (0.14 g, 0.42 mmol) in nitromethane (25 cm^3^) was stirred at room temperature for 1 hr. A small quantity of brown precipitate was removed by filtration, and the dark yellow filtrate was concentrated to half its original volume. Slow diffusion of diethyl ether vapor into the filtered solution afforded an orange polycrystalline solid, which turned brown when dried *in vacuo*. Yield 0.19 g, 64 %. Elemental analysis calcd (%) for C_52_H_48_B_4_F_16_Fe_2_N_20_ ⋅ 2CH_3_NO_2_ ⋅ 2H_2_O C 41.3, H 3.72, N 19.6; found C 41.1, H 3.29, N 19.3.

**Synthesis of [Fe_2_(μ**−***L*** ^**1**^ **)_2_][ClO_4_]_4_ (1[ClO_4_]_4_)**: Method as for **1[BF_4_]_2_**, using Fe[ClO_4_]_2_ ⋅ 6H_2_O (0.15 g, 0.42 mmol). The product was an orange polycrystalline solid, which turned brown upon drying. Yield 0.15 g, 49 %. ^1^H NMR (CD_3_CN): *δ*=−13.8 (4H), −10.6 (4H), −7.0 (4H), 5.6 (4H), 12.4 (4H), 23.8 (4H), 41.8 (4H), 44.2 (4H), 56.7 (4H), 60.3 (4H), 69.8 (4H), 71.0 (4H); HRMS (ESI): *m*/*z* calcd for C_26_H_24_N_10_+Na^+^: 499.2083 [*L* ^1^+Na]^+^; found: 499.2023; calcd for C_26_H_24_ClFeN_10_O_4_^+^: 631.1020 [Fe(*L* ^1^)ClO_4_]^+^; found: 631.0929; calcd for {C_52_H_48_Cl_3_Fe_2_N_20_O_12_}_z_^*z*+^: 1363.1525 [Fe_2*z*_(*L* ^1^)_2*z*_(ClO_4_)_3*z*_]^*z*+^; found: 1363.1526. The peak at *m*/*z* 1363.1526 is an overlay of monocation (*z*=1), dication (*z*=2) and trication (*z*=3) molecular ions; elemental analysis calcd (%) for C_52_H_48_Cl_4_Fe_2_N_20_O_16_ ⋅ CH_3_NO_2_ ⋅ H_2_O C 41.3, H 3.47, N 19.1; found C 41.0, H 3.15, N 18.9.

**Synthesis of [Fe_2_(μ**−***L*** ^**2**^ **)_2_][BF_4_]_4_ (2[BF_4_]_4_)**: A mixture of crude *L* ^2^ (0.20 g, 0.40 mmol) and Fe[BF_4_]_2_ ⋅ 6H_2_O (0.14 g, 0.40 mmol) in nitromethane (25 cm^3^) was stirred with mild heating, until all the solid had dissolved. The bright yellow solution was filtered and concentrated to ca. 5 cm^3^ volume. Slow diffusion of diethyl ether vapor into the filtered solution afforded a yellow crystalline solid, which decomposes to a yellow powder on exposure to air. Yield 0.21 g, 72 %. Elemental analysis calcd (%) for C_54_H_38_B_4_F_16_Fe_2_N_22_ ⋅ H_2_O 44.1, H 2.74, N 20.9; found C, 44.2, H, 2.43, N, 20.6.

**Synthesis of [Fe_2_(μ**−***L*** ^**2**^ **)_2_][ClO_4_]_4_ (2[ClO_4_]_4_)**: Method as for **2[BF_4_]_2_**, using Fe[ClO_4_]_2_ ⋅ 6H_2_O (0.15 g, 0.40 mmol). The product was a yellow polycrystalline solid. Yield 0.22 g, 73 %. ^1^H NMR (CD_3_CN): *δ*=−2.3 (4H), 0.9 (2H), 18.6 (4H), 36.1 (4H), 55.7 (4H), 56.7 (4H), 58.9 (4H), 68.8 (8H), 76.4 (4H). At least one other paramagnetic *L* ^2^‐containing species is also present in the spectrum, with 10–15 % of the integral compared to the main component; HRMS (ESI): *m*/*z* calcd for C_54_H_38_FeN_22_^2+^: 525.1495 [Fe(*L* ^2^)_2_]^2+^; found: 525.1571; calcd for {C_27_H_19_ClFeN_11_O_4_}_z_^*z*+^: 652.0659 [Fe_*z*_(*L* ^2^)_*z*_(ClO_4_)_*z*_]^*z*+^; found: 652.0635; calcd for C_27_H_19_Cl_3_Fe_2_N_11_O_12_^+^: 905.8979 [Fe_2_(*L* ^2^)(ClO_4_)_3_]^+^; found: 905.8891; calcd for C_54_H_38_ClFeN_22_O_4_^+^: 1149.2484 [Fe(*L* ^2^)_2_ClO_4_]^+^; found: 1149.2385; calcd for {C_54_H_38_Cl_3_Fe_2_N_22_O_12_}_z_^*z*+^: 1405.0775 [Fe_2*z*_(*L* ^2^)_2*z*_(ClO_4_)_3*z*_]^*z*+^; found: 1405.0681. The peaks at *m*/*z* 652.0635 and 1405.0681 are overlays of monocation (*z*=1) and dication (*z*=2) molecular ions; elemental analysis calcd (%) for C_54_H_38_Cl_4_Fe_2_N_22_O_16_ C 43.1, H 2.55, N 20.5; found C 43.2, H 2.50, N 20.3.

**Synthesis of {[Fe(*L*** ^**3**^ **)][BF_4_]_2_}**_***x***_ **⋅** ***x*** **CH_3_NO_2_ (3[BF_4_]_2_** **⋅ CH_3_NO_2_)**: A mixture of *L* ^3^ (0.15 g, 0.29 mmol) and Fe[BF_4_]_2_ ⋅ 6H_2_O (0.10 g, 0.29 mmol) in nitromethane (20 cm^3^) was stirred at room temperature until all the solid had dissolved. A small quantity of brown precipitate was removed by filtration, and the yellow filtrate was concentrated to half its original volume. Slow diffusion of diethyl ether vapor into the filtered solution afforded a glassy orange solid. Yield 0.11 g, 50 %. Elemental analysis calcd (%) for C_30_H_24_B_2_F_8_FeN_10_ ⋅ CH_3_NO_2_ C 45.7, H 3.34, N 18.9; found C 45.2, H 3.21, N 19.1.

**Synthesis of {[Fe(*L*** ^**3**^ **)][ClO_4_]_2_}**_***x***_ **⋅ 1.5*x*H_2_O (3[ClO_4_]_2_** **⋅ 1.5H_2_O)**: Method as for **3[BF_4_]_2_**, using Fe[ClO_4_]_2_ ⋅ 6H_2_O (0.11 g, 0.29 mmol). The product was a glassy orange solid. Yield 0.15 g, 66 %. The ^1^H NMR spectrum of this product is complex, containing multiple paramagnetic iron/*L* ^3^ environments (Figure S40). HRMS (ESI): *m*/*z* calcd for C_30_H_24_ClFeN_10_O_4_^+^: 679.1020 [Fe(*L* ^3^)ClO_4_]^+^; found: 679.0993; calcd for {C_60_H_48_Cl_3_Fe_2_N_20_O_12_}_z_^*z*+^: 1459.1525 [Fe_2*z*_(*L* ^3^)_2*z*_(ClO_4_)_3*z*_]^*z*+^; found: 1459.1513; calcd for C_150_H_120_Cl_8_Fe_5_N_50_O_32_^+^: 1848.6690 [Fe_5_(*L* ^3^)_5_(ClO_4_)_8_]^2+^; found: 1848.6669. The peak at *m*/*z* 1459.1513 is an overlay of monocation (*z*=1) and dication (*z*=2) molecular ions; elemental analysis calcd (%) for C_30_H_24_Cl_2_FeN_10_O_8_ ⋅ 1.5H_2_O C 44.7, H 3.37, N 17.4; found C 44.7, H 3.36, N 16.9.

**Crystal Structure Analyses**: Crystals of 1,3‐bpp were grown by slow evaporation of an NMR sample of the compound in CDCl_3_. The solvent‐free crystals **2[ClO_4_]_4_** were obtained by slow diffusion of diethyl ether vapor into a filtered solution of the complex in acetone. The other solvated crystals were grown similarly, by diethyl ether vapor diffusion in the appropriate solvent. Diffraction data for **1[BF_4_]_4_** ⋅ *n*Me_2_CO were recorded at station I19 of the Diamond synchrotron (*λ*=0.6889 Å). All other diffraction data were measured with an Agilent Supernova dual‐source diffractometer using monochromated Cu‐*K*_*α*_ (*λ*=1.5418 Å) radiation. The diffractometer was fitted with an Oxford Cryostream low‐temperature device.

Crystallographic experimental details and refinement protocols are given in the Supporting Information. All the structures were solved by direct methods *(SHELXS* ^[70]^), and developed by full least‐squares refinement on *F* ^2^ (*SHELXL‐2018* ^[70]^). Crystallographic figures were prepared using *XSEED*,^[71]^ while calculation of structural indices and preparation of publication materials was performed with *Olex2*.^[72]^

Deposition Number(s) 2169630 (*α*‐1,3‐bpp), 2169631 (**1[BF**_**4**_ **]**_**4**_ ⋅ *n*Me_2_CO, *T*=250 K), 2169632 (**1[BF**_**4**_ **]**_**4**_ ⋅ *n*Me_2_CO, *T*=100 K), 2169633 (**1[BF**_**4**_ **]**_**4**_ ⋅ 2MeCN ⋅ Et_2_O), 2169634 (**1[BF**_**4**_ **]**_**4**_ ⋅ *m*MeNO_2_), 2169635 (**1[BF**_**4**_ **]**_**4**_ ⋅ 2MeNO_2_), 2169636 (**2[ClO**_**4**_ **]**_**4**_) and 2169637 (**2[ClO**_**4**_ **]**_**4**_ ⋅ 3MeNO_2_ ⋅ 0.75H_2_O) contain(s) the supplementary crystallographic data for this paper. These data are provided free of charge by the joint Cambridge Crystallographic Data Centre and Fachinformationszentrum Karlsruhe Access Structures service.

## Conflict of interest

The authors declare no conflict of interest.

1

## Supporting information

As a service to our authors and readers, this journal provides supporting information supplied by the authors. Such materials are peer reviewed and may be re‐organized for online delivery, but are not copy‐edited or typeset. Technical support issues arising from supporting information (other than missing files) should be addressed to the authors.

Supporting Information

## Data Availability

Data supporting this study are openly available from the University of Leeds library at https://doi.org/10.5518/1205.
