# Supplementary material for: Di‐Iron(II) [2+2] Helicates of Bis‐(Dipyrazolylpyridine) Ligands: The Influence of the Ligand Linker Group on Spin State Properties
Source: Chemistry. 2022 Dec 27;29(9):e202202578. doi: 10.1002/chem.202202578 (PMC10108139; doi:10.1002/chem.202202578)
Supplement: Supplementary file 1 — Supporting Information [file CHEM-29-0-s001.pdf]

Chemistry–A European Journal

Supporting Information

# Di-Iron(II) [2+2] Helicates of Bis-(Dipyrazolylpyridine) Ligands: The Influence of the Ligand Linker Group on Spin State Properties

Rafal Kulmaczewski, Isaac T. Armstrong, Pip Catchpole, Emily S. J. Ratcliffe, Hari Babu Vasili, Stuart L. Warriner, Oscar Cespedes, and Malcolm A. Halcrow*

| | | Page |
|---|---|---|
| **Experimental – organic ligand synthesis** | | S3 |
| **Scheme S1** | Synthesis of *L*[1]-*L*[3]. | S3 |
| **Scheme S2** | Synthesis of *L*[4] and its byproducts. | S4 |
| **Scheme S3** | Attempted synthesis of 1,2-*bis*(3-{2-[pyrazol-1-yl]pyrid-6-yl}pyrazol-1-yl)ethane. | S5 |
| **Figure S1** | NMR spectra of *L*[1]. | S6 |
| **Figure S2** | NMR spectra of *L*[3]. | S7 |
| **Figure S3** | NMR spectra of *L*[4]. | S8 |
| **Figure S4** | Electrospray mass spectrum of *L*[1]. | S9 |
| **Figure S5** | Electrospray mass spectrum of *L*[2]. | S9 |
| **Figure S6** | Electrospray mass spectrum of *L*[3]. | S10 |
| **Figure S7** | Electrospray mass spectrum of *L*[4]. | S10 |
| **Figure S8** | NMR spectra of 3-(3-{2-[pyrazol-1-yl]pyrid-6-yl}pyrazol-1-yl)prop-1-ene, a byproduct in the synthesis of *L*[4]. | S11 |
| **Figure S9** | NMR spectrum of 5-{2-[pyrazol-1-yl]pyrid-6-yl}-2,3-dihydropyrazolo[1,2-a] pyrazolium iodide, a byproduct in the synthesis of *L*[4]. | S12 |
| **Figure S10** | NMR spectra of 1-(3-{2-[pyrazol-1-yl]pyrid-6-yl}pyrazol-1-yl)-2-iodoethane. | S13 |
| **Experimental – crystallography** | | S14 |
| **Table S1** | Experimental data for the crystal structure determinations. | S16 |
| **Figure S11** | The asymmetric unit of $\alpha$-1,3-bpp. | S18 |
| **Table S2** | Hydrogen bond parameters in the structure of $\alpha$-1,3-bpp. | S18 |
| **Figure S12** | Packing diagram of $\alpha$-1,3-bpp. | S19 |
| **Figure S13** | Partial packing diagram of $\beta$-1,3-bpp. | S19 |
| **Definitions of the structural parameters tabulated for the complexes.** | | S20 |
| **Scheme S4** | Angles used in the definitions of the coordination distortion parameters $\Sigma$ and $\Theta$. | S20 |
| **Scheme S5** | Definition of the Jahn-Teller distortion parameters $\theta$ and $\phi$. | S20 |
| **Figure S14** | The formula unit in the crystal structure of **1[BF$_4$]$_4$**·2MeCN·Et$_2$O. | S21 |
| **Figure S15** | The formula unit in the crystal structure of **1[BF$_4$]$_4$**·*n*Me$_2$CO at 100 K. | S22 |
| **Figure S16** | The formula unit in the crystal structure of **1[BF$_4$]$_4$**·*n*Me$_2$CO at 250 K. | S23 |
| **Figure S17** | The formula unit in the crystal structure of **1[BF$_4$]$_4$**·*m*MeNO$_2$. | S24 |
| **Figure S18** | The formula unit in the crystal structure of **1[BF$_4$]$_4$**·2MeNO$_2$. | S25 |
| **Table S3** | Selected bond lengths and angles for **1[BF$_4$]$_4$**·2MeCN·Et$_2$O and **1[BF$_4$]$_4$**·*n*Me$_2$CO. | S26 |
| **Table S4** | Selected bond lengths and angles for **1[BF$_4$]$_4$**·*m*MeNO$_2$ and **1[BF$_4$]$_4$**·2MeNO$_2$. | S27 |
| **Figure S19** | Space-filling views of the three conformations in crystal structures of $[Fe_2(\mu\text{-}L^1)_2]^{4+}$. | S28 |
| **Figure S20** | Powder diffraction data for **1[BF$_4$]$_4$** and **1[ClO$_4$]$_4$**, crystallized from nitromethane. | S29 |
| **Figure S21** | Powder diffraction data for **1[BF$_4$]$_4$** and **1[ClO$_4$]$_4$**, crystallized from acetone. | S30 |
| **Figure S20** | Powder diffraction data for **1[BF$_4$]$_4$** and **1[ClO$_4$]$_4$**, crystallized from acetonitrile. | S31 |
| **Figure S23** | Magnetic data for **1[BF$_4$]$_4$** and **1[ClO$_4$]$_4$**, crystallized from nitromethane solution. | S32 |
| **Figure S24** | Magnetic data for **1[BF$_4$]$_4$** and **1[ClO$_4$]$_4$**, crystallized from acetone solution. | S32 |
| **Figure S25** | Magnetic data for **1[BF$_4$]$_4$** and **1[ClO$_4$]$_4$**, crystallized from acetonitrile solution. | S33 |
| **Figure S26** | The asymmetric unit of **2[ClO$_4$]$_4$**. | S34 |

| | | Page |
|---|---|---|
| **Figure S27** | The asymmetric unit of **2[ClO$_4$]$_4$**·3MeNO$_2$·0.75H$_2$O. | S35 |
| **Figure S28** | The formula unit in a partial crystallographic refinement of **2[BF$_4$]$_4$**·MeNO$_2$·Et$_2$O. | S36 |
| **Table S5** | Selected bond lengths and angles for **2[ClO$_4$]$_4$** and **2[ClO$_4$]$_4$**·3MeNO$_2$·0.75H$_2$O. | S37 |
| **Figure S29** | Space-filling view of $[Fe_2(\mu\text{-}L^2)_2]^{4+}$. | S38 |
| **Table S6** | $\pi\cdots\pi$ Interactions in the crystal structures of **2[ClO$_4$]$_4$** and **2[ClO$_4$]$_4$**·3MeNO$_2$·0.75H$_2$O. | S38 |
| **Figure S30** | $\pi\cdots\pi$ Interactions in the crystal structures of **2[ClO$_4$]$_4$** and **2[ClO$_4$]$_4$**·3MeNO$_2$·0.75H$_2$O. | S39 |
| **Figure S31** | Powder diffraction pattern for **2[ClO$_4$]$_4$**. | S40 |
| **Figure S32** | Magnetic data for **2[BF$_4$]$_4$** and **2[ClO$_4$]$_4$**. | S40 |
| **Figure S33** | Powder diffraction pattern for **3[BF$_4$]$_4$** and **3[ClO$_4$]$_4$**. | S41 |
| **Figure S34** | Magnetic data for **3[BF$_4$]$_4$** and **3[ClO$_4$]$_4$**. | S41 |
| **Figure S35** | Electrospray mass spectrum of **1[ClO$_4$]$_4$**. | S42 |
| **Figure S36** | Electrospray mass spectrum of **2[ClO$_4$]$_4$**. | S43 |
| **Figure S37** | Electrospray mass spectrum of **3[ClO$_4$]$_4$**. | S44 |
| **Figure S38** | $^1$H NMR spectrum of **1[ClO$_4$]$_4$**. | S45 |
| **Figure S39** | $^1$H NMR spectrum of **2[ClO$_4$]$_4$**. | S46 |
| **Figure S40** | $^1$H NMR spectrum of **3[ClO$_4$]$_2$**. | S47 |
| **Figure S41** | Views of energy-minimized $[ML^1]^{z+}$ ($M^{z+} = Fe^{2+}$ or $Cr^0$). | S48 |
| **Scheme S6** | Atom numbering used in Table S7. | S48 |
| **Table S7** | Computed metric parameters for $[ML^1]^{z+}$ ($M^{z+} = Fe^{2+}$ or $Cr^0$). | S49 |
| **Table S8** | Computed minimized energies of $[ML^1]^{z+}$ ($M^{z+} = Fe^{2+}$ or $Cr^0$). | S49 |
| **Table S9** | Minimized energies of the spin states of the complexes computed in this work. | S50 |
| **Figure S42** | Views of the three conformations of energy-minimized $[Fe_2(\mu\text{-}L^1)_2]^{4+}$. | S51 |
| **Figure S43** | Views of $[Fe_2(\mu\text{-}L^2)_2]^{4+}$, minimized in its high-spin, mixed-spin and low-spin states. | S52 |
| **Scheme S7** | Atom numbering used in the Tables of metric parameters from the DFT minimizations for $[M_2(\mu\text{-}L^1)_2]^{2z+}$, $[M_2(\mu\text{-}L^2)_2]^{2z+}$ and $[M_2(\mu\text{-}L^3)_2]^{2z+}$ ($M^{z+} = Fe^{2+}$ or $Cr^0$). | S52 |
| **Table S10** | Computed metric parameters for the different helicate conformations of $[Fe_2(\mu\text{-}L^1)_2]^{4+}$. | S53 |
| **Table S11** | Computed metric parameters for $[Fe_2(\mu\text{-}L^2)_2]^{4+}$. | S56 |
| **Figure S44** | Views of the three conformations of energy-minimized $[Cr_2(\mu\text{-}L^1)_2]^0$. | S57 |
| **Figure S45** | Views of energy-minimized $[Cr_2(\mu\text{-}L^2)_2]^0$. | S58 |
| **Discussion of the chromium complex minimizations** | | S58 |
| **Table S12** | Computed metric parameters for the different helicate conformations of $[Cr_2(\mu\text{-}L^1)_2]^0$. | S59 |
| **Table S13** | Computed metric parameters for $[Cr_2(\mu\text{-}L^2)_2]^0$. | S62 |
| **Figure S46** | Spin-unrestricted frontier orbitals of the mixed-spin ($S$ = 2) state of $[Fe_2(\mu\text{-}L^1)_2]^{4+}$. | S63 |
| **Figure S47** | Spin-unrestricted frontier orbitals of the mixed-spin ($S$ = 2) state of $[Cr_2(\mu\text{-}L^1)_2]^0$. | S64 |
| **Figure S48** | Views of energy-minimized $[Fe_2(\mu\text{-}L^3)_2]^{4+}$ and $[Cr_2(\mu\text{-}L^3)_2]^0$. | S65 |
| **Table S14** | Computed metric parameters for $[Fe_2(\mu\text{-}L^3)_2]^{4+}$ and $[Cr_2(\mu\text{-}L^3)_2]^0$. | S66 |
| **Table S15** | Computed atomic coordinates for the DFT-minimized molecules in this work. | S68 |
| **References** | | S134 |

## Experimental

### Materials and Methods

The ligand precursor 2-[pyrazol-1-yl]-6-[1*H*-pyrazol-3-yl]pyridine (1,3-bpp) was prepared by the literature procedure.[1] Other reagents were purchased commercially and used as supplied. Synthetic protocols and characterization data for the metal complexes in this work are given in the main article.

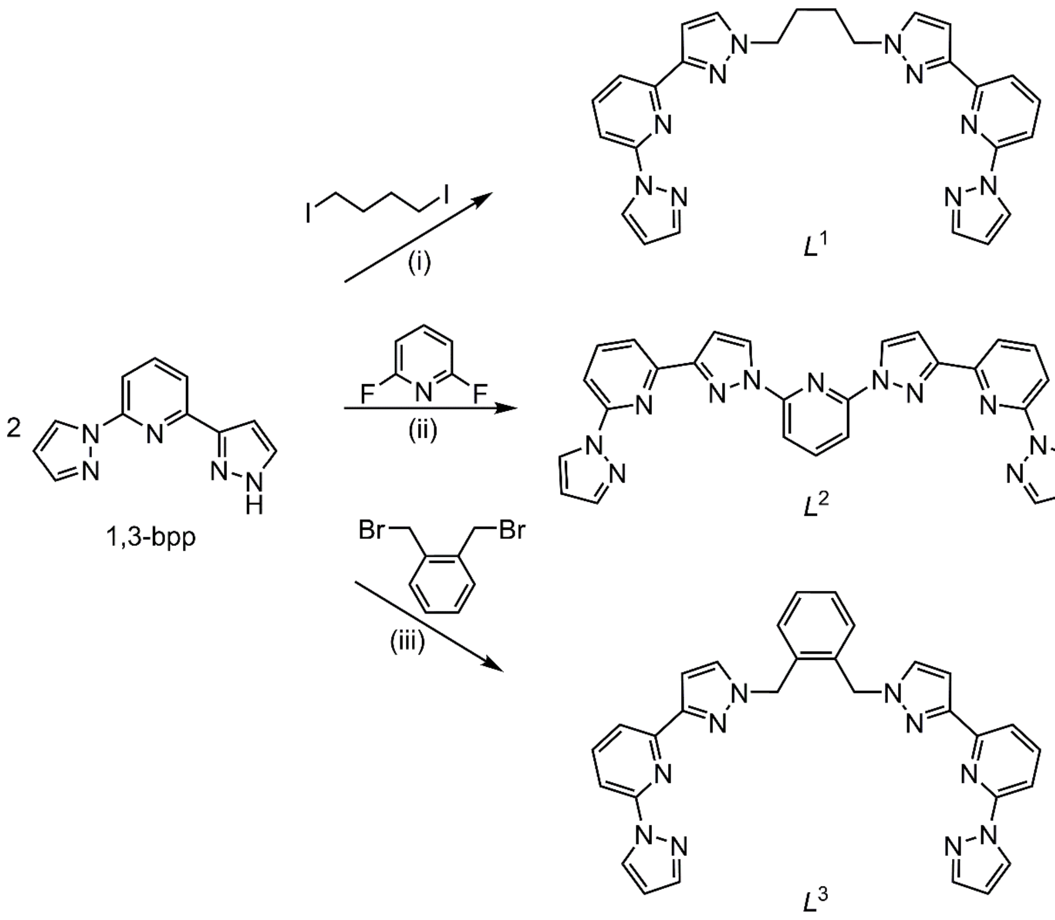


**Scheme S1** Synthesis of $L^1$-$L^3$. Reagents and conditions: (i) NaH, thf, 298 K then 0.5 eq 1,4-diiodobutane, reflux, 72 hrs; (ii) NaH, dmf, 298 K then 0.5 eq 2,6-difluoropyridine, reflux, 24 hrs; (iii) NaH, thf, 298 K then 0.5 eq 1,2-*bis*(bromomethyl)benzene, reflux, 72 hrs.

**Synthesis of 1,4-*bis*(3-{2-[pyrazol-1-yl]pyrid-6-yl}pyrazol-1-yl)butane ($L^1$).** 1,3-bpp (0.50 g, 2.36 mmol) was dissolved in tetrahydrofuran (25 $cm^3$) under a nitrogen atmosphere, then sodium hydride (60 wt % suspension in mineral oil; 0.10 g, 2.60 mmol) was added. After the effervescence ceased, the mixture was heated to reflux. 1,4-Diiodobutane (0.37 g, 1.18 mmol) was then added in one portion, and the mixture was stirred at reflux for 4 days. After cooling, $L^1$ precipitated from the mixture as a white powder which was collected, washed with water and dried *in vacuo*. This material was sufficiently pure for complexation studies, without any further purification. Yield 0.51 g, 91 %. Mp 280 °C dec. $^1$H NMR ($\{CD_3\}_2CO$) $\delta$ = 1.99 (m, 4H), 4.32 (br m, 4H), 6.54 (dd, 2H), 7.01 (d, 2H), 7.76 (dd, 2H), 7.81 (d, 2H), 7.89-8.00 (m, 6H), 8.78 (dd, 2H); $^{13}$C NMR ($\{CD_3\}_2CO$) $\delta$ = 28.2, 52.3, 105.1, 108.5, 110.9, 117.8, 127.7, 132.3, 140.5, 142.7, 151.5, 151.9, 152.3; HRMS (ESI): *m*/*z* calcd for $C_{26}H_{24}N_{10}+H^+$: 477.2258 [$M$+H]$^+$; found: 477.2269.

**Synthesis of 2,6-*bis*(3-{2-[pyrazol-1-yl]pyrid-6-yl}pyrazol-1-yl)pyridine ($L^2$).** 2-[Pyrazol-1-yl]-6-[1*H*-pyrazol-3-yl]pyridine (0.50 g, 2.36mmol) was dissolved in *N*,*N*-dimethylformamide (dmf; 25 $cm^3$). Sodium hydride (60 wt% dispersion in mineral oil) (0.10g, 2.60mmol) was added. Once effervescence stopped, the reaction was heated to 50°C. A solution of 2,6-difluoropyridine (0.14 g, 1.18 mmol) in dmf (5 $cm^3$) was added dropwise to the reaction mixture. The reaction was heated to reflux and stirred at reflux for 24 hrs. The reaction was then allowed to cool, before adding water. A white precipitate formed which was isolated by vacuum filtration and then dried.

The product could not be purified because of its insolubility, and was also insufficiently soluble in common solvents for NMR characterization. However, mass spectrometry identified the main product as $L^2$, with the monosubstituted compound 2-(3-{2-[pyrazol-1-yl]pyrid-6-yl}pyrazol-1-yl)-6-fluoropyridine as a significant minor contaminent (Scheme 1, main article). Yield 0.39 g, 66 %. HRMS (ESI): *m*/*z* calcd for $C_{27}H_{19}N_{11}+H^+$: 498.1903 [$M$+H]$^+$; found: 498.1895; calcd for $C_{27}H_{19}N_{11}+Na^+$: 520.1717 [$M$+Na]$^+$; found: 520.1713.

**Synthesis of 1,2-*bis*(3-{2-[pyrazol-1-yl]pyrid-6-yl}pyrazol-1-ylmethyl)benzene ($L^3$)**. 2-[Pyrazol-1-yl]-6-[1*H*-pyrazol-3-yl]pyridine (0.50 g, 2.36 mmol) was dissolved in tetrahydrofuran (25 $cm^3$) under a nitrogen atmosphere, then sodium hydride (60 wt % suspension in mineral oil; 0.10 g, 2.60 mmol) was added. After the effervescence had ceased, the mixture was heated to reflux. 1,2-Di(bromomethyl)benzene (0.31 g, 1.18 mmol) was then added in one portion, and the mixture was stirred at reflux for 4 days. After cooling, the precipitate was collected by filtration to afford $L^3$, as an off-white powder. Yield 0.55 g, 89 %. Mp 175-177 °C; $^1$H NMR ($CDCl_3$) $\delta$ = 5.47 (s, 4H), 6.39 (dd, 2H), 6.88 (d, 2H), 7.17 (m, 2H), 7.28 (d, 4H), 7.67 (d, 2H), 7.74-7.80 (m, 6H), 8.61 (d, 2H); $^{13}$C NMR ($CDCl_3$) $\delta$ = 53.7, 105.4, 107.5, 110.9, 117.5, 127.2, 129.2, 129.9, 131.0, 134.5, 139.3, 141.9, 150.5, 151.1, 151.4; HRMS (ESI): *m*/*z* calcd for $C_{30}H_{24}N_{10}+H^+$: 525.2264 [$M$+H]$^+$; found: 525.2266; calcd for $C_{30}H_{24}N_{10}+Na^+$: 547.2083 [$M$+Na]$^+$; found: 547.2069.

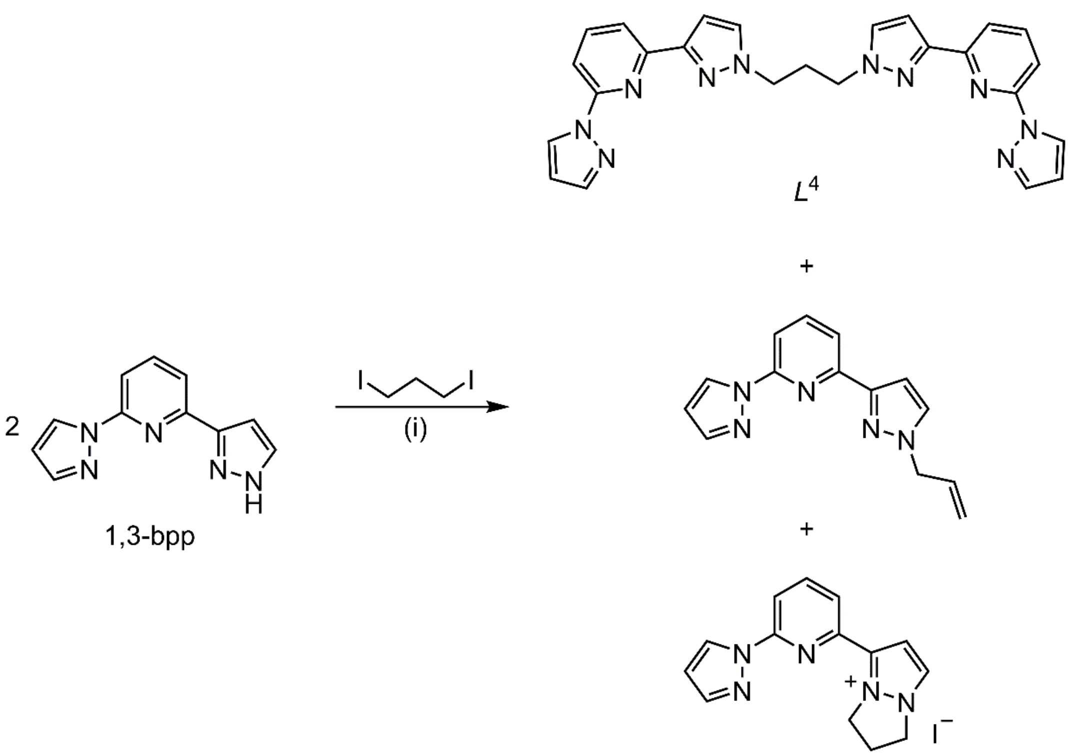


**Scheme S2** Synthesis of 1,3-*bis*(3-{2-[pyrazol-1-yl]pyrid-6-yl}pyrazol-1-yl)propane ($L^4$), and two byproducts. Reagents and conditions: (i) NaH, thf, 298 K then 0.5 eq 1,3-diiodopropane, reflux, 24 hrs.

**Synthesis of 1,3-*bis*(3-{2-[pyrazol-1-yl]pyrid-6-yl}pyrazol-1-yl)propane ($L^4$), 3-(3-{2-[pyrazol-1-yl]pyrid-6-yl}pyrazol-1-yl)prop-1-ene and 5-{2-[pyrazol-1-yl]pyrid-6-yl}-2,3-dihydropyrazolo[1,2-a] pyrazolium iodide.** Method as for $L^1$, using 1,3-diiodopropane (0.35 g, 1.18 mmol). The reaction was heated under reflux for 48 hrs, then cooled and quenched with water (25 $cm^3$) which yielded a white precipitate. This was collected, and proved to be the impurity 3-(3-{2-[pyrazol-1-yl]pyrid-6-yl}pyrazol-1-yl)prop-1-ene. Slow evaporation of the filtrate yielded $L^4$ as a white waxy solid, which was isolated by decanting off the remaining solvent and washed with water.

The yield of $L^4$ varied significantly when this reaction was repeated, between 20-55 %. In some cases, another byproduct was also detected in the aqueous filtrate, 5-{2-[pyrazol-1-yl]pyrid-6-yl}-2,3-dihydro-pyrazolo[1,2-a]pyrazolium iodide. This was isolated by evaporation of the filtrate to dryness after precipitation of $L^4$ was complete, in sufficient purity to confirm its identity.

For $L^4$: Yield 0.30 g, 55 %. $^1$H NMR ({$CD_3$}$_2$CO) $\delta$ = 2.44 (p, 2H), 4.26 (t, 4H), 6.40 (dd, 2H), 6.93 (d, 2H), 7.60 (d, 2H), 7.77-7.88 (m, 8H), 8.66 (dd, 2H); $^{13}$C NMR ({$CD_3$}$_2$SO) $\delta$ = 31.2, 49.5, 105.1, 108.5, 110.6, 117.4, 127.7, 132.6, 140.6, 142.6, 150.6, 151.0, 151.2; HRMS (ESI): *m*/*z* calcd for $C_{25}H_{22}N_{10}$+$Na^+$: 485.1921 [*M*+Na]$^+$; found: 485.1913.

For 3-(3-{2-[pyrazol-1-yl]pyrid-6-yl}pyrazol-1-yl)prop-1-ene: Yield 0.13 g, 22 %. $^1$H NMR ({$CD_3$}$_2$CO) $\delta$ = 4.74 (d, 2H), 5.11 (dd, 1H), 5.13 (m, 1H), 5.99 (ddt, 1H), 6.40 (dd, 1H), 6.92 (d, 1H), 7.58 (d, 1H), 7.62 (, 1H), 7.76-7.88 (m, 3H), 8.65 (dd, 1H); $^{13}$C NMR ({$CD_3$}$_2$CO) $\delta$ = 55.4, 105.5, 108.4, 111.0, 117.7, 118.3, 127.6, 131.9, 134.7, 140.4, 142.6, 151.7, 152.1, 152.3; HRMS (ESI): *m*/*z* calcd for $C_{14}H_{13}N_5$+$Na^+$: 274.1063 [*M*+Na]$^+$; found: 274.1076.

For 5-{2-[pyrazol-1-yl]pyrid-6-yl}-2,3-dihydropyrazolo[1,2-a]pyrazolium iodide: Yield 0.09 g, 10 %. $^1$H NMR ({$CD_3$}$_2$SO) $\delta$ = 1.79 (m, 2H), 3.64 (t, 2H), 4.31 (t, 2H), 6.64 (d, 1H), 7.09 (d, 1H), 7.84-7.91 (m, 2H), 7.96 (d, 1H), 8.01-8.06 (m, 2H), 8.83 (d, 1H). This ionic compound was too insoluble in common organic solvents to give a useful $^{13}$C NMR spectrum; MS (ESI): *m*/*z* calcd for $C_{14}H_{14}N_5^+$: 252.1249 [*M*]$^+$; found: 252.34.

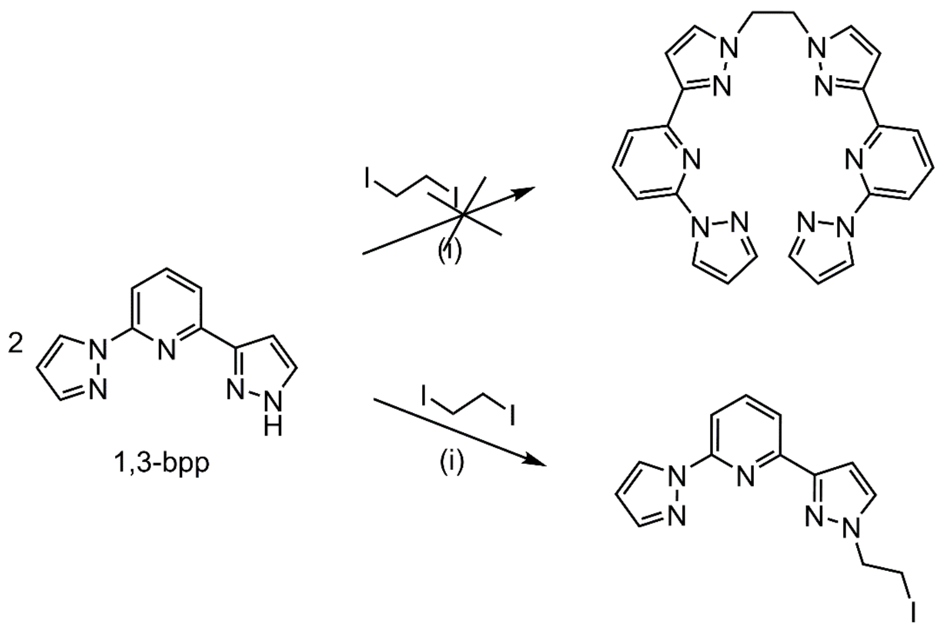


**Scheme S3** Attempted synthesis of 1,2-*bis*(3-{2-[pyrazol-1-yl]pyrid-6-yl}pyrazol-1-yl)ethane. Reagents and conditions: (i) NaH, diglyme, 298 K then 0.5 eq 1,2-diiodoethane, 130 ºC.

**Synthesis of 1-(3-{2-[pyrazol-1-yl]pyrid-6-yl}pyrazol-1-yl)-2-iodoethane.** 2-[Pyrazol-1-yl]-6-[1*H*-pyrazol-3-yl]pyridine (0.50 g, 2.36 mmol) was dissolved in diglyme (25 cm$^3$) under a nitrogen atmosphere, then sodium hydride (60 wt % suspension in mineral oil; 0.10 g, 2.60 mmol) was added. After the effervescence had ceased, the mixture was heated to reflux. 1,2-Diiodoethane (0.33 g, 1.18 mmol) was then added in one portion, and the mixture was heated to 130 °C. TLC and ES-MS analysis showed a reaction had occurred after 24 hr, but that a significant quantity of unreacted 1,3-bpp remained in the solution. The composition of the mixture did not change further upon longer reaction. The cooled reaction was quenched with water (25 cm$^3$), then extracted with dichloromethane (4x 50 cm$^3$). Evaporation of the dried organic fractions to dryness gave an off-white white residue, which was separated by flash silica chromatography using a mixed 3:2:1 ethyl acetate:diethyl ether:hexane eluent. 1-(3-{2-[Pyrazol-1-yl]pyrid-6-yl}pyrazol-1-yl)-2-iodoethane and unreacted 1,3-bpp were obtained as separate fractions from this column. Yield 0.18 g, 42 %. $^1$H NMR ({$CD_3$}$_2$SO) $\delta$ 1.84 (br t, 2H), 4.25 (br t, 2H), 6.60 (dd, 1H), 7.01 (d, 1H), 7.79-7.88 (m, 4H), 7.96 (t, 1H), 8.79 (d, 1H); $^{13}$C NMR ({$CD_3$}$_2$SO) $\delta$ 27.5, 51.5, 104.9, 108.5, 110.5, 117.3, 127.6, 132.4, 140.5, 142.6, 150.4, 150.9, 151.3; HRMS (ESI): *m*/*z* calcd for $C_{13}H_{12}IN_5$+$H^+$: 366.1614 [*M*+H]$^+$; found: 366.1540.

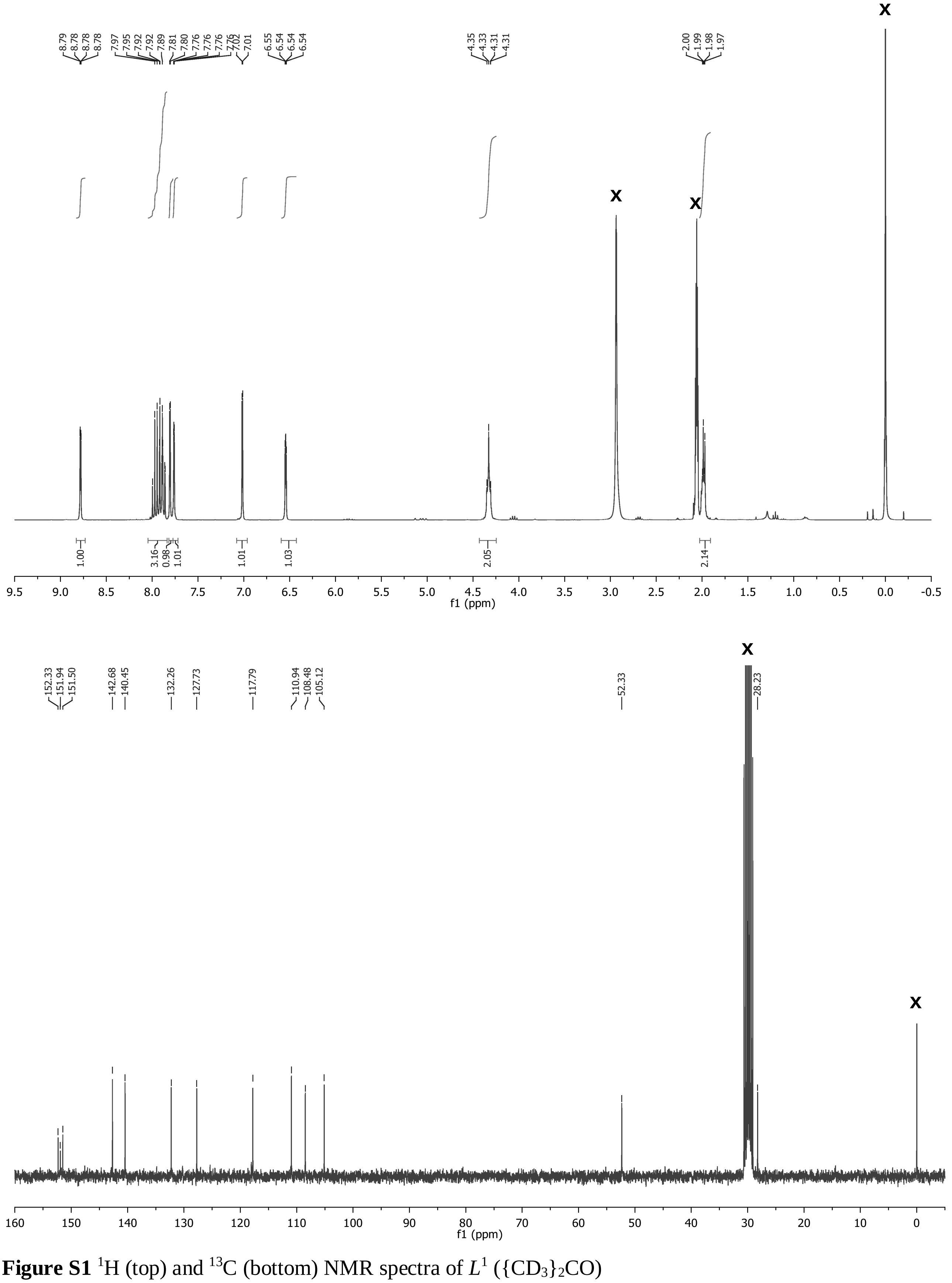

**Figure S1** $^{1}$H (top) and $^{13}$C (bottom) NMR spectra of $L^1$ ($\{CD_3\}_2CO$)

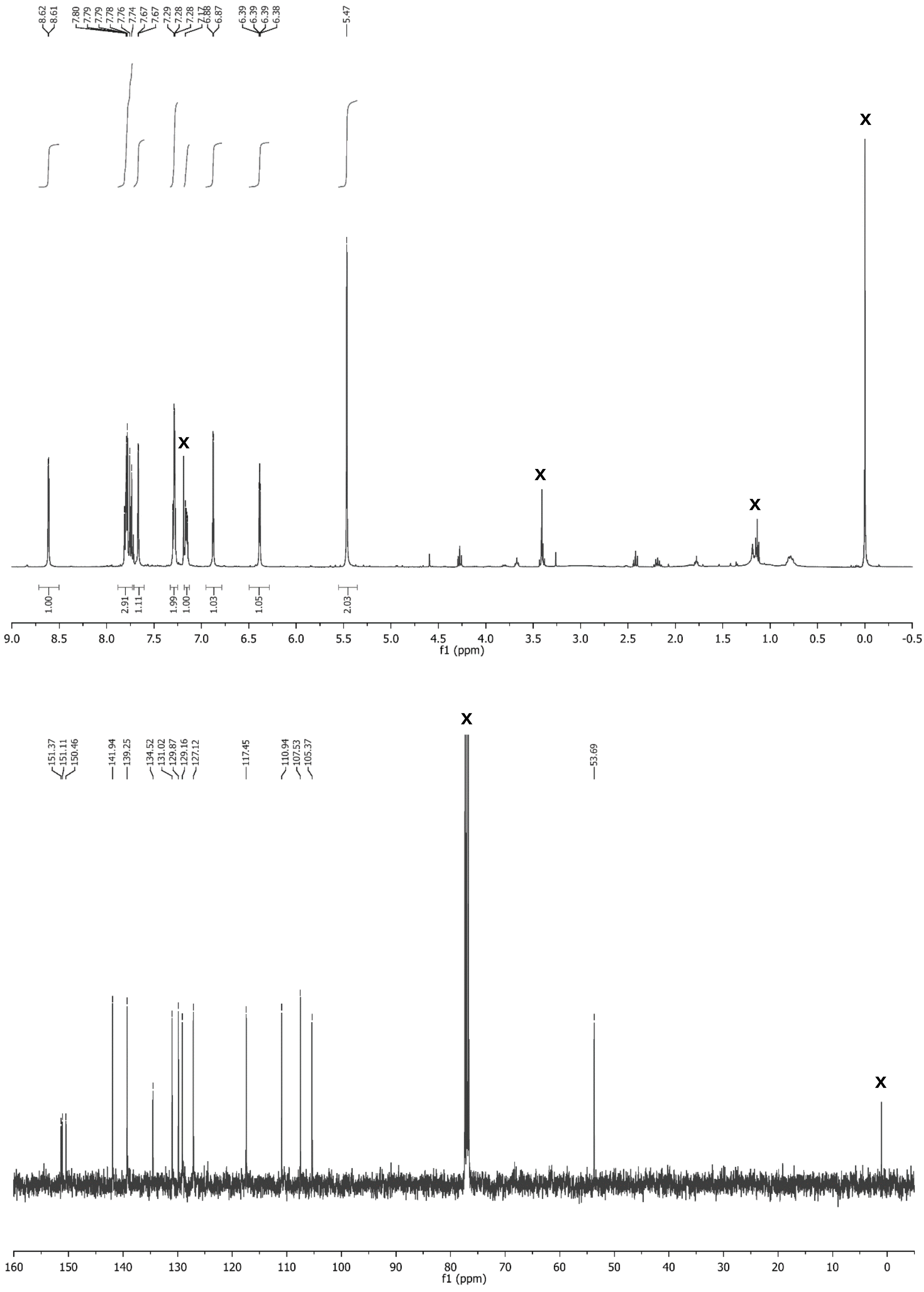


**Figure S2** $^{1}$H (top) and $^{13}$C (bottom) NMR spectra of $L^3$ ($CDCl_3$).

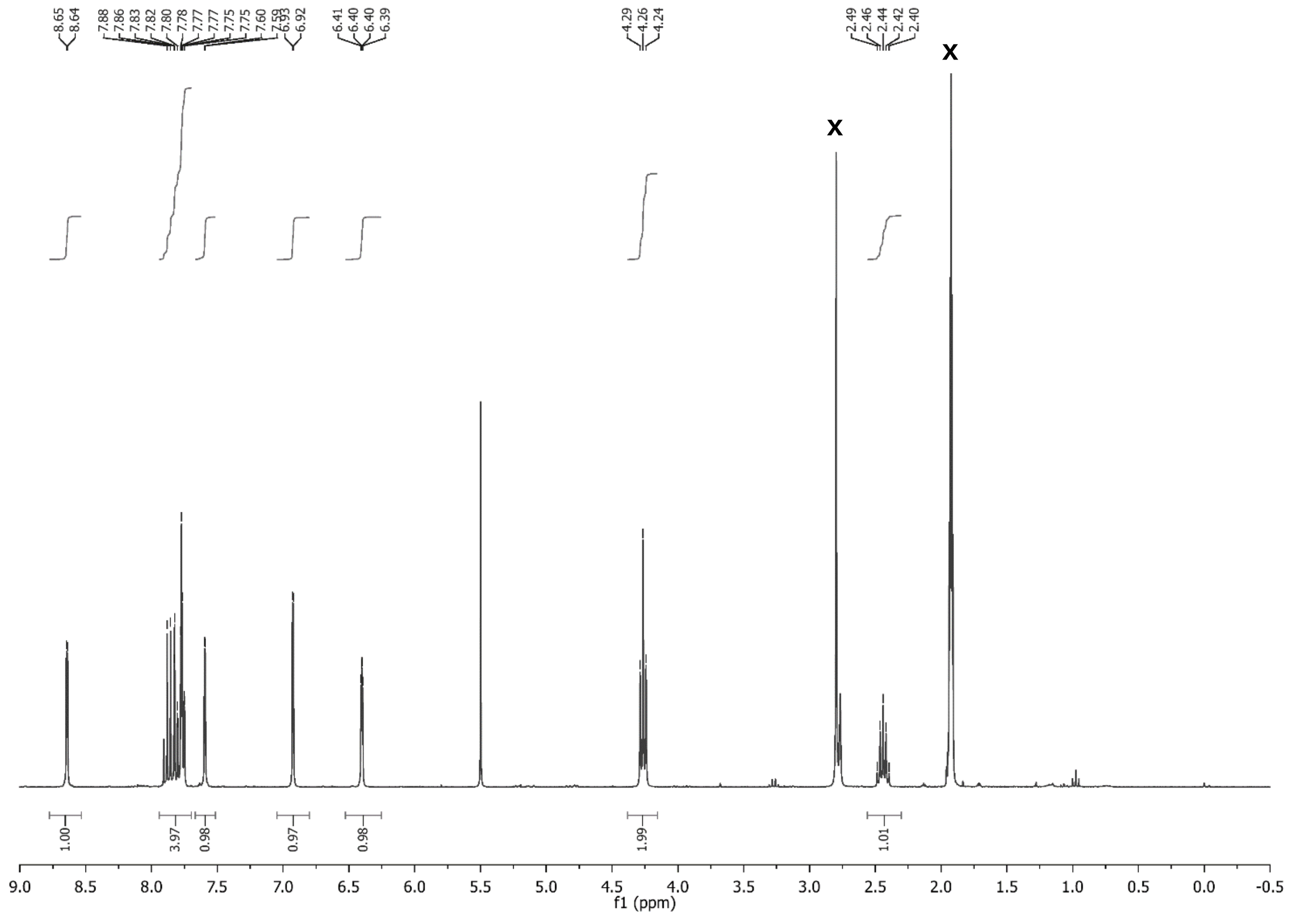

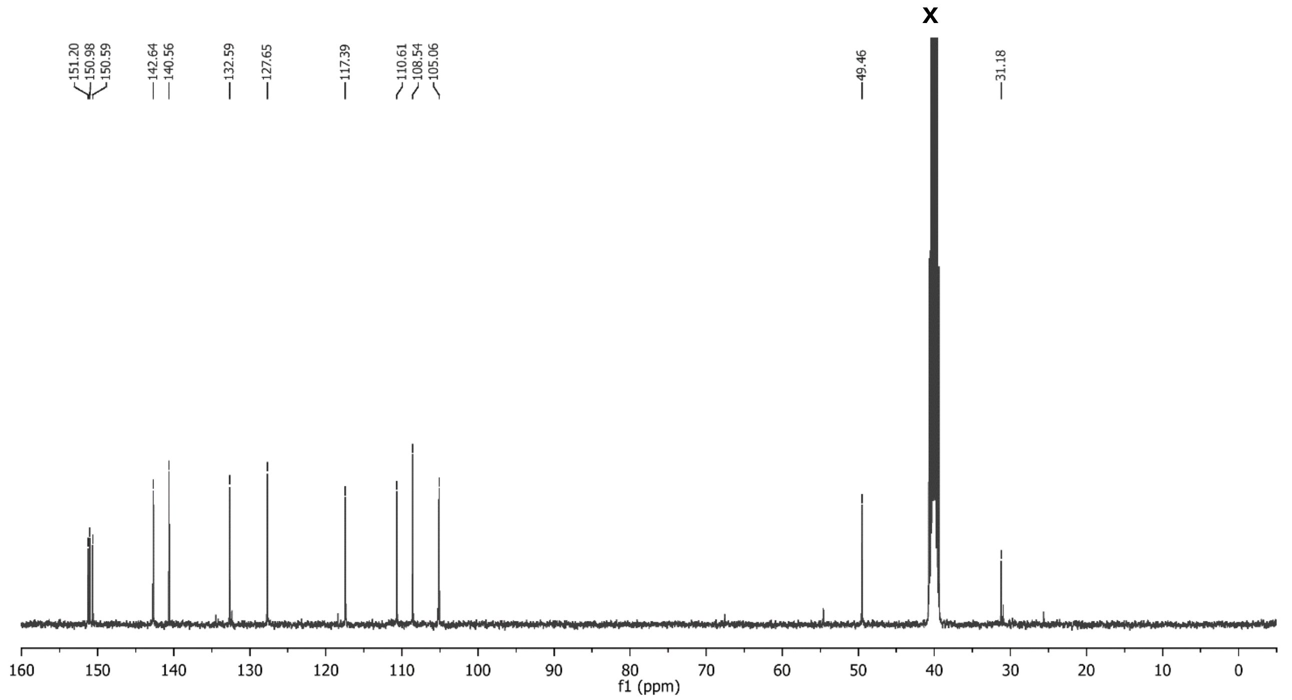

**Figure S3** $^{1}H$ (top, $\{CD_3\}_2CO$) and $^{13}C$ (bottom, $\{CD_3\}_2SO$) NMR spectra of $L^4$ (Scheme 2).

Different solvents were used for these spectra because the $^{13}C$ spectrum in $\{CD_3\}_2CO$ was too weak, while the pentet $^{1}H$ resonance at 2.44 ppm is masked by the residual solvent peak in $\{CD_3\}_2SO$.

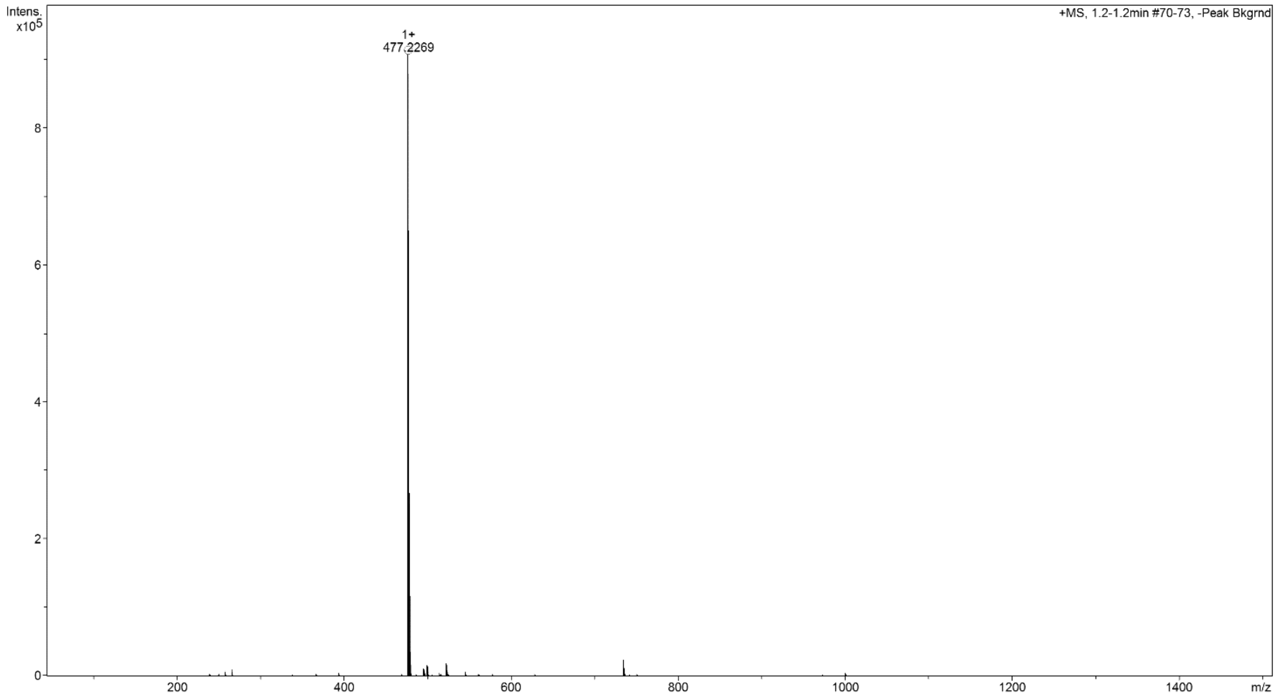


**Figure S4** Electrospray mass spectrum of $L^1$.

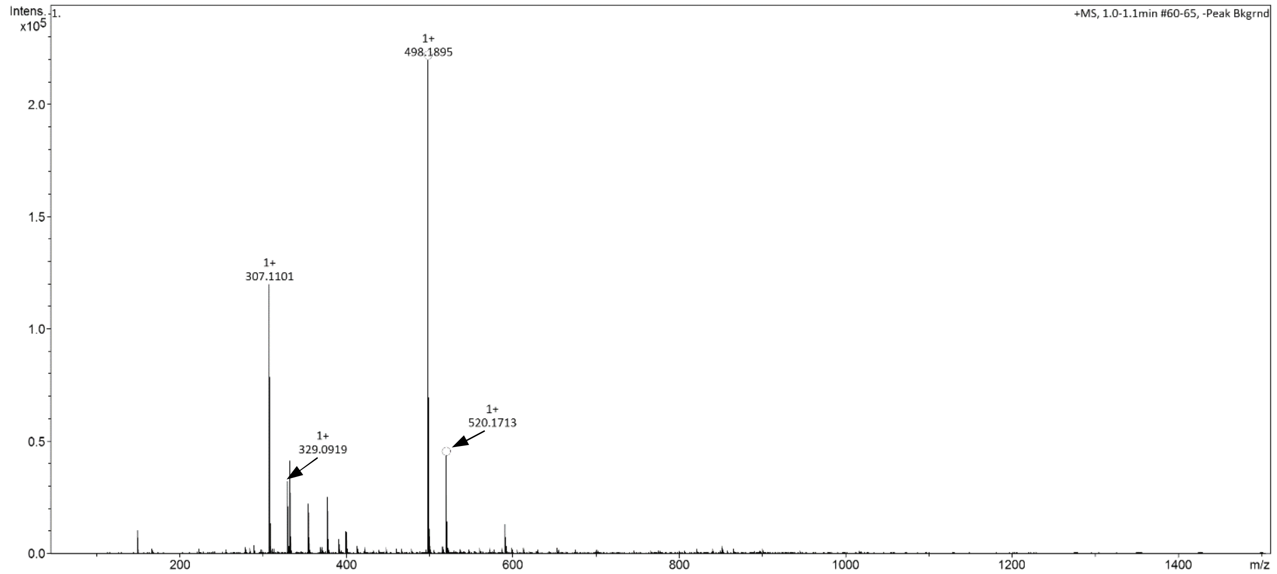


**Figure S5** Electrospray mass spectrum of $L^2$.

The peaks at *m*/*z* 307.1101 (calcd for $[M+H]^+$ *m*/*z* 307.1102) and 329.0919 (calcd for $[M+Na]^+$ *m*/*z* 329.0921) are from the monosubstituted byproduct 2-(3-{2-[pyrazol-1-yl]pyrid-6-yl}pyrazol-1-yl)-6-fluoropyridine (Scheme 1, main article). The other two labelled peaks are the $H^+$ and $Na^+$ adducts of $L^2$.

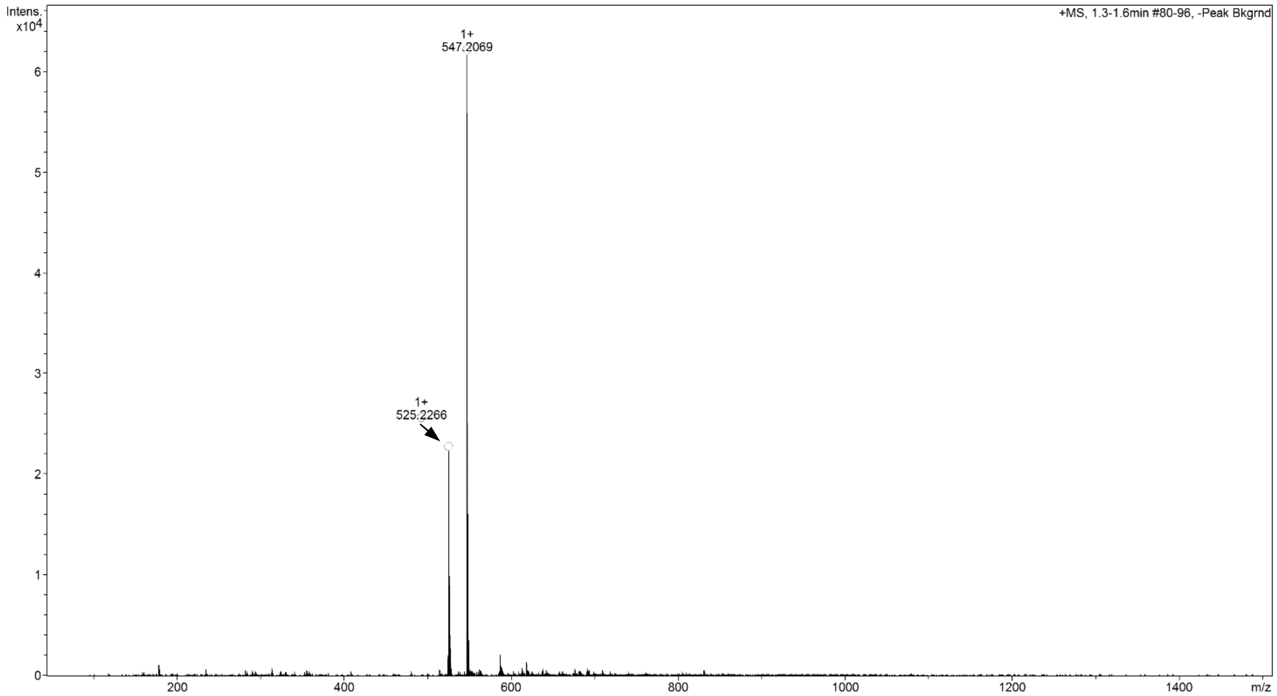


**Figure S6** Electrospray mass spectrum of $L^3$.

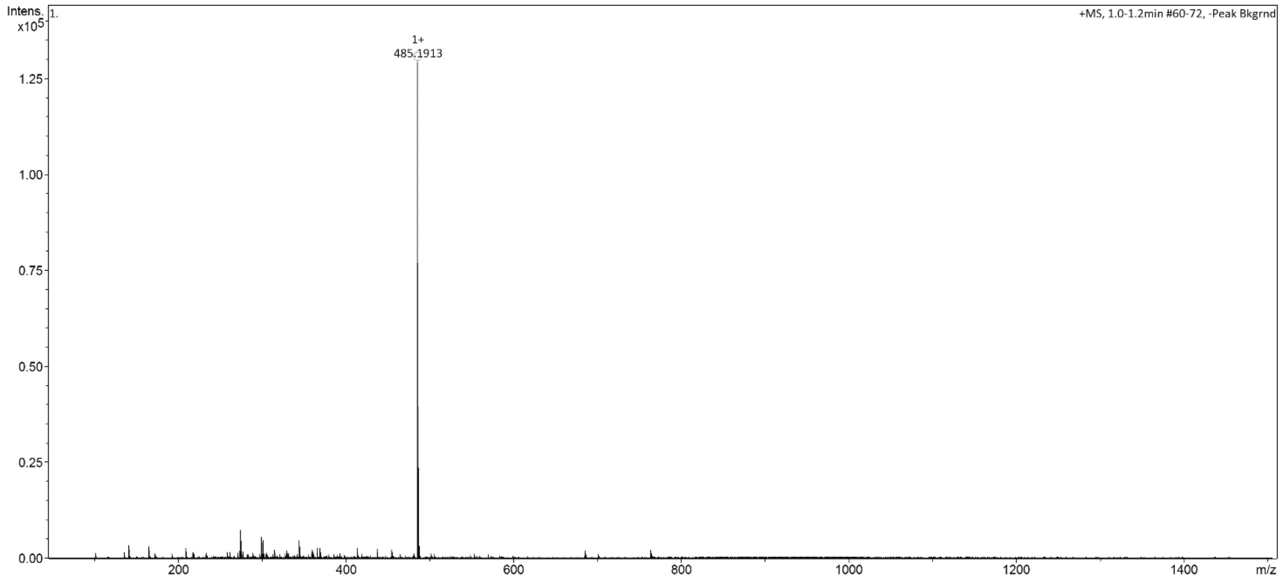


**Figure S7** Electrospray mass spectrum of $L^4$.

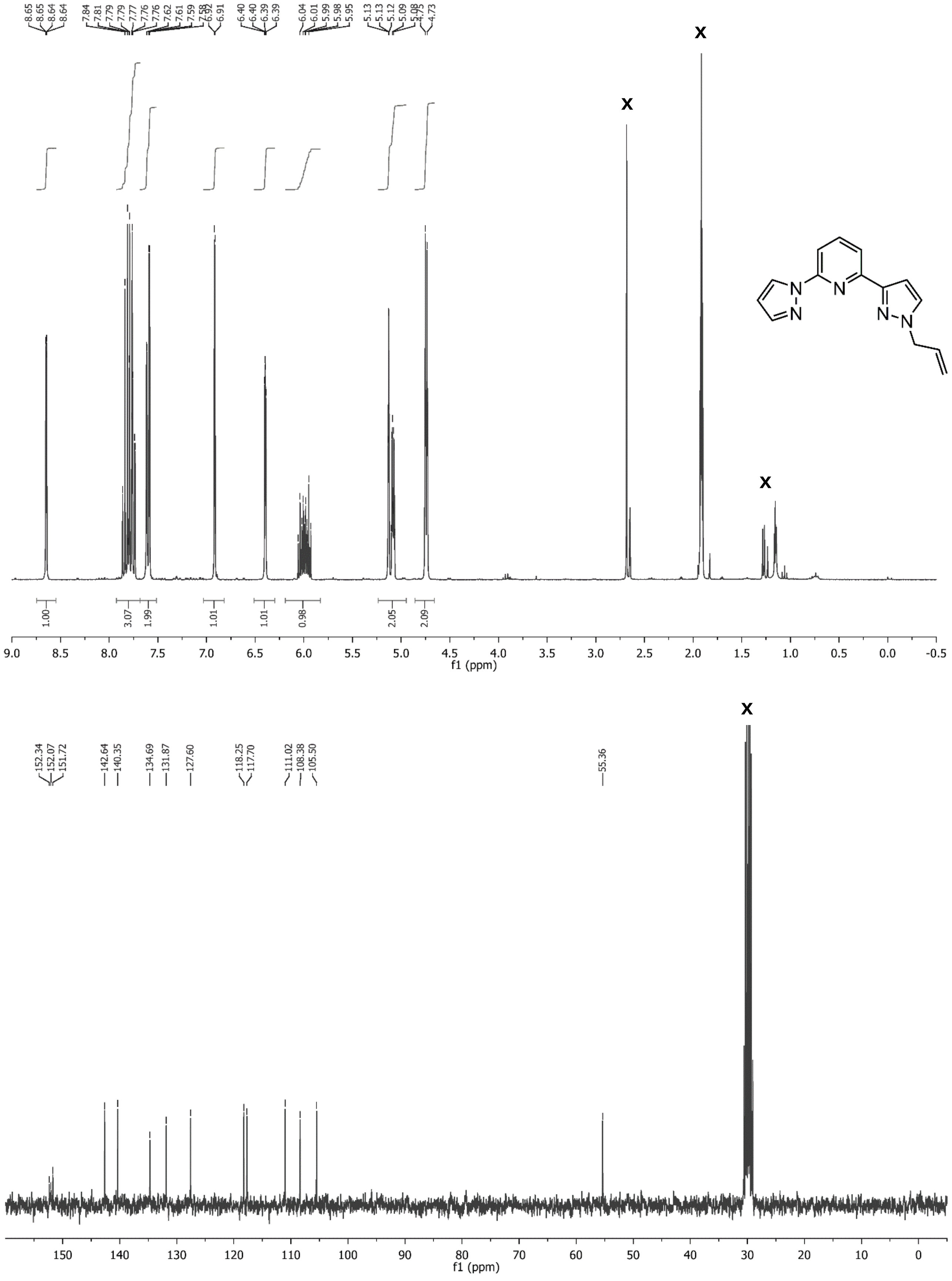


**Figure S8** $^{1}$H (top) and $^{13}$C (bottom) NMR spectra of 3-(3-{2-[pyrazol-1-yl]pyrid-6-yl}pyrazol-1-yl)prop-1-ene, a byproduct in the synthesis of $L^4$ (Scheme S2) ({$CD_3$}$_2$CO).

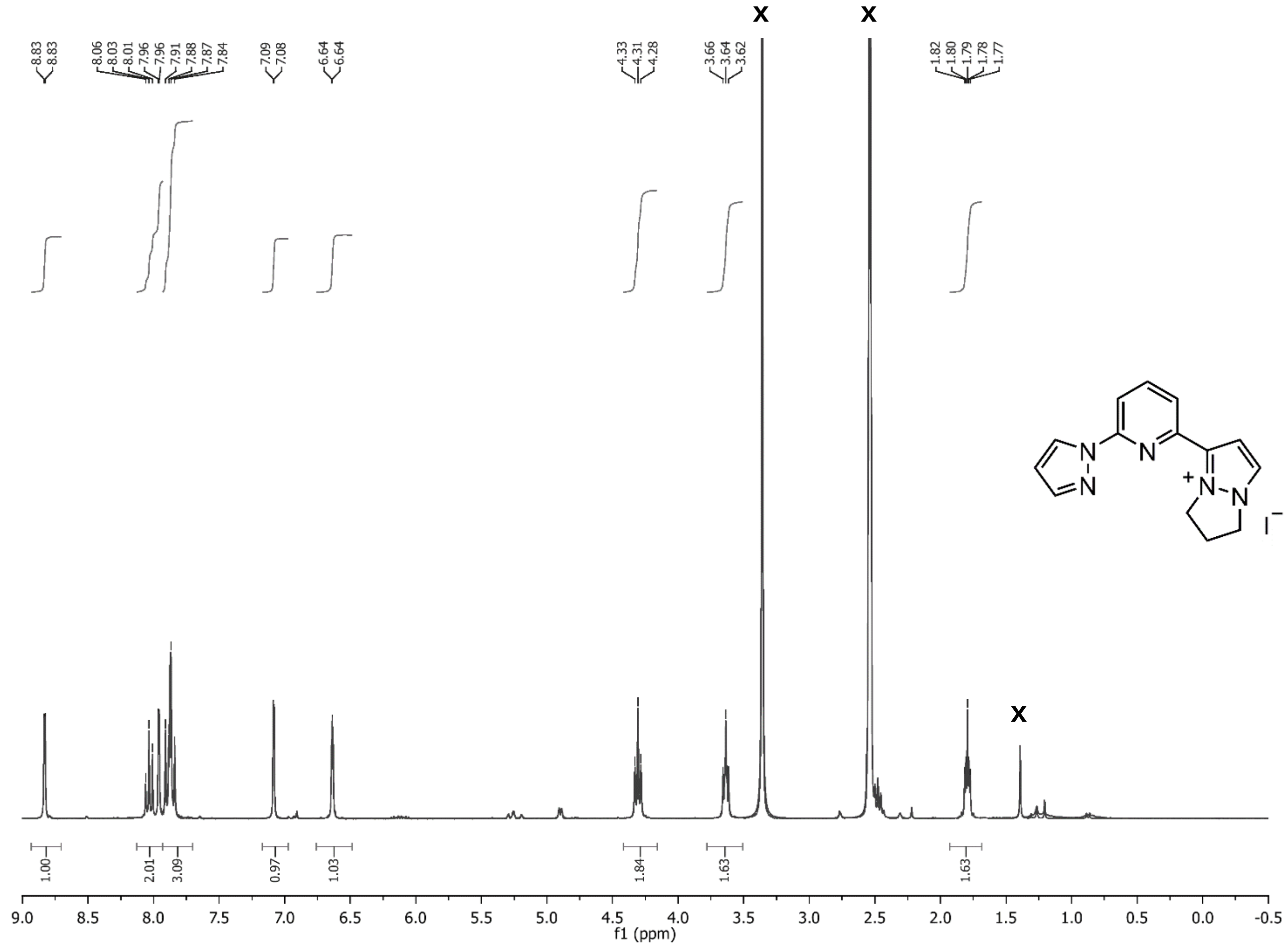


**Figure S9** $^1$H NMR spectrum of 5-{2-[pyrazol-1-yl]pyrid-6-yl}-2,3-dihydropyrazolo[1,2-a]pyrazolium iodide, a byproduct in the synthesis of $L^4$ (Scheme S2) ($\{CD_3\}_2SO$).

This ionic compound was too insoluble to afford a useful $^{13}$C NMR spectrum, but its identity was supported by mass spectrometry.

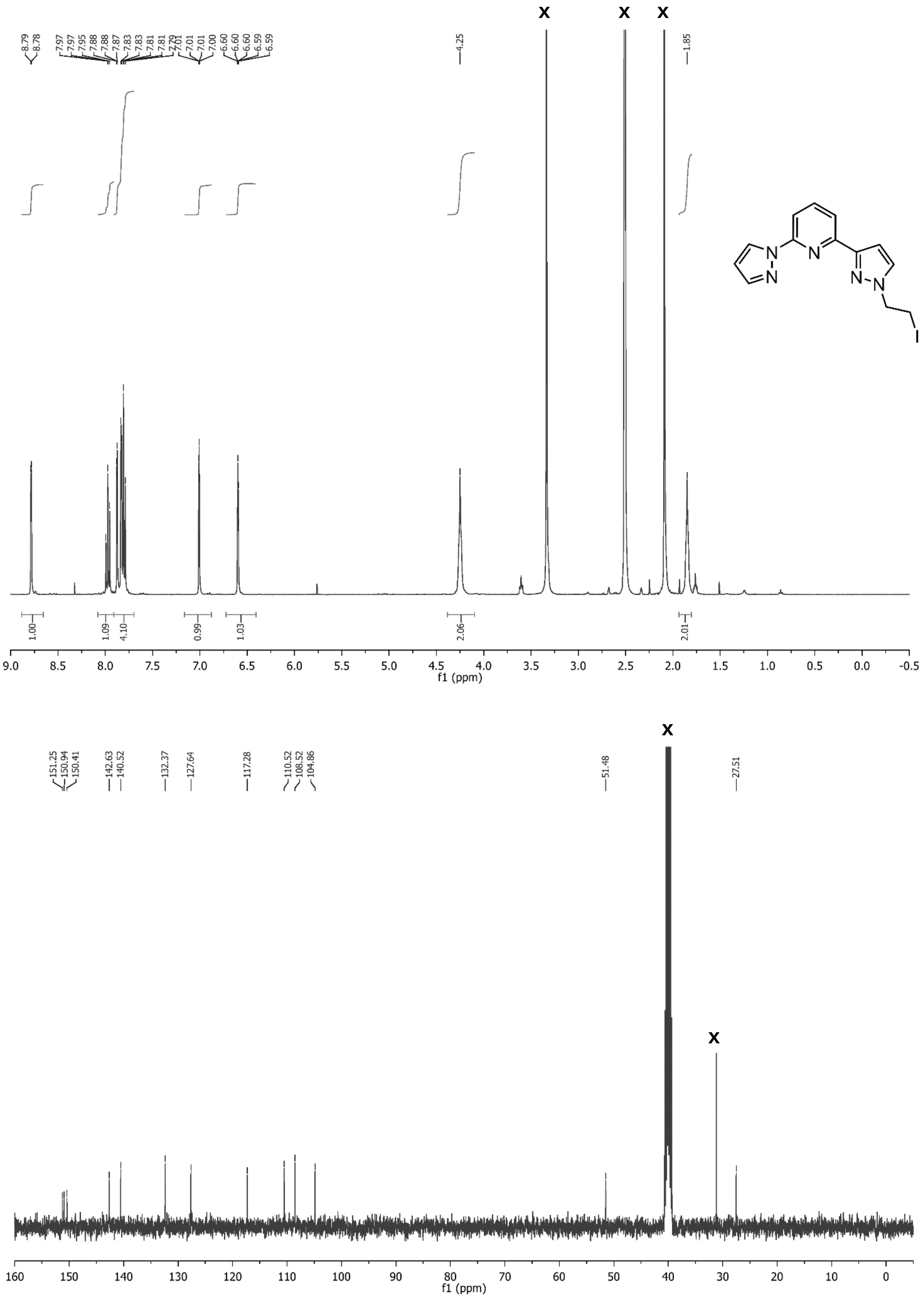


**Figure S10** $^{1}H$ (top) and $^{13}C$ (bottom) NMR spectra of 1-(3-{2-[pyrazol-1-yl]pyrid-6-yl}pyrazol-1-yl)-2-iodoethane ({$CD_3$}$_2$SO).

## Crystal Structure Analyses

Crystals of 1,3-bpp were grown by slow evaporation of an NMR sample of the compound in $CDCl_3$. The solvent-free crystals **2[ClO$_4$]$_4$** were obtained by slow diffusion of diethyl ether vapor into a filtered solution of the complex in acetone. The other solvated crystals were grown similarly, by diethyl ether vapor diffusion in the appropriate solvent. Diffraction data for **1[BF$_4$]$_4$**·*n*$Me_2CO$ were recorded at station I19 of the Diamond synchrotron ($\lambda$ = 0.6889 Å). All other diffraction data were measured with an Agilent Supernova dual-source diffractometer using monochromated Cu-$K_\alpha$ ($\lambda$ = 1.5418 Å) radiation. The diffractometer was fitted with an Oxford Cryostream low-temperature device.

Experimental details of the structure determinations in this study are given in Table S1. All the structures were solved by direct methods *(SHELXS*[2]), and developed by full least-squares refinement on $F^2$ (*SHELXL-2018*[2]). Crystallographic figures were prepared using *XSEED*,[3] while calculation of structural indices and preparation of publication materials was performed with *Olex2*.[4]

Unless otherwise stated, the following refinement protocols were used. Disordered anions were treated with refined B–F/Cl–O and F···F/O···O distance restraints; and disordered lattice solvent molecules were modelled using fixed bond length and angle restraints. All crystallographically ordered non-H atoms were refined anisotropically, and H atoms were placed in calculated positions and refined using a riding model.

**Structure refinement of $\alpha$-1,3-bpp.** The asymmetric unit contains four unique molecules of the compound. No disorder is present in the model, and no restraints were applied to the refinement. All non-H atoms were refined anisotropically, and H atoms were located in the Fourier map and refined, with $U_{iso}$ constrained to 1.2x$U_{eq}$ of the corresponding C or N atom.

**Structure refinement of $\beta$-1,3-bpp.** This crystal had a needle morphology, and diffracted weakly along its short axes. A solution of the dataset showed one unique molecule in the asymmetric unit, but the precision of the refinement is low. The final refinement residuals were $R_1[I > 2\sigma(I)]$ = 0.105, $wR_2$[all data] = 0.294.

This crystal has not been deposited with the CSD.

**Structure refinements of 1[BF$_4$]$_4$·*n*Me$_2$CO (*n* ≈ 2.5).** Two datasets were collected from the same crystal, at 100 K and 250 K (in that order), using synchrotron radiation.

At 100 K, three of the four unique $BF_4^-$ anions are disordered: one anion was disordered over two sites with 0.67:0.33 occupancy; one over two sites with 0.60:0.40 occupancy; and one over three equally occupied orientations. Two of the acetone solvent sites were also disordered, and refined over two sites with occupancies summing to 0.75.

The same residues were disordered at 250 K. One $BF_4^-$ ion refined over two sites with 0.67:0.33 occupancy, as above; the second over three sites with 0.50:0.35:0.15, with the latter two sharing a common half-occupied B atom; and the third one over three sites with 0.55:0.30:0.15 occupancies, all of which share a common wholly occupied B atom. The two disordered acetone molecules were treated with three sites, whose occupancies again sum to 0.75.

**Structure refinement of 1[BF$_4$]$_4$·2MeCN·Et$_2$O.** This refinement includes a minor merohedral twin domain, to account for a *CheckCif* B alert. The ratio of the twin domains in the final refinement is 0.97:0.03.

The asymmetric unit contains one formula unit of the compound. Two of the four $BF_4^-$ ions are disordered, one over two equally occuupied orientations and the other over three sites with occupancies of 0.6, 0.25 and 0.15. While the diethyl ether was modelled as ordered, both MeCN molecules were also disordered over two half-occupied positions. All fully occupied non-H atoms were refined anisotropically, and H atoms were placed in calculated positions and refined using a riding model. The highest residual Fourier peak of +1.2 $e$Å$^{-3}$ lies within one of the disordered solvent sites.

**Structure refinement of 1[BF$_4$]$_4$·*m*MeNO$_2$ (*m* ≈ 4.5).** Two morphologies were identified in crystallization vials of **1[BF$_4$]$_4$** from nitromethane/diethyl ether. This was the major component of the samples, which forms clusters of orange needles. A fragment was broken off one such cluster for characterization; the crystal used for the measurement was a racemic twin.

The asymmetric unit contains one dinuclear complex molecule; four disordered $BF_4^-$ ions; and five solvent sites, three of which were also disordered. All these disordered residues were refined over two orientations. In addition to the distance restraints listed above, the isotropic $U_{iso}$ displacement ellipsoids for each orientation of two of the anions were also constrained to be similar (*SHELXL SIMU* instruction). The disorder orientations for one solvent site had reduced occupancies, which sum to 0.5. That explains the substoichiometric solvent content in the crystal.

One pyrazolyl ring bound to each iron atom is also disordered over two sites, which were refined without restraints. The Fe–N distances to these groups imply the disorder corresponds to the high-spin and low-spin orientations of metal ion with a mixed spin state population.

All fully occupied non-H atoms were refined anisotropically, except for B(80) (a wholly occupied B atom at the center of a disordered anion) which became non-positive definite and was left isotropic. H atoms were placed in calculated positions and refined using a riding model. The highest residual Fourier peak of +1.2 $e$Å$^{-3}$ lies within one of the disordered anions.

**Structure refinement of 1[BF$_4$]$_4$·2MeNO$_2$.** This brown prismatic solvatomorph was the minor component in samples of **1[BF$_4$]$_4$** recrystallized from nitromethane/diethyl ether.

The asymmetric unit of this crystal contains half a complex cation, with Fe(1) and Fe(2) lying on the crystallographic $C_2$ axis; and, two anions and one solvent molecule on general crystallographic sites. No disorder is included in the model, and no restraints were applied to the refinement. The highest residual Fourier peak of +1.2 $e$Å$^{-3}$ lies within the ligand 1,4-butylenyl linker, and may indicate a minor degree of disorder in that residue.

**Structure refinement of 2[BF$_4$]$_4$·MeNO$_2$·Et$_2$O**. The asymmetric unit contains half a $[Fe_2(\mu\text{-}L^2)_2]^{4+}$ dimer cation spanning a crystallographic $C_2$ axis; two unique $BF_4^-$ ions; and a disordered region of solvent which was modelled as half a molecule of nitromethane, and two quarter molecules of diethyl ether. All the anion and solvent sites are disordered over two or three orientations. Large, elongated displacement ellipsoids on some C atoms imply the disorder might also extend to parts of the ligand backbone (Figure S28).

The crystal has a high mosaicity, reflecting the extensive disorder in the model. Since the two **2[ClO$_4$]$_4$** structure refinements are of higher quality, a better dataset of this crystal was not pursued. The final refinement residuals were $R_1[I > 2\sigma(I)]$ = 0.112, $wR_2$[all data] = 0.311, goodness of fit = 1.061.

This structure has not been deposited with the CCDC.

**Structure refinement of 2[ClO$_4$]$_4$.** Two of the four perchlorate ions in the asymmetric unit were clearly disordered and were modelled over two orientations, one with equal occupancies and the other with a refined occupancy ratio of 0.73:0.27. All fully occupied non-H atoms, plus partial Cl atoms with occupancy ≥0.5, were refined anisotropically.

**Structure refinement of 2[ClO$_4$]$_4$·3MeNO$_2$·0.75H$_2$O**. One perchlorate ion is disordered over two orientations with an occupancy ratio of 0.75:0.25. The water site (which is within hydrogen bonding distance of the disordered anion) is only partially occupied on the basis of its displacement parameter, and was given an occupancy of 0.75 to correlate with the anion disorder. The partial water H atoms were not located in the Fourier map and are not included in the model, but are accounted for in the molecular weight and density calculations.

CCDC 2169630-2169637 contain the supplementary crystallographic data for this paper (Table S1). These data can be obtained free of charge from The Cambridge Crystallographic Data Centre via **www.ccdc.cam.ac.uk/data_request/cif**.

**Table S1** Experimental data for the crystal structures in this work.

| | $\alpha$-1,3-bpp | $\beta$-1,3-bpp[c] | **1[BF$_4$]$_4$**$\cdot n$Me$_2$CO ($n \approx 2.5$) | | **1[BF$_4$]$_4$**$\cdot$2MeCN$\cdot$Et$_2$O | **1[BF$_4$]$_4$**$\cdot m$MeNO$_2$ ($m \approx 4.5$) |
|---|---|---|---|---|---|---|
| molecular formula | $C_{11}H_9N_5$ | $C_{11}H_9N_5$ | $C_{59.50}H_{63}B_4F_{16}Fe_2N_{20}O_{2.50}$ | | $C_{60}H_{64}B_4F_{16}Fe_2N_{22}O$ | $C_{56.50}H_{61.50}B_4F_{16}Fe_2N_{24.50}O_9$ |
| $M_r$ | 211.23 | 211.23 | 1557.24 | | 1568.27 | 1686.74 |
| crystal class | triclinic | orthorhombic | monoclinic | | orthorhombic | orthorhombic |
| space group | $P$-1 | $Pna2_1$ | $P2_1/n$ | | $Pna2_1$ | $P2_12_12_1$ |
| $a$ [Å] | 11.2826(6) | 9.604(3) | 16.5464(1) | 16.4234(4) | 38.4573(9) | 12.2092(2) |
| $b$ [Å] | 11.3276(5) | 23.193(4) | 18.8642(1) | 18.6230(4) | 12.2120(3) | 15.1695(4) |
| $c$ [Å] | 15.6422(9) | 4.4500(14) | 23.1657(1) | 22.8727(4) | 15.1061(4) | 39.0707(8) |
| $\alpha$ [°] | 83.201(4) | 90 | 90 | 90 | 90 | 90 |
| $\beta$ [°] | 89.292(5) | 90 | 94.325(1) | 93.875(2) | 90 | 90 |
| $\gamma$ [°] | 89.544(4) | 90 | 90 | 90 | 90 | 90 |
| $V$ [Å$^3$] | 1984.90(18) | 991.2(5) | 7210.23(7) | 6979.7(3) | 7094.4(3) | 7236.2(3) |
| $Z$ | 8 | 4 | 4 | 4 | 4 | 4 |
| $T$ [K] | 120(2) | 120(2) | 250(2) | 100(2) | 125(2) | 125(2) |
| $\mu$ [mm$^{-1}$] | 0.750[d] | 0.751[d] | 0.463[e] | 0.479[e] | 4.154[d] | 4.214[d] |
| $D_c$ [g cm$^{-3}$] | 1.414 | 1.415 | 1.435 | 1.482 | 1.468 | 1.548 |
| measured reflections | 17843 | – | 77069 | 71953 | 18503 | 50571 |
| independent reflections | 7504 | – | 15951 | 15362 | 9116 | 14278 |
| $R_{int}$ | 0.035 | – | 0.038 | 0.065 | 0.046 | 0.063 |
| parameters | 685 | – | 1000 | 955 | 958 | 986 |
| restraints | 0 | – | 116 | 94 | 63 | 140 |
| $R_1$ [$F_0 > 4\sigma(F_0)$][a] | 0.050 | – | 0.067 | 0.087 | 0.072 | 0.102 |
| $wR_2$, all data[b] | 0.128 | – | 0.228 | 0.296 | 0.212 | 0.285 |
| goodness of fit | 1.093 | – | 1.038 | 1.126 | 1.068 | 1.049 |
| $\Delta\rho_{min/max}$ [$e$Å$^{-3}$] | –0.28/0.21 | – | –0.60/0.89 | –1.04/0.99 | –0.40/1.07 | –0.68/1.24 |
| Flack parameter | – | – | – | – | 0.031(10)[f] | 0.307(11)[f] |
| CCDC | 2169630 | – | 2169631 | 2169632 | 2169633 | 2169634 |

[a]$R = \Sigma[|F_o| - |F_c|] / \Sigma|F_o|$ [b]$wR = [\Sigma w(F_o^2 - F_c^2) / \Sigma wF_o^4]^{1/2}$ [c]While a preliminary solution of this dataset was achieved (Figure S13), the needle crystal diffracted too weakly for a precise anisotropic refinement. This structure has not been deposited with the CSD. [d]Collected with Cu-$K_\alpha$ radiation. [e]Collected with synchrotron radiation. [f]This crystal was refined as a merohedral twin. [g]While a preliminary solution of this dataset was achieved (Figure S28), the dataset has high mosaicity which may reflect the extensive disorder in the model. This structure has not been deposited with the CSD.

**Table S1** continued

| | **1[BF$_4$]$_4$**·2MeNO$_2$ | **2[BF$_4$]$_4$**·MeNO$_2$·Et$_2$O[g] | **2[ClO$_4$]$_4$** | **2[ClO$_4$]$_4$**·3MeNO$_2$-·0.75H$_2$O |
|---|---|---|---|---|
| molecular formula | $C_{54}H_{54}B_4F_{16}Fe_2N_{22}O_4$ | $C_{59}H_{51}B_4F_{16}Fe_2N_{23}O_3$ | $C_{54}H_{38}Cl_4Fe_2N_{22}O_{16}$ | $C_{57}H_{48.50}Cl_4Fe_2N_{25}O_{22.75}$ |
| $M_r$ | 1534.13 | 1589.16 | 1504.56 | 1701.21 |
| crystal class | monoclinic | monoclinic | monoclinic | monoclinic |
| space group | *C*2 | *C*2/*c* | *P*2$_1$/*c* | *P*2$_1$/*c* |
| *a* [Å] | 20.6997(3) | 23.2784(6) | 23.0595(4) | 16.8762(2) |
| *b* [Å] | 15.7338(2) | 12.9520(4) | 12.1632(2) | 28.6155(3) |
| *c* [Å] | 11.1863(2) | 22.2358(4) | 23.6472(4) | 13.9828(1) |
| $\alpha$ [°] | 90 | 90 | 90 | 90 |
| $\beta$ [°] | 118.931(1) | 98.795(2) | 118.872(2) | 92.407(1) |
| $\gamma$ [°] | 90 | 90 | 90 | 90 |
| *V* [Å$^3$] | 3188.55(9) | 6625.3(3) | 5808.1(2) | 6746.63(12) |
| *Z* | 2 | 4 | 4 | 4 |
| *T* [K] | 125(2) | 120(2) | 120(2) | 120(2) |
| $\mu$ [mm$^{-1}$] | 4.644[d] | 4.490[d] | 6.509[d] | 5.772[d] |
| $D_c$ [g cm$^{-3}$] | 1.598 | 1.593 | 1.721 | 1.675 |
| measured reflections | 11759 | – | 24574 | 33698 |
| independent reflections | 6235 | – | 11335 | 12888 |
| $R_{int}$ | 0.023 | – | 0.024 | 0.033 |
| parameters | 462 | – | 889 | 994 |
| restraints | 1 | – | 40 | 20 |
| $R_1$ [$F_0 > 4\sigma(F_0)$][a] | 0.042 | – | 0.047 | 0.052 |
| $wR_2$, all data[b] | 0.111 | – | 0.132 | 0.147 |
| goodness of fit | 1.038 | – | 1.045 | 1.041 |
| $\Delta\rho_{min/max}$ [$e$Å$^{-3}$] | –0.40/1.18 | – | –0.61/0.84 | –1.51/1.19 |
| Flack parameter | –0.004(2) | – | – | – |
| CCDC | 2169635 | – | 2169636 | 2169637 |

[a]$R = \Sigma[|F_o| - |F_c|] / \Sigma|F_o|$ [b]$wR = [\Sigma w(F_o^2 - F_c^2) / \Sigma wF_o^4]^{1/2}$ [c]While a preliminary solution of this dataset was achieved (Figure S13), the needle crystal diffracted too weakly for a precise anisotropic refinement. This structure has not been deposited with the CSD. [d]Collected with Cu-$K_\alpha$ radiation. [e]Collected with synchrotron radiation. [f]This crystal was refined as a merohedral twin. [g]While a preliminary solution of this dataset was achieved (Figure S28), the dataset has high mosaicity which may reflect the extensive disorder in the model. This structure has not been deposited with the CSD.

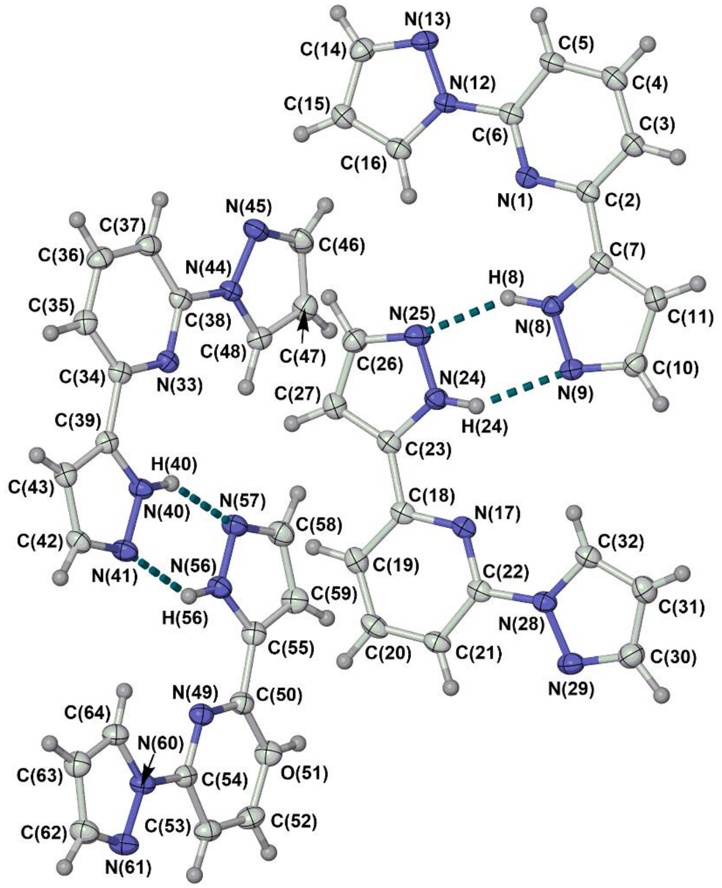


**Figure S11** The four unique molecules in the asymmetric unit of $\alpha$-1,3-bpp. Displacement ellipsoids are at the 50 % probability level, except for H atoms which have arbitrary radii. Color code: C, white; H, pale gray; N, blue.

This hydrogen-bonded cyclic dimer motif is commonly found in crystals of 1*H*-pyrazole derivatives.[5]

**Table S2** Hydrogen bond parameters for $\alpha$-1,3-bpp [Å, °] (Figure S11).

| | D–H | H···A | D···A | D–H···A |
|---|---|---|---|---|
| N(8)–H(8)···N(25) | 0.94(2) | 2.06(2) | 2.902(2) | 148(2) |
| N(24)–H(24)···N(9) | 0.87(3) | 2.12(3) | 2.903(2) | 149(2) |
| N(40)–H(40)···N(57) | 0.93(2) | 2.07(2) | 2.912(2) | 149(2) |
| N(56)–H(56)···N(41) | 0.88(3) | 2.18(3) | 2.928(2) | 143(2) |

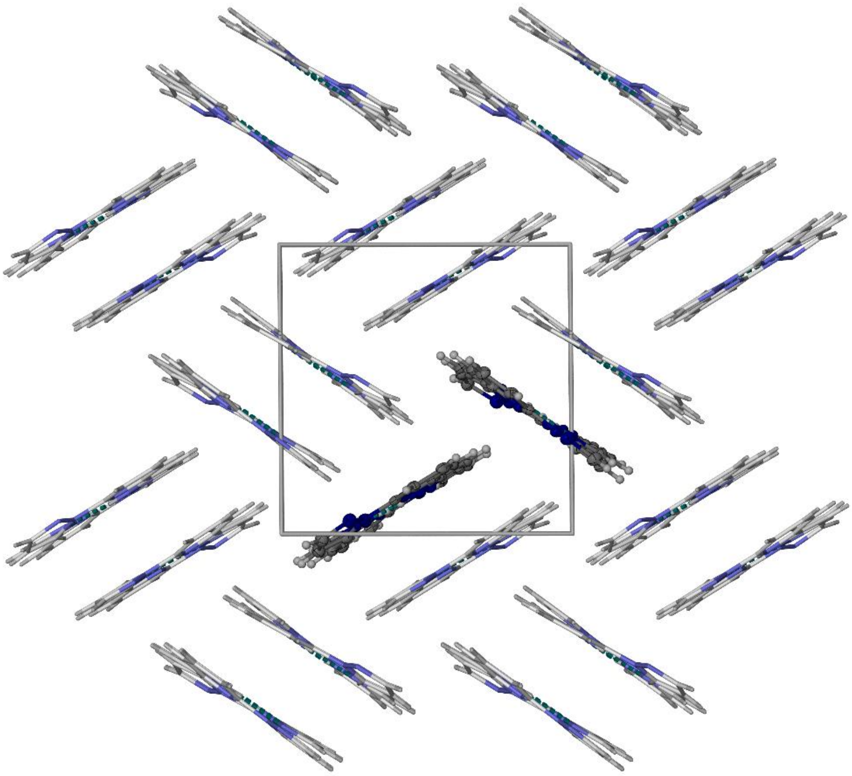

**Figure S12** Packing diagram of $\alpha$-1,3-bpp, viewed perpendicular to the [001] crystal vector. Molecules in the asymmetric unit are highlighted with dark coloration, and the dimensions of the unit cell are shown. Color code: C, white or dark gray; H, pale gray; N, pale or dark blue.

Each hydrogen-bonded pair of molecules (Figure S11) associates into a centrosymmetric face-to-face $\pi\cdots\pi$ dimer, which in turn propagate by translation along *c* (perpendicular to the Figure). The pairs-of-dimers within each bilayer are coplanar by symmetry, and separated by 3.280(2) Å [N(1)-C(32) and N($1^{i}$)-C($32^{i}$)] and 3.304(2) Å [N(33)-C(64) and N($33^{ii}$)-C($64^{ii}$)]. Symmetry codes: (i) 1–*x*, –*y*, 1–*z*; (ii) –*x*, 1–*y*, 1–*z*.

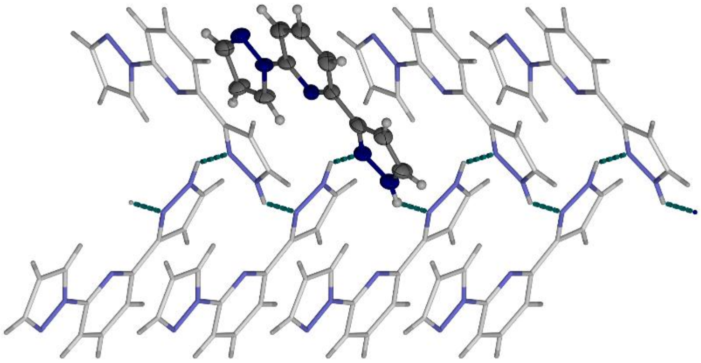

**Figure S13** Partial packing diagram of $\beta$-1,3-bpp, from the preliminary structure solution of that compound. Details as for Figure S12.

Molecules in this polymorph associate chains running parallel to [001], through N–H···N hydrogen bonds and face-to-face $\pi\cdots\pi$ interactions.

## Definitions of the Structural Parameters Tabulated for the Complexes

$V_{Oh}$ is the volume (in Å$^3$) of the $FeN_6$ coordination octahedron in the complex,[6] which is typically <10 Å$^3$ in low-spin $[Fe(bpp)_2]^{2+}$ (bpp = 2,6-di{pyrazol-1-yl}pyridine) derivatives and ≥11.5 Å$^3$ in their high-spin form.[7]

$\Sigma$ and $\Theta$ are defined as follows:

$$\Sigma = \sum_{i=1}^{12} |90 - \beta_i| \qquad \Theta = \sum_{j=1}^{24} |60 - \gamma_j|$$

where $\beta_i$ are the twelve *cis*-N–Fe–N angles about the iron atom and $\gamma_i$ are the 24 unique N–Fe–N angles measured on the projection of two triangular faces of the octahedron along their common pseudo-threefold axis (Scheme S1). $\Sigma$ is a general measure of the deviation of a metal ion from an ideal octahedral geometry, while $\Theta$ more specifically indicates its distortion towards a trigonal prismatic structure. A perfectly octahedral complex gives $\Sigma = \Theta = 0$.[6,8]

Because the high-spin state of a complex has a much more plastic structure than the low-spin, this is reflected in $\Sigma$ and $\Theta$ which are usually much larger in the high-spin state. The absolute values of these parameters depend on the metal/ligand combination in the compound under investigation, however.

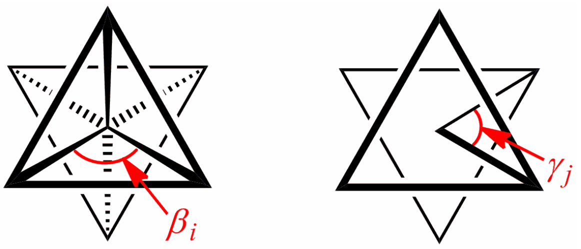


**Scheme S4** Angles used in the definitions of the coordination distortion parameters $\Sigma$ and $\Theta$.

The parameters in Scheme S2 define the magnitude of an angular Jahn-Teller distortion, that is often observed in high-spin $[Fe(bpp)_2]^{2+}$ derivatives ($\theta \leq 90°$, $\phi \leq 180°$).[9] They are also a useful indicator of the molecular geometry, in defining the disposition of the two ligands around the metal ion. Spin-crossover can be inhibited if $\theta$ and $\phi$ deviate significantly from their ideal values, because the associated rearrangement to a more regular low-spin coordination geometry ($\theta \approx 90°$, $\phi \approx 180°$) cannot be accommodated by a rigid solid lattice.[9,10] This could explain why Fe(1) in **1[BF$_4$]$_4$**·2MeCN·$Et_2O$ remains high-spin at low temperature, for example (Table S3, below).

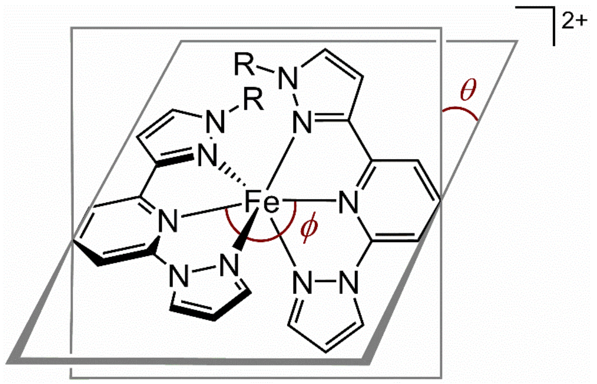


**Scheme S5** Definition of the Jahn-Teller distortion parameters $\theta$ and $\phi$ (R = the ditopic ligand linker group).

Typical values of all these parameters for $[Fe(bpp)_2]^{2+}$ derivatives are listed in refs. [7] and [11]-[13].

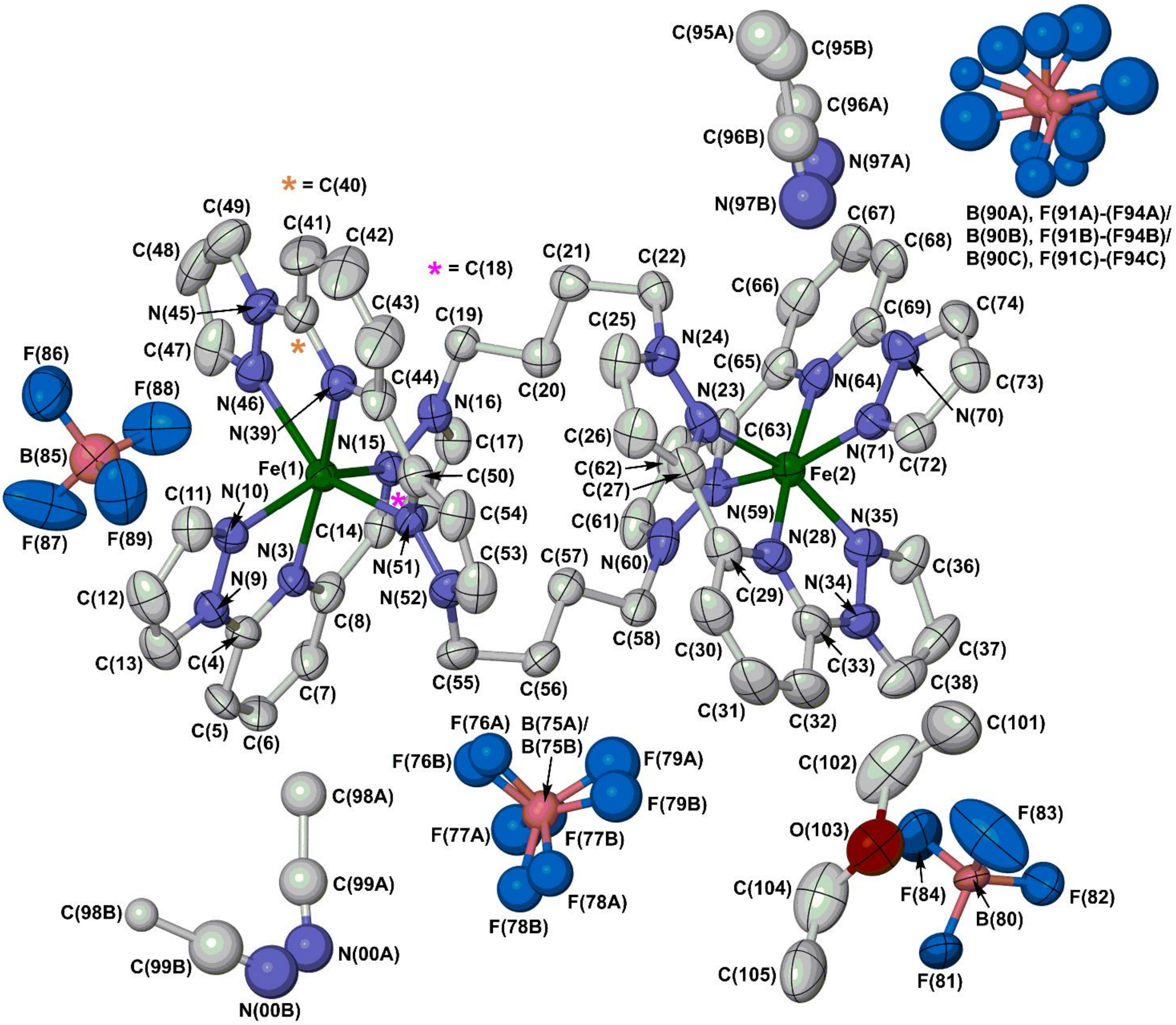


**Figure S14** The formula unit in the crystal structure of $[Fe_2(\mu\text{-}L^1)_2][BF_4]_4\cdot 2MeCN\cdot Et_2O$ ($\mathbf{1[BF_4]_4}\cdot 2MeCN\cdot Et_2O$), showing the full atom numbering scheme. Displacement ellipsoids are at the 50 % probability level, and H atoms are omitted for clarity.

Colour code: C, white; B, pink; F, cyan; Fe, green; N, blue; O, red.

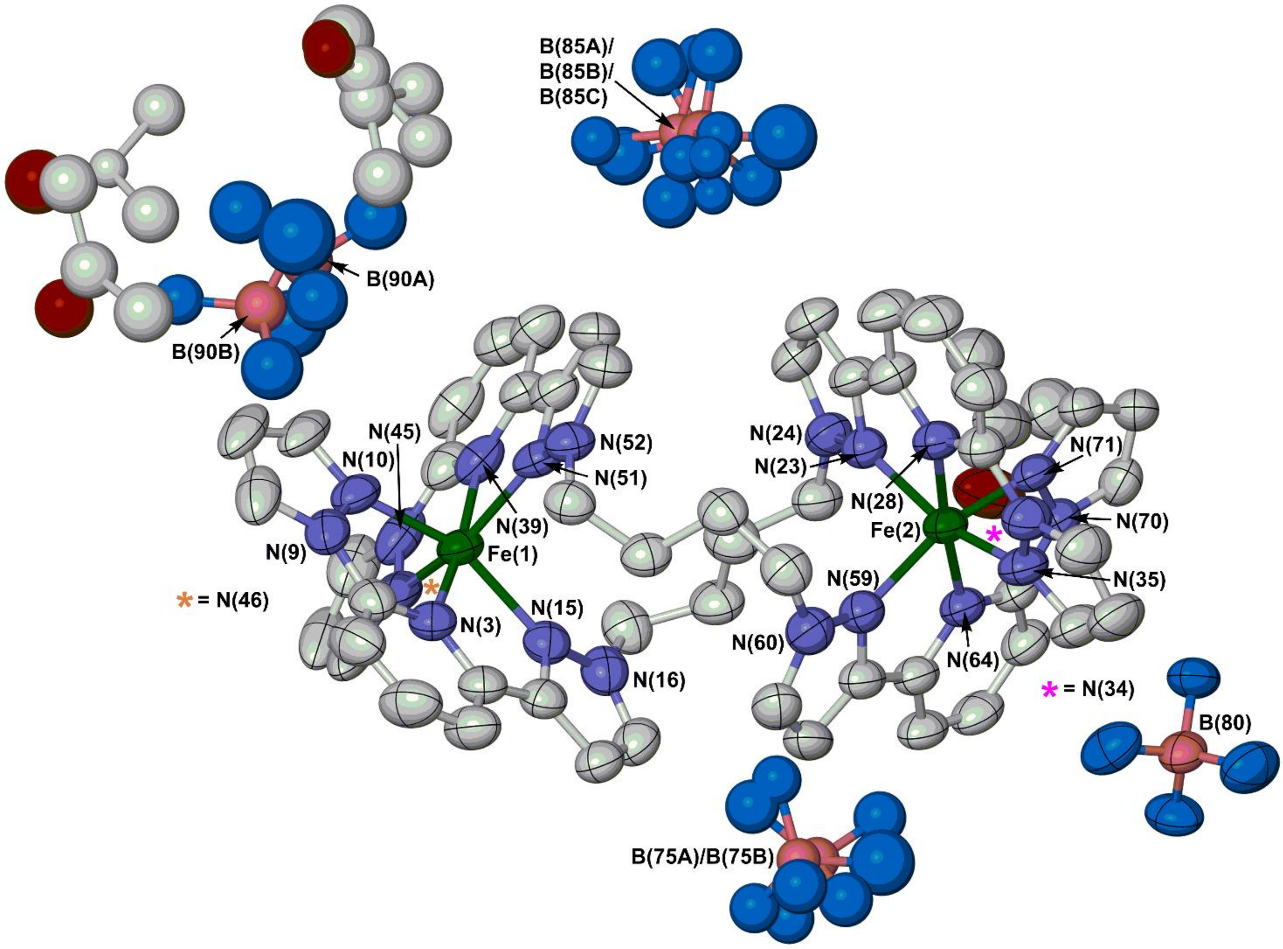


**Figure S15** The formula unit in the crystal structure of $[Fe_2(\mu\text{-}L^1)_2][BF_4]_4{\cdot}n Me_2CO$ (**1[BF$_4$]$_4$**$\cdot n Me_2CO$; $n \approx 2.5$) at 100 K, with selected atom numbering. Details as for Figure S14.

Colour code: C, white; B, pink; F, cyan; Fe, green; N, blue; O, red.

The full atom numbering of the cations and anions in this refinement corresponds to that in Figure S14.

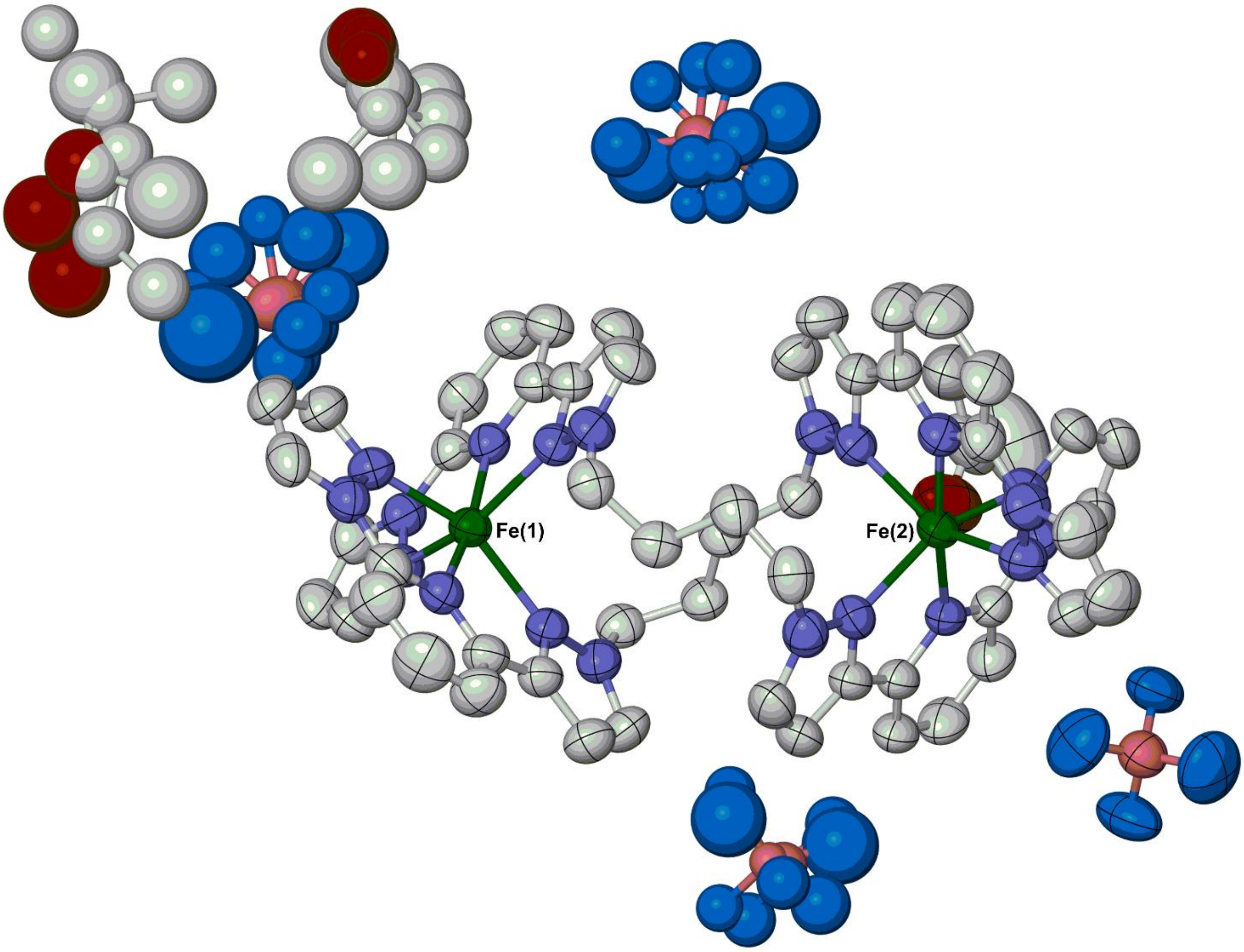


**Figure S16** The formula unit in the crystal structure of $[Fe_2(\mu\text{-}L^1)_2][BF_4]_4 \cdot n Me_2CO$ (**$1[BF_4]_4$**$\cdot n Me_2CO$; $n \approx 2.5$) at 250 K, with selected atom numbering. Details as for Figure S14. Colour code: C, white; B, pink; F, cyan; Fe, green; N, blue; O, red.

The full atom numbering of the cations and anions in this refinement corresponds to that in Figure S14 and S15.

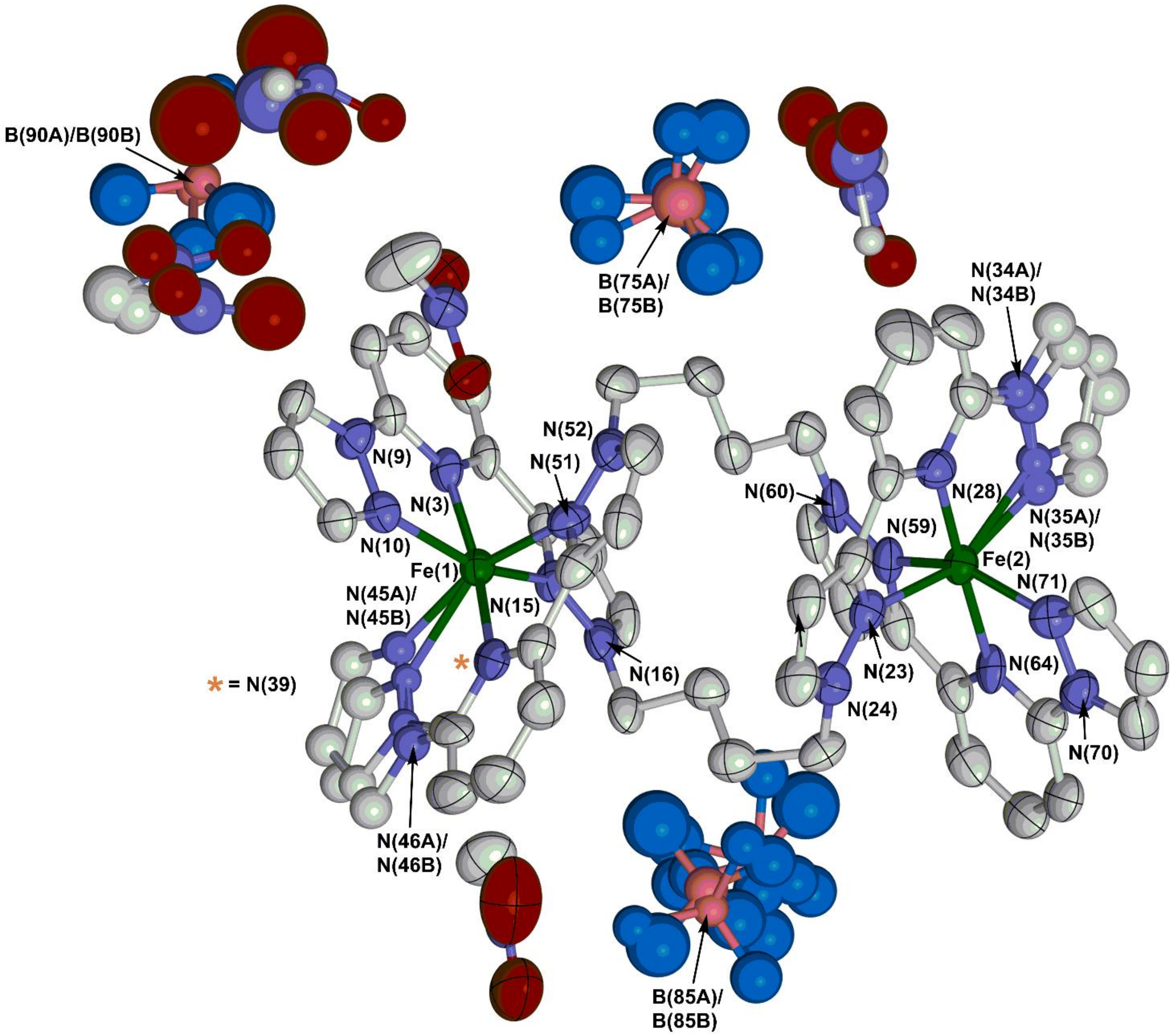


**Figure S17** The formula unit in the crystal structure of $[Fe_2(\mu\text{-}L^1)_2][BF_4]_4{\cdot}m\text{MeNO}_2$ (**1[BF$_4$]$_4$**$\cdot m\text{Me}_2\text{CO}$; $m \approx$ 4.5), with selected atom numbering. Details as for Figure S14.

Colour code: C, white; B, pink; F, cyan; Fe, green; N, blue; O, red.

The full atom numbering of the cations and anions in this refinement corresponds to that in Figures S14. Atom B(80) is hidden behind the disordered anion B(85)-F(89).

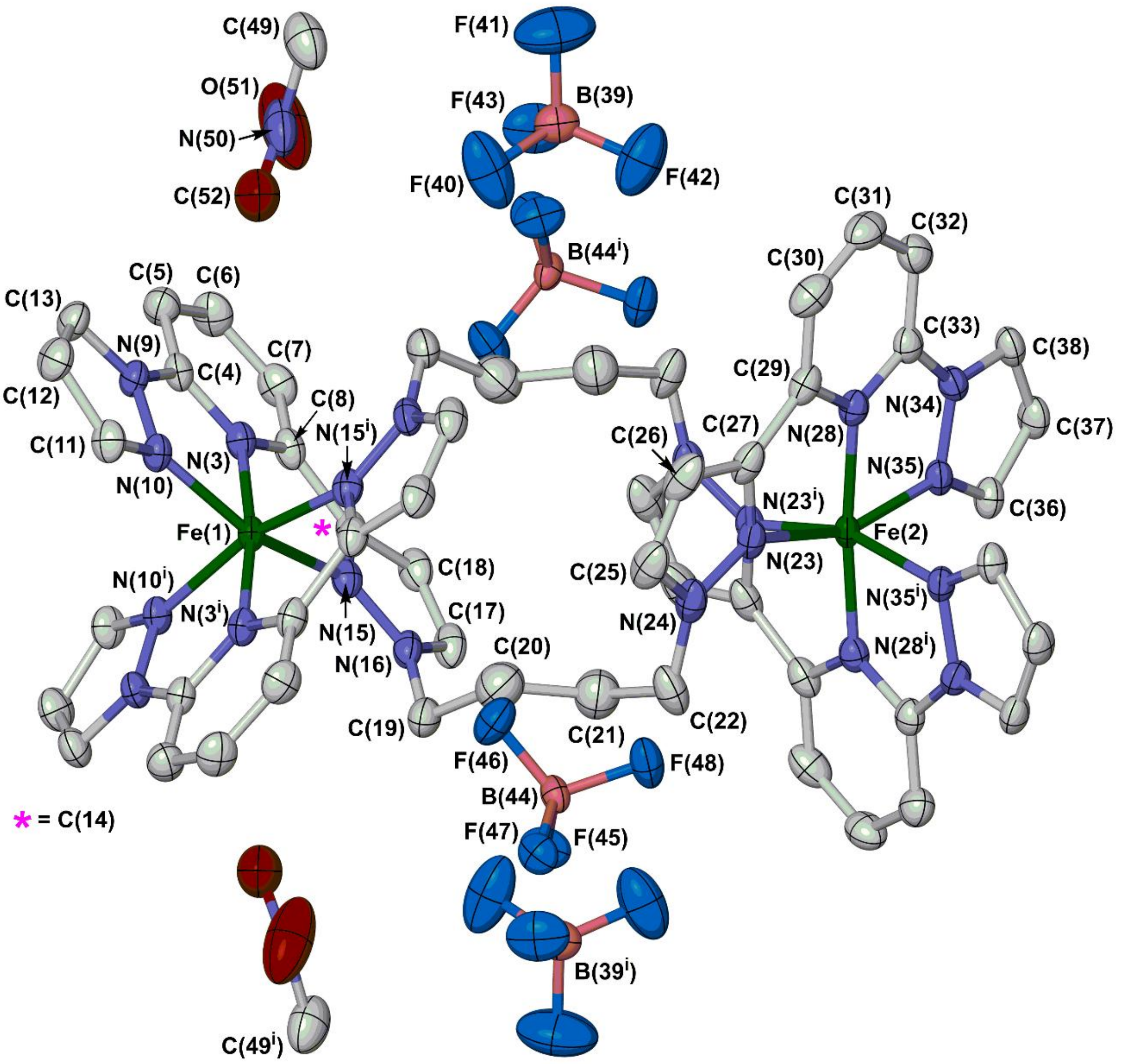


**Figure S18** The formula unit in the crystal structure of $[Fe_2(\mu\text{-}L^1)_2][BF_4]_4{\cdot}2MeNO_2$ (**1[BF$_4$]$_4$**$\cdot 2MeNO_2$), showing the full atom numbering scheme. Details as for Figure S14. Symmetry code: (i) $-x, y, -z$.

Colour code: C, white; B, pink; F, cyan; Fe, green; N, blue; O, red.

**Table S3** Selected bond lengths, angles and structural parameters [Å , °, Å$^3$] for $[Fe_2(\mu\text{-}L^1)_2][BF_4]_4\cdot 2MeCN\cdot Et_2O$ (**1[BF$_4$]$_4$**$\cdot$2MeCN$\cdot$Et$_2$O) and $[Fe_2(\mu\text{-}L^1)_2][BF_4]_4\cdot n Me_2CO$ (**1[BF$_4$]$_4$**$\cdot n$Me$_2$CO). See Figures S14-S16 for the atom numbering scheme, while definitions of $V_{Oh}$, $\Sigma$, $\Theta$, $\varphi$ and $\theta$ are given on page S20.

| | **1[BF$_4$]$_4$**$\cdot$2MeCN$\cdot$Et$_2$O | **1[BF$_4$]$_4$**$\cdot n$Me$_2$CO | |
|---|---|---|---|
| *T* | 125 K | 250 K | 100 K |
| Fe(1)–N(3) | 2.117(6) | 2.116(3) | 2.038(4) |
| Fe(1)–N(10) | 2.169(7) | 2.171(3) | 2.091(4) |
| Fe(1)–N(15) | 2.153(6) | 2.177(3) | 2.110(4) |
| Fe(1)–N(39) | 2.126(6) | 2.106(3) | 2.046(4) |
| Fe(1)–N(46) | 2.207(8) | 2.173(3) | 2.058(5) |
| Fe(1)–N(51) | 2.215(7) | 2.211(3) | 2.142(4) |
| Fe(2)–N(23) | 2.040(8) | 2.189(3) | 2.036(4) |
| Fe(2)–N(28) | 1.923(6) | 2.103(3) | 1.931(4) |
| Fe(2)–N(35) | 1.981(8) | 2.161(3) | 1.965(4) |
| Fe(2)–N(59) | 2.012(7) | 2.180(3) | 2.037(4) |
| Fe(2)–N(64) | 1.938(7) | 2.096(2) | 1.939(3) |
| Fe(2)–N(71) | 1.963(7) | 2.165(3) | 1.979(4) |
| Fe(1)···Fe(2) | 7.8570(18) | 8.1477(6) | 8.1221(9) |
| | | | |
| N(3)–Fe(1)–N(10) | 73.9(3) | 73.99(10) | 75.85(16) |
| N(3)–Fe(1)–N(15) | 74.5(3) | 74.91(10) | 76.29(14) |
| N(3)–Fe(1)–N(39) | 167.9(3) | 168.53(10) | 171.54(14) |
| N(3)–Fe(1)–N(46) | 112.2(3) | 110.82(10) | 107.32(19) |
| N(3)–Fe(1)–N(51) | 101.5(3) | 100.22(10) | 100.81(14) |
| N(10)–Fe(1)–N(15) | 148.0(3) | 148.54(10) | 151.86(16) |
| N(10)–Fe(1)–N(39) | 94.6(3) | 95.83(10) | 96.63(16) |
| N(10)–Fe(1)–N(46) | 100.1(3) | 92.79(11) | 93.40(17) |
| N(10)–Fe(1)–N(51) | 88.3(3) | 93.08(10) | 91.54(15) |
| N(15)–Fe(1)–N(39) | 117.3(3) | 115.57(10) | 111.43(15) |
| N(15)–Fe(1)–N(46) | 87.6(3) | 93.80(10) | 91.09(15) |
| N(15)–Fe(1)–N(51) | 102.4(3) | 96.96(10) | 97.48(14) |
| N(39)–Fe(1)–N(46) | 72.8(3) | 74.32(10) | 76.6(2) |
| N(39)–Fe(1)–N(51) | 73.9(3) | 74.64(10) | 75.23(15) |
| N(46)–Fe(1)–N(51) | 146.2(3) | 148.83(10) | 151.80(19) |
| N(23)–Fe(2)–N(28) | 79.9(3) | 75.03(10) | 78.33(15) |
| N(23)–Fe(2)–N(35) | 159.4(3) | 149.09(11) | 158.01(15) |
| N(23)–Fe(2)–N(59) | 94.1(3) | 97.57(10) | 96.25(15) |
| N(23)–Fe(2)–N(64) | 101.2(3) | 104.43(10) | 102.73(15) |
| N(23)–Fe(2)–N(71) | 91.1(3) | 89.74(10) | 89.02(14) |
| N(28)–Fe(2)–N(35) | 79.6(3) | 74.09(11) | 79.74(16) |
| N(28)–Fe(2)–N(59) | 106.6(3) | 111.50(11) | 105.61(16) |
| N(28)–Fe(2)–N(64) | 173.4(3) | 173.69(11) | 175.52(16) |
| N(28)–Fe(2)–N(71) | 93.8(3) | 99.38(11) | 96.04(16) |
| N(35)–Fe(2)–N(59) | 90.0(3) | 94.87(11) | 91.08(16) |
| N(35)–Fe(2)–N(64) | 99.4(3) | 106.10(10) | 99.02(15) |
| N(35)–Fe(2)–N(71) | 92.0(3) | 93.93(11) | 91.78(17) |
| N(59)–Fe(2)–N(64) | 79.9(3) | 74.81(10) | 78.67(15) |
| N(59)–Fe(2)–N(71) | 79.7(3) | 149.11(11) | 158.33(15) |
| N(64)–Fe(2)–N(71) | 159.5(3) | 74.31(10) | 79.67(15) |
| | | | |
| $V_{Oh}$ {Fe(1), Fe(2)} | 12.19(3), 9.92(2) | 12.318(10), 12.249(10) | 11.231(14), 9.965(12) |
| $\Sigma$ {Fe(1), Fe(2)} | 157(1), 89(1) | 141.2(3), 139.8(4) | 125.7(6), 97.1(5) |
| $\Theta$ {Fe(1), Fe(2)} | 493, 295 | 466, 458 | 483, 313 |
| $\varphi$ {Fe(1), Fe(2)} | 167.9(3), 173.4(3) | 168.53(10), 173.69(11) | 171.54(14), 175.52(16) |
| $\theta$ {Fe(1), Fe(2)} | 73.87(7), 84.22(7) | 85.96(3), 83.62(3) | 85.43(4), 84.30(4) |

**Table S4** Selected bond lengths, angles and structural parameters [Å , °, Å$^3$] for the crystal of $[Fe_2(\mu\text{-}L^1)_2][BF_4]_4\cdot m$MeNO$_2$ (**1[BF$_4$]$_4$**·$m$MeNO$_2$; $m \approx 4.5$) and $[Fe_2(\mu\text{-}L^1)_2][BF_4]_4\cdot$2MeNO$_2$ (**1[BF$_4$]$_4$**·2MeNO$_2$) at 125 K. See Figures S17-S18 for the atom numbering scheme, while definitions of $V_{Oh}$, $\Sigma$, $\Theta$, $\varphi$ and $\theta$ are given on page S20. Symmetry code: (i) –*x*, *y*, –*z*.

| | **1[BF$_4$]$_4$**·$m$MeNO$_2$ | | **1[BF$_4$]$_4$**·2MeNO$_2$[b] |
|---|---|---|---|
| Fe(1)–N(3) | 2.040(9) | Fe(1)–N(3) | 1.917(4) |
| Fe(1)–N(10) | 2.052(9) | Fe(1)–N(10) | 1.962(4) |
| Fe(1)–N(15) | 2.107(9) | Fe(1)–N(15) | 1.999(4) |
| Fe(1)–N(39) | 2.041(10) | | |
| Fe(1)–N(46)[a] | 2.232(18)/2.008(17) | | |
| Fe(1)–N(51) | 2.135(10) | | |
| Fe(2)–N(23) | 2.136(9) | Fe(2)–N(23) | 2.001(4) |
| Fe(2)–N(28) | 2.013(9) | Fe(2)–N(28) | 1.906(4) |
| Fe(2)–N(35)[a] | 2.20(2)/1.97(2) | Fe(2)–N(35) | 1.965(3) |
| Fe(2)–N(59) | 2.077(10) | | |
| Fe(2)–N(64) | 2.025(10) | | |
| Fe(2)–N(71) | 2.079(11) | | |
| Fe(1)···Fe(2) | 7.8406(22) | Fe(1)···Fe(2) | 8.5452(11) |
| | | | |
| N(3)–Fe(1)–N(10) | 76.0(3) | N(3)–Fe(1)–N(10) | 80.24(15) |
| N(3)–Fe(1)–N(15) | 75.9(4) | N(3)–Fe(1)–N(15) | 79.40(15) |
| N(3)–Fe(1)–N(39) | 170.6(3) | N(3)–Fe(1)–N(3$^i$) | 171.8(2) |
| N(3)–Fe(1)–N(46)[a] | 110.0(6)/96.7(7) | N(3)–Fe(1)–N(10$^i$) | 94.09(15) |
| N(3)–Fe(1)–N(51) | 104.3(4) | N(3)–Fe(1)–N(15$^i$) | 106.39(15) |
| N(10)–Fe(1)–N(15) | 151.5(4) | N(10)–Fe(1)–N(15) | 159.52(15) |
| N(10)–Fe(1)–N(39) | 94.6(4) | N(10)–Fe(1)–N(10$^i$) | 93.1(2) |
| N(10)–Fe(1)–N(46)[a] | 96.2(5)/90.6(6) | N(10)–Fe(1)–N(15$^i$) | 90.58(14) |
| N(10)–Fe(1)–N(51) | 93.3(4) | N(15)–Fe(1)–N(15$^i$) | 93.0(2) |
| N(15)–Fe(1)–N(39) | 113.4(4) | | |
| N(15)–Fe(1)–N(46)[a] | 89.1(5)/88.2(6) | | |
| N(15)–Fe(1)–N(51) | 98.0(4) | | |
| N(39)–Fe(1)–N(46)[a] | 70.0(6)/82.7(7) | | |
| N(39)–Fe(1)–N(51) | 76.4(4) | | |
| N(46)–Fe(1)–N(51)[a] | 145.7(6)/159.0(7) | | |
| N(23)–Fe(2)–N(28) | 76.7(4) | N(23)–Fe(2)–N(28) | 79.65(16) |
| N(23)–Fe(2)–N(35)[a] | 148.9(7)/158.3(8) | N(23)–Fe(2)–N(35) | 159.78(15) |
| N(23)–Fe(2)–N(59) | 96.4(4) | N(23)–Fe(2)–N(23$^i$) | 92.6(2) |
| N(23)–Fe(2)–N(64) | 100.4(4) | N(23)–Fe(2)–N(28$^i$) | 104.51(16) |
| N(23)–Fe(2)–N(71) | 90.3(4) | N(23)–Fe(2)–N(35$^i$) | 89.98(14) |
| N(28)–Fe(2)–N(35)[a] | 72.5(7)/81.7(8) | N(28)–Fe(2)–N(35) | 80.29(15) |
| N(28)–Fe(2)–N(59) | 110.6(4) | | |
| N(28)–Fe(2)–N(64) | 172.8(4) | N(28)–Fe(2)–N(28$^i$) | 174.1(2) |
| N(28)–Fe(2)–N(71) | 96.3(4) | N(28)–Fe(2)–N(35$^i$) | 95.69(15) |
| N(35)–Fe(2)–N(59)[a] | 90.8(6)/90.0(7) | | |
| N(35)–Fe(2)–N(64)[a] | 110.7(7)/101.2(8) | | |
| N(35)–Fe(2)–N(71)[a] | 96.7(6)/93.2(8) | N(35)–Fe(2)–N(35$^i$) | 94.5(2) |
| N(59)–Fe(2)–N(64) | 76.1(4) | | |
| N(59)–Fe(2)–N(71) | 153.1(4) | | |
| N(64)–Fe(2)–N(71) | 77.1(4) | | |
| | | | |
| $V_{Oh}$ {Fe(1), Fe(2)}[a] | 11.32(4)/11.06(4), 11.27(4)/10.91(4) | $V_{Oh}$ {Fe(1), Fe(2)} | 9.649(13), 9.651(12) |
| $\Sigma$ {Fe(1), Fe(2)}[a] | 142.4(16)/111.7(18), 129.8(17)/106.8(19) | $\Sigma$ {Fe(1), Fe(2)} | 88.9(5), 87.7(6) |
| $\Theta$ {Fe(1), Fe(2)}[a] | 469/357, 430/352 | $\Theta$ {Fe(1), Fe(2)} | 295, 290 |
| $\varphi$ {Fe(1), Fe(2)} | 170.6(3), 172.8(4) | $\varphi$ {Fe(1), Fe(2)} | 171.8(2), 174.1(2) |
| $\theta$ {Fe(1), Fe(2)}[a] | 79.46(10)/81.08(9), 79.65(11)/80.42(12) | $\theta$ {Fe(1), Fe(2)} | 87.66(3), 84.28(3) |

[a]This parameter involves an N atom which is disordered over two orientations. [b]The asymmetric unit of this crystal contains half a molecule of the complex, with Fe(1) and Fe(2) lying on a crystallographic $C_2$ axis.

(*a*)

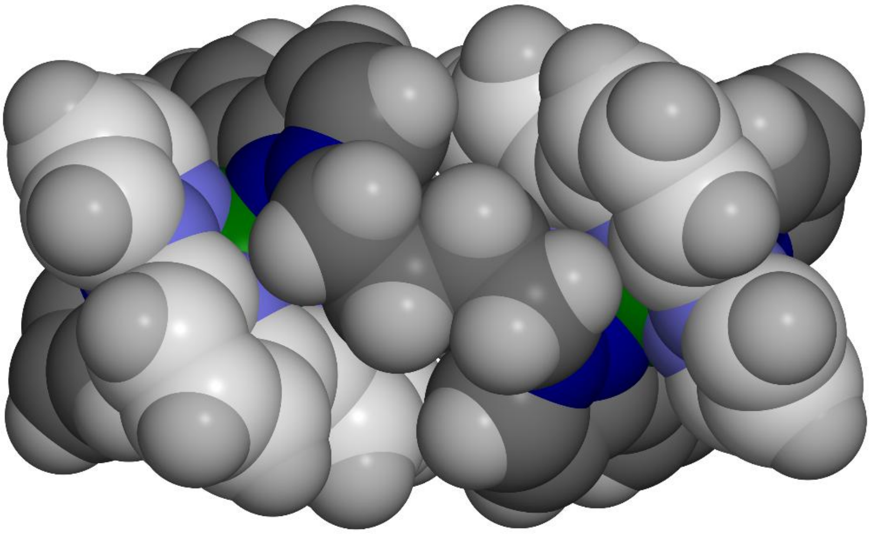

(*b*)

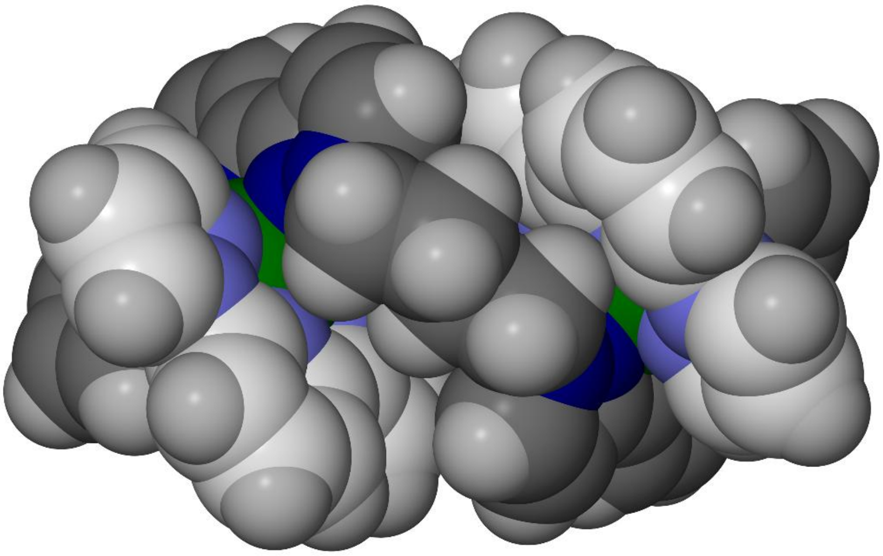

(*c*)

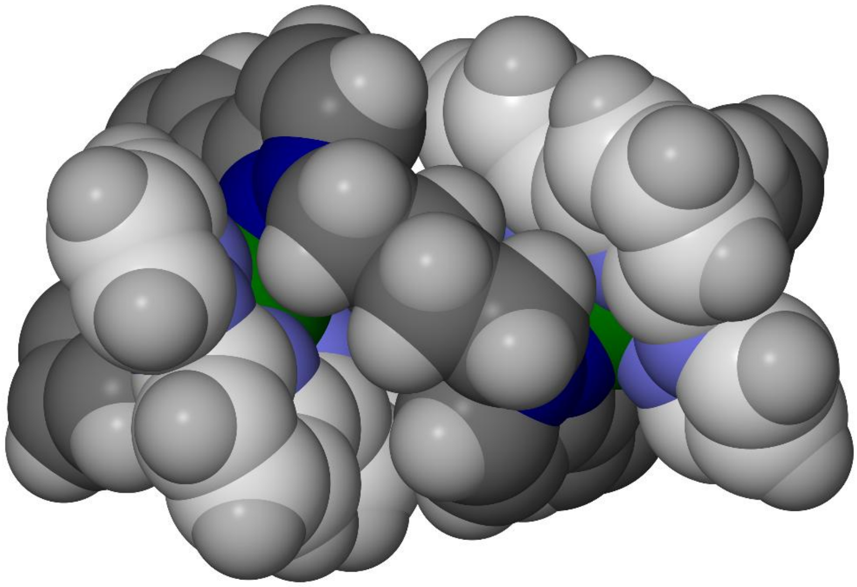

**Figure S19** Space-filling views of the three conformations of the $[Fe_2(\mu\text{-}L^1)_2]^{4+}$ helicate in: (a) $\mathbf{1[BF_4]_4}\cdot 2MeNO_2$; (b) $\mathbf{1[BF_4]_4}\cdot n Me_2CO$ at 100 K; and (c) $\mathbf{1[BF_4]_4}\cdot 2MeCN\cdot Et_2O$. The views are the same as in Figure 1 of the main article. The $L^1$ ligands in each molecule are distinguished with pale and dark coloration, and H atoms are omitted for clarity. Color code: C, white or gray; N, pale or dark blue; Fe, green.

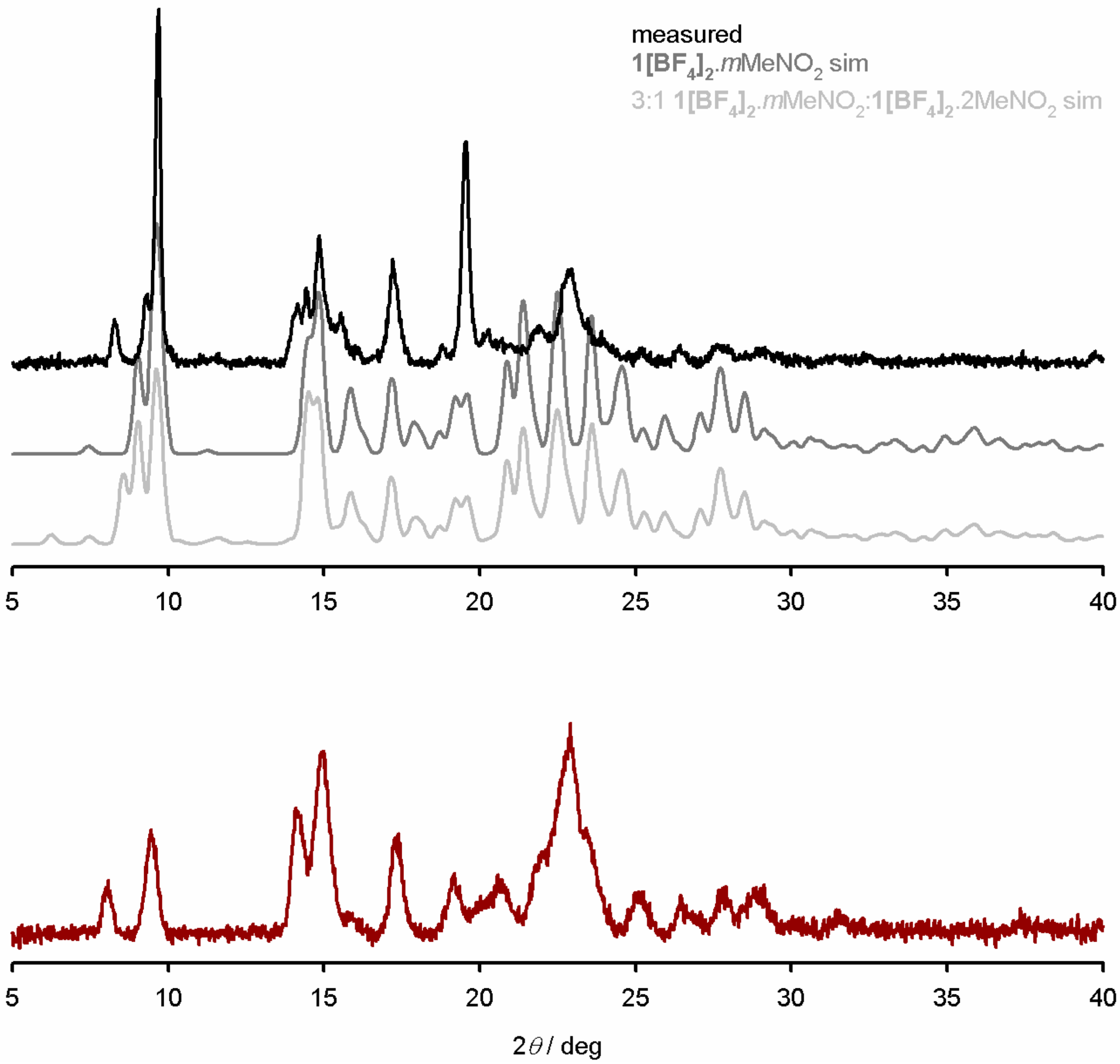


**Figure S20** Room temperature X-ray powder diffraction patterns for **1[BF$_4$]$_4$** (black) and **1[ClO$_4$]$_4$** (red), crystallized from nitromethane solution using diethyl ether antisolvent. Simulated data from the pure **1[BF$_4$]$_4$**·*m*MeNO$_2$ ($m \approx 4.5$) phase, and of a 3:1 mixture of both nitromethane solvate phases, are also shown (gray).

Needles of **1[BF$_4$]$_4$**·*m*MeNO$_2$ are the major component in the freshly crystallized material. The powder sample is poorly crystalline, which presumably reflects solvent loss. However, the features of its powder pattern show some similarity to the **1[BF$_4$]$_4$**·*m*MeNO$_2$ phase. A contribution from the minor, prismatic **1[BF$_4$]$_4$**·2MeNO$_2$ crystal phase makes only a small difference to the simulation however, and can't be discounted from these data.

Freshly crystallized **1[ClO$_4$]$_4$**·*m*MeNO$_2$ also has a needle morphology, and this air-dried sample is similarly poorly crystalline. Its powder pattern shows similarities to the corresponding $BF_4^-$ solvate salt however, which is consistent with the similar magnetic susceptibility data shown by the two materials (Figure S23).

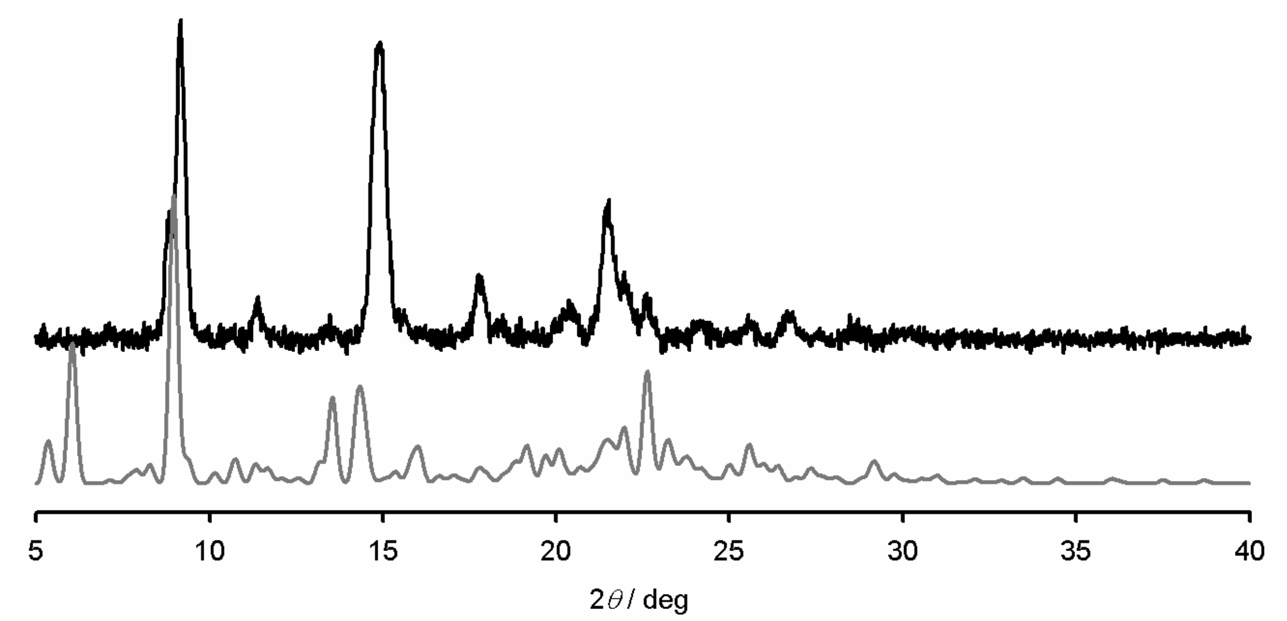


**Figure S21** Room temperature X-ray powder diffraction pattern for **1[BF$_4$]$_4$**, crystallized from acetone solution using diethyl ether antisolvent (black), and a simulation of the crystallographic **1[BF$_4$]$_4$**·*n*Me$_2$CO phase (gray).

The sample has lost much of its crystallinity due to desolvation, but it should retain some features of the crystallographic structure according to these data. Reflecting that, the two-step SCO observed in the magnetic data from this sample are consistent with the predictions from the crystal structure.

A powder pattern of **1[ClO$_4$]$_4$** crystallized from acetone solution was not obtained, because **1[ClO$_4$]$_4$** is so sparingly soluble in that solvent that its acetone solvate could only be obtained in mg quantities. Magnetic data strongly suggest it is isomorphous with **1[BF$_4$]$_4$**·*n*Me$_2$CO, however (Figure S24).

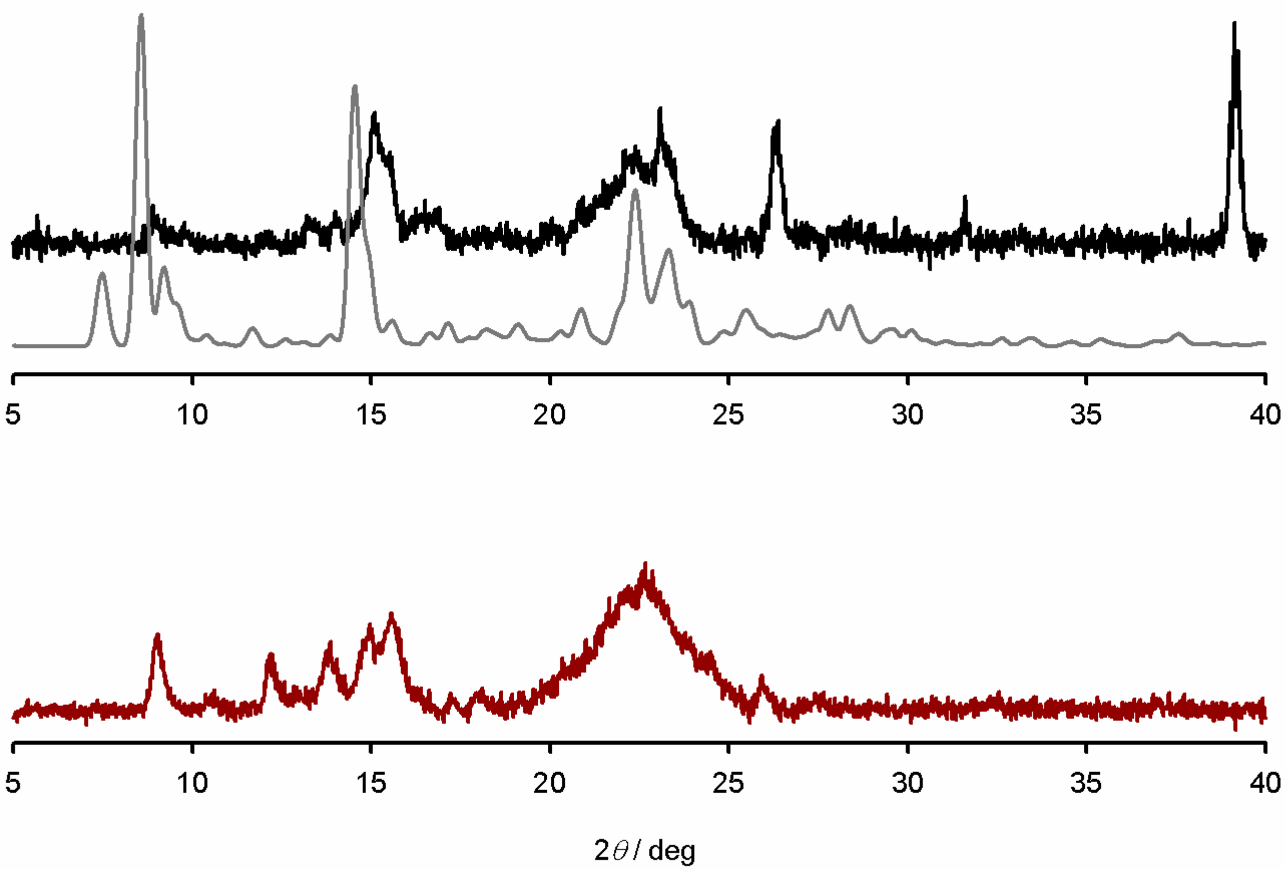


**Figure S20** Room temperature X-ray powder diffraction patterns for **1[BF$_4$]$_4$** (black) and **1[ClO$_4$]$_4$** (red), crystallized from acetonitrile solution using diethyl ether antisolvent. Simulated data from the **1[BF$_4$]$_4$**·2MeCN·Et$_2$O crystal phase are also included (gray).

While both powder patterns include similar features, the materials are very poorly crystalline, with a clear amorphous hump around $2\theta = 22°$ being present in the perchlorate salt. That may be a consequence of solvent loss from the samples upon exposure to air. Be that as it may, their poor crystallinity is consistent with the very gradual, incomplete SCO observed in their magnetic data (Figure S25).

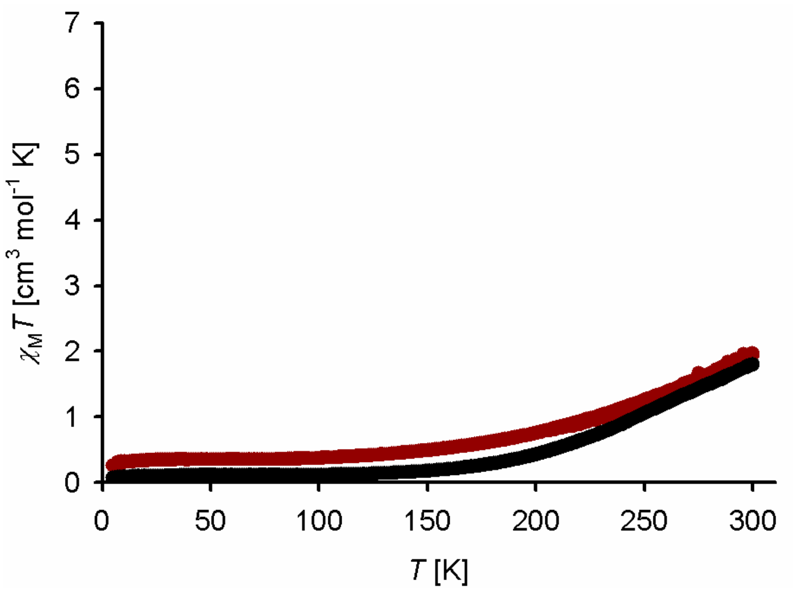


**Figure S23** Variable temperature magnetic susceptibility data for **1[BF$_4$]$_2$** (black) and **1[ClO$_4$]$_2$** (red), crystallized from nitromethane/diethyl ether solution. The $BF_4^-$ data are the same as in Figure 2 of the main article.

The spin state properties of these samples are essentially identical, reflecting their isomorphous nature (Figure S20).

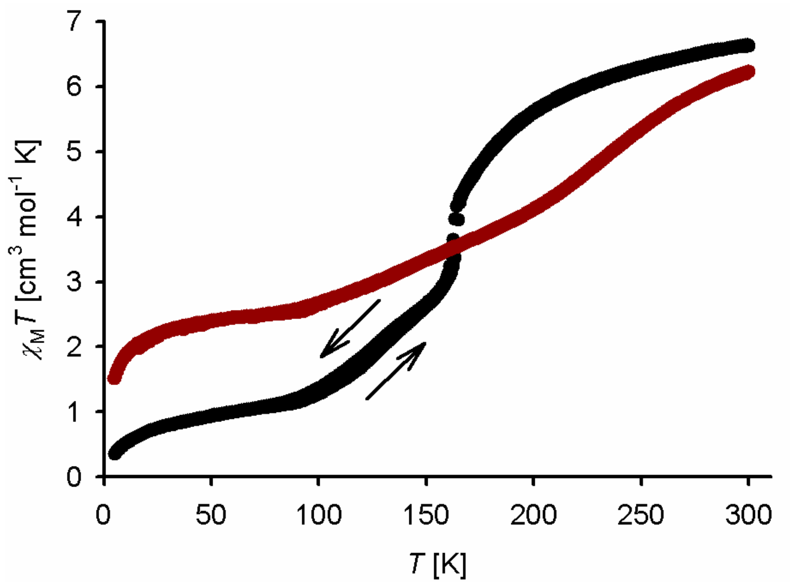


**Figure S24** Variable temperature magnetic susceptibility data for **1[BF$_4$]$_2$** (black) and **1[ClO$_4$]$_2$** (red), crystallized from acetone/diethyl ether solution. The $BF_4^-$ data are the same as in Figure 2 of the main article.

SCO in the $ClO_4^-$ sample occurs more gradually than in the $BF_4^-$ salt, and is less complete below 100 K. However, both materials show a clear discontinuity in their transition, which is consistent with the crystal structures of **1[BF$_4$]$_4$**·$n$Me$_2$CO.

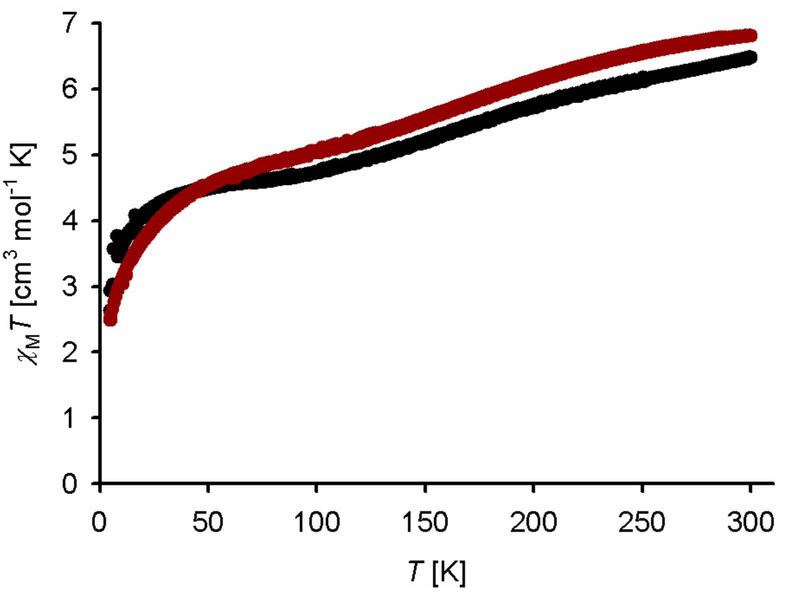


**Figure S25** Variable temperature magnetic susceptibility data for **1[BF$_4$]$_2$** (black) and **1[ClO$_4$]$_2$** (red), crystallized from acetonitrile/diethyl ether solution. The $BF_4^-$ data are the same as in Figure 2 of the main article.

These samples are essentially amorphous by powder diffraction (Figure S20), with is consistent with their very gradual and ill-defined thermal SCO.

Crystallographically, **1[BF$_4$]$_4$**·2MeCN·Et$_2$O contains one high-spin and one low-spin iron centre at 125 K; that is, a 50% low-spin population. That could be consistent with the partial SCO in these magnetic data, where the sample is *ca* 30 % low-spin at 100 K. However, the amorphous nature of the powder samples means the relationship between the two measurements should be interpreted with care.

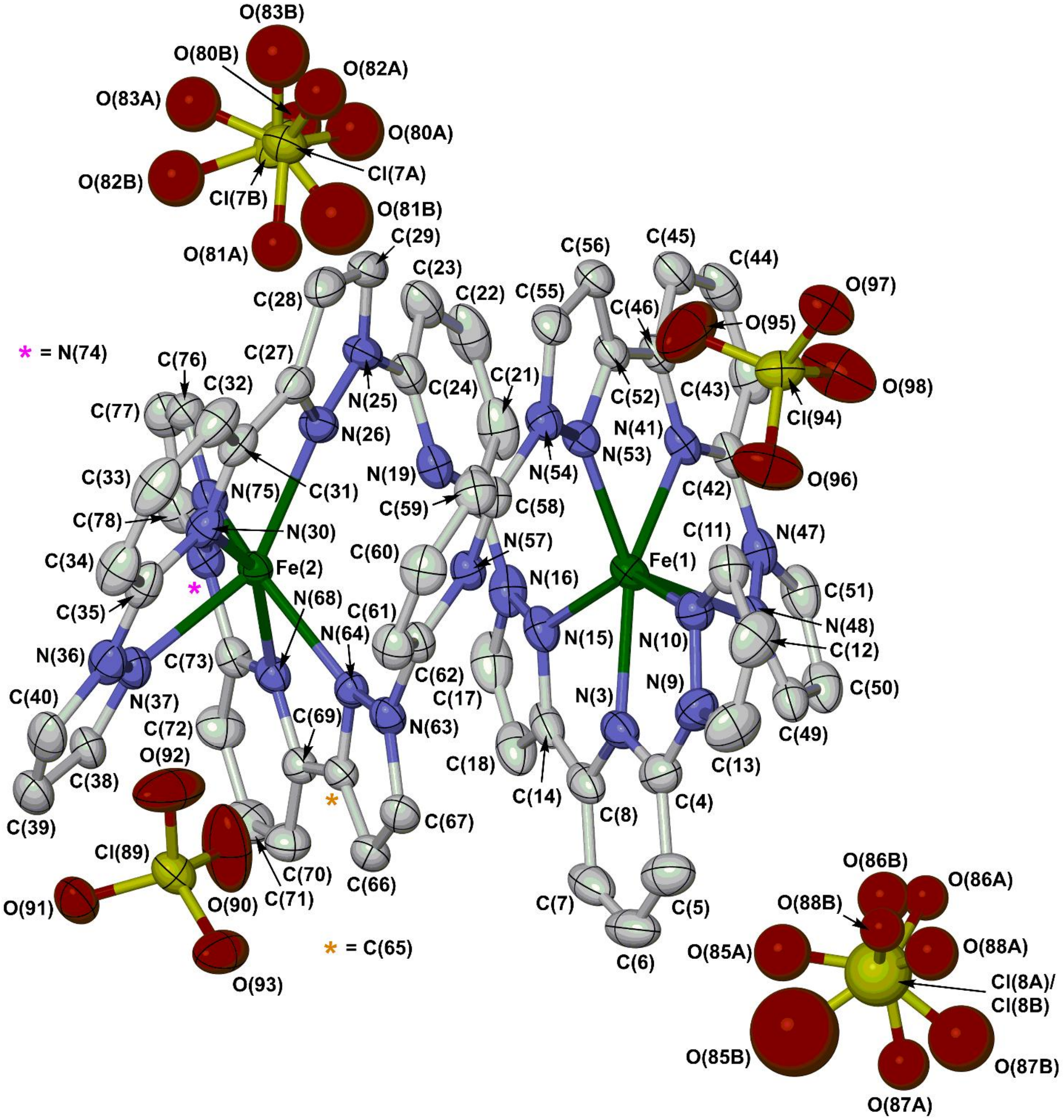


**Figure S26** The asymmetric unit of **2[ClO$_4$]$_4$**, showing the full atom numbering scheme. Displacement ellipsoids are at the 50 % probability level, and H atoms have been removed for clarity. Colour code: C, white; Cl, yellow; Fe, green; N, blue; O, red.

Atom C(20) is obscured behind C(58) and C(59).

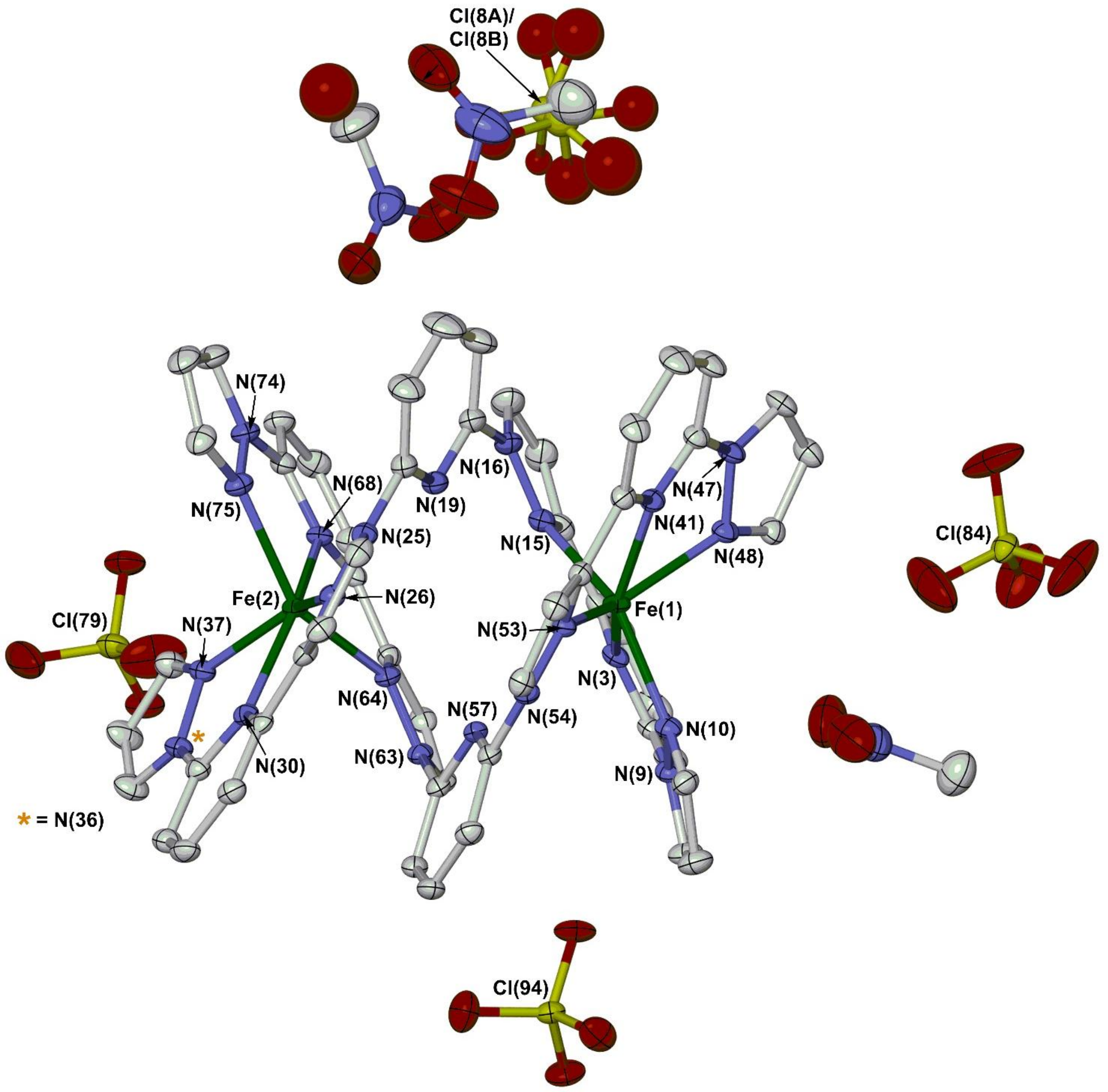


**Figure S27** The asymmetric unit of **2[ClO$_4$]$_4$**·3MeNO$_2$·0.75H$_2$O, with a partial atom numbering scheme. Displacement ellipsoids are at the 50 % probability level, and H atoms have been removed for clarity. Colour code: C, white; Cl, yellow; Fe, green; N, blue; O, red.

The full atom numbering of the cations and anions in this refinement corresponds to that in Figure S26.

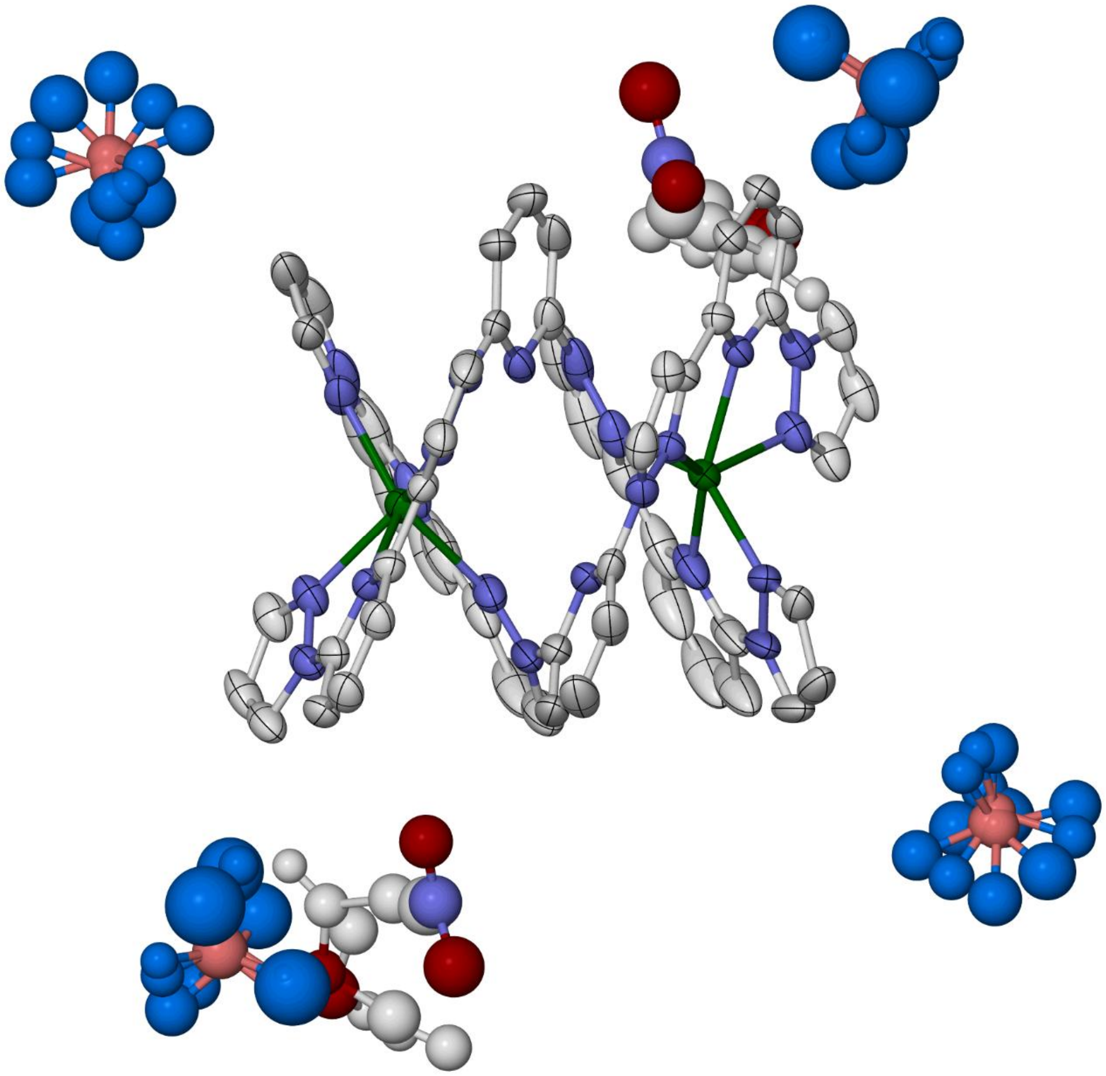

**Figure S28** The formula unit in a partial crystallographic refinement of **$2[BF_4]_4$**$\cdot MeNO_2\cdot Et_2O$. Displacement ellipsoids are at the 50 % probability level, and H atoms have been removed for clarity. Colour code: C, white; B, pink; F, cyan; Fe, green; N, blue; O, red.

The geometry of the helicate in this crystal is essentially identical to the two **$2[ClO_4]_4$** crystal refinements (Table S5). For example, the Fe···Fe distance in the complex is 5.1285(18) Å.

The crystal has a high mosaicity, reflecting the extensive anion and solvent disorder. Large, elongated displacement ellipsoids on some C atoms imply the disorder might also extend to parts of the ligand backbone. Since the two **$2[ClO_4]_4$** structure refinements are of higher quality, a better dataset of this crystal was not pursued and the structure has not been deposited with the CCDC.

**Table S5** Selected bond lengths, angles and structural parameters [Å , °, Å$^3$] for the crystals of $[Fe_2(\mu\text{-}L^2)_2][ClO_4]_4$ (**2[ClO$_4$]$_4$**). See Figures S26-S27 for the atom numbering scheme, while definitions of $V_{Oh}$, $\Sigma$, $\Theta$, $\varphi$ and $\theta$ are given on page S20.

| | **2[ClO$_4$]$_4$** | **2[ClO$_4$]$_4$**·3MeNO$_2$·0.75H$_2$O |
|---|---|---|
| Fe(1)–N(3) | 2.175(2) | 2.161(2) |
| Fe(1)–N(10) | 2.185(3) | 2.168(2) |
| Fe(1)–N(15) | 2.233(2) | 2.242(3) |
| Fe(1)–N(39) | 2.207(2) | 2.199(2) |
| Fe(1)–N(46) | 2.352(2) | 2.290(2) |
| Fe(1)–N(51) | 2.196(2) | 2.190(2) |
| Fe(2)–N(23) | 2.209(2) | 2.200(2) |
| Fe(2)–N(28) | 2.169(2) | 2.188(2) |
| Fe(2)–N(35) | 2.241(2) | 2.226(2) |
| Fe(2)–N(59) | 2.244(2) | 2.227(2) |
| Fe(2)–N(64) | 2.167(2) | 2.175(2) |
| Fe(2)–N(71) | 2.191(2) | 2.192(2) |
| Fe(1)···Fe(2) | 5.1401(6) | 5.1906(6) |
| | | |
| N(3)–Fe(1)–N(10) | 72.42(9) | 73.56(9) |
| N(3)–Fe(1)–N(15) | 71.90(10) | 71.57(9) |
| N(3)–Fe(1)–N(39) | 148.68(9) | 148.18(9) |
| N(3)–Fe(1)–N(46) | 80.66(9) | 80.77(9) |
| N(3)–Fe(1)–N(51) | 139.79(9) | 139.89(9) |
| N(10)–Fe(1)–N(15) | 143.69(9) | 143.59(9) |
| N(10)–Fe(1)–N(39) | 120.93(9) | 123.12(9) |
| N(10)–Fe(1)–N(46) | 101.85(9) | 101.87(9) |
| N(10)–Fe(1)–N(51) | 85.29(9) | 85.78(9) |
| N(15)–Fe(1)–N(39) | 93.58(9) | 92.58(9) |
| N(15)–Fe(1)–N(46) | 78.78(9) | 82.64(9) |
| N(15)–Fe(1)–N(51) | 118.94(9) | 115.08(9) |
| N(39)–Fe(1)–N(46) | 69.16(9) | 69.71(9) |
| N(39)–Fe(1)–N(51) | 71.52(9) | 71.67(9) |
| N(46)–Fe(1)–N(51) | 137.70(8) | 138.02(9) |
| N(23)–Fe(2)–N(28) | 72.19(9) | 71.68(9) |
| N(23)–Fe(2)–N(35) | 141.29(10) | 141.17(9) |
| N(23)–Fe(2)–N(59) | 110.89(9) | 112.76(9) |
| N(23)–Fe(2)–N(64) | 135.63(9) | 134.54(9) |
| N(23)–Fe(2)–N(71) | 84.68(9) | 85.28(9) |
| N(28)–Fe(2)–N(35) | 71.31(9) | 71.70(9) |
| N(28)–Fe(2)–N(59) | 94.57(9) | 97.02(8) |
| N(28)–Fe(2)–N(64) | 151.50(9) | 153.60(9) |
| N(28)–Fe(2)–N(71) | 124.89(9) | 119.88(9) |
| N(35)–Fe(2)–N(59) | 84.51(9) | 84.14(9) |
| N(35)–Fe(2)–N(64) | 82.47(9) | 83.22(9) |
| N(35)–Fe(2)–N(71) | 106.08(9) | 102.12(9) |
| N(59)–Fe(2)–N(64) | 71.45(9) | 71.76(9) |
| N(59)–Fe(2)–N(71) | 140.54(9) | 142.81(9) |
| N(64)–Fe(2)–N(71) | 72.47(9) | 72.67(9) |
| | | |
| $V_{Oh}$ {Fe(1), Fe(2)} | 11.912(9), 11.496(9) | 11.642(9), 11.658(9) |
| $\Sigma$ {Fe(1), Fe(2)} | 225.4(3), 213.0(3) | 216.7(3), 205.6(3) |
| $\Theta$ {Fe(1), Fe(2)} | 466, 459 | 464, 584 |
| $\varphi$ {Fe(1), Fe(2)} | 148.68(10), 151.50(9) | 148.18(9), 153.60(9) |
| $\theta$ {Fe(1), Fe(2)} | 52.71(5), 56.30(4) | 50.96(3), 61.08(2) |

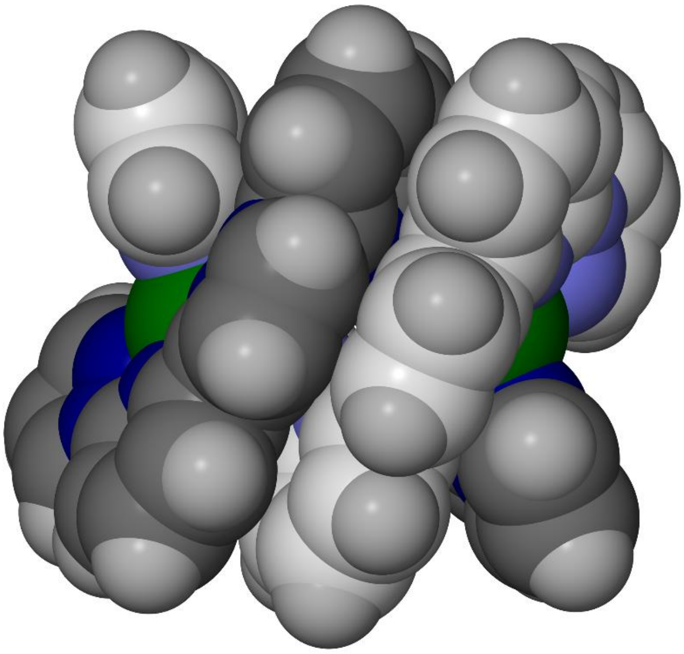

**Figure S29** Space-filling view of the $[Fe_2(\mu\text{-}L^2)_2]^{4+}$ cation in **2[ClO$_4$]$_4$**, oriented perpendicular to the Fe···Fe vector.

The two $L^2$ ligands in each molecule are distinguished with pale and dark coloration. Color code: C, white or gray; N, pale or dark blue; Fe, green.

**Table S6** Intramolecular and intermolecular $\pi\cdots\pi$ interactions in the crystal structures of **2[ClO$_4$]$_4$**. See Figure S26 for the atom numbering scheme, while the letters labelling each interaction correspond to those in Figure S30. Symmetry codes: (i) 1–*x*, 1–*y*, 1–*z*; (ii) 1–*x*, 1–*y*, –*z*.

| | Dihedral angle [deg] | Average interplanar spacing [Å] | Horizontal offset [Å] |
|---|---|---|---|
| **2[ClO$_4$]$_4$** | | | |
| [N(19)-C(24)]···[N(41)-C(46)] (**i**) | 5.9(2) | 3.465(9) | 0.84 |
| [N(30)-C(35)]···[N(57)-C(62)] (**ii**) | 7.76(16) | 3.609(10) | 1.05 |
| [N(3)-C(8), C(14)-C(18)]···[N(63)-C(73)] (**iii**) | 5.01(17) | 3.412(12) | 1.39 |
| [N(41)-C(51)]···[N(41$^{i}$)-C(51$^{i}$)] (**iv**) | 0 | 3.343(14) | 1.49 |
| | | | |
| **2[ClO$_4$]$_4$**·3MeNO$_2$·0.75H$_2$O | | | |
| [N(19)-C(24)]···[N(41)-C(46)] (**i**) | 9.73(18) | 3.570(10) | 1.17 |
| [N(30)-C(35)]···[N(57)-C(62)] (**ii**) | 9.97(12) | 3.704(9) | 1.16 |
| [N(3)-C(8), C(14)-C(18)]···[N(63)-C(73)] (**iii**) | 2.47(8) | 3.402(11) | 1.43 |
| [N(41)-C(51)]···[N(41$^{ii}$)-C(51$^{ii}$)] (**iv**) | 0 | 3.504(16) | 0.96 |

The Pauling van der Waals radius of an aromatic ring is 1.7 Å,[14] so only $\pi\cdots\pi$ contacts with an interplanar spacing ≤3.4 Å can be considered a strong stacking interaction. Longer interactions in the Table represent weaker van der Waals contacts between atoms in the neighboring groups.

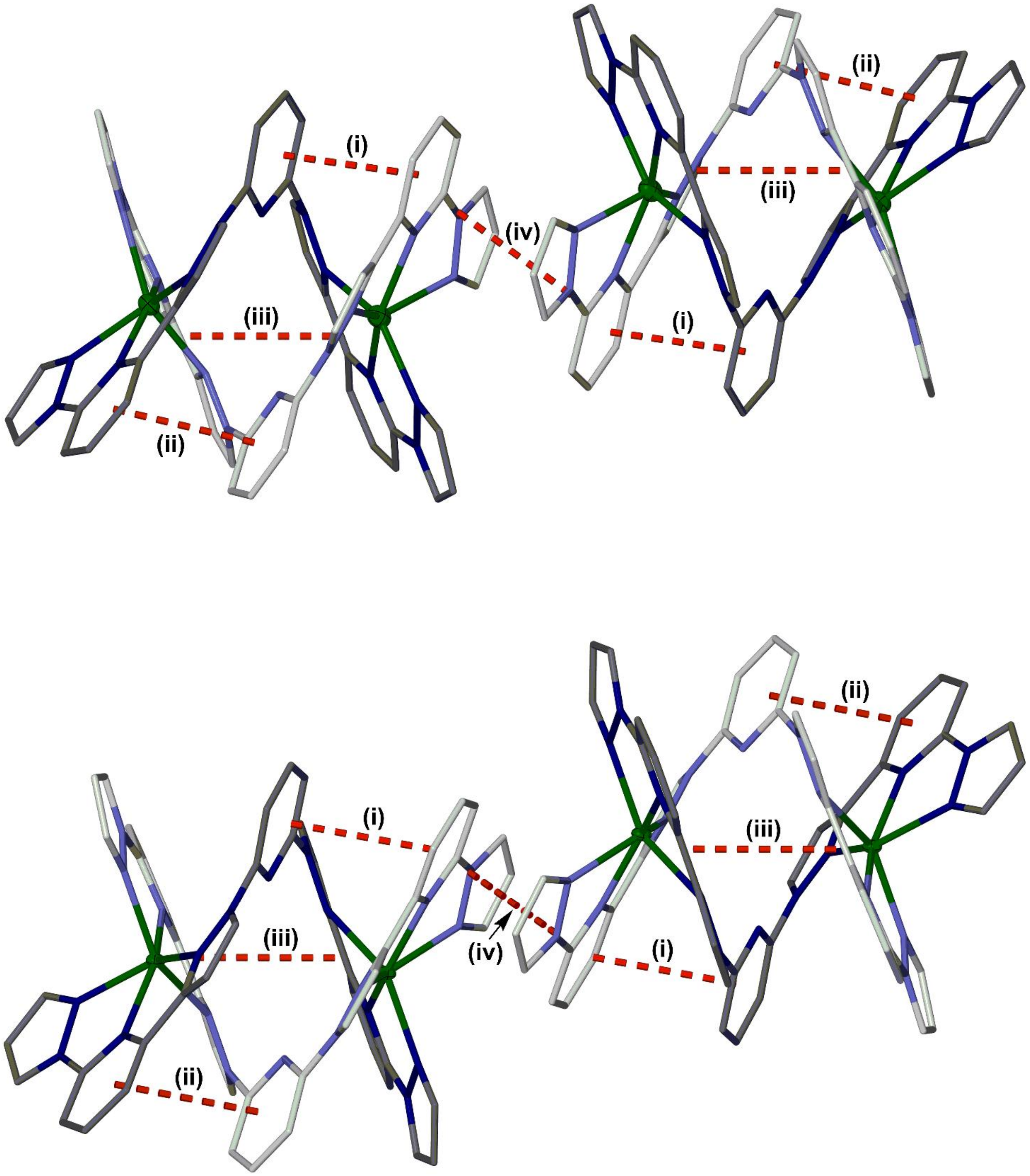


**Figure S30** Intramolecular and intermolecular $\pi\cdots\pi$ contacts in the centrosymmetric dimers of $[Fe_2(\mu\text{-}L^2)_2]^{4+}$ molecules in the crystal structures of **2[ClO₄]₄** (top) and **2[ClO₄]₄**·3MeNO₂·0.75H₂O (bottom). H atoms are omitted for clarity.

Color code: C, white or gray; N, pale or dark blue; Fe, green.

The label for each interaction refers to the corresponding entry in Table S6.

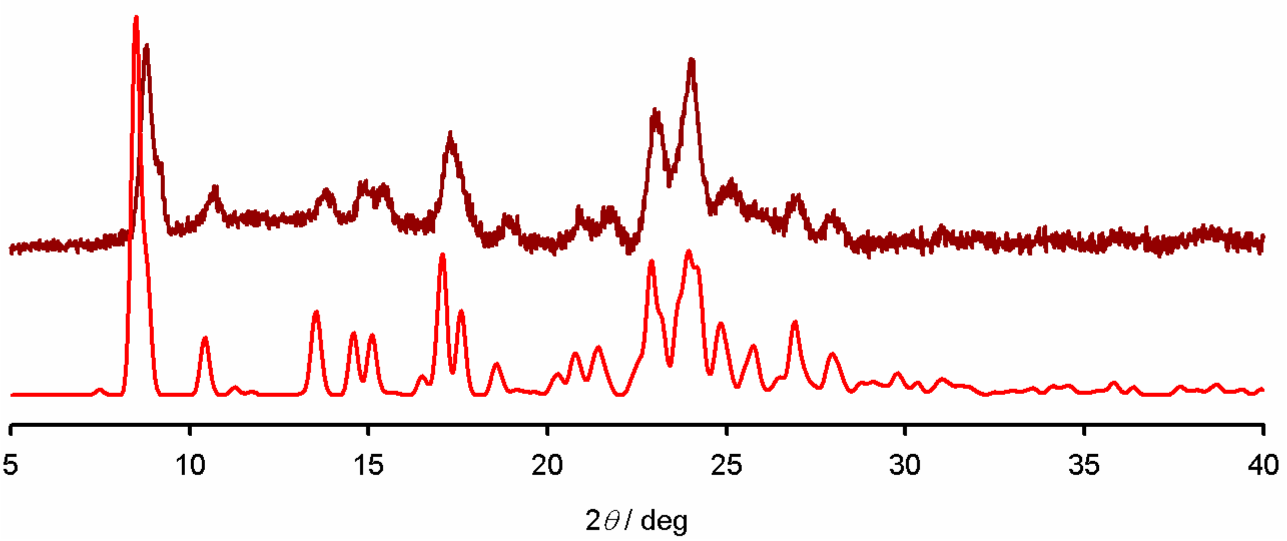


**Figure S31** Room temperature X-ray powder diffraction pattern for **2[ClO$_4$]$_4$**, crystallized from acetone solution using diethyl ether antisolvent (dark red), and a crystallographic simulation of the solvent-free **2[ClO$_4$]$_4$** phase (pale red).

Although the sample is only moderately crystalline, the agreement with the crystallographic simulation is good. Small differences in the peak positions might reflect the different temperatures of the measurement (298 K) and simulation (120 K).

A powder diffraction pattern of **2[BF$_4$]$_4$** was not measured during this study.

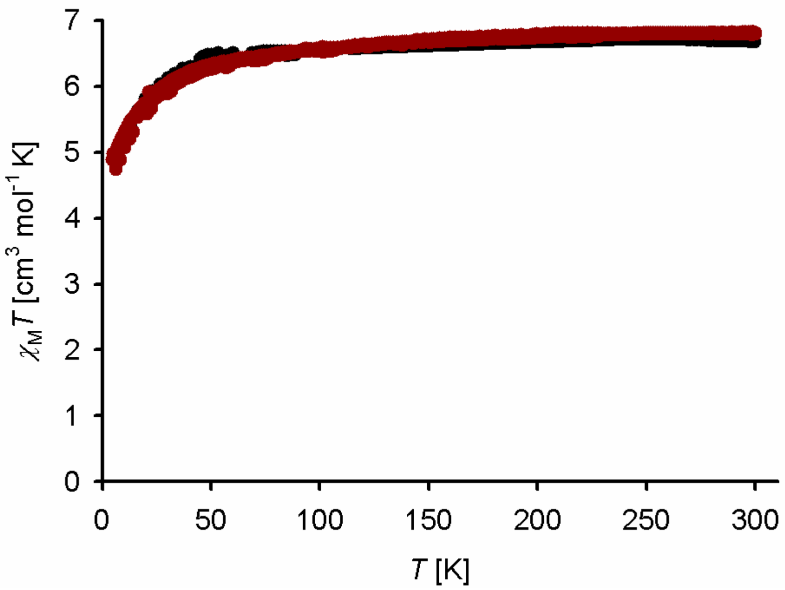


**Figure S32** Variable temperature magnetic susceptibility data for **2[BF$_4$]$_4$** (black) and **2[ClO$_4$]$_4$** (red). Scan rate 5 K min$^{-1}$.

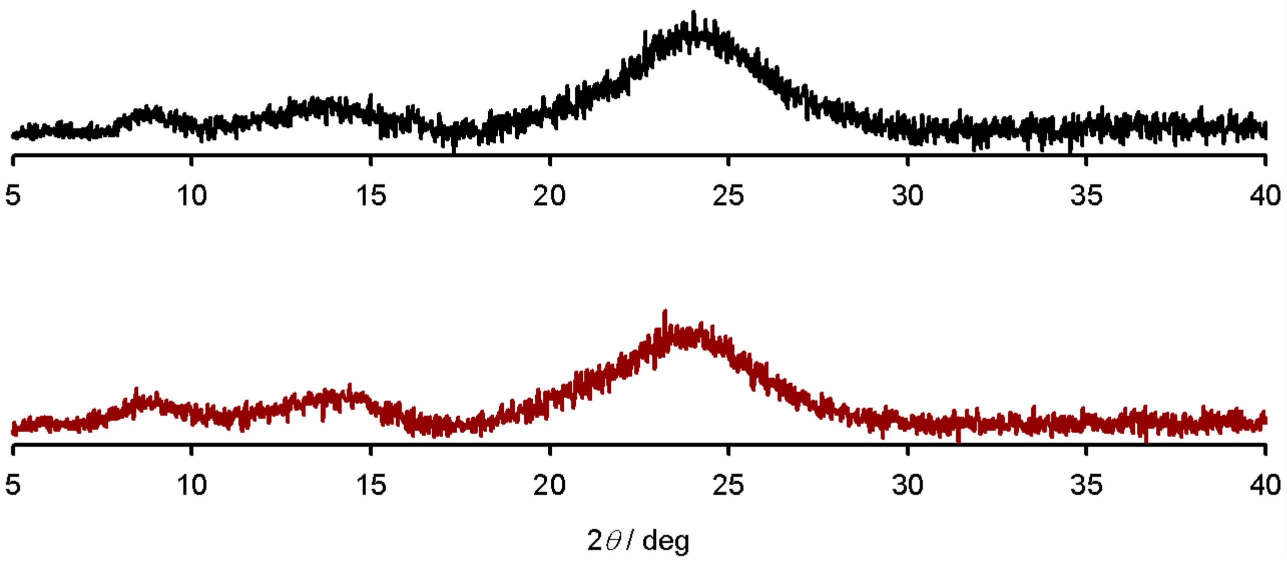


**Figure S33** Room temperature X-ray powder diffraction data for air-dried samples of **3[BF$_4$]$_2$**·$CH_3NO_2$ (black) and **3[ClO$_4$]$_2$**·1.5$H_2O$ (red).

These materials were crystallized from nitromethane using diethyl ether as antisolvent. Samples recrystallized from acetonitrile/diethyl ether gave almost identical powder patterns to those shown here.

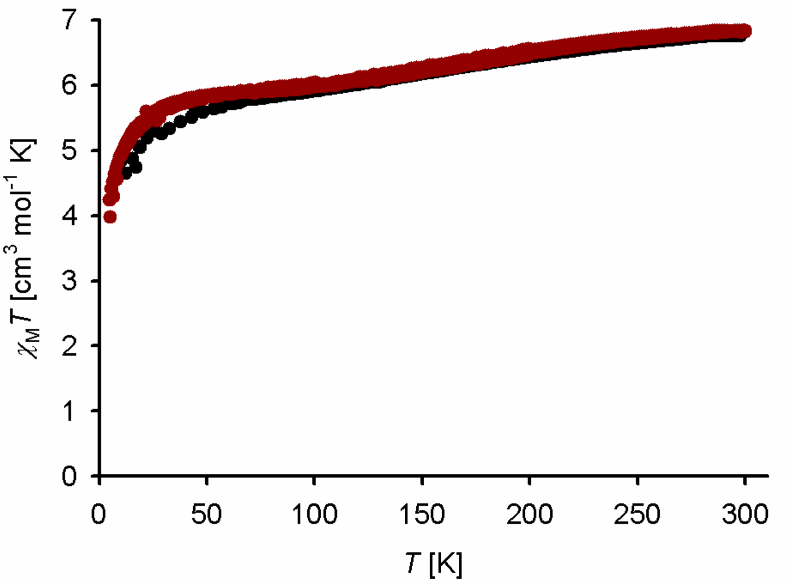


**Figure S34** Variable temperature magnetic susceptibility data for air-dried samples of **3[BF$_4$]$_2$**·$CH_3NO_2$ (black) and **3[ClO$_4$]$_2$**·1.5$H_2O$ (red).

The very gradual, ill-defined SCO in these solids is consistent with their amorphous nature (Figure S33), which implies they contain mixtures of iron switching centers in heterogeneous environments.

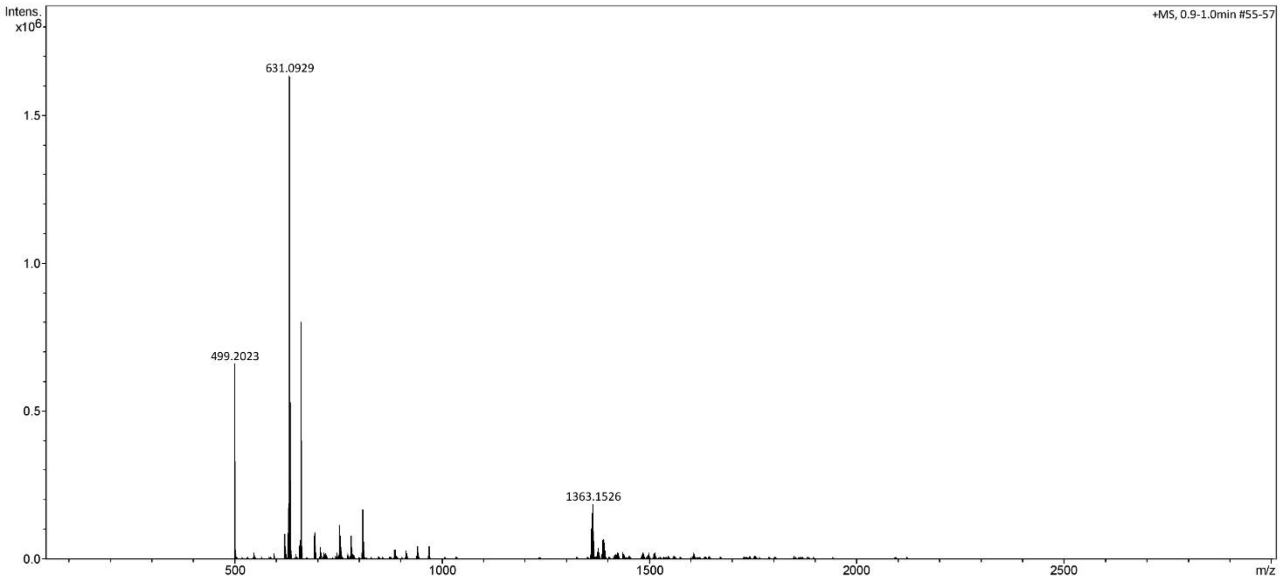


**Figure S35** Electrospray mass spectrum of **1[ClO$_4$]$_4$** from MeCN solution.

Figure 5 of the main article shows an expansion and simulation of the *m*/*z* = 1363.1526 peak, which is a combination of $[Fe_2(L^1)_2(ClO_4)_3]^+$, $[Fe_4(L^1)_4(ClO_4)_6]^{2+}$ and $[Fe_6(L^1)_6(ClO_4)_9]^{3+}$ in a 0.3:0.6:0.1 ratio. There is no evidence for lower charge states of the hexanuclear assembly in the spectrum.

This spectrum shows more fragmentation than for **2[ClO$_4$]$_4$** and **3[ClO$_4$]$_2$** (Figures S36 and S37), including uncoordinated $L^1$ as its sodium adduct (the sodium derives from the calibrant solution used in the spectrometer).That reflects the greater lability of this system in solution, which is in fast exchange by NMR (Figure S38). None-the-less, the presence of mono-, di-, tetra- and hexanuclear molecular ions is clear in the spectrum.

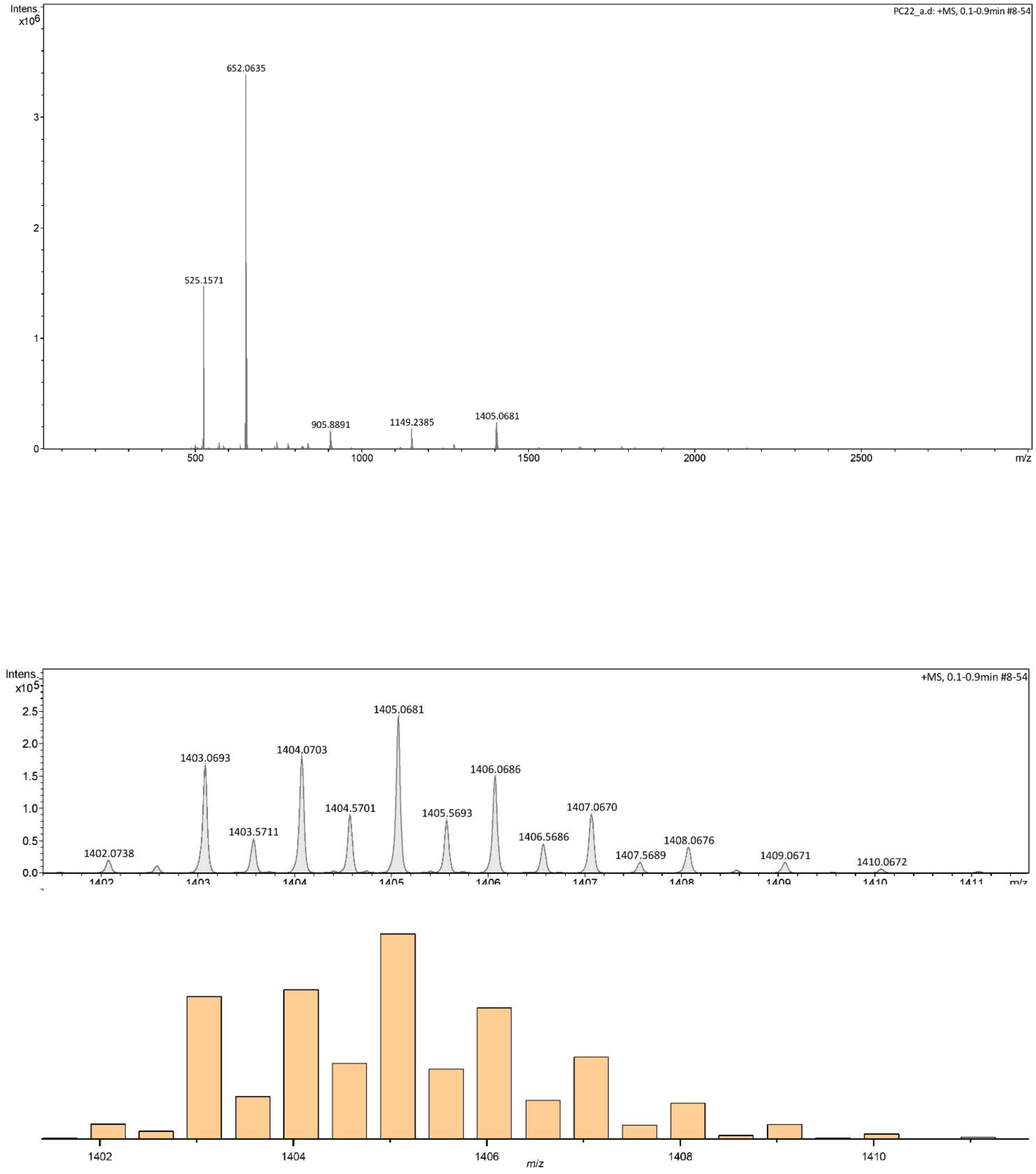


**Figure S36** Electrospray mass spectrum of **2[ClO$_4$]$_4$** from MeCN solution. Top: full spectrum. Bottom: expansion and simulation of the highest significant molecular ion.

The simulation is a combination of $[Fe_2(L^2)_2(ClO_4)_3]^+$ and $[Fe_4(L^2)_4(ClO_4)_6]^{2+}$, in a 0.6:0.4 ratio.

In contrast to **1[ClO$_4$]$_4$** and **3[ClO$_4$]$_2$**, there is no evidence for any larger assembly species in the spectrum.

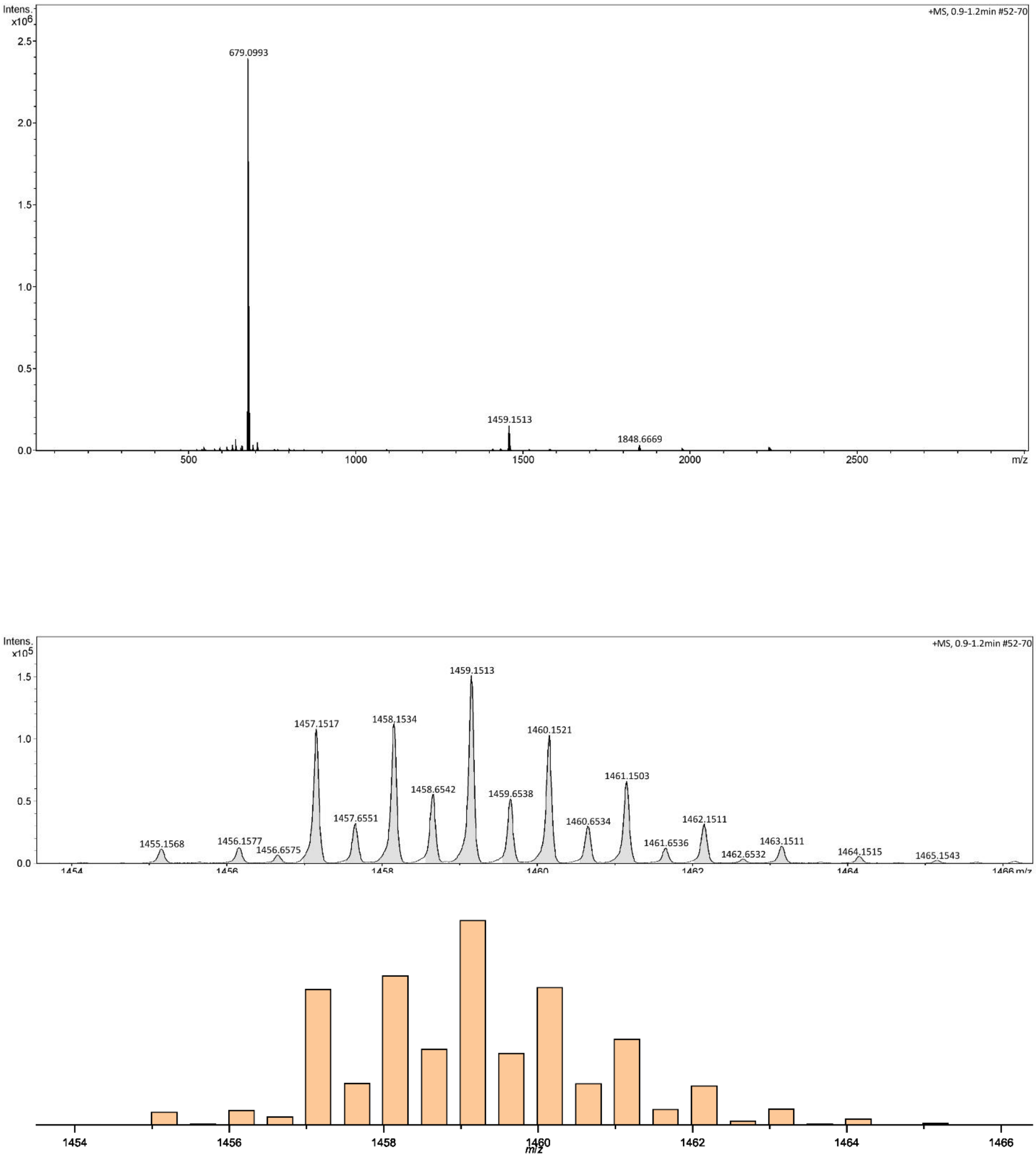


**Figure S37** Electrospray mass spectrum of **3[ClO$_4$]$_2$** from MeCN solution. Top: full spectrum. Bottom: expansion and simulation of the highest significant molecular ion.

The simulation is a combination of [Fe$_2$($L^3$)$_2$(ClO$_4$)$_3$]$^+$ and [Fe$_4$($L^3$)$_4$(ClO$_4$)$_6$]$^{2+}$, in a 0.6:0.4 ratio.

There are also weak higher mass peaks in the spectrum from [Fe$_5$($L^3$)$_5$(ClO$_4$)$_8$]$^{2+}$ ($m$/z = 1848.6669) and [Fe$_6$($L^3$)$_6$(ClO$_4$)$_{10}$]$^{2+}$ ($m$/z = 2238.1873).

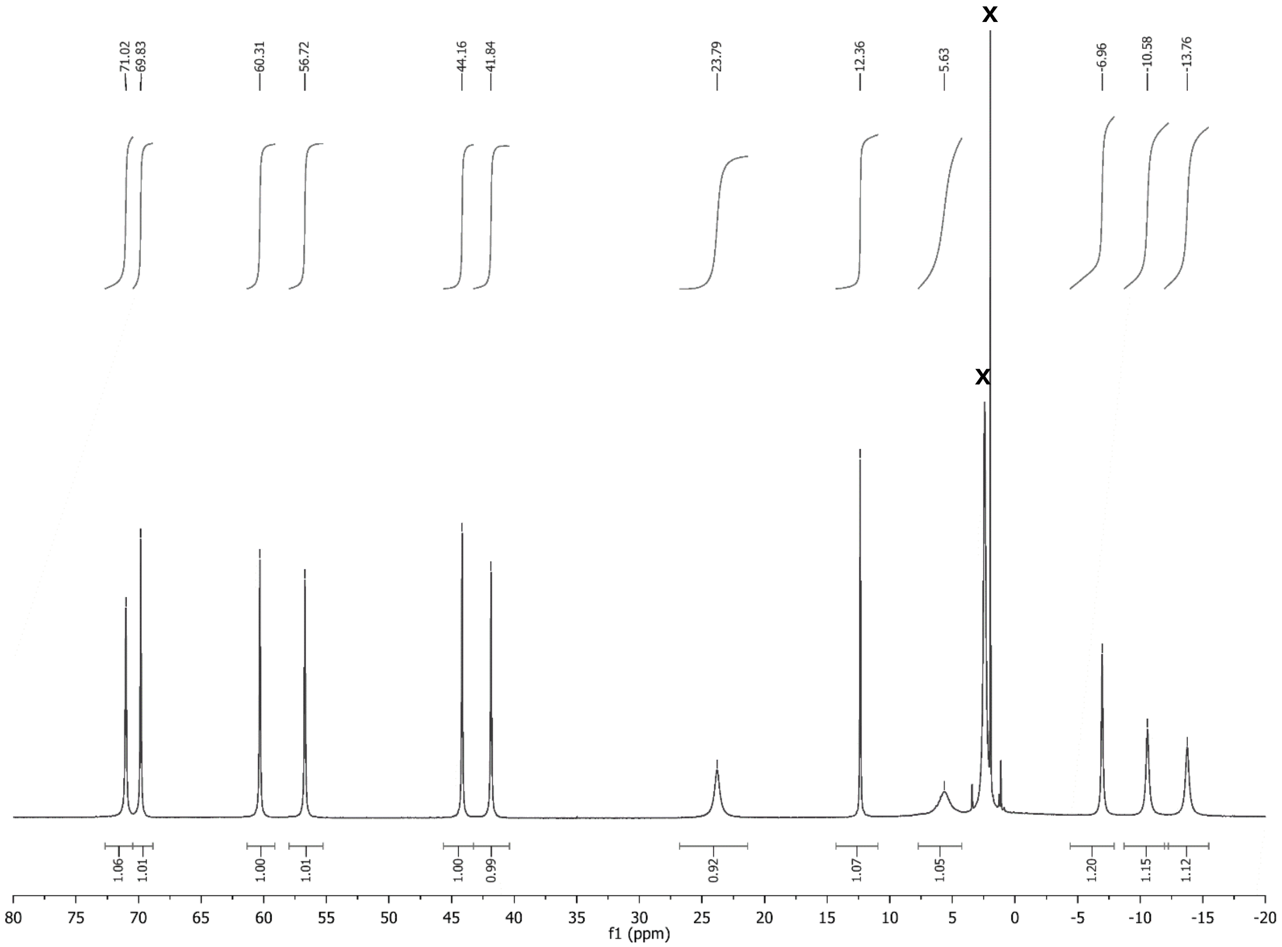


**Figure S38** $^1$H NMR spectra of **1[ClO$_4$]$_4$** ($CD_3CN$).

A $C_2$-symmetric $L^1$ environment with diastereotopic $CH_2$ groups should give twelve resonances of equal integral. These are all clearly resolved in the spectrum. The four upfield peaks at –13.8, –10.6, –7.0 and 5.6 ppm are broader than the others, and are assigned to the conformationally flexible butanediyl group resonances.

There are no peaks in the diamagnetic region corresponding to uncoordinated $L^1$, or to dangling 1,3-bpp residues in a monodentate $L^1$ complex.

The mass spectrum of **1[ClO$_4$]$_4$** contains mono-, di-, tetra- and hexa-nuclear assembly structures as well as some non-specific fragmentation (Figure S35). Since only one averaged ligand environment is present in this spectrum, those species must be in fast chemical exchange at room temperature.

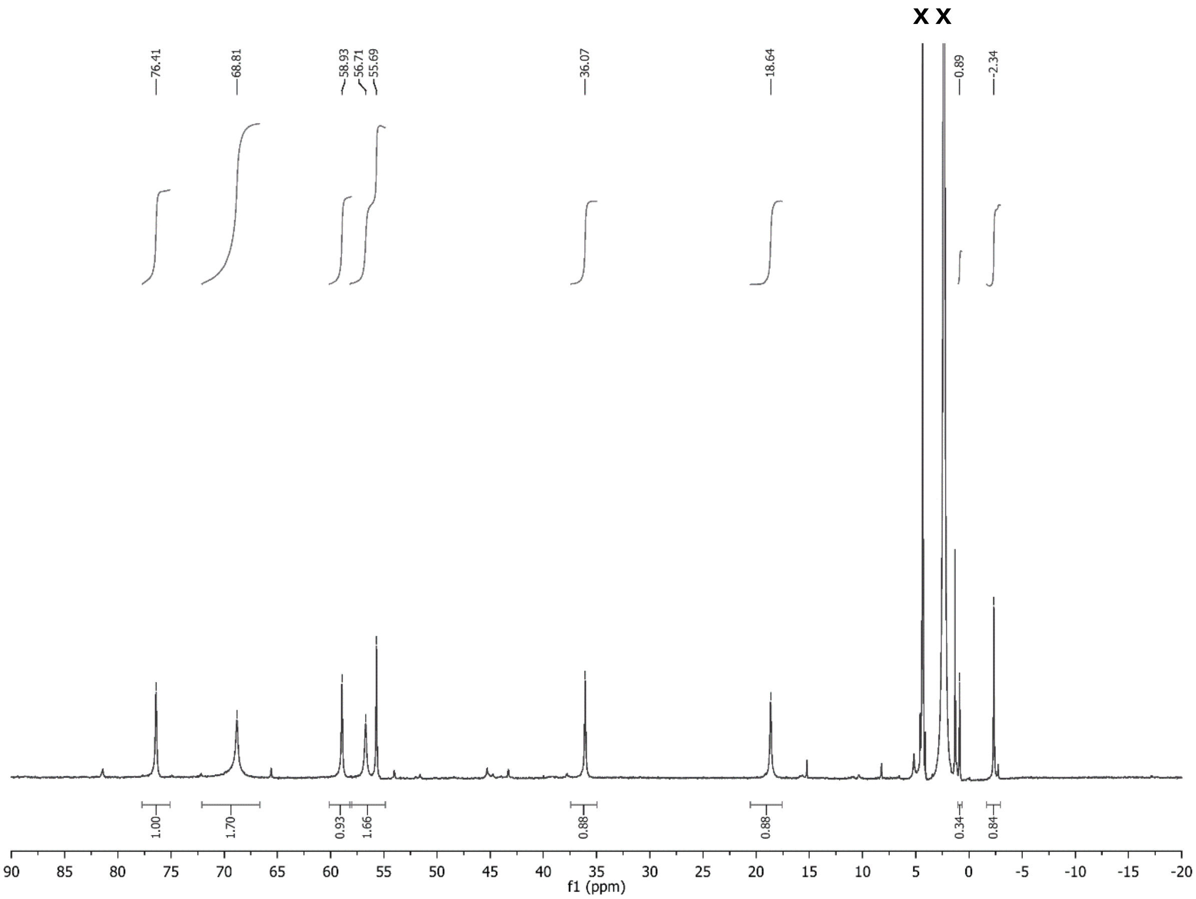


**Figure S39** $^{1}$H NMR spectrum of **2[ClO$_4$]$_4$** ($CD_3CN$).

Ten resonances are expected for a $C_2$-symmetric $L^2$ ligand with one peak, from the central pyridyl $H^4$ environment, having half the integral of the others. The main peaks in the spectrum correspond to that pattern.

The spectrum also contains at least one other, minor paramagnetic component, with 10-15 % integral compared to the main species. Mass spectrometry implies this could be a $[Fe_4(\mu\text{-}L^2)_4]^{8+}$ metallacycle (Figure S36). In contrast to **1[ClO$_4$]$_4$**, (Figure S38), these species are in slow exchange on the NMR timescale.

There are no peaks in the diamagnetic region corresponding to uncoordinated $L^2$, or to dangling 1,3-bpp residues in a monodentate $L^2$ complex.

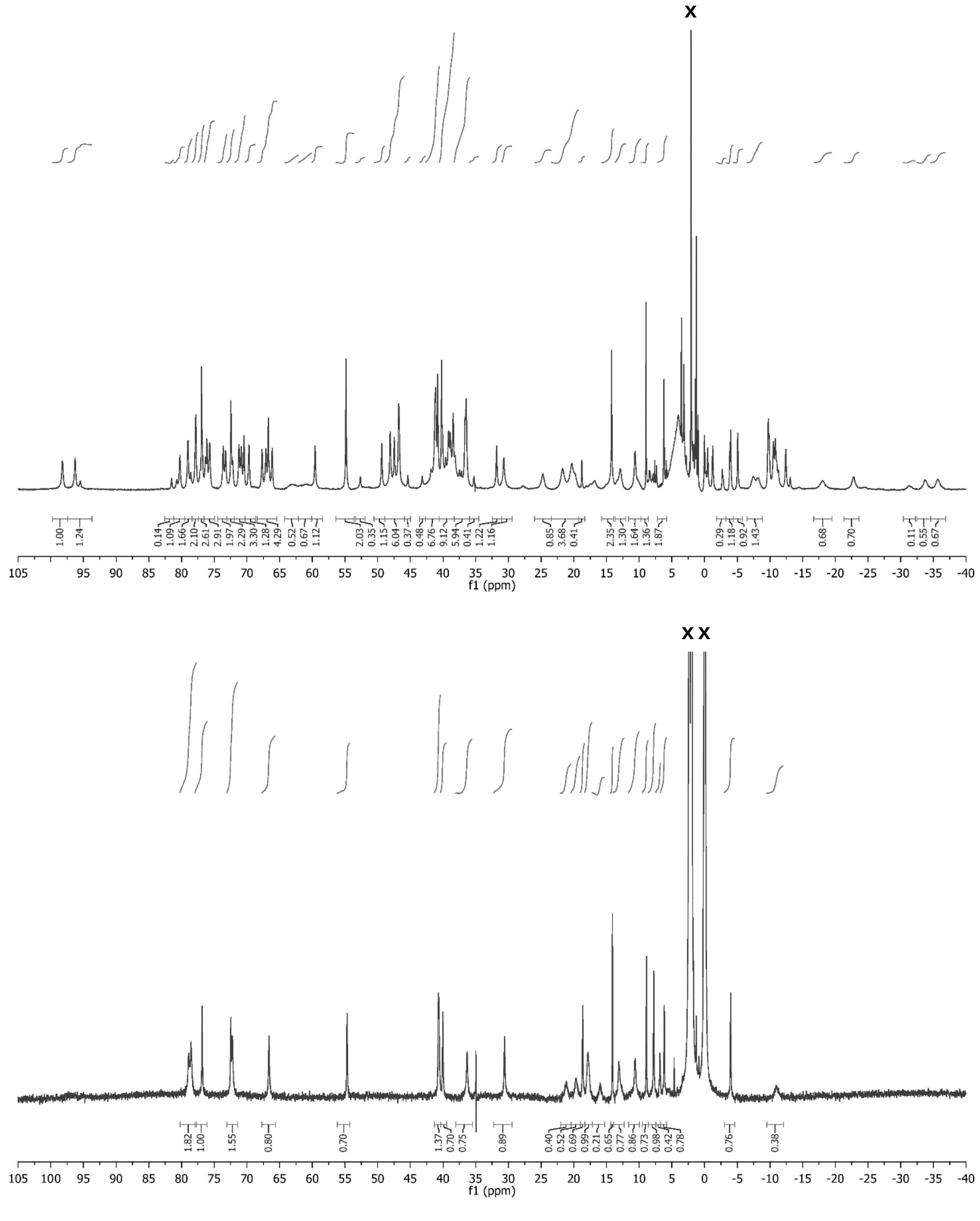


**Figure S40** $^{1}H$ NMR spectra of **3[ClO$_4$]$_2$** in $CD_3CN$ (top), and $\{CD_3\}_2CO$ (bottom). The feature in the bottom spectrum at 35 ppm is a spectrometer artefact.

A $C_2$-symmetric $L^3$ environment with diastereotopic $CH_2$ groups should give twelve unique resonances of equal integral. At least 25 peaks can be distinguished in the $\{CD_3\}_2CO$ spectrum, which are mostly grouped into three sets with an approximate integral ratio of 1:0.7:0.4. Hence, the solution appears to contain three different iron(II)/$L^3$ assembly components.

The $CD_3CN$ spectrum is more complicated, but at least one species is common to both spectra.

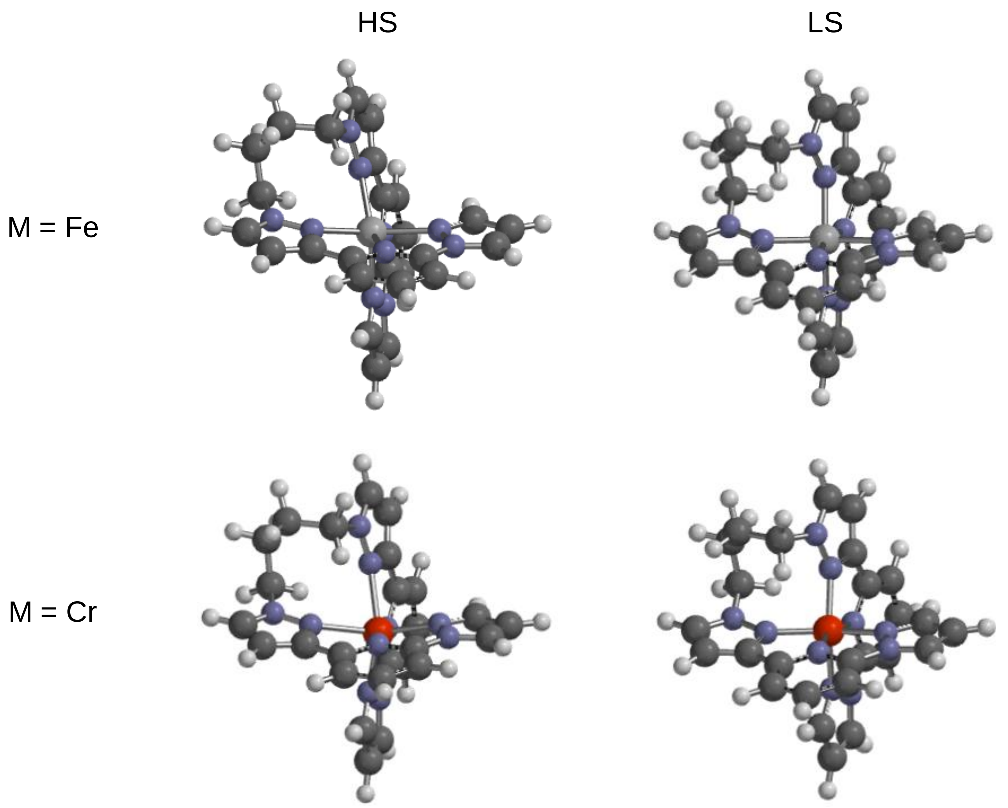


**Figure S41** Views of energy-minimized $[M(L)]^{z+}$ ($M^{z+} = Fe^{2+}$ or $Cr^0$) computed in their high-spin and low-spin states (HS = high-spin, LS = low-spin).

Color code: C, dark gray; H, white; Cr, orange; Fe, pale gray; N, blue.

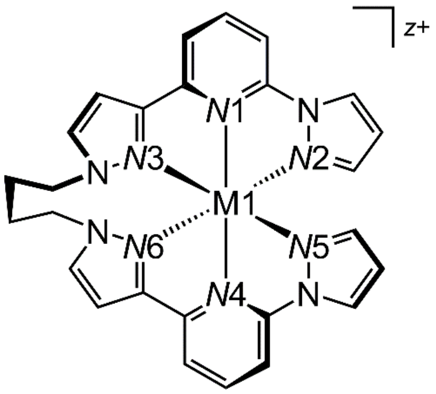


**Scheme S6** Atom numbering used in Table S7 ($M^{z+} = Fe^{2+}$ or $Cr^0$).

**Table S7** Computed metric parameters for $[ML^1]^{z+}$ ($M^{z+} = Fe^{2+}$ or $Cr^0$) [Å, deg]. See Scheme S6 for the atom numbering scheme.[a]

| | $M^{z+} = Fe^{2+}$ | | $M^{z+} = Cr^0$ | |
|---|---|---|---|---|
| | HS ($S$ = 2) | LS ($S$ = 0) | HS ($S$ = 2) | LS ($S$ = 0) |
| Fe1–*N*1 | 2.182 | 1.945 | 2.054 | 1.974 |
| Fe1–*N*2 | 2.213 | 1.978 | 2.102 | 2.013 |
| Fe1–*N*3 | 2.229 | 2.038 | 2.360 | 2.036 |
| Fe1–*N*4 | 2.182 | 1.945 | 2.054 | 1.974 |
| Fe1–*N*5 | 2.213 | 1.978 | 2.102 | 2.013 |
| Fe1–*N*6 | 2.229 | 2.038 | 2.360 | 2.036 |
| | | | | |
| *N*1–Fe1–*N*2 | 72.5 | 79.5 | 75.6 | 77.1 |
| *N*1–Fe1–*N*3 | 72.7 | 77.6 | 71.4 | 75.7 |
| *N*1–Fe1–*N*4 ($\phi$) | 152.9 | 165.0 | 170.2 | 161.2 |
| *N*1–Fe1–*N*5 | 88.5 | 90.2 | 98.7 | 90.0 |
| *N*1–Fe1–*N*6 | 128.9 | 113.4 | 116.4 | 118.0 |
| *N*2–Fe1–*N*3 | 142.5 | 156.3 | 141.4 | 152.0 |
| *N*2–Fe1–*N*4 | 88.5 | 90.2 | 98.7 | 90.0 |
| *N*2–Fe1–*N*5 | 92.2 | 94.1 | 110.8 | 93.7 |
| *N*2–Fe1–*N*6 | 100.6 | 92.2 | 93.7 | 92.9 |
| *N*3–Fe1–*N*4 | 128.9 | 113.4 | 116.4 | 118.0 |
| *N*3–Fe1–*N*5 | 100.6 | 92.2 | 93.7 | 92.9 |
| *N*3–Fe1–*N*6 | 90.4 | 91.1 | 83.6 | 93.9 |
| *N*4–Fe1–*N*5 | 72.5 | 79.5 | 75.6 | 77.1 |
| *N*4–Fe1–*N*6 | 72.7 | 77.6 | 71.4 | 75.7 |
| *N*5–Fe1–*N*6 | 142.5 | 156.3 | 141.4 | 152.0 |
| | | | | |
| $\alpha$ | 72.6 | 78.6 | 73.5 | 76.4 |
| $\theta$ | 74.4 | 86.3 | 82.0 | 83.2 |

[a]HS = high-spin, LS = low-spin. $\alpha$ is the average ligand bite angle in the molecule; see page S20 for definitions of $\phi$ and $\theta$.

**Table S8** Computed minimized energies of $[ML^1]^{z+}$ ($M^{z+} = Fe^{2+}$ or $Cr^0$). The energy difference between mononuclear $[ML^1]^{z+}$ and helicate $[M_2(\mu\text{-}L^1)_2]^{2z+}$, $\Delta E_{monomer/dimer}$, is also presented.[a]

| | $[ML^1]^{z+}$ $E$\{HS\} [Ha] | $[ML^1]^{z+}$ $E$\{LS\} [Ha] | $\Delta E_{monomer/dimer}$\{HS\} [kcal mol$^{-1}$][b] | $\Delta E_{monomer/dimer}$\{LS\} [kcal mol$^{-1}$][b] |
|---|---|---|---|---|
| M = Fe | –2815.484288 | –2815.490595 | +115.2 | +100.1 |
| M = Cr | –2596.626320 | –2596.668539 | – | –26.9 |

[a]HS = high-spin, LS = low-spin. [b]$\Delta E_{monomer/dimer} = E\{[M_2(\mu\text{-}L^1)_2]^{2z+}\}–2E\{[M(L^1)]^{z+}\}$, per mole of helicate molecules.

$\Delta E_{monomer/dimer}$ is calculated using the energies for conformation (a) of $[M_2(\mu\text{-}L^1)_2]^{2z+}$, taken from Table S9. $\Delta E_{monomer/dimer}$ for conformations (b) and (c) also lie within 2 kcal mol$^{-1}$ of these values.

A positive $\Delta E_{monomer/dimer}$ means the monomeric complex is lower energy than the helicate molecule by this protocol. By this measure, $[Fe_2(\mu\text{-}L^1)_2]^{4+}$ should not exist but $[Cr_2(\mu\text{-}L^1)_2]^0$ is the more stable molecule, under these conditions. $\Delta E_{monomer/dimer}$ for the high-spin chromium complex is not considered, for the reasons discussed in the main article.

Since $[Fe_2(\mu\text{-}L^1)_2]^{4+}$ is stable under ambient conditions, $\Delta E_{monomer/dimer}$ for M = Fe is anomalous. That may reflect destabilization of the dimer by electrostatic repulsion between its positively charged $Fe^{2+}$ ions in the gas phase, which is not a factor for electroneutral $[Cr_2(\mu\text{-}L^1)_2]^0$. This is discussed further in the main article.

**Table S9** Minimized energies of the high-spin (HS, $S$ = 4), mixed-spin (MS, $S$ = 2) and low-spin (LS, $S$ = 0) states of all the complexes computed in this work. Data from Table S8, and Table 3 of the main article, are repeated here for comparison.

| | $E$(HS) [Ha] | $E$(MS) [Ha] | $E$(LS) [Ha] | $\Delta E$\{HS–LS\} [kcal mol$^{-1}$] | $\Delta E_{rel}$\{HS–LS\} [kcal mol$^{-1}$][a] | $\Delta E$\{HS–LS, MS\} [kcal mol$^{-1}$][b] |
|---|---|---|---|---|---|---|
| $[FeL^1]^{2+}$ | –2815.484288 | – | –2815.490595 | +4.0 | – | – |
| $[Fe_2(\mu\text{-}L^1)_2]^{4+}$, conformation (a) | –5630.785068 | –5630.802870 | –5630.820271 | +22.1 | 0 | +0.1 |
| $[Fe_2(\mu\text{-}L^1)_2]^{4+}$, conformation (b) | –5630.786168 | –5630.802732 | –5630.819907 | +21.2 | –0.9 | –0.2 |
| $[Fe_2(\mu\text{-}L^1)_2]^{4+}$, conformation (c) | –5630.788009 | –5630.802622 | –5630.818614 | +19.2 | –2.9 | –0.4 |
| $[Fe_2(\mu\text{-}L^2)_2]^{4+}$ | –5810.391891 | –5810.402230 | –5810.409082 | +10.8 | –11.3 | +1.1 |
| $[Fe_2(\mu\text{-}L^3)_2]^{4+}$ | –5935.545352 | –5935.560026 | –5935.572551 | +17.1 | –5.0 | +0.7 |
| | | | | | | |
| $[CrL^1]^0$ | –2596.626320 | – | –2596.668539 | +26.5 | – | – |
| $[Cr_2(\mu\text{-}L^1)_2]^0$, conformation (a) | –5193.299195 | –5193.339800 | –5193.380018 | +50.7 | 0 | +0.1 |
| $[Cr_2(\mu\text{-}L^1)_2]^0$, conformation (b) | –5193.301449 | –5193.340148 | –5193.380851 | +49.8 | –0.9 | –0.6 |
| $[Cr_2(\mu\text{-}L^1)_2]^0$, conformation (c) | –5193.301617 | –5193.342532 | –5193.379687 | +49.0 | –1.7 | +1.2 |
| $[Cr_2(\mu\text{-}L^2)_2]^0$ | –5372.929391 | –5372.960542 | –5372.984020 | +34.3 | –16.4 | –2.4 |
| $[Cr_2(\mu\text{-}L^3)_2]^0$ | –5498.054423 | –5498.054423 | –5498.133476 | +49.6 | –1.1 | 0.0 |

[a] A positive $\Delta E_{rel}$\{HS−LS\} means the low-spin state is more stable than for conformation (a) of $[M_2(\mu\text{-}L^1)_2]^{2z+}$ ($M^{z+}$ = $Fe^{2+}$ or $Cr^0$), and *vice versa*. [b] A positive $\Delta E$\{HS–LS, MS\} means the mixed-spin state is more stable than an equimolar mixture of high-spin and low-spin molecules, and *vice versa*.

$\Delta E_{rel}$\{HS–LS\} for mononuclear $[FeL^1]^{2+}$ and $[CrL^1]^0$ are not included in the Table. While $\Delta E$\{HS–LS\} for $[CrL^1]^0$ is approximately half that of the dinuclear chromium complexes, as expected, it seems clear that $\Delta E$\{HS–LS\} for $[FeL^1]^{2+}$ and the di-iron complexes cannot be directly compared. That might be a consequence of electrostatic repulsion between the iron atoms in the diiron helicate molecules, as discussed in the main article.

$\Delta E_{rel}$\{HS–LS\} for the chromium complexes mirrors the corresponding iron complexes, except perhaps for $[Cr_2(\mu\text{-}L^3)_2]^0$ whose minimized conformation is quite different from $[Fe_2(\mu\text{-}L^3)_2]^{4+}$ (Figure S48). The significance of that observation is uncertain however, because of the different electronic structures of the high-spin chromium and iron centers in these calculations. This is discussed in the main article, and on page S58 below.

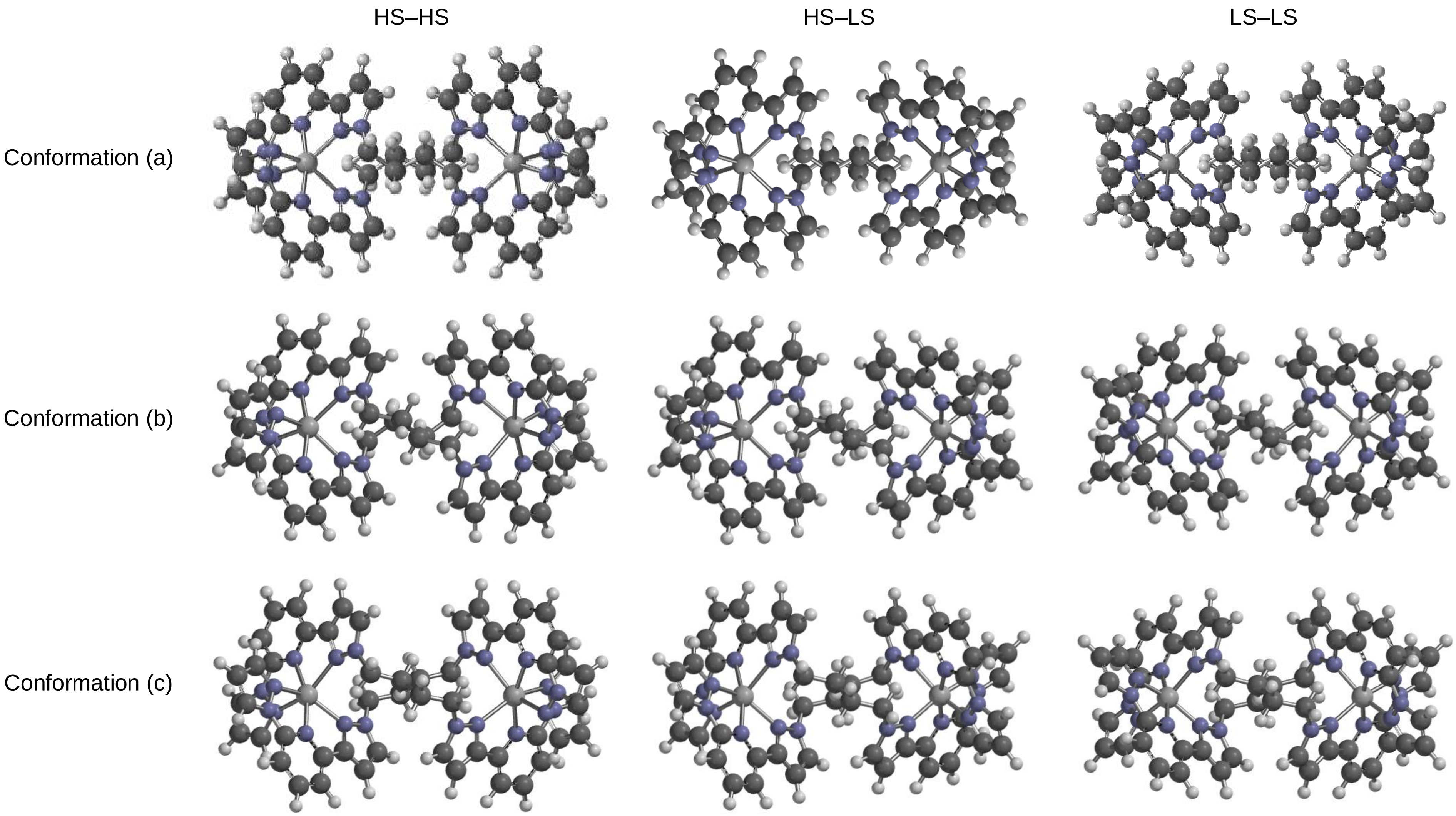


**Figure S42** Views of the three conformations of $[Fe_2(\mu\text{-}L^1)_2]^{4+}$, minimized in their high-spin, mixed-spin and low-spin states (HS = high-spin, LS = low-spin). Analogous views of the crystallographic structures are in Figure 1, main article. Color code: C, dark gray; H, white; Fe, pale gray; N, blue.

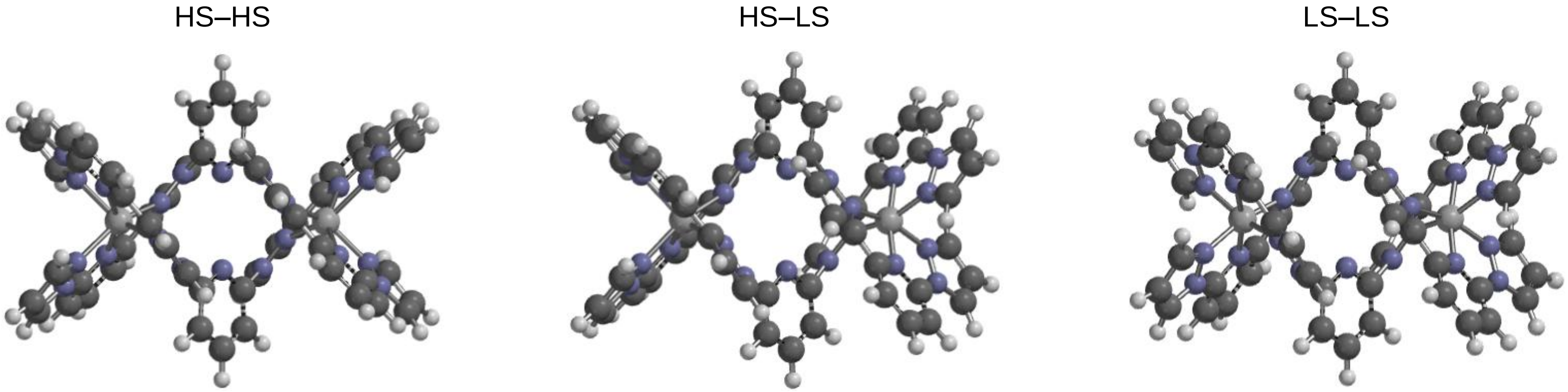


**Figure S43** Views of energy-minimized $[Fe_2(\mu\text{-}L^2)_2]^{4+}$, minimized in its high-spin, mixed-spin and low-spin states. Details as for Figure S42. A view of the crystallographic structure is in Figure 3, main article.

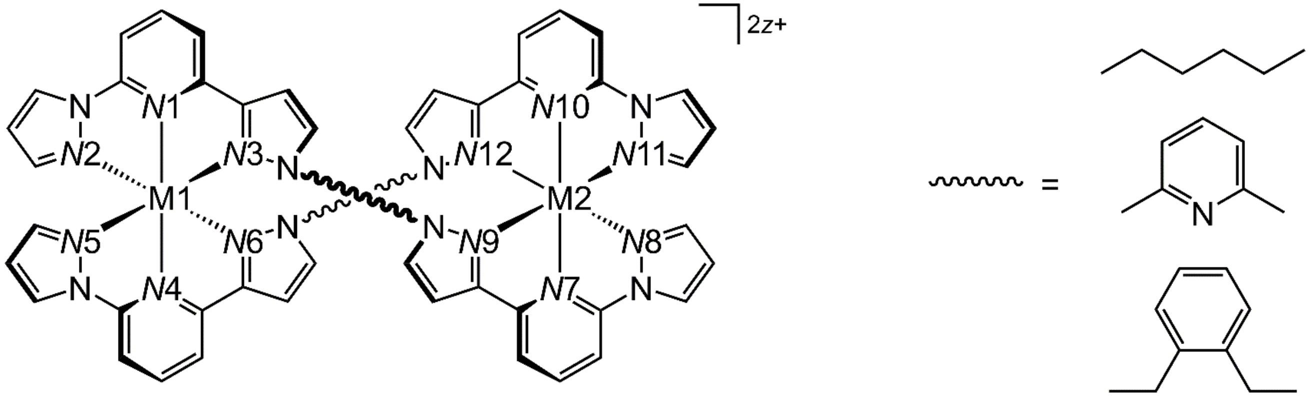


**Scheme S7** Atom numbering used in the Tables of metric parameters from the DFT minimizations for $[M_2(\mu\text{-}L^1)_2]^{2z+}$, $[M_2(\mu\text{-}L^2)_2]^{2z+}$ and $[M_2(\mu\text{-}L^3)_2]^{2z+}$ ($M^{z+} = Fe^{2+}$ or $Cr^0$). This scheme is different from the crystallographic atom numbering in this study.

**Table S10** Computed metric parameters for the different helicate conformations of $[Fe_2(\mu\text{-}L^1)_2]^{4+}$ [Å, deg]. See Scheme S7 for the atom numbering scheme in the Table. Where available, crystallographic values are also given in square brackets for comparison.[a]

| | $[Fe_2(\mu\text{-}L^1)_2]^{4+}$ – conformation (a) | | |
|---|---|---|---|
| | HS–HS ($S$ = 4) | HS–LS ($S$ = 2) | LS–LS ($S$ = 0) |
| Fe1–*N*1 | 2.149 | 2.149 | 1.927 [1.917(4)] |
| Fe1–*N*2 | 2.205 | 2.206 | 1.974 [1.962(4)] |
| Fe1–*N*3 | 2.258 | 2.258 | 2.040 [1.999(4)] |
| Fe1–*N*4 | 2.149 | 2.149 | 1.928 [1.917(4)] |
| Fe1–*N*5 | 2.205 | 2.206 | 1.975 [1.962(4)] |
| Fe1–*N*6 | 2.258 | 2.258 | 2.040 [1.999(4)] |
| Fe2–*N*7 | 2.152 | 1.931 | 1.933 [1.906(4)] |
| Fe2–*N*8 | 2.198 | 1.976 | 1.975 [1.965(3)] |
| Fe2–*N*9 | 2.262 | 2.036 | 2.043 [2.001(4)] |
| Fe2–*N*10 | 2.152 | 1.931 | 1.934 [1.906(4)] |
| Fe2–*N*11 | 2.197 | 1.976 | 1.975 [1.965(3)] |
| Fe2–*N*12 | 2.262 | 2.036 | 2.043 [2.001(4)] |
| Fe1···Fe2 | 9.411 | 9.191 | 8.983 [8.5452(11)] |
| | | | |
| *N*1–Fe1–*N*2 | 73.4 | 73.5 | 79.9 [80.24(15)] |
| *N*1–Fe1–*N*3 | 74.3 | 74.4 | 79.6 [79.40(15)] |
| *N*1–Fe1–*N*4 ($\phi$ {Fe1}) | 165.7 | 166.0 | 170.7 [171.8(2)] |
| *N*1–Fe1–*N*5 | 96.5 | 96.7 | 93.8 [94.09(15)] |
| *N*1–Fe1–*N*6 | 116.3 | 115.9 | 106.8 [106.39(15)] |
| *N*2–Fe1–*N*3 | 147.2 | 147.4 | 159.4 [159.52(15)] |
| *N*2–Fe1–*N*4 | 96.5 | 96.8 | 93.5 [94.09(15)] |
| *N*2–Fe1–*N*5 | 92.7 | 92.8 | 91.4 [93.1(2)] |
| *N*2–Fe1–*N*6 | 96.6 | 96.1 | 91.4 [90.58(14)] |
| *N*3–Fe1–*N*4 | 116.4 | 115.8 | 107.1 [106.39(15)] |
| *N*3–Fe1–*N*5 | 96.6 | 96.1 | 91.4 [90.58(14)] |
| *N*3–Fe1–*N*6 | 92.4 | 93.2 | 93.2 [93.0(2)] |
| *N*4–Fe1–*N*5 | 73.4 | 73.5 | 79.9 [80.24(15)] |
| *N*4–Fe1–*N*6 | 74.3 | 74.4 | 79.6 [79.40(15)] |
| *N*5–Fe1–*N*6 | 147.2 | 147.4 | 159.4 [159.52(15)] |
| *N*7–Fe2–*N*8 | 73.6 | 79.8 | 79.8 [80.29(15)] |
| *N*7–Fe2–*N*9 | 74.0 | 79.4 | 79.2 [79.65(16)] |
| *N*7–Fe2–*N*10 ($\phi$ {Fe2}) | 167.7 | 170.7 | 170.3 [174.1(2)] |
| *N*7–Fe2–*N*11 | 97.6 | 93.7 | 93.4 [95.69(15)] |
| *N*7–Fe2–*N*12 | 115.1 | 107.2 | 107.6 [104.51(16)] |
| *N*8–Fe2–*N*9 | 147.3 | 159.0 | 159.0 [159.78(15)] |
| *N*8–Fe2–*N*10 | 97.6 | 93.7 | 93.3 [95.69(15)] |
| *N*8–Fe2–*N*11 | 92.2 | 91.1 | 91.3 [94.5(2)] |
| *N*8–Fe2–*N*12 | 97.0 | 91.7 | 91.3 [89.98(14)] |
| *N*9–Fe2–*N*10 | 115.1 | 107.2 | 107.7 [104.51(16)] |
| *N*9–Fe2–*N*11 | 97.0 | 91.7 | 91.3 [89.98(14)] |
| *N*9–Fe2–*N*12 | 92.0 | 93.0 | 93.7 [92.6(2)] |
| *N*10–Fe2–*N*11 | 73.6 | 79.8 | 79.8 [80.29(15)] |
| *N*10–Fe2–*N*12 | 74.0 | 79.4 | 79.2 [79.65(16)] |
| *N*11–Fe2–*N*12 | 147.3 | 159.1 | 159.0 [159.78(15)] |
| | | | |
| $\alpha$ {Fe1} | 73.9 | 74.0 | 79.8 [79.8(2)] |
| $\alpha$ {Fe2} | 73.8 | 79.6 | 79.5 [80.0(2)] |
| $\theta$ {Fe1} | 85.9 | 86.3 | 89.1 [87.66(3)] |
| $\theta$ {Fe2} | 86.0 | 89.9 | 89.0 [84.28(3)] |

[a]HS = high-spin, LS = low-spin. $\alpha$ is the average ligand bite angle in the molecule; see page S20 for definitions of $\phi$ and $\theta$.

**Table S10 (continued).**[a]

| | $[Fe_2(\mu\text{-}L^1)_2]^{4+}$ – conformation (b) | | |
|---|---|---|---|
| | HS–HS ($S$ = 4) | HS–LS ($S$ = 2) | LS–LS ($S$ = 0) |
| Fe1–*N*1 | 2.154 [2.116(3)] | 2.149 | 1.931 |
| Fe1–*N*2 | 2.196 [2.171(3)] | 2.203 | 1.974 |
| Fe1–*N*3 | 2.249 [2.177(3)] | 2.274 | 2.039 |
| Fe1–*N*4 | 2.153 [2.106(3)] | 2.152 | 1.932 |
| Fe1–*N*5 | 2.205 [2.173(3)] | 2.195 | 1.972 |
| Fe1–*N*6 | 2.264 [2.211(3)] | 2.260 | 2.050 |
| Fe2–*N*7 | 2.148 [2.103(3)] | 1.931 | 1.933 |
| Fe2–*N*8 | 2.198 [2.161(3)] | 1.975 | 1.976 |
| Fe2–*N*9 | 2.274 [2.189(3)] | 2.046 | 2.045 |
| Fe2–*N*10 | 2.152 [2.096(2)] | 1.933 | 1.931 |
| Fe2–*N*11 | 2.194 [2.165(3)] | 1.977 | 1.975 |
| Fe2–*N*12 | 2.274 [2.180(3)] | 2.040 | 2.051 |
| Fe1···Fe2 | 9.372 [8.1477(6)] | 9.151 | 8.944 |
| | | | |
| *N*1–Fe1–*N*2 | 73.6 [73.99(10)] | 73.4 | 79.8 |
| *N*1–Fe1–*N*3 | 74.0 [74.91(10)] | 74.2 | 79.5 |
| *N*1–Fe1–*N*4 ($\phi$ {Fe1}) | 166.6 [168.53(10)] | 166.9 | 170.3 |
| *N*1–Fe1–*N*5 | 97.1 [110.82(10)] | 97.4 | 93.0 |
| *N*1–Fe1–*N*6 | 116.2 [100.22(10)] | 115.3 | 107.9 |
| *N*2–Fe1–*N*3 | 147.2 [148.54(10)] | 147.2 | 159.3 |
| *N*2–Fe1–*N*4 | 97.2 [95.83(10)] | 97.1 | 93.7 |
| *N*2–Fe1–*N*5 | 92.9 [92.79(11)] | 93.0 | 91.1 |
| *N*2–Fe1–*N*6 | 97.1 [93.08(10)] | 95.4 | 91.6 |
| *N*3–Fe1–*N*4 | 115.7 [115.57(10)] | 115.7 | 107.0 |
| *N*3–Fe1–*N*5 | 96.2 [93.80(10)] | 96.3 | 91.1 |
| *N*3–Fe1–*N*6 | 92.3 [96.96(10)] | 93.5 | 93.7 |
| *N*4–Fe1–*N*5 | 73.3 [74.32(10)] | 73.7 | 79.8 |
| *N*4–Fe1–*N*6 | 74.0 [74.64(10)] | 74.0 | 79.3 |
| *N*5–Fe1–*N*6 | 146.8 [148.83(10)] | 147.3 | 159.1 |
| *N*7–Fe2–*N*8 | 73.7 [74.09(11)] | 79.8 | 79.8 |
| *N*7–Fe2–*N*9 | 74.1 [75.03(10)] | 79.3 | 79.3 |
| *N*7–Fe2–*N*10 ($\phi$ {Fe2}) | 167.5 [173.69(11)] | 170.0 | 169.7 |
| *N*7–Fe2–*N*11 | 96.6 [99.38(11)] | 93.8 | 92.5 |
| *N*7–Fe2–*N*12 | 116.5 [111.50(11)] | 107.1 | 108.5 |
| *N*8–Fe2–*N*9 | 146.5 [149.09(11)] | 159.0 | 159.0 |
| *N*8–Fe2–*N*10 | 100.0 [106.10(10)] | 92.8 | 93.5 |
| *N*8–Fe2–*N*11 | 100.7 [93.93(11)] | 91.8 | 91.6 |
| *N*8–Fe2–*N*12 | 91.1 [94.87(11)] | 91.0 | 90.9 |
| *N*9–Fe2–*N*10 | 113.4 [104.43(10)] | 108.2 | 107.4 |
| *N*9–Fe2–*N*11 | 91.8 [89.74(10)] | 90.8 | 90.8 |
| *N*9–Fe2–*N*12 | 95.3 [97.57(10)] | 94.0 | 94.2 |
| *N*10–Fe2–*N*11 | 73.6 [74.31(10)] | 79.7 | 79.8 |
| *N*10–Fe2–*N*12 | 73.8 [74.81(10)] | 79.4 | 79.3 |
| *N*11–Fe2–*N*12 | 146.9 [149.11(11)] | 159.0 | 159.1 |
| | | | |
| $\alpha$ {Fe1} | 73.7 [74.5(2)] | 73.8 | 79.6 |
| $\alpha$ {Fe2} | 73.8 [74.6(2)] | 79.6 | 79.6 |
| $\theta$ {Fe1} | 85.9 [85.96(3)] | 87.7 | 89.2 |
| $\theta$ {Fe2} | 88.4 [83.62(3)] | 87.8 | 87.6 |

[a]HS = high-spin, LS = low-spin. $\alpha$ is the average ligand bite angle in the molecule; see page S20 for definitions of $\phi$ and $\theta$.

**Table S10 (continued).**[a]

| | $[Fe_2(\mu\text{-}L^1)_2]^{4+}$ – conformation (c) | | |
|---|---|---|---|
| | HS–HS ($S$ = 4) | HS–LS ($S$ = 2) | LS–LS ($S$ = 0) |
| Fe1–*N*1 | 2.150 | 2.154 [2.117(6)] | 1.932 |
| Fe1–*N*2 | 2.200 | 2.193 [2.169(7)] | 1.973 |
| Fe1–*N*3 | 2.264 | 2.294 [2.153(6)] | 2.047 |
| Fe1–*N*4 | 2.154 | 2.153 [2.126(6)] | 1.931 |
| Fe1–*N*5 | 2.201 | 2.194 [2.207(8)] | 1.974 |
| Fe1–*N*6 | 2.266 | 2.288 [2.215(7)] | 2.050 |
| Fe2–*N*7 | 2.150 | 1.930 [1.923(6)] | 1.931 |
| Fe2–*N*8 | 2.199 | 1.974 [1.981(8)] | 1.973 |
| Fe2–*N*9 | 2.261 | 2.042 [2.040(8)] | 2.049 |
| Fe2–*N*10 | 2.149 | 1.931 [1.938(7)] | 1.932 |
| Fe2–*N*11 | 2.205 | 1.972 [1.963(7)] | 1.975 |
| Fe2–*N*12 | 2.280 | 2.043 [2.012(7)] | 2.045 |
| Fe1···Fe2 | 9.467 | 9.071 [7.8570(18)] | 8.942 |
| | | | |
| *N*1–Fe1–*N*2 | 73.5 | 73.8 [73.9(3)] | 79.8 |
| *N*1–Fe1–*N*3 | 74.3 | 73.7 [74.5(3)] | 79.4 |
| *N*1–Fe1–*N*4 ($\phi$ {Fe1}) | 162.0 | 168.1 [167.9(3)] | 169.9 |
| *N*1–Fe1–*N*5 | 94.3 | 98.8 [112.2(3)] | 93.4 |
| *N*1–Fe1–*N*6 | 119.2 | 114.3 [101.5(3)] | 107.5 |
| *N*2–Fe1–*N*3 | 147.3 | 147.0 [148.0(3)] | 159.2 |
| *N*2–Fe1–*N*4 | 93.6 | 98.3 [94.6(3)] | 92.8 |
| *N*2–Fe1–*N*5 | 93.2 | 100.7 [100.1(3)] | 91.6 |
| *N*2–Fe1–*N*6 | 95.9 | 90.3 [88.3(3)] | 91.0 |
| *N*3–Fe1–*N*4 | 119.1 | 114.8 [117.3(3)] | 108.0 |
| *N*3–Fe1–*N*5 | 95.1 | 90.0 [87.6(3)] | 90.5 |
| *N*3–Fe1–*N*6 | 94.4 | 97.6 [102.4(3)] | 94.4 |
| *N*4–Fe1–*N*5 | 73.4 | 73.6 [72.8(3)] | 79.9 |
| *N*4–Fe1–*N*6 | 73.9 | 73.9 [73.9(3)] | 79.3 |
| *N*5–Fe1–*N*6 | 146.5 | 146.8 [146.2(3)] | 159.1 |
| *N*7–Fe2–*N*8 | 73.5 | 79.9 [79.6(3)] | 79.9 |
| *N*7–Fe2–*N*9 | 74.1 | 79.3 [79.9(3)] | 79.3 |
| *N*7–Fe2–*N*10 ($\phi$ {Fe2}) | 162.3 | 169.9 [173.4(3)] | 169.7 |
| *N*7–Fe2–*N*11 | 94.0 | 93.2 [93.8(3)] | 93.2 |
| *N*7–Fe2–*N*12 | 118.9 | 107.6 [106.6(3)] | 107.7 |
| *N*8–Fe2–*N*9 | 146.7 | 159.1 [159.4(3)] | 159.1 |
| *N*8–Fe2–*N*10 | 94.4 | 93.0 [99.4(3)] | 92.9 |
| *N*8–Fe2–*N*11 | 93.8 | 91.5 [92.0(3)] | 91.7 |
| *N*8–Fe2–*N*12 | 94.8 | 91.0 [90.0(3)] | 90.6 |
| *N*9–Fe2–*N*10 | 118.8 | 107.0 [101.2(3)] | 107.9 |
| *N*9–Fe2–*N*11 | 95.6 | 91.4 [91.1(3)] | 90.8 |
| *N*9–Fe2–*N*12 | 94.4 | 93.5 [94.1(3)] | 94.5 |
| *N*10–Fe2–*N*11 | 73.5 | 79.8 [79.7(3)] | 79.7 |
| *N*10–Fe2–*N*12 | 74.3 | 79.5 [79.9(3)] | 79.4 |
| *N*11–Fe2–*N*12 | 147.1 | 159.2 [159.5(3)] | 159.1 |
| | | | |
| $\alpha$ {Fe1} | 73.8 | 73.8 [73.8(6)] | 79.6 |
| $\alpha$ {Fe2} | 73.8 | 79.6 [79.8(6)] | 79.6 |
| $\theta$ {Fe1} | 87.4 | 86.2 [73.87(7)] | 87.3 |
| $\theta$ {Fe2} | 87.8 | 89.0 [84.22(7)] | 87.3 |

[a]HS = high-spin, LS = low-spin. $\alpha$ is the average ligand bite angle in the molecule; see page S20 for definitions of $\phi$ and $\theta$.

**Table S11** Computed metric parameters for the helicate $[Fe_2(\mu\text{-}L^2)_2]^{4+}$ [Å, deg]. See Scheme S7 for the atom numbering scheme. Crystallographic values from **$2[ClO_4]_2$** are also given in square brackets.[a]

| | HS–HS ($S$ = 4) | HS–LS ($S$ = 2) | LS–LS ($S$ = 0) |
|---|---|---|---|
| Fe1–*N*1 | 2.151 [2.175(2)] | 2.150 | 1.931 |
| Fe1–*N*2 | 2.222 [2.185(3)] | 2.224 | 1.976 |
| Fe1–*N*3 | 2.281 [2.233(2)] | 2.322 | 2.062 |
| Fe1–*N*4 | 2.151 [2.207(2)] | 2.150 | 1.931 |
| Fe1–*N*5 | 2.222 [2.352(2)] | 2.224 | 1.976 |
| Fe1–*N*6 | 2.282 [2.196(2)] | 2.321 | 2.062 |
| Fe2–*N*7 | 2.151 [2.169(2)] | 1.929 | 1.931 |
| Fe2–*N*8 | 2.222 [2.241(2)] | 1.977 | 1.976 |
| Fe2–*N*9 | 2.281 [2.209(2)] | 2.044 | 2.062 |
| Fe2–*N*10 | 2.151 [2.167(2)] | 1.928 | 1.931 |
| Fe2–*N*11 | 2.222 [2.191(2)] | 1.973 | 1.976 |
| Fe2–*N*12 | 2.282 [2.244(2)] | 2.044 | 2.062 |
| Fe1···Fe2 | 7.418 [5.1401(6)] | 7.320 | 7.340 |
| | | | |
| *N*1–Fe1–*N*2 | 72.9 [72.42(9)] | 73.0 | 79.7 |
| *N*1–Fe1–*N*3 | 74.4 [71.90(10)] | 74.2 | 79.1 |
| *N*1–Fe1–*N*4 ($\phi$ {Fe1}) | 155.8 [148.68(9)] | 154.7 | 168.9 |
| *N*1–Fe1–*N*5 | 90.5 [80.66(9)] | 89.9 | 92.6 |
| *N*1–Fe1–*N*6 | 122.6 [139.79(9)] | 122.7 | 108.5 |
| *N*2–Fe1–*N*3 | 146.7 [143.69(9)] | 146.8 | 158.8 |
| *N*2–Fe1–*N*4 | 90.4 [120.93(9)] | 89.9 | 92.6 |
| *N*2–Fe1–*N*5 | 94.3 [101.85(9)] | 95.7 | 93.0 |
| *N*2–Fe1–*N*6 | 92.1 [85.29(9)] | 89.2 | 88.7 |
| *N*3–Fe1–*N*4 | 122.6 [93.58(9)] | 122.7 | 108.5 |
| *N*3–Fe1–*N*5 | 92.2 [78.78(9)] | 89.2 | 88.7 |
| *N*3–Fe1–*N*6 | 100.1 [118.94(9)] | 104.3 | 97.3 |
| *N*4–Fe1–*N*5 | 72.9 [69.16(9)] | 73.0 | 79.7 |
| *N*4–Fe1–*N*6 | 74.4 [71.52(9)] | 74.2 | 79.1 |
| *N*5–Fe1–*N*6 | 146.7 [137.70(8)] | 146.8 | 158.8 |
| *N*7–Fe2–*N*8 | 72.9 [71.31(9)] | 79.8 | 79.7 |
| *N*7–Fe2–*N*9 | 74.4 [72.19(9)] | 79.3 | 79.1 |
| *N*7–Fe2–*N*10 ($\phi$ {Fe2}) | 155.8 [151.50(9)] | 168.8 | 168.9 |
| *N*7–Fe2–*N*11 | 90.5 [124.89(9)] | 92.5 | 92.6 |
| *N*7–Fe2–*N*12 | 122.6 [94.57(9)] | 108.4 | 108.5 |
| *N*8–Fe2–*N*9 | 146.7 [141.29(10)] | 159.0 | 158.8 |
| *N*8–Fe2–*N*10 | 90.4 [82.47(9)] | 92.4 | 92.6 |
| *N*8–Fe2–*N*11 | 94.3 [106.08(9)] | 92.6 | 93.0 |
| *N*8–Fe2–*N*12 | 92.1 [84.51(9)] | 89.4 | 88.7 |
| *N*9–Fe2–*N*10 | 122.6 [135.63(9)] | 108.5 | 108.5 |
| *N*9–Fe2–*N*11 | 92.2 [84.68(9)] | 89.5 | 88.7 |
| *N*9–Fe2–*N*12 | 100.1 [110.89(9)] | 96.1 | 97.3 |
| *N*10–Fe2–*N*11 | 72.9 [72.47(9)] | 79.8 | 79.7 |
| *N*10–Fe2–*N*12 | 74.4 [71.45(9)] | 79.4 | 79.1 |
| *N*11–Fe2–*N*12 | 146.7 [140.54(9)] | 159.1 | 158.8 |
| | | | |
| $\alpha$ {Fe1} | 73.7 [71.25(19)] | 73.6 | 79.4 |
| $\alpha$ {Fe2} | 73.7 [71.89(18)] | 79.6 | 79.4 |
| $\theta$ {Fe1} | 87.3 [52.71(5)] | 82.8 | 81.5 |
| $\theta$ {Fe2} | 87.3 [56.30(4)] | 83.7 | 81.5 |

[a]HS = high-spin, LS = low-spin. $\alpha$ is the average ligand bite angle in the molecule; see page S20 for definitions of $\phi$ and $\theta$.

The calculations reproduce the large $\phi$ coordination geometry distortion in the crystal structures, but badly underestimate the $\theta$ distortion (page S20).

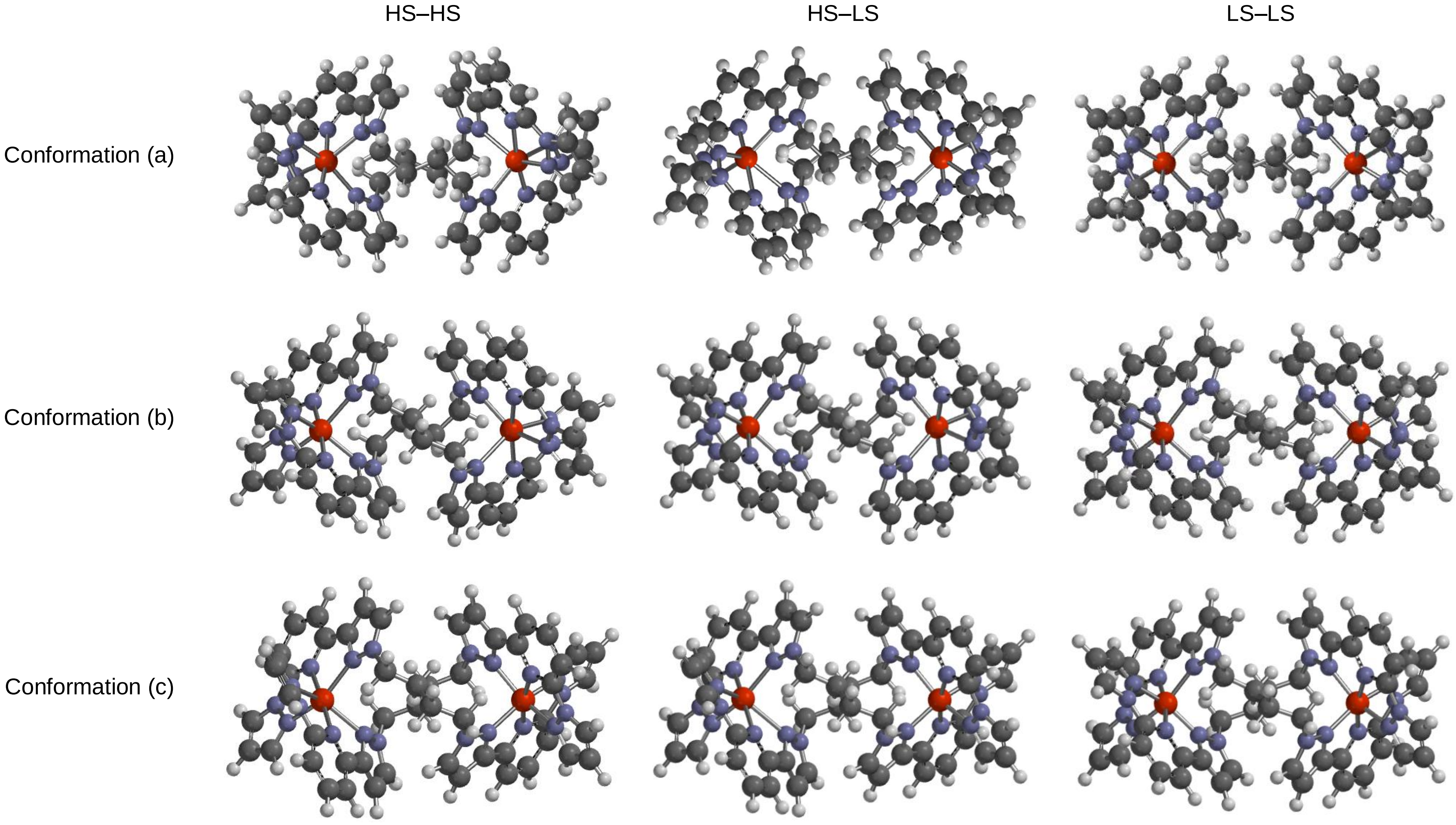


**Figure S44** Views of the three conformations of $[Cr_2(\mu\text{-}L^1)_2]^0$, minimized in their high-spin, mixed-spin and low-spin states (HS = high-spin, LS = low-spin). Color code: C, dark gray; H, white; Cr, orange; N, blue.

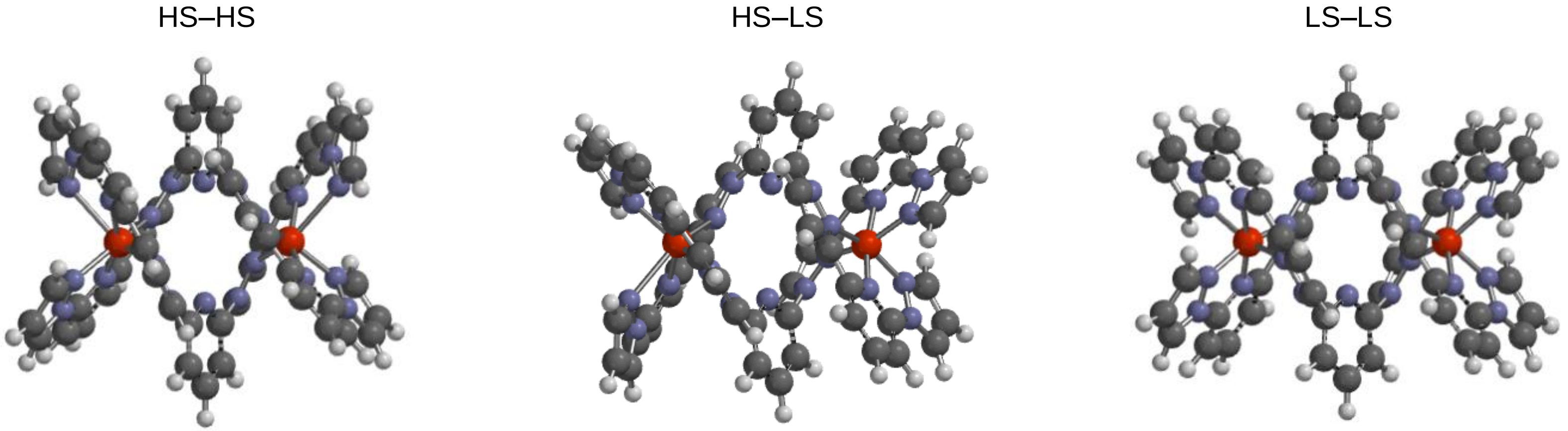


**Figure S45** Views of energy-minimized $[Cr_2(\mu\text{-}L^2)_2]^0$, minimized in its high-spin, mixed-spin and low-spin states. Details as for Figure S44.

**Discussion of the chromium complex minimizations**

The minimized low-spin ($S$ = 0) chromium(0) complex structures are chemically reasonable, and consistent with that formulation. However, the “high-spin” chromium centers have more varied coordination geometries, mostly with pronounced axial elongations or tending towards five-coordination with one very long Cr–N bond. These are more reminiscent of high-spin, Jahn-Teller-active chromium(II) ions.

The minimized mixed-spin ($S$ = 2) $Cr_2$ helicates consistently contain one low-spin Cr(0) ion and one high-spin Cr(II) center. This is evident from their metric parameters; and, from their $\alpha$- and $\beta$-HOMOs which are both $\pi$-orbitals localized on different bpp moieties, coordinated to the putative Cr(II) ion (Figure S47). We assign the formal electronic structure $[Cr^0Cr^{II}(L^{\bullet -})_2]$ (L = $L^1$-$L^3$), with antiferromagnetically coupled ligand radical spins, to those computed molecules.

The minimized high-spin ($S$ = 4) $Cr_2$ helicates are more variable. One Cr atom always resembles the Cr(II) ions in the mixed-spin minimizations, but the structure of the other Cr atom varies significantly between the compounds. In some minimizations the second Cr atom also has a distorted Cr(II)-like geometry, while in others it has a more regular pattern of Cr–N bonds which are just slightly longer than in the low-spin Cr(0) case. The pattern of $\alpha$- and $\beta$-frontier orbitals in these compounds also differs between the molecules, although their HOMOs are always ligand-centered.

The detailed interpretation of the high-spin chromium minimizations is complex, and beyond the scope of this study. However, it is clear that high-spin chromium(0) complexes do not exist by this protocol, and the spin state energies of the iron complexes and their isoelectronic chromium analogues cannot be directly compared.

**Table S12** Computed metric parameters for the different helicate conformations of $[Cr_2(\mu\text{-}L^1)_2]^0$ [Å, deg]. See Scheme S7 for the atom numbering scheme in the Table.[a]

| | Conformation (a) | | |
|---|---|---|---|
| | HS–HS ($S$ = 4)[b] | HS–LS ($S$ = 2)[c] | LS–LS ($S$ = 0) |
| Cr1–*N*1 | 2.074 | 1.962 | 1.961 |
| Cr1–*N*2 | 2.207 | 2.114 | 2.008 |
| Cr1–*N*3 | 2.162 | 2.121 | 2.030 |
| Cr1–*N*4 | 2.162 | 2.168 | 1.961 |
| Cr1–*N*5 | 2.430 | 2.217 | 2.008 |
| Cr1–*N*6 | 2.436 | 2.562 | 2.030 |
| Cr2–*N*7 | 1.984 | 1.960 | 1.961 |
| Cr2–*N*8 | 2.025 | 2.013 | 2.008 |
| Cr2–*N*9 | 2.158 | 2.034 | 2.030 |
| Cr2–*N*10 | 1.964 | 1.956 | 1.961 |
| Cr2–*N*11 | 2.042 | 2.010 | 2.008 |
| Cr2–*N*12 | 2.107 | 2.038 | 2.030 |
| Cr1···Cr2 | 8.512 | 8.711 | 8.462 |
| | | | |
| *N*1–Cr1–*N*2 | 73.8 | 76.5 | 77.3 |
| *N*1–Cr1–*N*3 | 76.5 | 77.2 | 77.0 |
| *N*1–Cr1–*N*4 ($\phi$ {Cr1}) | 169.2 | 168.8 | 169.7 |
| *N*1–Cr1–*N*5 | 110.6 | 114.8 | 95.5 |
| *N*1–Cr1–*N*6 | 105.3 | 102.3 | 110.1 |
| *N*2–Cr1–*N*3 | 150.3 | 153.3 | 154.3 |
| *N*2–Cr1–*N*4 | 95.5 | 95.0 | 95.5 |
| *N*2–Cr1–*N*5 | 95.4 | 99.1 | 92.6 |
| *N*2–Cr1–*N*6 | 90.9 | 88.4 | 91.0 |
| *N*3–Cr1–*N*4 | 114.2 | 110.4 | 110.1 |
| *N*3–Cr1–*N*5 | 92.9 | 96.2 | 91.0 |
| *N*3–Cr1–*N*6 | 99.0 | 92.4 | 96.7 |
| *N*4–Cr1–*N*5 | 71.0 | 73.3 | 77.3 |
| *N*4–Cr1–*N*6 | 73.0 | 69.9 | 77.0 |
| *N*5–Cr1–*N*6 | 143.9 | 143.0 | 154.3 |
| *N*7–Cr2–*N*8 | 77.0 | 77.2 | 77.3 |
| *N*7–Cr2–*N*9 | 76.4 | 76.9 | 77.0 |
| *N*7–Cr2–*N*10 ($\phi$ {Cr2}) | 173.9 | 169.7 | 169.7 |
| *N*7–Cr2–*N*11 | 97.1 | 96.1 | 95.5 |
| *N*7–Cr2–*N*12 | 108.9 | 109.6 | 110.1 |
| *N*8–Cr2–*N*9 | 153.1 | 154.2 | 154.3 |
| *N*8–Cr2–*N*10 | 100.8 | 94.9 | 95.5 |
| *N*8–Cr2–*N*11 | 94.6 | 92.1 | 92.6 |
| *N*8–Cr2–*N*12 | 92.7 | 92.3 | 91.0 |
| *N*9–Cr2–*N*10 | 106.1 | 110.8 | 110.1 |
| *N*9–Cr2–*N*11 | 92.4 | 91.6 | 91.0 |
| *N*9–Cr2–*N*12 | 92.4 | 95.4 | 96.7 |
| *N*10–Cr2–*N*11 | 77.3 | 77.3 | 77.3 |
| *N*10–Cr2–*N*12 | 76.7 | 77.1 | 77.0 |
| *N*11–Cr2–*N*12 | 153.9 | 154.3 | 154.3 |
| | | | |
| $\alpha$ {Cr1} | 73.6 | 74.2 | 77.2 |
| $\alpha$ {Cr2} | 76.9 | 77.1 | 77.2 |
| $\theta$ {Cr1} | 86.6 | 83.9 | 87.8 |
| $\theta$ {Cr2} | 84.9 | 89.8 | 87.8 |

[a]HS = high-spin, LS = low-spin. $\alpha$ is the average ligand bite angle in the molecule; see page S20 for definitions of $\phi$ and $\theta$. [b]Cr1 in this molecule minimized to a Jahn-Teller elongated [4+2] coordination geometry, but Cr2 has a more regular pseudo-octahedral geometry. [c]High-spin Cr1 in this molecule minimized to a pseudo-five-coordinate [5+1] coordination geometry, with one very long Cr–N interaction.

**Table S12 (continued).**[a]

| | Conformation (b) | | |
|---|---|---|---|
| | HS–HS ($S$ = 4)[b] | HS–LS ($S$ = 2)[c] | LS–LS ($S$ = 0) |
| Cr1–*N*1 | 2.088 | 1.971 | 1.958 |
| Cr1–*N*2 | 2.266 | 2.125 | 2.013 |
| Cr1–*N*3 | 2.169 | 2.125 | 2.035 |
| Cr1–*N*4 | 2.158 | 2.190 | 1.959 |
| Cr1–*N*5 | 2.410 | 2.516 | 2.011 |
| Cr1–*N*6 | 2.430 | 2.262 | 2.042 |
| Cr2–*N*7 | 1.981 | 1.958 | 1.959 |
| Cr2–*N*8 | 2.042 | 2.012 | 2.011 |
| Cr2–*N*9 | 2.205 | 2.040 | 2.038 |
| Cr2–*N*10 | 1.980 | 1.957 | 1.962 |
| Cr2–*N*11 | 2.042 | 2.013 | 2.010 |
| Cr2–*N*12 | 2.204 | 2.032 | 2.031 |
| Cr1···Cr2 | 8.371 | 8.309 | 8.522 |
| | | | |
| *N*1–Cr1–*N*2 | 72.7 | 76.3 | 77.3 |
| *N*1–Cr1–*N*3 | 76.7 | 77.1 | 77.1 |
| *N*1–Cr1–*N*4 ($\phi$ {Cr1}) | 169.7 | 168.9 | 168.5 |
| *N*1–Cr1–*N*5 | 109.4 | 104.5 | 94.7 |
| *N*1–Cr1–*N*6 | 105.6 | 112.7 | 110.9 |
| *N*2–Cr1–*N*3 | 149.3 | 152.1 | 154.3 |
| *N*2–Cr1–*N*4 | 97.1 | 95.1 | 94.7 |
| *N*2–Cr1–*N*5 | 92.9 | 92.1 | 92.4 |
| *N*2–Cr1–*N*6 | 90.6 | 93.7 | 91.1 |
| *N*3–Cr1–*N*4 | 113.5 | 110.1 | 110.8 |
| *N*3–Cr1–*N*5 | 94.8 | 86.8 | 90.5 |
| *N*3–Cr1–*N*6 | 100.1 | 104.0 | 97.2 |
| *N*4–Cr1–*N*5 | 71.4 | 68.3 | 77.3 |
| *N*4–Cr1–*N*6 | 72.8 | 74.5 | 77.1 |
| *N*5–Cr1–*N*6 | 144.2 | 142.7 | 154.3 |
| *N*7–Cr2–*N*8 | 76.9 | 77.3 | 77.3 |
| *N*7–Cr2–*N*9 | 75.6 | 77.1 | 77.1 |
| *N*7–Cr2–*N*10 ($\phi$ {Cr2}) | 175.3 | 169.3 | 169.6 |
| *N*7–Cr2–*N*11 | 99.4 | 95.2 | 94.2 |
| *N*7–Cr2–*N*12 | 108.2 | 110.3 | 111.6 |
| *N*8–Cr2–*N*9 | 151.7 | 154.3 | 154.3 |
| *N*8–Cr2–*N*10 | 100.6 | 95.2 | 96.7 |
| *N*8–Cr2–*N*11 | 98.8 | 92.3 | 92.1 |
| *N*8–Cr2–*N*12 | 91.5 | 91.2 | 91.3 |
| *N*9–Cr2–*N*10 | 107.3 | 110.4 | 108.8 |
| *N*9–Cr2–*N*11 | 92.1 | 91.3 | 91.3 |
| *N*9–Cr2–*N*12 | 90.8 | 96.4 | 96.7 |
| *N*10–Cr2–*N*11 | 77.0 | 77.3 | 77.3 |
| *N*10–Cr2–*N*12 | 75.6 | 77.1 | 76.8 |
| *N*11–Cr2–*N*12 | 152.1 | 154.4 | 154.1 |
| | | | |
| $\alpha$ {Cr1} | 73.4 | 74.1 | 77.2 |
| $\alpha$ {Cr2} | 76.3 | 77.2 | 77.1 |
| $\theta$ {Cr1} | 87.7 | 83.1 | 88.4 |
| $\theta$ {Cr2} | 84.7 | 88.4 | 87.6 |

[a]HS = high-spin, LS = low-spin. $\alpha$ is the average ligand bite angle in the molecule; see page S20 for definitions of $\phi$ and $\theta$. [b]Cr1 in this molecule minimized to a Jahn-Teller elongated [4+2] coordination geometry, but Cr2 has a more regular pseudo-octahedral geometry. [c]High-spin Cr1 in this molecule minimized to a pseudo-five-coordinate [5+1] coordination geometry, with one very long Cr–N interaction.

**Table S12 (continued).**[a]

| | Conformation (c) | | |
|---|---|---|---|
| | HS–HS ($S$ = 4)[b] | HS–LS ($S$ = 2)[c] | LS–LS ($S$ = 0) |
| Cr1–*N*1 | 2.064 | 1.968 | 1.959 |
| Cr1–*N*2 | 2.121 | 2.092 | 2.011 |
| Cr1–*N*3 | 2.205 | 2.156 | 2.036 |
| Cr1–*N*4 | 2.153 | 2.169 | 1.959 |
| Cr1–*N*5 | 2.449 | 2.226 | 2.013 |
| Cr1–*N*6 | 2.468 | 2.588 | 2.037 |
| Cr2–*N*7 | 1.972 | 1.963 | 1.962 |
| Cr2–*N*8 | 2.044 | 2.011 | 2.012 |
| Cr2–*N*9 | 2.089 | 2.032 | 2.042 |
| Cr2–*N*10 | 1.969 | 1.961 | 1.960 |
| Cr2–*N*11 | 2.061 | 2.009 | 2.012 |
| Cr2–*N*12 | 2.065 | 2.026 | 2.032 |
| Cr1···Cr2 | 8.578 | 8.546 | 8.355 |
| | | | |
| *N*1–Cr1–*N*2 | 75.6 | 76.9 | 77.3 |
| *N*1–Cr1–*N*3 | 75.5 | 76.7 | 77.2 |
| *N*1–Cr1–*N*4 ($\phi$ {Cr1}) | 177.1 | 174.5 | 169.7 |
| *N*1–Cr1–*N*5 | 106.1 | 110.9 | 95.9 |
| *N*1–Cr1–*N*6 | 109.7 | 106.2 | 109.4 |
| *N*2–Cr1–*N*3 | 151.1 | 152.8 | 154.5 |
| *N*2–Cr1–*N*4 | 102.6 | 99.3 | 95.1 |
| *N*2–Cr1–*N*5 | 99.9 | 103.5 | 92.2 |
| *N*2–Cr1–*N*6 | 92.4 | 87.9 | 90.3 |
| *N*3–Cr1–*N*4 | 106.4 | 106.6 | 110.2 |
| *N*3–Cr1–*N*5 | 89.1 | 91.6 | 90.3 |
| *N*3–Cr1–*N*6 | 96.2 | 93.6 | 98.2 |
| *N*4–Cr1–*N*5 | 71.8 | 73.6 | 77.4 |
| *N*4–Cr1–*N*6 | 72.5 | 69.6 | 77.2 |
| *N*5–Cr1–*N*6 | 144.0 | 172.7 | 154.5 |
| *N*7–Cr2–*N*8 | 76.8 | 77.2 | 77.3 |
| *N*7–Cr2–*N*9 | 77.2 | 76.9 | 77.0 |
| *N*7–Cr2–*N*10 ($\phi$ {Cr2}) | 170.1 | 169.6 | 169.6 |
| *N*7–Cr2–*N*11 | 96.6 | 95.7 | 95.0 |
| *N*7–Cr2–*N*12 | 109.4 | 110.1 | 110.6 |
| *N*8–Cr2–*N*9 | 154.0 | 154.1 | 154.2 |
| *N*8–Cr2–*N*10 | 96.2 | 95.1 | 96.0 |
| *N*8–Cr2–*N*11 | 93.9 | 92.2 | 92.9 |
| *N*8–Cr2–*N*12 | 92.2 | 92.0 | 90.2 |
| *N*9–Cr2–*N*10 | 109.9 | 110.8 | 109.6 |
| *N*9–Cr2–*N*11 | 90.9 | 92.0 | 89.9 |
| *N*9–Cr2–*N*12 | 94.6 | 95.3 | 98.3 |
| *N*10–Cr2–*N*11 | 76.8 | 77.4 | 77.3 |
| *N*10–Cr2–*N*12 | 77.5 | 76.9 | 77.0 |
| *N*11–Cr2–*N*12 | 154.0 | 154.2 | 154.3 |
| | | | |
| $\alpha$ {Cr1} | 73.9 | 74.2 | 77.3 |
| $\alpha$ {Cr2} | 77.1 | 77.1 | 77.2 |
| $\theta$ {Cr1} | 76.9 | 77.8 | 85.6 |
| $\theta$ {Cr2} | 89.8 | 89.7 | 85.3 |

[a]HS = high-spin, LS = low-spin. $\alpha$ is the average ligand bite angle in the molecule; see page S20 for definitions of $\phi$ and $\theta$. [b]Cr1 in this molecule minimized to a Jahn-Teller elongated [4+2] coordination geometry, but Cr2 has a more regular pseudo-octahedral geometry. [c]High-spin Cr1 in this molecule minimized to a pseudo-five-coordinate [5+1] coordination geometry, with one very long Cr–N interaction.

**Table S13** Computed metric parameters for $[Cr_2(\mu\text{-}L^2)_2]^0$ [Å, deg]. See Scheme S7 for the atom numbering scheme in the Table.[a]

| | HS–HS ($S$ = 4)[b] | HS–LS ($S$ = 2)[b] | LS–LS ($S$ = 0) |
|---|---|---|---|
| Cr1–*N*1 | 1.988 | 1.988 | 1.961 |
| Cr1–*N*2 | 2.157 | 2.143 | 2.011 |
| Cr1–*N*3 | 2.117 | 2.139 | 2.049 |
| Cr1–*N*4 | 2.211 | 2.215 | 1.961 |
| Cr1–*N*5 | 2.608 | 2.623 | 2.010 |
| Cr1–*N*6 | 2.288 | 2.372 | 2.049 |
| Cr2–*N*7 | 1.988 | 1.969 | 1.961 |
| Cr2–*N*8 | 2.157 | 2.015 | 2.011 |
| Cr2–*N*9 | 2.117 | 2.052 | 2.049 |
| Cr2–*N*10 | 2.211 | 1.963 | 1.961 |
| Cr2–*N*11 | 2.608 | 2.010 | 2.011 |
| Cr2–*N*12 | 2.289 | 2.051 | 2.049 |
| Cr1···Cr2 | 5.781 | 6.362 | 6.720 |
| | | | |
| *N*1–Cr1–*N*2 | 75.7 | 75.8 | 77.2 |
| *N*1–Cr1–*N*3 | 76.8 | 76.7 | 76.8 |
| *N*1–Cr1–*N*4 ($\phi$ {Cr1}) | 159.1 | 157.3 | 168.5 |
| *N*1–Cr1–*N*5 | 95.3 | 93.3 | 94.9 |
| *N*1–Cr1–*N*6 | 121.4 | 124.3 | 110.5 |
| *N*2–Cr1–*N*3 | 151.2 | 151.9 | 154.0 |
| *N*2–Cr1–*N*4 | 92.2 | 92.6 | 94.9 |
| *N*2–Cr1–*N*5 | 93.1 | 96.7 | 94.6 |
| *N*2–Cr1–*N*6 | 84.2 | 83.5 | 86.0 |
| *N*3–Cr1–*N*4 | 111.2 | 110.9 | 110.5 |
| *N*3–Cr1–*N*5 | 81.0 | 78.8 | 86.0 |
| *N*3–Cr1–*N*6 | 117.9 | 117.6 | 104.7 |
| *N*4–Cr1–*N*5 | 68.1 | 68.3 | 77.2 |
| *N*4–Cr1–*N*6 | 73.0 | 72.4 | 76.8 |
| *N*5–Cr1–*N*6 | 140.9 | 140.7 | 154.0 |
| *N*7–Cr2–*N*8 | 75.7 | 77.2 | 77.2 |
| *N*7–Cr2–*N*9 | 76.8 | 76.7 | 76.8 |
| *N*7–Cr2–*N*10 ($\phi$ {Cr2}) | 159.1 | 170.2 | 168.5 |
| *N*7–Cr2–*N*11 | 95.3 | 96.5 | 94.9 |
| *N*7–Cr2–*N*12 | 121.4 | 109.0 | 110.5 |
| *N*8–Cr2–*N*9 | 151.1 | 153.8 | 154.0 |
| *N*8–Cr2–*N*10 | 92.2 | 95.5 | 94.9 |
| *N*8–Cr2–*N*11 | 93.1 | 93.8 | 94.6 |
| *N*8–Cr2–*N*12 | 84.2 | 87.6 | 86.0 |
| *N*9–Cr2–*N*10 | 111.2 | 110.2 | 110.5 |
| *N*9–Cr2–*N*11 | 81.0 | 87.2 | 86.0 |
| *N*9–Cr2–*N*12 | 117.9 | 102.7 | 104.7 |
| *N*10–Cr2–*N*11 | 68.1 | 77.3 | 77.2 |
| *N*10–Cr2–*N*12 | 73.0 | 76.8 | 76.8 |
| *N*11–Cr2–*N*12 | 140.8 | 154.1 | 154.0 |
| | | | |
| $\alpha$ {Cr1} | 73.4 | 73.3 | 77.0 |
| $\alpha$ {Cr2} | 73.4 | 77.0 | 77.0 |
| $\theta$ {Cr1} | 67.9 | 68.1 | 76.4 |
| $\theta$ {Cr2} | 67.9 | 79.2 | 76.4 |

[a]HS = high-spin, LS = low-spin. $\alpha$ is the average ligand bite angle in the molecule; see page S20 for definitions of $\phi$ and $\theta$. [b]All the high-spin Cr ions in these spin states minimized to a pseudo-five-coordinate [5+1] coordination geometry, with one very long Cr–N interaction.

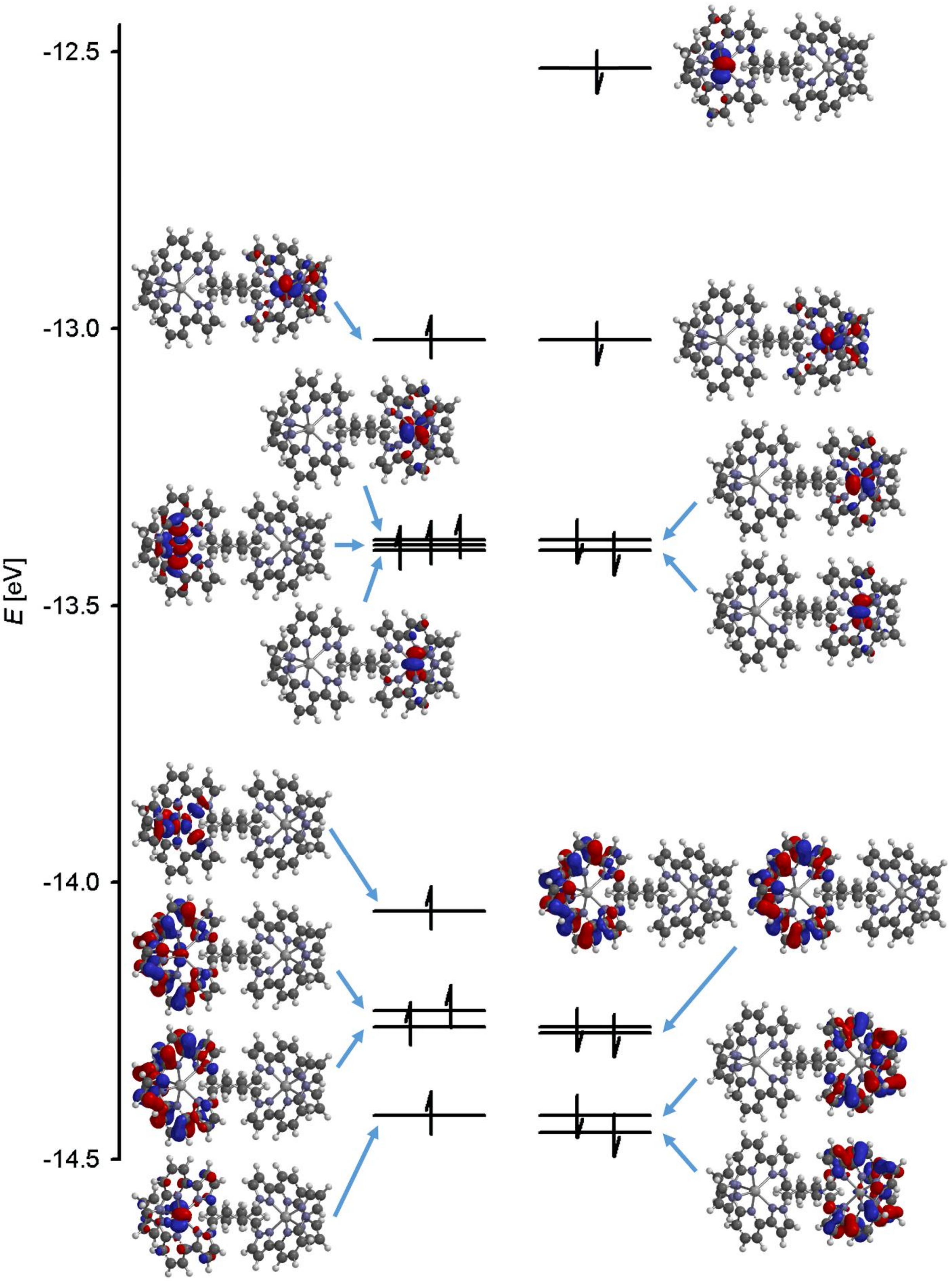


**Figure S46** Spin-unrestricted frontier HOMO orbitals of the mixed-spin state of $[Fe_2(\mu\text{-}L^1)_2]^{4+}$ [conformation (a)]. The high-spin iron atom is on the left in the orbital plots, and the low-spin iron atom is on the right.

The diagram includes the paired $\alpha$ and $\beta$ *d*-orbital spins in the low-spin iron atom; the one *d*-orbital spin pair in the high-spin iron atom; two of the four $\alpha$-spin unpaired *d*-electrons on the high-spin iron atom (the other two lie below –14.5 eV); and, spin-paired ligand-centered $\pi$-orbitals. There are no ligand-centered unpaired spins in this diagram, in contrast to the chromium complex in the next Figure.

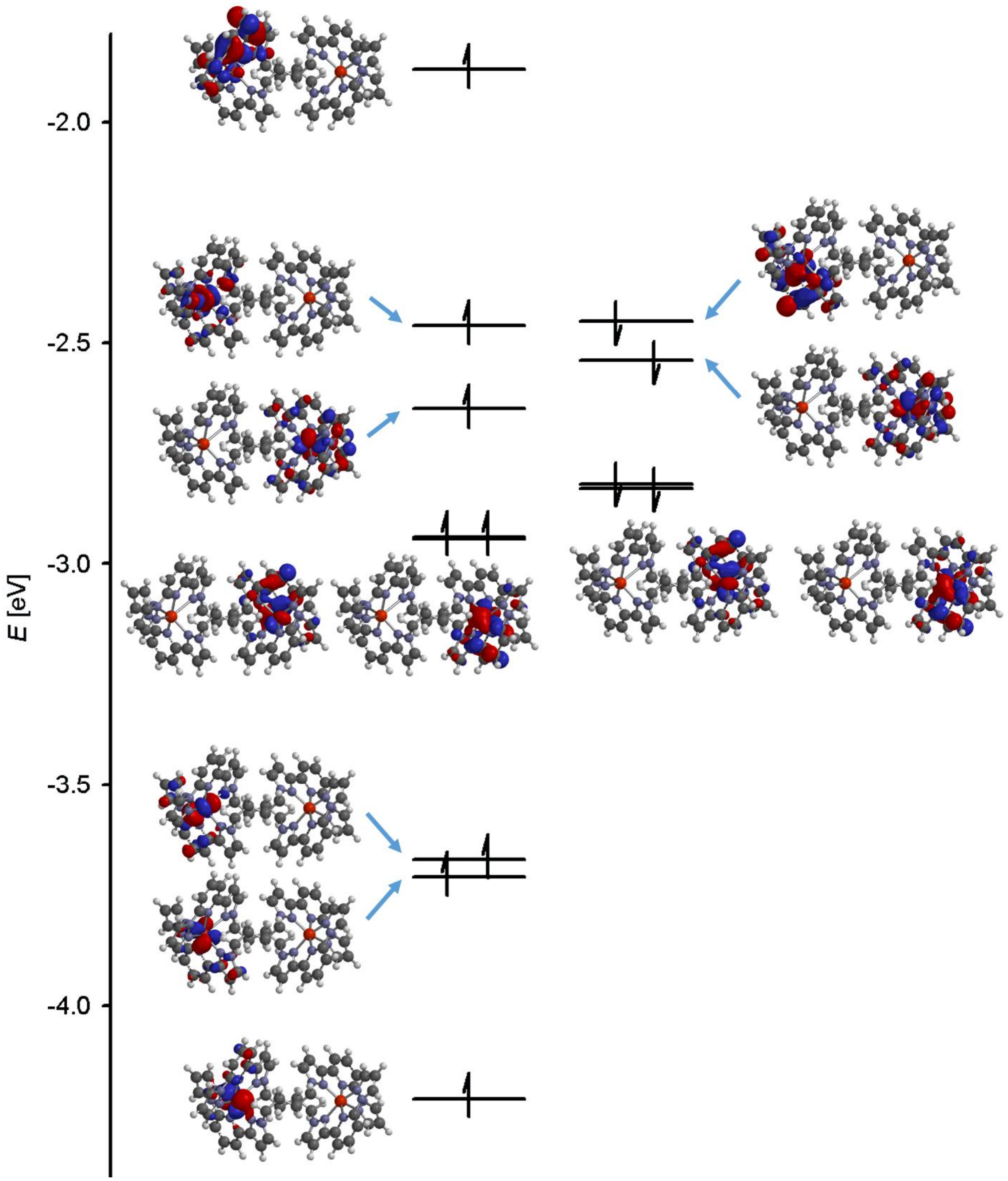


**Figure S47** Spin-unrestricted frontier HOMO orbitals of the mixed-spin state of $[Cr_2(\mu\text{-}L^1)_2]^0$ [conformation (a)]. The high-spin chromium atom is on the left in the orbital plots, and the low-spin chromium atom is on the right.

The next highest occupied MOs, below –5 eV, are spin-paired ligand-based $\pi$-orbitals.

The $\alpha$- and $\beta$-HOMOs are antiferromagnetically coupled unpaired spins, on the two different bpp moieties coordinated to the high-spin chromium ion. The paired $\alpha$ and $\beta$ spins in the low-spin chromium atom $t_{2g}$ *d*-orbitals, and the four unpaired $\alpha$ spins in the "high-spin" chromium atom *d*-orbitals, are also present in the diagram.

This electron distribution is consistent with a $[Cr^0Cr^{II}(L^{1\bullet-})_2]$ formulation, with a high-spin chromium(II) center. That is supported by the metric parameters in the minimized model (Table S12).

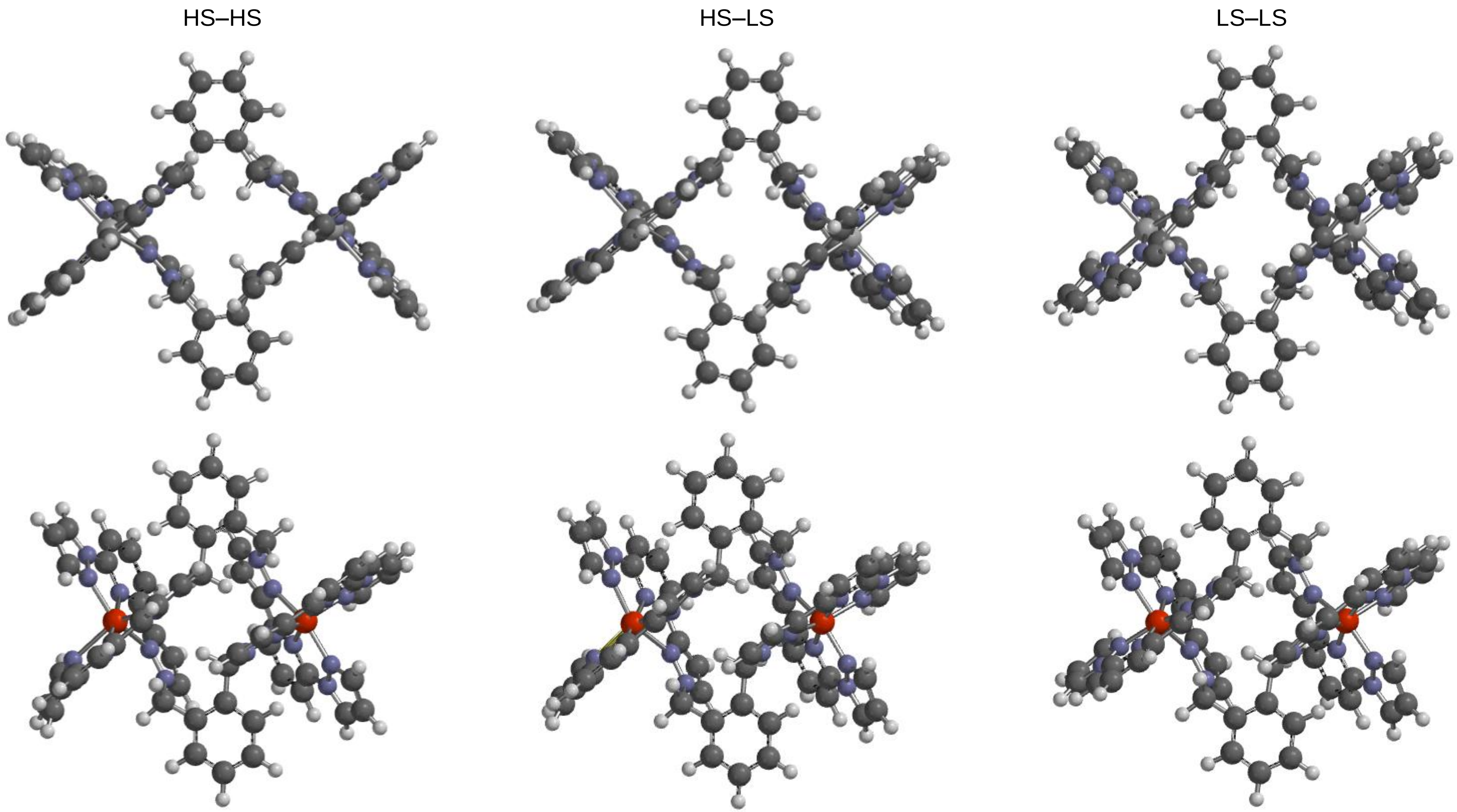


**Figure S48** The two conformational minima found for $[Fe_2(\mu\text{-}L^3)_2]^{4+}$ (top) and $[Cr_2(\mu\text{-}L^3)_2]^0$ (bottom), from DFT minimizations in their high-spin, mixed-spin and low-spin states. Details as for Figure S42 and S44.

MM2 minimizations of $[M_2(\mu\text{-}L^3)_2]^{n+}$ (M = Fe(II) or Cr(0)) yielded the bottom conformation in the Figure, which was retained when the chromium complex was minimized by DFT. However DFT minimizations of $[Fe_2(\mu\text{-}L^3)_2]^{4+}$, starting from the MM2 structure, transformed to the more extended top conformation. Since no crystallographic or magnetic data are available for $[Fe_2(\mu\text{-}L^3)_2]^{4+}$, the relevance of these conformations to its experimental properties is unclear.

**Table S14** Computed metric parameters for the helicates $[Fe_2(\mu\text{-}L^3)_2]^{4+}$ and $[Cr_2(\mu\text{-}L^3)_2]^0$ [Å, deg]. See Scheme S7 for the atom numbering scheme in the Table.[a]

| | $[Fe_2(\mu\text{-}L^3)_2]^{4+}$ | | |
|---|---|---|---|
| | HS–HS ($S$ = 4) | HS–LS ($S$ = 2) | LS–LS ($S$ = 0) |
| Fe1–*N*1 | 2.156 | 2.161 | 1.935 |
| Fe1–*N*2 | 2.209 | 2.211 | 1.975 |
| Fe1–*N*3 | 2.267 | 2.262 | 2.035 |
| Fe1–*N*4 | 2.158 | 2.160 | 1.936 |
| Fe1–*N*5 | 2.213 | 2.208 | 1.975 |
| Fe1–*N*6 | 2.249 | 2.282 | 2.040 |
| Fe2–*N*7 | 2.160 | 1.933 | 1.935 |
| Fe2–*N*8 | 2.215 | 1.975 | 1.975 |
| Fe2–*N*9 | 2.249 | 2.041 | 2.039 |
| Fe2–*N*10 | 2.159 | 1.934 | 1.934 |
| Fe2–*N*11 | 2.212 | 1.975 | 1.976 |
| Fe2–*N*12 | 2.273 | 2.036 | 2.049 |
| Fe1···Fe2 | 9.171 | 8.890 | 8.529 |
| | | | |
| *N*1–Fe1–*N*2 | 73.2 | 73.0 | 79.8 |
| *N*1–Fe1–*N*3 | 74.0 | 73.9 | 79.0 |
| *N*1–Fe1–*N*4 ($\phi$ {Fe1}) | 160.0 | 157.2 | 169.4 |
| *N*1–Fe1–*N*5 | 92.7 | 91.1 | 93.3 |
| *N*1–Fe1–*N*6 | 121.1 | 123.1 | 108.0 |
| *N*2–Fe1–*N*3 | 146.4 | 145.7 | 158.8 |
| *N*2–Fe1–*N*4 | 93.3 | 91.1 | 92.3 |
| *N*2–Fe1–*N*5 | 93.2 | 93.1 | 91.6 |
| *N*2–Fe1–*N*6 | 95.8 | 96.1 | 91.2 |
| *N*3–Fe1–*N*4 | 120.4 | 123.2 | 108.9 |
| *N*3–Fe1–*N*5 | 95.6 | 96.0 | 91.0 |
| *N*3–Fe1–*N*6 | 94.6 | 94.7 | 94.0 |
| *N*4–Fe1–*N*5 | 73.0 | 73.2 | 79.8 |
| *N*4–Fe1–*N*6 | 74.1 | 73.8 | 79.1 |
| *N*5–Fe1–*N*6 | 146.3 | 145.8 | 158.8 |
| *N*7–Fe2–*N*8 | 72.9 | 79.8 | 79.8 |
| *N*7–Fe2–*N*9 | 74.0 | 79.2 | 79.1 |
| *N*7–Fe2–*N*10 ($\phi$ {Fe2}) | 157.8 | 169.5 | 169.6 |
| *N*7–Fe2–*N*11 | 91.3 | 93.4 | 92.5 |
| *N*7–Fe2–*N*12 | 122.9 | 107.8 | 108.7 |
| *N*8–Fe2–*N*9 | 145.8 | 158.9 | 158.8 |
| *N*8–Fe2–*N*10 | 91.7 | 92.4 | 93.3 |
| *N*8–Fe2–*N*11 | 92.7 | 91.4 | 91.7 |
| *N*8–Fe2–*N*12 | 96.9 | 91.6 | 91.1 |
| *N*9–Fe2–*N*10 | 122.5 | 108.7 | 107.9 |
| *N*9–Fe2–*N*11 | 96.6 | 91.4 | 91.1 |
| *N*9–Fe2–*N*12 | 93.6 | 93.3 | 93.9 |
| *N*10–Fe2–*N*11 | 73.1 | 79.8 | 79.8 |
| *N*10–Fe2–*N*12 | 73.9 | 79.2 | 79.0 |
| *N*11–Fe2–*N*12 | 145.8 | 158.8 | 158.7 |
| | | | |
| $\alpha$ {Fe1} | 73.6 | 73.5 | 79.4 |
| $\alpha$ {Fe2} | 73.5 | 79.3 | 79.4 |
| $\theta$ {Fe1} | 86.0 | 83.3 | 88.9 |
| $\theta$ {Fe2} | 82.4 | 89.7 | 88.9 |

[a]HS = high-spin, LS = low-spin. $\alpha$ is the average ligand bite angle in the molecule; see page S20 for definitions of $\phi$ and $\theta$. [b]Cr1 in this molecule minimized to a Jahn-Teller elongated [4+2] coordination geometry, but Cr2 has a more regular pseudo-octahedral geometry. [c]High-spin Cr1 in this molecule minimized to a pseudo-five-coordinate [5+1] coordination geometry, with one very long Cr–N interaction.

**Table S14** continued.

| | $[Cr_2(\mu\text{-}L^3)_2]^0$ | | |
|---|---|---|---|
| | HS–HS[b] | HS–LS[c] | LS–LS |
| Cr1–*N*1 | 2.077 | 1.973 | 1.968 |
| Cr1–*N*2 | 2.124 | 2.091 | 2.007 |
| Cr1–*N*3 | 2.226 | 2.185 | 2.037 |
| Cr1–*N*4 | 2.169 | 2.173 | 1.962 |
| Cr1–*N*5 | 2.434 | 2.209 | 2.011 |
| Cr1–*N*6 | 2.508 | 2.627 | 2.034 |
| Cr2–*N*7 | 1.973 | 1.961 | 1.961 |
| Cr2–*N*8 | 2.080 | 2.009 | 2.011 |
| Cr2–*N*9 | 2.058 | 2.036 | 2.036 |
| Cr2–*N*10 | 1.976 | 1.964 | 1.969 |
| Cr2–*N*11 | 2.076 | 2.010 | 2.006 |
| Cr2–*N*12 | 2.062 | 2.041 | 2.033 |
| Cr1···Cr2 | 7.684 | 7.667 | 7.650 |
| | | | |
| *N*1–Cr1–*N*2 | 75.5 | 76.9 | 77.3 |
| *N*1–Cr1–*N*3 | 74.8 | 75.9 | 76.6 |
| *N*1–Cr1–*N*4 ($\phi$ {Cr1}) | 171.8 | 178.7 | 167.5 |
| *N*1–Cr1–*N*5 | 100.0 | 105.3 | 93.0 |
| *N*1–Cr1–*N*6 | 117.2 | 112.3 | 113.0 |
| *N*2–Cr1–*N*3 | 149.9 | 150.3 | 153.8 |
| *N*2–Cr1–*N*4 | 104.2 | 102.3 | 95.3 |
| *N*2–Cr1–*N*5 | 103.4 | 108.9 | 93.6 |
| *N*2–Cr1–*N*6 | 92.2 | 85.7 | 90.0 |
| *N*3–Cr1–*N*4 | 105.9 | 105.1 | 110.8 |
| *N*3–Cr1–*N*5 | 86.6 | 89.6 | 90.1 |
| *N*3–Cr1–*N*6 | 96.9 | 93.9 | 98.1 |
| *N*4–Cr1–*N*5 | 72.0 | 73.9 | 77.3 |
| *N*4–Cr1–*N*6 | 71.0 | 68.6 | 76.6 |
| *N*5–Cr1–*N*6 | 142.3 | 141.9 | 153.9 |
| *N*7–Cr2–*N*8 | 76.9 | 77.3 | 77.3 |
| *N*7–Cr2–*N*9 | 76.8 | 76.7 | 76.7 |
| *N*7–Cr2–*N*10 ($\phi$ {Cr2}) | 169.5 | 168.9 | 167.6 |
| *N*7–Cr2–*N*11 | 97.0 | 96.0 | 95.1 |
| *N*7–Cr2–*N*12 | 109.2 | 109.9 | 111.1 |
| *N*8–Cr2–*N*9 | 153.7 | 154.0 | 153.9 |
| *N*8–Cr2–*N*10 | 94.9 | 94.0 | 93.3 |
| *N*8–Cr2–*N*11 | 93.1 | 93.1 | 93.6 |
| *N*8–Cr2–*N*12 | 90.5 | 90.7 | 89.9 |
| *N*9–Cr2–*N*10 | 111.4 | 111.9 | 112.7 |
| *N*9–Cr2–*N*11 | 91.2 | 90.6 | 90.0 |
| *N*9–Cr2–*N*12 | 97.0 | 97.1 | 98.2 |
| *N*10–Cr2–*N*11 | 76.8 | 77.3 | 77.2 |
| *N*10–Cr2–*N*12 | 77.0 | 76.8 | 76.6 |
| *N*11–Cr2–*N*12 | 153.7 | 154.0 | 153.8 |
| | | | |
| $\alpha$ {Cr1} | 73.2 | 73.8 | 77.0 |
| $\alpha$ {Cr2} | 76.9 | 77.0 | 77.0 |
| $\theta$ {Cr1} | 78.2 | 78.5 | 86.5 |
| $\theta$ {Cr2} | 88.9 | 87.8 | 86.4 |

[a]HS = high-spin, LS = low-spin. $\alpha$ is the average ligand bite angle in the molecule; see page S20 for definitions of $\phi$ and $\theta$. [b]Cr1 in this molecule minimized to a Jahn-Teller elongated [4+2] coordination geometry, but Cr2 has a more regular pseudo-octahedral geometry. [c]High-spin Cr1 in this molecule minimized to a pseudo-five-coordinate [5+1] coordination geometry, with one very long Cr–N interaction.

**Table S15** Computed atomic coordinates for the DFT-minimized molecules in this work.

$[Fe(L^1)]^{2+}$, low-spin

| | | | | |
|---|---|---|---|---|
| 1 | Fe | 0.0000000 | 0.0000001 | -0.4715781 |
| 2 | N | -1.9240068 | -0.1326615 | -0.7255458 |
| 3 | C | -2.7562769 | 0.7978486 | -0.1603596 |
| 4 | C | -4.1196071 | 0.8424512 | -0.5027067 |
| 5 | C | -4.6118116 | -0.0871718 | -1.4370224 |
| 6 | H | -5.6757050 | -0.0788971 | -1.7119737 |
| 7 | C | -3.7475566 | -1.0186464 | -2.0406089 |
| 8 | H | -4.1149147 | -1.7296408 | -2.7911190 |
| 9 | C | -2.3962019 | -0.9870743 | -1.6628621 |
| 10 | C | -2.0355246 | 1.6016465 | 0.8186951 |
| 11 | N | -0.7166487 | 1.2669337 | 0.9552927 |
| 12 | N | -0.2679825 | 1.9318404 | 2.0443722 |
| 13 | C | -1.2562667 | 2.7224562 | 2.5716999 |
| 14 | H | -1.0755667 | 3.3294393 | 3.4655323 |
| 15 | C | -2.4063573 | 2.5481301 | 1.8065648 |
| 16 | H | -3.3782997 | 3.0258150 | 1.9599928 |
| 17 | N | -1.3667757 | -1.7695651 | -2.2090901 |
| 18 | N | -0.0855620 | -1.4454583 | -1.8193415 |
| 19 | C | 0.7232256 | -2.2786277 | -2.4977669 |
| 20 | H | 1.8095062 | -2.2298065 | -2.3726355 |
| 21 | C | -0.0329095 | -3.1485973 | -3.3252274 |
| 22 | H | 0.3474382 | -3.9326652 | -3.9867791 |
| 23 | C | -1.3606380 | -2.7994793 | -3.1207336 |
| 24 | H | -2.2869116 | -3.2022737 | -3.5416313 |
| 25 | N | 1.9240069 | 0.1326615 | -0.7255457 |
| 26 | C | 2.3962020 | 0.9870742 | -1.6628622 |
| 27 | C | 3.7475567 | 1.0186461 | -2.0406091 |
| 28 | H | 4.1149147 | 1.7296403 | -2.7911194 |
| 29 | C | 4.6118115 | 0.0871713 | -1.4370227 |
| 30 | H | 5.6757049 | 0.0788963 | -1.7119742 |
| 31 | C | 4.1196070 | -0.8424516 | -0.5027069 |
| 32 | C | 2.7562769 | -0.7978488 | -0.1603597 |
| 33 | N | 1.3667759 | 1.7695651 | -2.2090899 |
| 34 | N | 0.0855621 | 1.4454585 | -1.8193414 |
| 35 | C | -0.7232254 | 2.2786279 | -2.4977668 |
| 36 | H | -1.8095060 | 2.2298067 | -2.3726354 |
| 37 | C | 0.0329097 | 3.1485977 | -3.3252270 |
| 38 | H | -0.3474380 | 3.9326657 | -3.9867784 |
| 39 | C | 1.3606382 | 2.7994796 | -3.1207333 |
| 40 | H | 2.2869117 | 3.2022739 | -3.5416311 |
| 41 | C | 2.0355245 | -1.6016467 | 0.8186950 |
| 42 | N | 0.7166486 | -1.2669337 | 0.9552926 |
| 43 | N | 0.2679824 | -1.9318404 | 2.0443724 |
| 44 | C | 1.2562666 | -2.7224562 | 2.5717003 |
| 45 | H | 1.0755666 | -3.3294390 | 3.4655328 |
| 46 | C | 2.4063572 | -2.5481302 | 1.8065650 |
| 47 | H | 3.3782996 | -3.0258152 | 1.9599929 |
| 48 | H | -4.7826264 | 1.5872223 | -0.0435707 |
| 49 | H | 4.7826262 | -1.5872228 | -0.0435708 |
| 50 | C | -0.9323487 | -1.4514040 | 2.7354747 |
| 51 | H | -1.5647892 | -0.9982471 | 1.9541082 |
| 52 | H | -1.4766601 | -2.3305468 | 3.1298844 |
| 53 | C | 0.9323487 | 1.4514042 | 2.7354746 |
| 54 | H | 1.4766600 | 2.3305471 | 3.1298842 |
| 55 | H | 1.5647892 | 0.9982472 | 1.9541082 |
| 56 | C | -0.6334076 | -0.4514012 | 3.8947568 |
| 57 | H | -0.5682947 | -1.0292246 | 4.8383623 |
| 58 | H | -1.5420744 | 0.1746777 | 4.0100266 |

| | | | | |
|---|---|---|---|---|
| 59 | C | 0.6334076 | 0.4514014 | 3.8947567 |
| 60 | H | 1.5420743 | -0.1746775 | 4.0100265 |
| 61 | H | 0.5682948 | 1.0292249 | 4.8383622 |

$[Fe(L^1)]^{2+}$, high-spin

| | | | | |
|---|---|---|---|---|
| 1 | Fe | -0.0000001 | 0.0000001 | 0.3453636 |
| 2 | N | -0.1176735 | -2.1179235 | 0.8573418 |
| 3 | C | 0.6655255 | -3.0163170 | 0.2005641 |
| 4 | C | 0.7129942 | -4.3645937 | 0.6011033 |
| 5 | C | -0.0780448 | -4.7669645 | 1.6923360 |
| 6 | H | -0.0598112 | -5.8132589 | 2.0285069 |
| 7 | C | -0.9051517 | -3.8420477 | 2.3509614 |
| 8 | H | -1.5357394 | -4.1528696 | 3.1929674 |
| 9 | C | -0.8896945 | -2.5158477 | 1.8814715 |
| 10 | C | 1.3682121 | -2.4347675 | -0.9498866 |
| 11 | N | 1.1124046 | -1.1245934 | -1.2245034 |
| 12 | N | 1.7923721 | -0.8374491 | -2.3555020 |
| 13 | C | 2.4884776 | -1.9241626 | -2.8041206 |
| 14 | H | 3.1035740 | -1.8721142 | -3.7091797 |
| 15 | C | 2.2433327 | -2.9783666 | -1.9224814 |
| 16 | H | 2.6458172 | -3.9933659 | -1.9867815 |
| 17 | N | -1.6891525 | -1.4777783 | 2.4219087 |
| 18 | N | -1.5778545 | -0.2252411 | 1.8805970 |
| 19 | C | -2.4714016 | 0.5329462 | 2.5370775 |
| 20 | H | -2.5779521 | 1.5947105 | 2.2879587 |
| 21 | C | -3.1712319 | -0.2263636 | 3.5084751 |
| 22 | H | -3.9563204 | 0.1140302 | 4.1904183 |
| 23 | C | -2.6450552 | -1.5085492 | 3.4099789 |
| 24 | H | -2.8828166 | -2.4235517 | 3.9606977 |
| 25 | N | 0.1176731 | 2.1179234 | 0.8573422 |
| 26 | C | 0.8896942 | 2.5158477 | 1.8814718 |
| 27 | C | 0.9051514 | 3.8420478 | 2.3509617 |
| 28 | H | 1.5357392 | 4.1528696 | 3.1929677 |
| 29 | C | 0.0780445 | 4.7669646 | 1.6923364 |
| 30 | H | 0.0598109 | 5.8132590 | 2.0285073 |
| 31 | C | -0.7129942 | 4.3645938 | 0.6011035 |
| 32 | C | -0.6655257 | 3.0163171 | 0.2005643 |
| 33 | N | 1.6891525 | 1.4777783 | 2.4219088 |
| 34 | N | 1.5778545 | 0.2252410 | 1.8805967 |
| 35 | C | 2.4714017 | -0.5329465 | 2.5370769 |
| 36 | H | 2.5779524 | -1.5947107 | 2.2879581 |
| 37 | C | 3.1712321 | 0.2263636 | 3.5084747 |
| 38 | H | 3.9563207 | -0.1140304 | 4.1904177 |
| 39 | C | 2.6450554 | 1.5085493 | 3.4099789 |
| 40 | H | 2.8828166 | 2.4235517 | 3.9606978 |
| 41 | C | -1.3682120 | 2.4347678 | -0.9498866 |
| 42 | N | -1.1124048 | 1.1245935 | -1.2245032 |
| 43 | N | -1.7923720 | 0.8374493 | -2.3555020 |
| 44 | C | -2.4884770 | 1.9241629 | -2.8041208 |
| 45 | H | -3.1035735 | 1.8721146 | -3.7091799 |
| 46 | C | -2.2433322 | 2.9783670 | -1.9224817 |
| 47 | H | -2.6458160 | 3.9933665 | -1.9867823 |
| 48 | H | 1.3441907 | -5.0870549 | 0.0676855 |
| 49 | H | -1.3441904 | 5.0870551 | 0.0676855 |
| 50 | C | -1.6056120 | -0.4617900 | -3.0165084 |
| 51 | H | -1.2727631 | -1.1490612 | -2.2198072 |
| 52 | H | -2.5990287 | -0.8131751 | -3.3558023 |
| 53 | C | 1.6056117 | 0.4617900 | -3.0165088 |
| 54 | H | 2.5990282 | 0.8131754 | -3.3558028 |
| 55 | H | 1.2727627 | 1.1490612 | -2.2198077 |
| 56 | C | -0.6194491 | -0.4620583 | -4.2143952 |

| | | | | |
|---|---|---|---|---|
| 57 | H | -1.1841437 | -0.2159761 | -5.1363313 |
| 58 | H | -0.3063497 | -1.5183374 | -4.3474299 |
| 59 | C | 0.6194486 | 0.4620576 | -4.2143956 |
| 60 | H | 0.3063494 | 1.5183367 | -4.3474309 |
| 61 | H | 1.1841432 | 0.2159748 | -5.1363315 |

$[Fe_2(\mu\text{-}L^1)_2]^{4+}$ conformation (a), low-spin

| | | | | |
|---|---|---|---|---|
| 1 | Fe | 0.011734 | 0.004533 | 4.504167 |
| 2 | Fe | 0.000982 | -0.001209 | -4.478566 |
| 3 | C | -2.605667 | -0.795246 | -1.919525 |
| 4 | C | -2.444265 | 0.091904 | -0.663225 |
| 5 | C | -2.374326 | -0.661776 | 0.682337 |
| 6 | C | -2.720172 | 0.166919 | 1.941954 |
| 7 | H | -2.738377 | -0.161150 | -2.813032 |
| 8 | H | -3.514792 | -1.422079 | -1.828267 |
| 9 | H | -3.334898 | 0.756596 | -0.668300 |
| 10 | H | -1.563265 | 0.754248 | -0.789611 |
| 11 | H | -1.380851 | -1.137499 | 0.812757 |
| 12 | H | -3.115659 | -1.489362 | 0.678564 |
| 13 | H | -3.747533 | 0.573803 | 1.858666 |
| 14 | H | -2.702271 | -0.485797 | 2.831545 |
| 15 | N | -1.493871 | -1.709983 | -2.215151 |
| 16 | N | -0.491401 | -1.407770 | -3.080869 |
| 17 | C | 0.348183 | -2.490221 | -3.071859 |
| 18 | C | -0.138337 | -3.491540 | -2.196611 |
| 19 | C | -1.311953 | -2.957750 | -1.675110 |
| 20 | H | -2.045110 | -3.381922 | -0.982062 |
| 21 | H | 0.284826 | -4.480656 | -1.998241 |
| 22 | C | 1.497491 | -2.402909 | -3.967325 |
| 23 | C | 2.497038 | -3.363515 | -4.204312 |
| 24 | C | 3.493707 | -3.080143 | -5.158002 |
| 25 | C | 3.474022 | -1.867739 | -5.872516 |
| 26 | C | 2.437387 | -0.964145 | -5.587749 |
| 27 | N | 1.497844 | -1.214314 | -4.643658 |
| 28 | H | 4.230642 | -1.648817 | -6.636344 |
| 29 | H | 4.283336 | -3.816275 | -5.363473 |
| 30 | H | 2.488497 | -4.322054 | -3.669000 |
| 31 | N | 2.206458 | 0.263090 | -6.227968 |
| 32 | N | 1.058594 | 0.934880 | -5.858863 |
| 33 | C | 1.030275 | 2.049182 | -6.610247 |
| 34 | C | 2.159781 | 2.105219 | -7.468546 |
| 35 | C | 2.888588 | 0.953923 | -7.204521 |
| 36 | H | 3.825538 | 0.580968 | -7.631919 |
| 37 | H | 2.406662 | 2.888719 | -8.191998 |
| 38 | H | 0.208685 | 2.767119 | -6.514576 |
| 39 | N | -1.496922 | 1.210181 | -4.642144 |
| 40 | C | -2.438734 | 0.957652 | -5.583170 |
| 41 | C | -3.479307 | 1.857970 | -5.863693 |
| 42 | C | -3.500699 | 3.069691 | -5.148150 |
| 43 | C | -2.502098 | 3.355490 | -4.197373 |
| 44 | C | -1.498748 | 2.397827 | -3.964128 |
| 45 | H | -2.495376 | 4.313476 | -3.660969 |
| 46 | H | -4.293583 | 3.803305 | -5.350279 |
| 47 | H | -4.237814 | 1.637024 | -6.624977 |
| 48 | N | -2.206171 | -0.268449 | -6.224938 |
| 49 | N | -1.056374 | -0.937897 | -5.858262 |
| 50 | C | -1.026642 | -2.050925 | -6.611373 |
| 51 | C | -2.157192 | -2.108335 | -7.468337 |
| 52 | C | -2.888207 | -0.959400 | -7.201522 |
| 53 | H | -0.203366 | -2.766907 | -6.518091 |
| 54 | H | -2.403149 | -2.891239 | -8.192689 |

| | | | | |
|---|---|---|---|---|
| 55 | H | -3.826453 | -0.588025 | -7.627101 |
| 56 | C | -0.348096 | 2.486876 | -3.070634 |
| 57 | C | 0.138058 | 3.488234 | -2.195702 |
| 58 | N | 0.493153 | 1.405800 | -3.081364 |
| 59 | C | 1.313373 | 2.956243 | -1.676450 |
| 60 | N | 1.496536 | 1.709208 | -2.217368 |
| 61 | H | -0.286513 | 4.476270 | -1.996038 |
| 62 | H | 2.046911 | 3.381223 | -0.984464 |
| 63 | C | 2.610040 | 0.796125 | -1.923930 |
| 64 | C | 2.451937 | -0.092012 | -0.668382 |
| 65 | C | 2.386402 | 0.660169 | 0.678008 |
| 66 | C | 2.733707 | -0.170524 | 1.935320 |
| 67 | H | 2.721789 | 0.480595 | 2.825114 |
| 68 | H | 3.758315 | -0.580924 | 1.847012 |
| 69 | H | 1.394185 | 1.137526 | 0.811605 |
| 70 | H | 3.129278 | 1.486212 | 0.673275 |
| 71 | H | 1.570305 | -0.753957 | -0.792923 |
| 72 | H | 3.342254 | -0.756851 | -0.676576 |
| 73 | H | 2.742405 | 0.163798 | -2.817992 |
| 74 | H | 3.517809 | 1.423920 | -1.832924 |
| 75 | N | -1.842153 | 1.308563 | 2.235204 |
| 76 | N | -0.799209 | 1.246175 | 3.102941 |
| 77 | C | -0.217720 | 2.487049 | 3.076381 |
| 78 | C | -0.911142 | 3.344168 | 2.187911 |
| 79 | C | -1.938996 | 2.558385 | 1.678660 |
| 80 | H | -2.747912 | 2.800531 | 0.982635 |
| 81 | H | -0.716556 | 4.399117 | 1.975137 |
| 82 | N | 1.852706 | -1.308760 | 2.231523 |
| 83 | N | 0.811687 | -1.241038 | 3.100716 |
| 84 | C | 0.224848 | -2.479472 | 3.076870 |
| 85 | C | 1.942954 | -2.559275 | 1.675847 |
| 86 | C | 0.912698 | -3.340108 | 2.187805 |
| 87 | H | 2.749444 | -2.805536 | 0.978621 |
| 88 | H | 0.713309 | -4.394328 | 1.976591 |
| 89 | N | -1.173291 | -1.506795 | 4.663583 |
| 90 | C | -0.920459 | -2.654624 | 3.965358 |
| 91 | C | -1.705988 | -3.803357 | 4.166948 |
| 92 | C | -2.156938 | -1.473032 | 5.596582 |
| 93 | C | 0.927924 | 2.668642 | 3.962942 |
| 94 | N | 1.192583 | 1.520558 | 4.656575 |
| 95 | C | 2.174424 | 1.494406 | 5.591673 |
| 96 | C | 2.992484 | 2.605916 | 5.849553 |
| 97 | C | 1.700909 | 3.825370 | 4.169806 |
| 98 | C | 2.744411 | 3.779841 | 5.114442 |
| 99 | C | -2.987435 | -2.576093 | 5.848835 |
| 100 | C | -2.750452 | -3.749688 | 5.110040 |
| 101 | H | 1.483629 | 4.749669 | 3.618506 |
| 102 | H | 3.360407 | 4.671761 | 5.297553 |
| 103 | H | 3.785585 | 2.570447 | 6.607614 |
| 104 | H | -3.782218 | -2.534351 | 6.604662 |
| 105 | H | -3.376939 | -4.634773 | 5.288219 |
| 106 | H | -1.498850 | -4.727269 | 3.611614 |
| 107 | N | -2.197758 | -0.232297 | 6.251553 |
| 108 | N | -1.225688 | 0.677156 | 5.887860 |
| 109 | C | -1.447347 | 1.769436 | 6.638925 |
| 110 | C | -2.564345 | 1.572700 | 7.493464 |
| 111 | C | -3.020668 | 0.289763 | 7.224936 |
| 112 | H | -2.980438 | 2.280632 | 8.217291 |
| 113 | H | -0.805144 | 2.652151 | 6.546152 |
| 114 | H | -3.854026 | -0.282103 | 7.646119 |
| 115 | N | 2.225862 | 0.252782 | 6.244434 |
| 116 | N | 1.261041 | -0.664598 | 5.880002 |

| | | | | |
|---|---|---|---|---|
| 117 | C | 1.495818 | -1.758217 | 6.625390 |
| 118 | C | 2.613622 | -1.553904 | 7.477795 |
| 119 | C | 3.056722 | -0.265498 | 7.213168 |
| 120 | H | 3.885909 | 0.313189 | 7.634423 |
| 121 | H | 3.036848 | -2.262281 | 8.197048 |
| 122 | H | 0.857524 | -2.644836 | 6.532886 |

$[Fe_2(\mu\text{-}L^1)_2]^{4+}$ conformation (a), mixed-spin

| | | | | |
|---|---|---|---|---|
| 1 | Fe | 0.0001145 | 0.0001303 | 4.6052896 |
| 2 | Fe | 0.0005091 | 0.0002666 | -4.5853518 |
| 3 | C | -2.4894889 | -1.0304943 | -1.8112276 |
| 4 | C | -2.3699051 | -0.1284539 | -0.5626211 |
| 5 | C | -2.2987060 | -0.8625062 | 0.7930272 |
| 6 | C | -2.7073449 | -0.0318608 | 2.0326007 |
| 7 | H | -2.6343683 | -0.4042465 | -2.7100854 |
| 8 | H | -3.3822518 | -1.6811193 | -1.7250925 |
| 9 | H | -3.2806296 | 0.5076145 | -0.5889499 |
| 10 | H | -1.5090082 | 0.5603506 | -0.6843538 |
| 11 | H | -1.2900009 | -1.2951479 | 0.9502264 |
| 12 | H | -3.0052458 | -1.7198115 | 0.7844251 |
| 13 | H | -3.7565196 | 0.3094843 | 1.9294657 |
| 14 | H | -2.6593352 | -0.6634421 | 2.9358426 |
| 15 | N | -1.3490009 | -1.9130435 | -2.1081578 |
| 16 | N | -0.4135250 | -1.5878155 | -3.0338710 |
| 17 | C | 0.4164988 | -2.6656877 | -3.1234931 |
| 18 | C | 0.0004052 | -3.6873408 | -2.2318970 |
| 19 | C | -1.1345568 | -3.1714274 | -1.6124991 |
| 20 | H | -1.8207986 | -3.6206211 | -0.8873134 |
| 21 | H | 0.4326567 | -4.6818292 | -2.0883625 |
| 22 | C | 1.5037984 | -2.6254721 | -4.1138767 |
| 23 | C | 2.4304939 | -3.6586714 | -4.3432650 |
| 24 | C | 3.3883712 | -3.4891459 | -5.3608691 |
| 25 | C | 3.4181322 | -2.3067845 | -6.1178717 |
| 26 | C | 2.4624182 | -1.3189139 | -5.8135198 |
| 27 | N | 1.5377266 | -1.4780452 | -4.8469136 |
| 28 | H | 4.1572929 | -2.1706895 | -6.9168803 |
| 29 | H | 4.1156358 | -4.2861688 | -5.5716235 |
| 30 | H | 2.4020061 | -4.5839652 | -3.7535282 |
| 31 | N | 2.3739155 | -0.0762590 | -6.4839898 |
| 32 | N | 1.3873603 | 0.7979443 | -6.1047171 |
| 33 | C | 1.5533706 | 1.8846894 | -6.8778284 |
| 34 | C | 2.6501516 | 1.7237575 | -7.7621353 |
| 35 | C | 3.1524235 | 0.4587446 | -7.4864262 |
| 36 | H | 3.9938190 | -0.0862413 | -7.9257714 |
| 37 | H | 3.0242889 | 2.4337298 | -8.5067230 |
| 38 | H | 0.8796188 | 2.7432223 | -6.7752644 |
| 39 | N | -1.5380192 | 1.4775054 | -4.8467349 |
| 40 | C | -2.4619173 | 1.3185373 | -5.8140974 |
| 41 | C | -3.4188471 | 2.3054176 | -6.1176684 |
| 42 | C | -3.3913903 | 3.4867022 | -5.3588572 |
| 43 | C | -2.4344755 | 3.6560517 | -4.3403688 |
| 44 | C | -1.5063689 | 2.6238319 | -4.1119356 |
| 45 | H | -2.4077493 | 4.5804666 | -3.7491641 |
| 46 | H | -4.1196546 | 4.2829716 | -5.5689568 |
| 47 | H | -4.1571712 | 2.1695177 | -6.9174925 |
| 48 | N | -2.3709881 | 0.0770323 | -6.4864237 |
| 49 | N | -1.3825789 | -0.7955078 | -6.1083510 |
| 50 | C | -1.5463804 | -1.8818562 | -6.8824946 |
| 51 | C | -2.6436169 | -1.7221533 | -7.7665174 |
| 52 | C | -3.1484814 | -0.4584619 | -7.4893505 |
| 53 | H | -0.8707709 | -2.7390399 | -6.7809491 |

| | | | | |
|---|---|---|---|---|
| 54 | H | -3.0163576 | -2.4320337 | -8.5118924 |
| 55 | H | -3.9910640 | 0.0852332 | -7.9280267 |
| 56 | C | -0.4196441 | 2.6641998 | -3.1210032 |
| 57 | C | -0.0043794 | 3.6855842 | -2.2287160 |
| 58 | N | 0.4112477 | 1.5869098 | -3.0320351 |
| 59 | C | 1.1310481 | 3.1702370 | -1.6097417 |
| 60 | N | 1.3466021 | 1.9123633 | -2.1062707 |
| 61 | H | -0.4374568 | 4.6796298 | -2.0845984 |
| 62 | H | 1.8169921 | 3.6195245 | -0.8843365 |
| 63 | C | 2.4879934 | 1.0307125 | -1.8101577 |
| 64 | C | 2.3700503 | 0.1285457 | -0.5614997 |
| 65 | C | 2.2991449 | 0.8624838 | 0.7941922 |
| 66 | C | 2.7082055 | 0.0317860 | 2.0335850 |
| 67 | H | 2.6603493 | 0.6633044 | 2.9368780 |
| 68 | H | 3.7573588 | -0.3095300 | 1.9301880 |
| 69 | H | 1.2903886 | 1.2948987 | 0.9516622 |
| 70 | H | 3.0055409 | 1.7199091 | 0.7854438 |
| 71 | H | 1.5095490 | -0.5608579 | -0.6826668 |
| 72 | H | 3.2812232 | -0.5068436 | -0.5884692 |
| 73 | H | 2.6327625 | 0.4045614 | -2.7091065 |
| 74 | H | 3.3803410 | 1.6820146 | -1.7247166 |
| 75 | N | -1.9044463 | 1.1672148 | 2.3125564 |
| 76 | N | -0.8789793 | 1.1866444 | 3.2031149 |
| 77 | C | -0.3796402 | 2.4620790 | 3.1709507 |
| 78 | C | -1.1103810 | 3.2601176 | 2.2578102 |
| 79 | C | -2.0731722 | 2.4017033 | 1.7381552 |
| 80 | H | -2.8811880 | 2.5829013 | 1.0228681 |
| 81 | H | -0.9805962 | 4.3224422 | 2.0324622 |
| 82 | N | 1.9053779 | -1.1673175 | 2.3135664 |
| 83 | N | 0.8797177 | -1.1866878 | 3.2038660 |
| 84 | C | 0.3802414 | -2.4620630 | 3.1715501 |
| 85 | C | 2.0741618 | -2.4018155 | 1.7392055 |
| 86 | C | 1.1111898 | -3.2601639 | 2.2586282 |
| 87 | H | 2.8823474 | -2.5830771 | 1.0241238 |
| 88 | H | 0.9813695 | -4.3224639 | 2.0331843 |
| 89 | N | -1.0800236 | -1.5932508 | 4.7625568 |
| 90 | C | -0.7381964 | -2.7233556 | 4.0724520 |
| 91 | C | -1.4209373 | -3.9324447 | 4.2950821 |
| 92 | C | -2.0498055 | -1.6341558 | 5.7096066 |
| 93 | C | 0.7384677 | 2.7235686 | 4.0721990 |
| 94 | N | 1.0801604 | 1.5935891 | 4.7625716 |
| 95 | C | 2.0495121 | 1.6347686 | 5.7100532 |
| 96 | C | 2.7785893 | 2.8026580 | 5.9850108 |
| 97 | C | 1.4209969 | 3.9327614 | 4.2949074 |
| 98 | C | 2.4522908 | 3.9593932 | 5.2533025 |
| 99 | C | -2.7791107 | -2.8019289 | 5.9844524 |
| 100 | C | -2.4526120 | -3.9588174 | 5.2530747 |
| 101 | H | 1.1420408 | 4.8417558 | 3.7460566 |
| 102 | H | 2.9967492 | 4.8939481 | 5.4494902 |
| 103 | H | 3.5624367 | 2.8216456 | 6.7531278 |
| 104 | H | -3.5632895 | -2.8207157 | 6.7522362 |
| 105 | H | -2.9972335 | -4.8932896 | 5.4492043 |
| 106 | H | -1.1418571 | -4.8415508 | 3.7464796 |
| 107 | N | -2.1839926 | -0.3968510 | 6.3589590 |
| 108 | N | -1.2856218 | 0.5822110 | 5.9882464 |
| 109 | C | -1.5890937 | 1.6581136 | 6.7346543 |
| 110 | C | -2.6849884 | 1.3803170 | 7.5936943 |
| 111 | C | -3.0417307 | 0.0648683 | 7.3324348 |
| 112 | H | -3.1531351 | 2.0578826 | 8.3148350 |
| 113 | H | -1.0177515 | 2.5872431 | 6.6350357 |
| 114 | H | -3.8279356 | -0.5672703 | 7.7577282 |
| 115 | N | 2.1835015 | 0.3976133 | 6.3597240 |

| | | | | |
|---|---|---|---|---|
| 116 | N | 1.2852917 | -0.5815567 | 5.9888997 |
| 117 | C | 1.5884891 | -1.6572688 | 6.7356914 |
| 118 | C | 2.6840482 | -1.3792444 | 7.5950872 |
| 119 | C | 3.0408725 | -0.0638560 | 7.3336435 |
| 120 | H | 3.8269099 | 0.5683976 | 7.7590759 |
| 121 | H | 3.1519344 | -2.0566315 | 8.3165649 |
| 122 | H | 1.0172134 | -2.5864385 | 6.6360721 |

$[Fe_2(\mu\text{-}L^1)_2]^{4+}$ conformation (a), high-spin

| | | | | |
|---|---|---|---|---|
| 1 | Fe | -0.000452 | -0.000226 | 4.711878 |
| 2 | Fe | -0.000116 | 0.000174 | -4.698937 |
| 3 | C | -2.524896 | -0.832823 | -1.895734 |
| 4 | C | -2.311487 | 0.062205 | -0.655480 |
| 5 | C | -2.248400 | -0.666250 | 0.704135 |
| 6 | C | -2.669222 | 0.175539 | 1.930258 |
| 7 | H | -2.641555 | -0.199990 | -2.794093 |
| 8 | H | -3.462573 | -1.412752 | -1.786073 |
| 9 | H | -3.182366 | 0.752347 | -0.662111 |
| 10 | H | -1.416908 | 0.701123 | -0.801358 |
| 11 | H | -1.241569 | -1.099925 | 0.874580 |
| 12 | H | -2.955722 | -1.522699 | 0.697755 |
| 13 | H | -3.717817 | 0.514582 | 1.812483 |
| 14 | H | -2.633463 | -0.446794 | 2.843180 |
| 15 | N | -1.466085 | -1.805581 | -2.212756 |
| 16 | N | -0.508361 | -1.548958 | -3.136799 |
| 17 | C | 0.235007 | -2.687598 | -3.233440 |
| 18 | C | -0.256936 | -3.677656 | -2.344575 |
| 19 | C | -1.349074 | -3.079148 | -1.722646 |
| 20 | H | -2.064207 | -3.476859 | -0.995191 |
| 21 | H | 0.098774 | -4.702554 | -2.204052 |
| 22 | C | 1.319212 | -2.724972 | -4.226769 |
| 23 | C | 2.179256 | -3.815782 | -4.448501 |
| 24 | C | 3.148547 | -3.712164 | -5.463379 |
| 25 | C | 3.255661 | -2.537290 | -6.225074 |
| 26 | C | 2.362281 | -1.490423 | -5.929885 |
| 27 | N | 1.424593 | -1.586620 | -4.967519 |
| 28 | H | 4.005868 | -2.450265 | -7.020333 |
| 29 | H | 3.825394 | -4.554102 | -5.667302 |
| 30 | H | 2.091889 | -4.733860 | -3.853152 |
| 31 | N | 2.356971 | -0.246681 | -6.604144 |
| 32 | N | 1.436350 | 0.694340 | -6.220760 |
| 33 | C | 1.670777 | 1.765028 | -6.998354 |
| 34 | C | 2.746523 | 1.525644 | -7.890978 |
| 35 | C | 3.162766 | 0.230054 | -7.614357 |
| 36 | H | 3.960274 | -0.374177 | -8.058545 |
| 37 | H | 3.162972 | 2.205816 | -8.640872 |
| 38 | H | 1.057944 | 2.668208 | -6.893026 |
| 39 | N | -1.423777 | 1.588116 | -4.966629 |
| 40 | C | -2.360969 | 1.493255 | -5.929730 |
| 41 | C | -3.253144 | 2.541226 | -6.225106 |
| 42 | C | -3.145268 | 3.715409 | -5.462568 |
| 43 | C | -2.176489 | 3.817393 | -4.446688 |
| 44 | C | -1.317632 | 2.725853 | -4.225143 |
| 45 | H | -2.088587 | 4.734897 | -3.850676 |
| 46 | H | -3.821130 | 4.558038 | -5.666533 |
| 47 | H | -4.003002 | 2.455572 | -7.021143 |
| 48 | N | -2.356592 | 0.249786 | -6.604487 |
| 49 | N | -1.437202 | -0.692566 | -6.221064 |
| 50 | C | -1.672266 | -1.762518 | -6.999524 |
| 51 | C | -2.747245 | -1.521540 | -7.892599 |
| 52 | C | -3.162326 | -0.225696 | -7.615283 |

| | | | | |
|---|---|---|---|---|
| 53 | H | -1.060382 | -2.666231 | -6.894494 |
| 54 | H | -3.164071 | -2.200916 | -8.643096 |
| 55 | H | -3.959012 | 0.379295 | -8.059192 |
| 56 | C | -0.233965 | 2.686927 | -3.231182 |
| 57 | C | 0.258079 | 3.676039 | -2.341285 |
| 58 | N | 0.508661 | 1.547850 | -3.134977 |
| 59 | C | 1.349604 | 3.076563 | -1.719330 |
| 60 | N | 1.466025 | 1.803221 | -2.210183 |
| 61 | H | -0.097040 | 4.701057 | -2.200282 |
| 62 | H | 2.064680 | 3.473442 | -0.991349 |
| 63 | C | 2.524164 | 0.829749 | -1.893521 |
| 64 | C | 2.310642 | -0.065232 | -0.653473 |
| 65 | C | 2.247437 | 0.663141 | 0.706322 |
| 66 | C | 2.669694 | -0.178536 | 1.931998 |
| 67 | H | 2.635086 | 0.443832 | 2.844777 |
| 68 | H | 3.717911 | -0.517735 | 1.812990 |
| 69 | H | 1.240109 | 1.095895 | 0.877183 |
| 70 | H | 2.954084 | 1.520247 | 0.699935 |
| 71 | H | 1.415935 | -0.704115 | -0.799788 |
| 72 | H | 3.181525 | -0.755598 | -0.659996 |
| 73 | H | 2.639938 | 0.197069 | -2.792011 |
| 74 | H | 3.462347 | 1.408841 | -1.783971 |
| 75 | N | -1.870378 | 1.377417 | 2.219268 |
| 76 | N | -0.875761 | 1.369743 | 3.138906 |
| 77 | C | -0.420082 | 2.652118 | 3.210699 |
| 78 | C | -1.135923 | 3.483641 | 2.311849 |
| 79 | C | -2.059458 | 2.634426 | 1.708720 |
| 80 | H | -2.855700 | 2.841550 | 0.986454 |
| 81 | H | -1.031998 | 4.560456 | 2.152076 |
| 82 | N | 1.871042 | -1.380244 | 2.222104 |
| 83 | N | 0.876254 | -1.371918 | 3.141483 |
| 84 | C | 0.420989 | -2.654456 | 3.214733 |
| 85 | C | 2.060433 | -2.637665 | 1.712875 |
| 86 | C | 1.137255 | -3.486733 | 2.317062 |
| 87 | H | 2.856722 | -2.845290 | 0.990871 |
| 88 | H | 1.033721 | -4.563833 | 2.158473 |
| 89 | N | -1.009534 | -1.886714 | 4.944060 |
| 90 | C | -0.626357 | -2.961461 | 4.200221 |
| 91 | C | -1.192223 | -4.231290 | 4.416963 |
| 92 | C | -1.935916 | -2.028215 | 5.912122 |
| 93 | C | 0.626937 | 2.959921 | 4.196165 |
| 94 | N | 1.008892 | 1.886407 | 4.942155 |
| 95 | C | 1.934359 | 2.029238 | 5.911090 |
| 96 | C | 2.542343 | 3.264948 | 6.201001 |
| 97 | C | 1.193854 | 4.229023 | 4.410574 |
| 98 | C | 2.155196 | 4.371825 | 5.428619 |
| 99 | C | -2.543252 | -3.264311 | 6.203845 |
| 100 | C | -2.154296 | -4.372984 | 5.434391 |
| 101 | H | 0.885140 | 5.094031 | 3.809555 |
| 102 | H | 2.604068 | 5.354927 | 5.628248 |
| 103 | H | 3.286005 | 3.373334 | 7.000272 |
| 104 | H | -3.287538 | -3.371781 | 7.002609 |
| 105 | H | -2.602229 | -5.356715 | 5.635921 |
| 106 | H | -0.882014 | -5.097676 | 3.818194 |
| 107 | N | -2.229425 | -0.825550 | 6.597622 |
| 108 | N | -1.553973 | 0.311880 | 6.234361 |
| 109 | C | -2.041054 | 1.286617 | 7.021141 |
| 110 | C | -3.037312 | 0.788776 | 7.898325 |
| 111 | C | -3.132854 | -0.564726 | 7.603665 |
| 112 | H | -3.608874 | 1.341360 | 8.650872 |
| 113 | H | -1.656903 | 2.309500 | 6.933692 |
| 114 | H | -3.770002 | -1.343781 | 8.033270 |

| | | | | |
|---|---|---|---|---|
| 115 | N | 2.226691 | 0.827862 | 6.599004 |
| 116 | N | 1.551711 | -0.310324 | 6.236245 |
| 117 | C | 2.038424 | -1.284142 | 7.024440 |
| 118 | C | 3.033663 | -0.784530 | 7.902575 |
| 119 | C | 3.129037 | 0.568855 | 7.606690 |
| 120 | H | 3.765306 | 1.349058 | 8.036498 |
| 121 | H | 3.604547 | -1.335979 | 8.656490 |
| 122 | H | 1.654789 | -2.307539 | 6.937556 |

$[Fe_2(\mu\text{-}L^1)_2]^{4+}$ conformation (b), low-spin

| | | | | |
|---|---|---|---|---|
| 1 | Fe | 4.463430 | -0.362385 | -0.007911 |
| 2 | Fe | -4.449634 | 0.378081 | 0.011564 |
| 3 | N | -4.484131 | 2.145987 | 0.786662 |
| 4 | C | -5.350188 | 3.061635 | 0.286926 |
| 5 | C | -5.537979 | 4.319168 | 0.883241 |
| 6 | C | -4.804728 | 4.599658 | 2.050789 |
| 7 | C | -3.931928 | 3.638417 | 2.595745 |
| 8 | C | -3.794233 | 2.402690 | 1.939175 |
| 9 | N | -6.018724 | 2.568355 | -0.844698 |
| 10 | N | -5.738459 | 1.262869 | -1.194223 |
| 11 | C | -6.497727 | 1.002143 | -2.272338 |
| 12 | C | -7.272860 | 2.137420 | -2.627119 |
| 13 | C | -6.948442 | 3.117507 | -1.699770 |
| 14 | C | -3.005593 | 1.242657 | 2.343003 |
| 15 | N | -3.103675 | 0.147126 | 1.525892 |
| 16 | N | -2.318121 | -0.807293 | 2.088444 |
| 17 | C | -1.750681 | -0.348976 | 3.248841 |
| 18 | C | -2.160900 | 0.965813 | 3.444822 |
| 19 | C | -2.204702 | -2.170218 | 1.541488 |
| 20 | H | -2.730845 | -2.152181 | 0.572267 |
| 21 | H | -2.761263 | -2.855293 | 2.213223 |
| 22 | C | -0.759564 | -2.679446 | 1.373083 |
| 23 | H | -0.220146 | -2.624174 | 2.342334 |
| 24 | C | 0.047984 | -1.990265 | 0.257829 |
| 25 | H | 0.157725 | -0.905530 | 0.467143 |
| 26 | H | -0.518411 | -2.069357 | -0.693580 |
| 27 | C | 1.446567 | -2.618928 | 0.048897 |
| 28 | H | 2.133787 | -2.379190 | 0.879090 |
| 29 | H | 1.367854 | -3.723393 | -0.008423 |
| 30 | N | 3.067428 | -1.223070 | -1.228789 |
| 31 | N | 2.090662 | -2.165947 | -1.191784 |
| 32 | C | 1.677866 | -2.490517 | -2.459221 |
| 33 | C | 2.402608 | -1.722521 | -3.363704 |
| 34 | C | 3.258682 | -0.936664 | -2.554666 |
| 35 | N | 4.891994 | 0.481526 | -1.692800 |
| 36 | C | 4.289698 | 0.054782 | -2.844097 |
| 37 | C | 4.703010 | 0.546052 | -4.095106 |
| 38 | C | 5.761802 | 1.473278 | -4.139651 |
| 39 | C | 6.407844 | 1.876941 | -2.956673 |
| 40 | C | 5.942901 | 1.336138 | -1.747240 |
| 41 | N | 6.490189 | 1.556147 | -0.473271 |
| 42 | N | 5.939946 | 0.823189 | 0.557928 |
| 43 | C | 6.632249 | 1.164565 | 1.658438 |
| 44 | C | 7.634338 | 2.120195 | 1.345300 |
| 45 | C | 7.520721 | 2.348348 | -0.019062 |
| 46 | N | -4.741385 | -1.370524 | -0.756208 |
| 47 | C | -5.750869 | -2.135211 | -0.273026 |
| 48 | C | -6.107347 | -3.361693 | -0.856415 |
| 49 | C | -5.390270 | -3.771961 | -1.995807 |
| 50 | C | -4.363508 | -2.965205 | -2.523432 |
| 51 | C | -4.058769 | -1.753772 | -1.877121 |

| | | | | |
|---|---|---|---|---|
| 52 | N | -6.372189 | -1.525863 | 0.827664 |
| 53 | N | -5.913326 | -0.269165 | 1.164485 |
| 54 | C | -6.661430 | 0.124940 | 2.209847 |
| 55 | C | -7.607166 | -0.875557 | 2.555811 |
| 56 | C | -7.400351 | -1.913165 | 1.657742 |
| 57 | C | -3.069735 | -0.748381 | -2.253467 |
| 58 | N | -3.010357 | 0.358658 | -1.448144 |
| 59 | N | -2.067996 | 1.166627 | -2.000727 |
| 60 | C | -1.545392 | 0.601460 | -3.135677 |
| 61 | C | -2.158503 | -0.631133 | -3.331360 |
| 62 | C | -1.695025 | 2.474313 | -1.441416 |
| 63 | H | -2.563938 | 2.825401 | -0.858530 |
| 64 | H | -1.581496 | 3.164049 | -2.301250 |
| 65 | C | -0.428444 | 2.510463 | -0.555626 |
| 66 | H | -0.592208 | 1.871963 | 0.337625 |
| 67 | H | -0.374796 | 3.555424 | -0.181520 |
| 68 | C | 0.902398 | 2.151587 | -1.250786 |
| 69 | H | 0.957494 | 1.063078 | -1.457188 |
| 70 | H | 0.954847 | 2.659913 | -2.237488 |
| 71 | C | 2.185916 | 2.585893 | -0.502209 |
| 72 | H | 3.075948 | 2.356158 | -1.113798 |
| 73 | H | 2.179963 | 3.680275 | -0.330528 |
| 74 | N | 3.100036 | 0.810370 | 0.977486 |
| 75 | N | 2.394410 | 1.957600 | 0.808631 |
| 76 | C | 1.837599 | 2.368507 | 1.992616 |
| 77 | C | 2.178143 | 1.447075 | 2.976644 |
| 78 | C | 2.966595 | 0.483739 | 2.301885 |
| 79 | N | 4.377100 | -1.266386 | 1.695753 |
| 80 | C | 3.681845 | -0.712158 | 2.734833 |
| 81 | C | 3.738292 | -1.278593 | 4.020608 |
| 82 | C | 4.535081 | -2.422184 | 4.220840 |
| 83 | C | 5.277171 | -2.968928 | 3.158006 |
| 84 | C | 5.176059 | -2.341778 | 1.905754 |
| 85 | N | 5.881502 | -2.690169 | 0.744364 |
| 86 | N | 5.714526 | -1.853657 | -0.339705 |
| 87 | C | 6.500868 | -2.351253 | -1.309919 |
| 88 | C | 7.181424 | -3.512682 | -0.858334 |
| 89 | C | 6.769076 | -3.702592 | 0.452979 |
| 90 | H | 6.394829 | 0.718278 | 2.629917 |
| 91 | H | 8.351772 | 2.581025 | 2.031720 |
| 92 | H | 8.086622 | 3.002455 | -0.690244 |
| 93 | H | 2.340374 | -1.751383 | -4.455443 |
| 94 | H | 0.909137 | -3.252980 | -2.621941 |
| 95 | H | 1.258167 | 3.295421 | 2.046391 |
| 96 | H | 1.925316 | 1.490338 | 4.039797 |
| 97 | H | 6.556777 | -1.866132 | -2.290318 |
| 98 | H | 7.888103 | -4.130098 | -1.421990 |
| 99 | H | 7.038185 | -4.467026 | 1.189062 |
| 100 | H | -7.304387 | 4.146543 | -1.585273 |
| 101 | H | -7.982411 | 2.225481 | -3.455996 |
| 102 | H | -6.466606 | 0.019254 | -2.754587 |
| 103 | H | -1.904436 | 1.619823 | 4.283031 |
| 104 | H | -1.116180 | -0.996029 | 3.862502 |
| 105 | H | -6.503832 | 1.104512 | 2.674025 |
| 106 | H | -8.348868 | -0.839197 | 3.360131 |
| 107 | H | -7.899891 | -2.881409 | 1.549339 |
| 108 | H | -1.997889 | -1.326644 | -4.160098 |
| 109 | H | -0.795367 | 1.129276 | -3.732838 |
| 110 | H | -3.823220 | -3.266861 | -3.430225 |
| 111 | H | -5.650837 | -4.721104 | -2.485110 |
| 112 | H | -6.926925 | -3.970368 | -0.453806 |
| 113 | H | 4.221643 | 0.199558 | -5.019001 |

| | | | | |
|---|---|---|---|---|
| 114 | H | 6.104984 | 1.870328 | -5.105487 |
| 115 | H | 7.256183 | 2.572797 | -2.984723 |
| 116 | H | -3.385597 | 3.842232 | 3.526060 |
| 117 | H | -4.932935 | 5.570909 | 2.549309 |
| 118 | H | -6.242238 | 5.051164 | 0.467593 |
| 119 | H | 5.926861 | -3.840195 | 3.310356 |
| 120 | H | 4.599414 | -2.881276 | 5.217446 |
| 121 | H | 3.188378 | -0.825132 | 4.855649 |
| 122 | H | -0.843274 | -3.764380 | 1.152863 |

$[Fe_2(\mu\text{-}L^1)_2]^{4+}$ conformation (b), mixed-spin

| | | | | |
|---|---|---|---|---|
| 1 | Fe | 4.567851 | -0.389103 | -0.006705 |
| 2 | Fe | -4.548708 | 0.406345 | 0.014470 |
| 3 | N | -4.655549 | 2.282193 | 1.063152 |
| 4 | C | -5.545506 | 3.212502 | 0.665820 |
| 5 | C | -5.759577 | 4.407652 | 1.376498 |
| 6 | C | -4.997229 | 4.613554 | 2.537119 |
| 7 | C | -4.064835 | 3.647516 | 2.958354 |
| 8 | C | -3.922152 | 2.475653 | 2.195161 |
| 9 | N | -6.240417 | 2.866812 | -0.516950 |
| 10 | N | -5.968461 | 1.655631 | -1.100192 |
| 11 | C | -6.745743 | 1.600749 | -2.195094 |
| 12 | C | -7.527927 | 2.775623 | -2.328792 |
| 13 | C | -7.182786 | 3.564065 | -1.239300 |
| 14 | C | -3.037364 | 1.347604 | 2.523017 |
| 15 | N | -3.019518 | 0.281800 | 1.673342 |
| 16 | N | -2.182490 | -0.626917 | 2.228904 |
| 17 | C | -1.678488 | -0.170469 | 3.416386 |
| 18 | C | -2.194927 | 1.104970 | 3.637612 |
| 19 | C | -2.065407 | -1.989851 | 1.677990 |
| 20 | H | -2.572452 | -1.961462 | 0.695947 |
| 21 | H | -2.651715 | -2.667046 | 2.332585 |
| 22 | C | -0.630474 | -2.532567 | 1.543266 |
| 23 | H | -0.095883 | -2.447816 | 2.512812 |
| 24 | C | 0.205280 | -1.921411 | 0.402829 |
| 25 | H | 0.386184 | -0.841376 | 0.581707 |
| 26 | H | -0.372750 | -1.992291 | -0.542844 |
| 27 | C | 1.558405 | -2.645272 | 0.202038 |
| 28 | H | 2.269893 | -2.427400 | 1.017500 |
| 29 | H | 1.405632 | -3.742790 | 0.183033 |
| 30 | N | 3.184800 | -1.342735 | -1.164834 |
| 31 | N | 2.212564 | -2.285156 | -1.063096 |
| 32 | C | 1.795425 | -2.692092 | -2.305335 |
| 33 | C | 2.514238 | -1.982625 | -3.260069 |
| 34 | C | 3.371521 | -1.143659 | -2.507226 |
| 35 | N | 4.987963 | 0.348233 | -1.743246 |
| 36 | C | 4.396070 | -0.167665 | -2.863131 |
| 37 | C | 4.810490 | 0.236997 | -4.144163 |
| 38 | C | 5.858509 | 1.171432 | -4.250365 |
| 39 | C | 6.493188 | 1.667775 | -3.097017 |
| 40 | C | 6.028395 | 1.209979 | -1.854037 |
| 41 | N | 6.565839 | 1.526883 | -0.596635 |
| 42 | N | 6.025951 | 0.853792 | 0.480218 |
| 43 | C | 6.703391 | 1.286024 | 1.557836 |
| 44 | C | 7.685418 | 2.240709 | 1.184179 |
| 45 | C | 7.575378 | 2.371635 | -0.193519 |
| 46 | N | -4.929811 | -1.437958 | -1.021657 |
| 47 | C | -5.948352 | -2.228776 | -0.629788 |
| 48 | C | -6.321629 | -3.390086 | -1.333004 |
| 49 | C | -5.583434 | -3.715797 | -2.482438 |
| 50 | C | -4.512855 | -2.900082 | -2.893136 |

| | | | | |
|---|---|---|---|---|
| 51 | C | -4.210435 | -1.753842 | -2.134683 |
| 52 | N | -6.597854 | -1.776116 | 0.542824 |
| 53 | N | -6.147195 | -0.620857 | 1.128662 |
| 54 | C | -6.915962 | -0.446336 | 2.216992 |
| 55 | C | -7.870608 | -1.487423 | 2.343998 |
| 56 | C | -7.642072 | -2.320640 | 1.257307 |
| 57 | C | -3.147889 | -0.789167 | -2.452478 |
| 58 | N | -2.979613 | 0.282882 | -1.627492 |
| 59 | N | -2.001321 | 1.033923 | -2.189575 |
| 60 | C | -1.545472 | 0.464602 | -3.348382 |
| 61 | C | -2.249047 | -0.718855 | -3.548270 |
| 62 | C | -1.608905 | 2.342837 | -1.642044 |
| 63 | H | -2.475441 | 2.701112 | -1.056667 |
| 64 | H | -1.499323 | 3.028808 | -2.505532 |
| 65 | C | -0.340793 | 2.384824 | -0.761929 |
| 66 | H | -0.501028 | 1.754872 | 0.137274 |
| 67 | H | -0.287504 | 3.433082 | -0.397845 |
| 68 | C | 0.989120 | 2.020428 | -1.453921 |
| 69 | H | 1.049546 | 0.928390 | -1.636433 |
| 70 | H | 1.034589 | 2.504886 | -2.452763 |
| 71 | C | 2.270280 | 2.487874 | -0.721269 |
| 72 | H | 3.163579 | 2.210287 | -1.307235 |
| 73 | H | 2.272195 | 3.591313 | -0.622918 |
| 74 | N | 3.189325 | 0.830020 | 0.888491 |
| 75 | N | 2.462758 | 1.947319 | 0.631471 |
| 76 | C | 1.881052 | 2.425294 | 1.778062 |
| 77 | C | 2.225211 | 1.579903 | 2.826341 |
| 78 | C | 3.043241 | 0.590662 | 2.229651 |
| 79 | N | 4.478612 | -1.178753 | 1.752818 |
| 80 | C | 3.769442 | -0.563953 | 2.747865 |
| 81 | C | 3.822561 | -1.042658 | 4.069220 |
| 82 | C | 4.629731 | -2.161572 | 4.350735 |
| 83 | C | 5.384186 | -2.770778 | 3.331591 |
| 84 | C | 5.287333 | -2.228883 | 2.039680 |
| 85 | N | 6.006146 | -2.645380 | 0.909211 |
| 86 | N | 5.835934 | -1.886811 | -0.230285 |
| 87 | C | 6.638838 | -2.435883 | -1.157964 |
| 88 | C | 7.333104 | -3.553316 | -0.623498 |
| 89 | C | 6.912067 | -3.660637 | 0.694432 |
| 90 | H | 6.469589 | 0.901814 | 2.556399 |
| 91 | H | 8.387851 | 2.764004 | 1.840650 |
| 92 | H | 8.129846 | 2.992072 | -0.905250 |
| 93 | H | 2.447859 | -2.082973 | -4.347203 |
| 94 | H | 1.025866 | -3.463272 | -2.415506 |
| 95 | H | 1.285564 | 3.343134 | 1.765524 |
| 96 | H | 1.954926 | 1.689474 | 3.880184 |
| 97 | H | 6.695686 | -2.016749 | -2.168190 |
| 98 | H | 8.054411 | -4.196078 | -1.138115 |
| 99 | H | 7.185162 | -4.369474 | 1.482913 |
| 100 | H | -7.537305 | 4.553984 | -0.935094 |
| 101 | H | -8.252324 | 3.016188 | -3.113229 |
| 102 | H | -6.718725 | 0.718440 | -2.844768 |
| 103 | H | -2.011501 | 1.747767 | 4.504025 |
| 104 | H | -1.021365 | -0.789556 | 4.035938 |
| 105 | H | -6.763267 | 0.421364 | 2.869083 |
| 106 | H | -8.630419 | -1.612795 | 3.122055 |
| 107 | H | -8.141723 | -3.244250 | 0.948143 |
| 108 | H | -2.153969 | -1.404983 | -4.394829 |
| 109 | H | -0.784909 | 0.957431 | -3.962489 |
| 110 | H | -3.938264 | -3.149719 | -3.794799 |
| 111 | H | -5.850693 | -4.610838 | -3.063493 |
| 112 | H | -7.159572 | -4.018712 | -1.007071 |

| | | | | |
|---|---|---|---|---|
| 113 | H | 4.338408 | -0.181648 | -5.042501 |
| 114 | H | 6.202772 | 1.501569 | -5.240603 |
| 115 | H | 7.333741 | 2.369529 | -3.171975 |
| 116 | H | -3.474414 | 3.802611 | 3.870554 |
| 117 | H | -5.138304 | 5.533373 | 3.121366 |
| 118 | H | -6.495405 | 5.153271 | 1.051049 |
| 119 | H | 6.040038 | -3.624208 | 3.546358 |
| 120 | H | 4.691983 | -2.552191 | 5.376422 |
| 121 | H | 3.262396 | -0.539680 | 4.868394 |
| 122 | H | -0.738272 | -3.624082 | 1.371415 |

$[Fe_2(\mu\text{-}L^1)_2]^{4+}$ conformation (b), high-spin

| | | | | |
|---|---|---|---|---|
| 1 | Fe | -4.651552 | 0.429962 | 0.001045 |
| 2 | Fe | 4.684325 | -0.395815 | 0.013638 |
| 3 | N | -4.770262 | 2.231770 | 1.175948 |
| 4 | C | -5.675441 | 3.174235 | 0.849797 |
| 5 | C | -5.901306 | 4.313340 | 1.645073 |
| 6 | C | -5.134745 | 4.446284 | 2.814174 |
| 7 | C | -4.183736 | 3.467282 | 3.159020 |
| 8 | C | -4.028699 | 2.355538 | 2.311775 |
| 9 | N | -6.369436 | 2.904347 | -0.353506 |
| 10 | N | -6.075644 | 1.747045 | -1.028121 |
| 11 | C | -6.856842 | 1.759248 | -2.121884 |
| 12 | C | -7.661160 | 2.925972 | -2.165375 |
| 13 | C | -7.327641 | 3.635815 | -1.018938 |
| 14 | C | -3.116948 | 1.223788 | 2.541890 |
| 15 | N | -3.111985 | 0.214325 | 1.625858 |
| 16 | N | -2.235080 | -0.709365 | 2.086315 |
| 17 | C | -1.692004 | -0.318151 | 3.280297 |
| 18 | C | -2.222722 | 0.929056 | 3.602521 |
| 19 | C | -2.136444 | -2.035115 | 1.446076 |
| 20 | H | -2.633506 | -1.929045 | 0.464121 |
| 21 | H | -2.743118 | -2.741879 | 2.048757 |
| 22 | C | -0.712205 | -2.597953 | 1.288458 |
| 23 | H | -0.180186 | -2.569659 | 2.262976 |
| 24 | C | 0.144500 | -1.956784 | 0.178595 |
| 25 | H | 0.323191 | -0.882698 | 0.391425 |
| 26 | H | -0.415109 | -2.002218 | -0.779915 |
| 27 | C | 1.497866 | -2.684059 | -0.005304 |
| 28 | H | 2.201362 | -2.456403 | 0.816991 |
| 29 | H | 1.340849 | -3.781294 | -0.009491 |
| 30 | N | 3.174199 | -1.433095 | -1.332794 |
| 31 | N | 2.180788 | -2.350620 | -1.262606 |
| 32 | C | 1.865738 | -2.839383 | -2.501935 |
| 33 | C | 2.690233 | -2.209148 | -3.429730 |
| 34 | C | 3.494480 | -1.334864 | -2.655322 |
| 35 | N | 5.166053 | 0.211626 | -1.989934 |
| 36 | C | 4.573205 | -0.413283 | -3.043771 |
| 37 | C | 5.000888 | -0.171254 | -4.362268 |
| 38 | C | 6.057549 | 0.734765 | -4.569404 |
| 39 | C | 6.670054 | 1.374499 | -3.479361 |
| 40 | C | 6.183591 | 1.070088 | -2.194255 |
| 41 | N | 6.711221 | 1.617607 | -1.000555 |
| 42 | N | 6.186695 | 1.198481 | 0.196632 |
| 43 | C | 6.878770 | 1.855552 | 1.142601 |
| 44 | C | 7.859971 | 2.701487 | 0.565955 |
| 45 | C | 7.725722 | 2.527680 | -0.804751 |
| 46 | N | -5.033196 | -1.340930 | -1.162302 |
| 47 | C | -6.060811 | -2.149213 | -0.834381 |
| 48 | C | -6.435701 | -3.256482 | -1.618413 |
| 49 | C | -5.687802 | -3.509512 | -2.779235 |

| | | | | |
|---|---|---|---|---|
| 50 | C | -4.606022 | -2.678185 | -3.122472 |
| 51 | C | -4.304090 | -1.588062 | -2.286479 |
| 52 | N | -6.715900 | -1.775377 | 0.362709 |
| 53 | N | -6.251000 | -0.676793 | 1.039571 |
| 54 | C | -7.029786 | -0.569422 | 2.129782 |
| 55 | C | -8.002932 | -1.599920 | 2.168678 |
| 56 | C | -7.777211 | -2.352548 | 1.023406 |
| 57 | C | -3.230315 | -0.612805 | -2.522244 |
| 58 | N | -3.074035 | 0.398832 | -1.622165 |
| 59 | N | -2.078943 | 1.179285 | -2.107294 |
| 60 | C | -1.600316 | 0.688262 | -3.292335 |
| 61 | C | -2.306937 | -0.473179 | -3.590633 |
| 62 | C | -1.696281 | 2.446206 | -1.462046 |
| 63 | H | -2.581729 | 2.778259 | -0.889348 |
| 64 | H | -1.545982 | 3.182533 | -2.276336 |
| 65 | C | -0.460310 | 2.418190 | -0.536727 |
| 66 | H | -0.661540 | 1.750564 | 0.325659 |
| 67 | H | -0.399328 | 3.446902 | -0.121687 |
| 68 | C | 0.882935 | 2.059543 | -1.204572 |
| 69 | H | 0.943148 | 0.971556 | -1.411483 |
| 70 | H | 0.954272 | 2.564776 | -2.191575 |
| 71 | C | 2.149071 | 2.509803 | -0.440246 |
| 72 | H | 3.049868 | 2.248222 | -1.025603 |
| 73 | H | 2.146888 | 3.611300 | -0.318358 |
| 74 | N | 3.147775 | 0.867875 | 1.115089 |
| 75 | N | 2.346799 | 1.939452 | 0.901245 |
| 76 | C | 1.824854 | 2.408509 | 2.077069 |
| 77 | C | 2.300921 | 1.601735 | 3.107098 |
| 78 | C | 3.130413 | 0.648973 | 2.460302 |
| 79 | N | 4.657340 | -1.117464 | 2.040891 |
| 80 | C | 3.959073 | -0.441416 | 2.995437 |
| 81 | C | 4.075226 | -0.779481 | 4.355909 |
| 82 | C | 4.930330 | -1.837517 | 4.716017 |
| 83 | C | 5.653156 | -2.530463 | 3.731559 |
| 84 | C | 5.483903 | -2.121660 | 2.396250 |
| 85 | N | 6.165709 | -2.706581 | 1.303453 |
| 86 | N | 5.971167 | -2.172215 | 0.054608 |
| 87 | C | 6.750673 | -2.891191 | -0.771880 |
| 88 | C | 7.454982 | -3.897136 | -0.064213 |
| 89 | C | 7.060854 | -3.751282 | 1.260047 |
| 90 | H | 6.649293 | 1.698443 | 2.203160 |
| 91 | H | 8.571662 | 3.354560 | 1.079972 |
| 92 | H | 8.272650 | 2.980913 | -1.637650 |
| 93 | H | 2.717915 | -2.384245 | -4.509250 |
| 94 | H | 1.095000 | -3.606591 | -2.633036 |
| 95 | H | 1.179493 | 3.292809 | 2.100103 |
| 96 | H | 2.100972 | 1.714793 | 4.176308 |
| 97 | H | 6.777499 | -2.663979 | -1.844480 |
| 98 | H | 8.159404 | -4.632516 | -0.464927 |
| 99 | H | 7.349737 | -4.310446 | 2.155539 |
| 100 | H | -7.699554 | 4.593234 | -0.639970 |
| 101 | H | -8.392215 | 3.211767 | -2.928540 |
| 102 | H | -6.814920 | 0.929133 | -2.836227 |
| 103 | H | -2.011327 | 1.519155 | 4.498814 |
| 104 | H | -0.998027 | -0.959433 | 3.832564 |
| 105 | H | -6.865702 | 0.243199 | 2.846822 |
| 106 | H | -8.772630 | -1.770544 | 2.928244 |
| 107 | H | -8.290511 | -3.240818 | 0.640722 |
| 108 | H | -2.196998 | -1.100908 | -4.479740 |
| 109 | H | -0.822786 | 1.215402 | -3.854557 |
| 110 | H | -4.022332 | -2.871218 | -4.031892 |
| 111 | H | -5.955437 | -4.360794 | -3.421780 |

| | | | | |
|---|---|---|---|---|
| 112 | H | -7.280686 | -3.900895 | -1.345085 |
| 113 | H | 4.527155 | -0.679311 | -5.211934 |
| 114 | H | 6.414106 | 0.941190 | -5.588699 |
| 115 | H | 7.502029 | 2.072350 | -3.635129 |
| 116 | H | -3.588921 | 3.565050 | 4.076367 |
| 117 | H | -5.286464 | 5.319519 | 3.464866 |
| 118 | H | -6.647775 | 5.071793 | 1.377709 |
| 119 | H | 6.330656 | -3.348626 | 4.005663 |
| 120 | H | 5.043794 | -2.120751 | 5.771317 |
| 121 | H | 3.521914 | -0.223665 | 5.123666 |
| 122 | H | -0.841662 | -3.678214 | 1.067586 |

$[Fe_2(\mu\text{-}L^1)_2]^{4+}$ conformation (c), low-spin

| | | | | |
|---|---|---|---|---|
| 1 | Fe | 0.127109 | 0.096823 | 4.466402 |
| 2 | Fe | -0.146474 | -0.063919 | -4.469664 |
| 3 | N | 1.970122 | -0.374720 | 4.800690 |
| 4 | C | 2.653940 | 0.302373 | 5.755079 |
| 5 | C | 3.949488 | -0.065135 | 6.152674 |
| 6 | H | 4.493728 | 0.497340 | 6.922135 |
| 7 | C | 4.518168 | -1.199460 | 5.545198 |
| 8 | H | 5.527799 | -1.520940 | 5.837766 |
| 9 | C | 3.793428 | -1.937004 | 4.589439 |
| 10 | H | 4.220008 | -2.843319 | 4.139988 |
| 11 | C | 2.504126 | -1.500813 | 4.237250 |
| 12 | N | 1.887596 | 1.351258 | 6.287984 |
| 13 | N | 0.592268 | 1.451352 | 5.823169 |
| 14 | C | 0.043188 | 2.480947 | 6.490429 |
| 15 | H | -0.995169 | 2.776697 | 6.307033 |
| 16 | C | 0.979307 | 3.051283 | 7.392524 |
| 17 | H | 0.821178 | 3.897221 | 8.069062 |
| 18 | C | 2.143129 | 2.310218 | 7.242793 |
| 19 | H | 3.117140 | 2.391477 | 7.736234 |
| 20 | C | 1.555539 | -2.119568 | 3.316277 |
| 21 | N | 0.358950 | -1.467448 | 3.166912 |
| 22 | N | -0.370043 | -2.228191 | 2.309319 |
| 23 | C | 0.333055 | -3.341131 | 1.924902 |
| 24 | H | -0.107135 | -4.080104 | 1.247363 |
| 25 | C | 1.575421 | -3.309368 | 2.547864 |
| 26 | H | 2.366947 | -4.061545 | 2.482382 |
| 27 | C | -1.668056 | -1.822011 | 1.749760 |
| 28 | H | -2.086574 | -1.049066 | 2.418115 |
| 29 | H | -2.337329 | -2.704205 | 1.804356 |
| 30 | C | -1.545804 | -1.327922 | 0.288068 |
| 31 | H | -1.004126 | -0.360455 | 0.278820 |
| 32 | H | -0.910473 | -2.048315 | -0.267922 |
| 33 | C | -2.896807 | -1.196115 | -0.437297 |
| 34 | H | -3.564385 | -0.489817 | 0.099522 |
| 35 | H | -3.429491 | -2.169878 | -0.414946 |
| 36 | C | -2.795761 | -0.798572 | -1.925735 |
| 37 | H | -2.110569 | -1.472094 | -2.469910 |
| 38 | H | -3.795449 | -0.879700 | -2.399362 |
| 39 | N | -1.267023 | 0.836332 | -3.009480 |
| 40 | N | -2.287110 | 0.565201 | -2.155459 |
| 41 | C | -2.751423 | 1.713810 | -1.570054 |
| 42 | H | -3.597727 | 1.694631 | -0.876775 |
| 43 | C | -1.990926 | 2.780040 | -2.040080 |
| 44 | H | -2.108750 | 3.837782 | -1.788302 |
| 45 | C | -1.064757 | 2.189656 | -2.932859 |
| 46 | N | 0.603137 | 1.715511 | -4.488717 |
| 47 | C | 0.011813 | 2.715830 | -3.767130 |
| 48 | C | 0.440200 | 4.048405 | -3.903284 |

| | | | | |
|---|---|---|---|---|
| 49 | H | -0.043059 | 4.851513 | -3.331542 |
| 50 | C | 1.480588 | 4.334806 | -4.808153 |
| 51 | H | 1.829579 | 5.369144 | -4.936439 |
| 52 | C | 2.060096 | 3.308697 | -5.576973 |
| 53 | H | 2.847483 | 3.526445 | -6.309840 |
| 54 | C | 1.571330 | 2.005114 | -5.392602 |
| 55 | N | 1.957960 | 0.863839 | -6.112039 |
| 56 | N | 1.242168 | -0.286976 | -5.853553 |
| 57 | C | 1.744494 | -1.222949 | -6.676823 |
| 58 | H | 1.343639 | -2.242168 | -6.676186 |
| 59 | C | 2.789542 | -0.683518 | -7.472277 |
| 60 | H | 3.382783 | -1.202638 | -8.231836 |
| 61 | C | 2.901024 | 0.646501 | -7.092386 |
| 62 | H | 3.567706 | 1.441514 | -7.441985 |
| 63 | N | -1.737893 | 0.597011 | 4.470194 |
| 64 | C | -2.568456 | 0.011776 | 5.368244 |
| 65 | C | -3.899062 | 0.427098 | 5.539688 |
| 66 | H | -4.561098 | -0.057127 | 6.268827 |
| 67 | C | -4.346462 | 1.510943 | 4.761781 |
| 68 | H | -5.378616 | 1.870136 | 4.879428 |
| 69 | C | -3.474877 | 2.152920 | 3.860996 |
| 70 | H | -3.808811 | 3.023435 | 3.281327 |
| 71 | C | -2.158371 | 1.673898 | 3.738567 |
| 72 | N | -1.911142 | -0.993131 | 6.093859 |
| 73 | N | -0.560207 | -1.132783 | 5.849697 |
| 74 | C | -0.138645 | -2.105591 | 6.676274 |
| 75 | H | 0.911988 | -2.414920 | 6.689103 |
| 76 | C | -1.214659 | -2.600935 | 7.459037 |
| 77 | H | -1.176753 | -3.389701 | 8.217170 |
| 78 | C | -2.328965 | -1.872200 | 7.068239 |
| 79 | H | -3.370028 | -1.912334 | 7.404752 |
| 80 | C | -1.074481 | 2.200698 | 2.914390 |
| 81 | N | 0.114176 | 1.523918 | 2.995430 |
| 82 | N | 0.965774 | 2.169557 | 2.158683 |
| 83 | C | 0.355231 | 3.250493 | 1.578686 |
| 84 | H | 0.901024 | 3.908996 | 0.895536 |
| 85 | C | -0.958052 | 3.304189 | 2.035131 |
| 86 | H | -1.715401 | 4.052302 | 1.784511 |
| 87 | C | 2.353553 | 1.727683 | 1.934652 |
| 88 | H | 2.459200 | 0.776582 | 2.485913 |
| 89 | H | 3.032966 | 2.467395 | 2.404765 |
| 90 | C | 2.733566 | 1.554724 | 0.449048 |
| 91 | H | 3.828522 | 1.372102 | 0.433147 |
| 92 | H | 2.593674 | 2.514836 | -0.091780 |
| 93 | C | 2.006432 | 0.408706 | -0.276697 |
| 94 | H | 0.910164 | 0.575707 | -0.265013 |
| 95 | H | 2.185304 | -0.536432 | 0.277577 |
| 96 | C | 2.466663 | 0.209118 | -1.741114 |
| 97 | H | 2.103617 | 1.016690 | -2.400734 |
| 98 | H | 3.573693 | 0.204748 | -1.801561 |
| 99 | N | 0.950689 | -1.187179 | -3.159803 |
| 100 | N | 2.000815 | -1.066978 | -2.304862 |
| 101 | C | 2.464564 | -2.300237 | -1.923973 |
| 102 | H | 3.320840 | -2.396064 | -1.248404 |
| 103 | C | 1.685518 | -3.270309 | -2.543192 |
| 104 | H | 1.804618 | -4.355796 | -2.478688 |
| 105 | C | 0.746738 | -2.534964 | -3.306783 |
| 106 | N | -0.892293 | -1.816427 | -4.795253 |
| 107 | C | -0.322178 | -2.919395 | -4.222794 |
| 108 | C | -0.762248 | -4.211594 | -4.560057 |
| 109 | H | -0.300284 | -5.097055 | -4.104102 |
| 110 | C | -1.795552 | -4.346239 | -5.506909 |

| | | | | |
|---|---|---|---|---|
| 111 | H | -2.157347 | -5.345528 | -5.787645 |
| 112 | C | -2.354051 | -3.210453 | -6.121533 |
| 113 | H | -3.138009 | -3.308128 | -6.883393 |
| 114 | C | -1.853267 | -1.955130 | -5.740634 |
| 115 | N | -2.223506 | -0.712935 | -6.279124 |
| 116 | N | -1.510699 | 0.378633 | -5.827631 |
| 117 | C | -2.000456 | 1.436677 | -6.497142 |
| 118 | H | -1.599685 | 2.441327 | -6.326385 |
| 119 | C | -3.032375 | 1.035478 | -7.385081 |
| 120 | H | -3.613404 | 1.671849 | -8.060132 |
| 121 | C | -3.150445 | -0.338142 | -7.225729 |
| 122 | H | -3.812299 | -1.064612 | -7.708405 |

$[Fe_2(\mu\text{-}L^1)_2]^{4+}$ conformation (c), mixed-spin

| | | | | |
|---|---|---|---|---|
| 1 | Fe | 0.095393 | 0.101960 | 4.523399 |
| 2 | Fe | -0.130226 | -0.072655 | -4.542741 |
| 3 | N | 2.091376 | -0.608329 | 4.914083 |
| 4 | C | 2.840683 | 0.015503 | 5.844010 |
| 5 | C | 4.087286 | -0.477946 | 6.270823 |
| 6 | H | 4.687315 | 0.037428 | 7.031064 |
| 7 | C | 4.537045 | -1.678803 | 5.697989 |
| 8 | H | 5.502907 | -2.100662 | 6.010650 |
| 9 | C | 3.752163 | -2.346631 | 4.739985 |
| 10 | H | 4.092643 | -3.293432 | 4.301359 |
| 11 | C | 2.519694 | -1.779513 | 4.367213 |
| 12 | N | 2.246232 | 1.192381 | 6.358349 |
| 13 | N | 0.996187 | 1.538643 | 5.913240 |
| 14 | C | 0.665753 | 2.650149 | 6.593760 |
| 15 | H | -0.301618 | 3.135763 | 6.420707 |
| 16 | C | 1.699509 | 3.031792 | 7.485156 |
| 17 | H | 1.717856 | 3.887883 | 8.167143 |
| 18 | C | 2.695254 | 2.079112 | 7.310628 |
| 19 | H | 3.672943 | 1.974464 | 7.791173 |
| 20 | C | 1.572833 | -2.369406 | 3.409107 |
| 21 | N | 0.424472 | -1.688843 | 3.128622 |
| 22 | N | -0.269547 | -2.478026 | 2.273911 |
| 23 | C | 0.407029 | -3.637068 | 2.004673 |
| 24 | H | -0.013474 | -4.403830 | 1.344975 |
| 25 | C | 1.601955 | -3.607413 | 2.717266 |
| 26 | H | 2.366160 | -4.389203 | 2.752013 |
| 27 | C | -1.547638 | -2.069317 | 1.675313 |
| 28 | H | -1.980102 | -1.295248 | 2.336676 |
| 29 | H | -2.219939 | -2.949872 | 1.712022 |
| 30 | C | -1.401756 | -1.563326 | 0.221339 |
| 31 | H | -0.871103 | -0.589810 | 0.231450 |
| 32 | H | -0.753139 | -2.271860 | -0.335437 |
| 33 | C | -2.753889 | -1.440200 | -0.505499 |
| 34 | H | -3.440975 | -0.774327 | 0.057628 |
| 35 | H | -3.259534 | -2.428426 | -0.516489 |
| 36 | C | -2.675412 | -0.987692 | -1.977539 |
| 37 | H | -1.969020 | -1.613928 | -2.549357 |
| 38 | H | -3.674047 | -1.091782 | -2.449436 |
| 39 | N | -1.278549 | 0.752787 | -3.070301 |
| 40 | N | -2.223807 | 0.403023 | -2.161575 |
| 41 | C | -2.702818 | 1.507832 | -1.507063 |
| 42 | H | -3.495647 | 1.421648 | -0.757964 |
| 43 | C | -2.030754 | 2.625776 | -1.991213 |
| 44 | H | -2.178785 | 3.666891 | -1.691500 |
| 45 | C | -1.141077 | 2.112052 | -2.964889 |
| 46 | N | 0.489353 | 1.755084 | -4.587477 |
| 47 | C | -0.137904 | 2.712658 | -3.839415 |

| | | | | |
|---|---|---|---|---|
| 48 | C | 0.201394 | 4.069943 | -3.982754 |
| 49 | H | -0.311525 | 4.839615 | -3.391234 |
| 50 | C | 1.194605 | 4.423399 | -4.916553 |
| 51 | H | 1.473266 | 5.478111 | -5.050836 |
| 52 | C | 1.819452 | 3.437636 | -5.702237 |
| 53 | H | 2.575304 | 3.705916 | -6.451462 |
| 54 | C | 1.419895 | 2.105351 | -5.508924 |
| 55 | N | 1.873179 | 0.989471 | -6.229465 |
| 56 | N | 1.254598 | -0.209955 | -5.942611 |
| 57 | C | 1.812122 | -1.114692 | -6.765014 |
| 58 | H | 1.493032 | -2.162234 | -6.743788 |
| 59 | C | 2.796189 | -0.506132 | -7.588201 |
| 60 | H | 3.414392 | -0.987682 | -8.352576 |
| 61 | C | 2.811081 | 0.833421 | -7.226060 |
| 62 | H | 3.408350 | 1.672962 | -7.596232 |
| 63 | N | -1.924953 | 0.844091 | 4.576745 |
| 64 | C | -2.795451 | 0.326981 | 5.466088 |
| 65 | C | -4.078473 | 0.866949 | 5.674597 |
| 66 | H | -4.776243 | 0.437354 | 6.404006 |
| 67 | C | -4.433434 | 1.998134 | 4.922204 |
| 68 | H | -5.423285 | 2.454939 | 5.063846 |
| 69 | C | -3.524977 | 2.552334 | 4.001749 |
| 70 | H | -3.793524 | 3.447072 | 3.425364 |
| 71 | C | -2.265101 | 1.944838 | 3.850083 |
| 72 | N | -2.288756 | -0.788936 | 6.172465 |
| 73 | N | -0.999700 | -1.186376 | 5.921853 |
| 74 | C | -0.772870 | -2.220128 | 6.750300 |
| 75 | H | 0.198286 | -2.728586 | 6.746689 |
| 76 | C | -1.913161 | -2.500039 | 7.544859 |
| 77 | H | -2.028085 | -3.278449 | 8.305918 |
| 78 | C | -2.864192 | -1.567551 | 7.152229 |
| 79 | H | -3.890100 | -1.406696 | 7.498099 |
| 80 | C | -1.197788 | 2.423875 | 2.958882 |
| 81 | N | -0.030642 | 1.721249 | 2.911816 |
| 82 | N | 0.776123 | 2.399466 | 2.061597 |
| 83 | C | 0.157812 | 3.519528 | 1.577303 |
| 84 | H | 0.672794 | 4.208495 | 0.899887 |
| 85 | C | -1.121396 | 3.569305 | 2.125558 |
| 86 | H | -1.873991 | 4.346929 | 1.965671 |
| 87 | C | 2.172909 | 1.992468 | 1.824191 |
| 88 | H | 2.308298 | 1.043151 | 2.375299 |
| 89 | H | 2.831303 | 2.748844 | 2.298012 |
| 90 | C | 2.561229 | 1.836989 | 0.341058 |
| 91 | H | 3.665449 | 1.722860 | 0.325760 |
| 92 | H | 2.360406 | 2.784169 | -0.203327 |
| 93 | C | 1.910241 | 0.643999 | -0.381133 |
| 94 | H | 0.808031 | 0.763980 | -0.415346 |
| 95 | H | 2.106269 | -0.279421 | 0.203574 |
| 96 | C | 2.444599 | 0.432928 | -1.817941 |
| 97 | H | 2.069730 | 1.201151 | -2.516595 |
| 98 | H | 3.551540 | 0.490832 | -1.829986 |
| 99 | N | 1.049812 | -1.092660 | -3.222806 |
| 100 | N | 2.083725 | -0.882272 | -2.367186 |
| 101 | C | 2.631708 | -2.072014 | -1.960509 |
| 102 | H | 3.489187 | -2.093961 | -1.279756 |
| 103 | C | 1.926441 | -3.106237 | -2.564619 |
| 104 | H | 2.121486 | -4.179027 | -2.477261 |
| 105 | C | 0.942087 | -2.453676 | -3.346424 |
| 106 | N | -0.749661 | -1.877255 | -4.838391 |
| 107 | C | -0.097091 | -2.927916 | -4.255315 |
| 108 | C | -0.441178 | -4.252807 | -4.576556 |
| 109 | H | 0.085877 | -5.096236 | -4.111653 |

| | | | | |
|---|---|---|---|---|
| 110 | C | -1.461874 | -4.474648 | -5.520678 |
| 111 | H | -1.747736 | -5.501204 | -5.790279 |
| 112 | C | -2.104722 | -3.390628 | -6.146204 |
| 113 | H | -2.879375 | -3.554747 | -6.906037 |
| 114 | C | -1.699925 | -2.097274 | -5.778984 |
| 115 | N | -2.162963 | -0.891233 | -6.328474 |
| 116 | N | -1.532938 | 0.255474 | -5.889241 |
| 117 | C | -2.106895 | 1.268080 | -6.561741 |
| 118 | H | -1.783977 | 2.301824 | -6.399326 |
| 119 | C | -3.111070 | 0.782338 | -7.439936 |
| 120 | H | -3.744173 | 1.367364 | -8.114589 |
| 121 | C | -3.122253 | -0.595172 | -7.270915 |
| 122 | H | -3.730000 | -1.373254 | -7.744374 |

$[Fe_2(\mu\text{-}L^1)_2]^{4+}$ conformation (c), high-spin

| | | | | |
|---|---|---|---|---|
| 1 | Fe | 0.076295 | 0.094286 | 4.726176 |
| 2 | Fe | -0.100785 | -0.041075 | -4.738458 |
| 3 | N | 1.933549 | -0.824748 | 5.300746 |
| 4 | C | 2.700998 | -0.249139 | 6.245315 |
| 5 | C | 3.882589 | -0.842918 | 6.726313 |
| 6 | H | 4.496157 | -0.370620 | 7.503434 |
| 7 | C | 4.251865 | -2.082482 | 6.178168 |
| 8 | H | 5.167467 | -2.579511 | 6.530152 |
| 9 | C | 3.454422 | -2.690051 | 5.190262 |
| 10 | H | 3.737591 | -3.659775 | 4.761487 |
| 11 | C | 2.284528 | -2.028938 | 4.773020 |
| 12 | N | 2.196817 | 0.990784 | 6.707872 |
| 13 | N | 1.031553 | 1.461745 | 6.160368 |
| 14 | C | 0.791581 | 2.632374 | 6.776969 |
| 15 | H | -0.098537 | 3.217692 | 6.519661 |
| 16 | C | 1.801758 | 2.929393 | 7.725768 |
| 17 | H | 1.876436 | 3.805523 | 8.377937 |
| 18 | C | 2.684137 | 1.858448 | 7.658456 |
| 19 | H | 3.605962 | 1.658768 | 8.213083 |
| 20 | C | 1.326655 | -2.540917 | 3.781705 |
| 21 | N | 0.285971 | -1.737585 | 3.412466 |
| 22 | N | -0.467548 | -2.480281 | 2.565782 |
| 23 | C | 0.069424 | -3.727090 | 2.385906 |
| 24 | H | -0.423190 | -4.473177 | 1.753206 |
| 25 | C | 1.231921 | -3.804564 | 3.146505 |
| 26 | H | 1.889299 | -4.672220 | 3.253857 |
| 27 | C | -1.667671 | -1.962813 | 1.894971 |
| 28 | H | -2.102505 | -1.183302 | 2.548190 |
| 29 | H | -2.392916 | -2.799836 | 1.863575 |
| 30 | C | -1.397200 | -1.436158 | 0.466099 |
| 31 | H | -0.883729 | -0.455546 | 0.528508 |
| 32 | H | -0.693199 | -2.132651 | -0.036452 |
| 33 | C | -2.679933 | -1.330841 | -0.382158 |
| 34 | H | -3.424637 | -0.666499 | 0.104012 |
| 35 | H | -3.172336 | -2.324977 | -0.422765 |
| 36 | C | -2.470357 | -0.908637 | -1.848003 |
| 37 | H | -1.654634 | -1.492021 | -2.316036 |
| 38 | H | -3.397052 | -1.111496 | -2.424643 |
| 39 | N | -1.246079 | 0.879288 | -3.019318 |
| 40 | N | -2.117606 | 0.510152 | -2.049582 |
| 41 | C | -2.708676 | 1.603363 | -1.477125 |
| 42 | H | -3.472182 | 1.500836 | -0.699822 |
| 43 | C | -2.183325 | 2.740478 | -2.086516 |
| 44 | H | -2.448907 | 3.780383 | -1.875707 |
| 45 | C | -1.273632 | 2.242120 | -3.052932 |
| 46 | N | 0.238538 | 2.077831 | -4.871868 |

| | | | | |
|---|---|---|---|---|
| 47 | C | -0.441615 | 2.926915 | -4.052886 |
| 48 | C | -0.341152 | 4.321289 | -4.210742 |
| 49 | H | -0.893775 | 5.004533 | -3.553338 |
| 50 | C | 0.471232 | 4.824201 | -5.244349 |
| 51 | H | 0.557557 | 5.908810 | -5.400047 |
| 52 | C | 1.171266 | 3.946353 | -6.087406 |
| 53 | H | 1.802192 | 4.331818 | -6.898110 |
| 54 | C | 1.020771 | 2.566561 | -5.853948 |
| 55 | N | 1.661703 | 1.564429 | -6.619185 |
| 56 | N | 1.412420 | 0.253258 | -6.306141 |
| 57 | C | 2.156256 | -0.469056 | -7.161504 |
| 58 | H | 2.133021 | -1.564101 | -7.123910 |
| 59 | C | 2.896443 | 0.370011 | -8.032186 |
| 60 | H | 3.584168 | 0.072117 | -8.830074 |
| 61 | C | 2.556387 | 1.664229 | -7.661066 |
| 62 | H | 2.883657 | 2.629927 | -8.059180 |
| 63 | N | -1.823757 | 1.105405 | 4.819343 |
| 64 | C | -2.703465 | 0.781732 | 5.787176 |
| 65 | C | -3.897914 | 1.495647 | 5.997170 |
| 66 | H | -4.602072 | 1.227497 | 6.794662 |
| 67 | C | -4.159099 | 2.582850 | 5.148247 |
| 68 | H | -5.078821 | 3.169186 | 5.285413 |
| 69 | C | -3.247863 | 2.925475 | 4.132430 |
| 70 | H | -3.444962 | 3.777957 | 3.469564 |
| 71 | C | -2.074785 | 2.160836 | 3.995859 |
| 72 | N | -2.305996 | -0.328973 | 6.567249 |
| 73 | N | -1.100517 | -0.918138 | 6.286737 |
| 74 | C | -0.984548 | -1.934399 | 7.158743 |
| 75 | H | -0.091628 | -2.570038 | 7.147468 |
| 76 | C | -2.117081 | -2.012369 | 8.008786 |
| 77 | H | -2.306185 | -2.730352 | 8.813050 |
| 78 | C | -2.942287 | -0.970537 | 7.605753 |
| 79 | H | -3.918964 | -0.649489 | 7.980643 |
| 80 | C | -1.008650 | 2.406552 | 3.013853 |
| 81 | N | 0.054301 | 1.552315 | 2.992116 |
| 82 | N | 0.891570 | 2.021340 | 2.034629 |
| 83 | C | 0.397017 | 3.162234 | 1.464266 |
| 84 | H | 0.955344 | 3.712239 | 0.700666 |
| 85 | C | -0.832479 | 3.438169 | 2.057276 |
| 86 | H | -1.489706 | 4.286152 | 1.844849 |
| 87 | C | 2.232368 | 1.434518 | 1.843376 |
| 88 | H | 2.195537 | 0.432578 | 2.311787 |
| 89 | H | 2.954338 | 2.046251 | 2.422817 |
| 90 | C | 2.705076 | 1.338912 | 0.380895 |
| 91 | H | 3.797341 | 1.145830 | 0.428954 |
| 92 | H | 2.617520 | 2.326847 | -0.118533 |
| 93 | C | 2.037883 | 0.234995 | -0.462355 |
| 94 | H | 0.940352 | 0.386035 | -0.518317 |
| 95 | H | 2.197854 | -0.742678 | 0.040876 |
| 96 | C | 2.612187 | 0.147778 | -1.895440 |
| 97 | H | 2.232185 | 0.959342 | -2.542654 |
| 98 | H | 3.715835 | 0.240203 | -1.874660 |
| 99 | N | 1.254540 | -1.289950 | -3.396148 |
| 100 | N | 2.313447 | -1.124310 | -2.566964 |
| 101 | C | 2.990462 | -2.299887 | -2.381595 |
| 102 | H | 3.890202 | -2.346648 | -1.758563 |
| 103 | C | 2.343876 | -3.284420 | -3.122070 |
| 104 | H | 2.640333 | -4.332690 | -3.222058 |
| 105 | C | 1.267126 | -2.608715 | -3.750112 |
| 106 | N | -0.487124 | -2.089301 | -5.263020 |
| 107 | C | 0.267670 | -3.083347 | -4.718437 |
| 108 | C | 0.082630 | -4.425912 | -5.097475 |

| | | | | |
|---|---|---|---|---|
| 109 | H | 0.689324 | -5.226098 | -4.653917 |
| 110 | C | -0.894266 | -4.721785 | -6.065979 |
| 111 | H | -1.054052 | -5.760961 | -6.388491 |
| 112 | C | -1.667054 | -3.693994 | -6.630746 |
| 113 | H | -2.425299 | -3.916902 | -7.391395 |
| 114 | C | -1.422250 | -2.381017 | -6.187249 |
| 115 | N | -2.118784 | -1.246462 | -6.667735 |
| 116 | N | -1.787044 | -0.017584 | -6.158638 |
| 117 | C | -2.596022 | 0.858441 | -6.778878 |
| 118 | H | -2.528933 | 1.928642 | -6.550795 |
| 119 | C | -3.458300 | 0.202802 | -7.693453 |
| 120 | H | -4.218866 | 0.650369 | -8.340910 |
| 121 | C | -3.127072 | -1.142642 | -7.599162 |
| 122 | H | -3.529882 | -2.014427 | -8.123410 |

$[Fe_2(\mu\text{-}L^2)_2]^{4+}$, low-spin

| | | | | |
|---|---|---|---|---|
| 1 | Fe | 3.669890 | -0.000000 | 0.000001 |
| 2 | Fe | -3.669917 | -0.000000 | -0.000001 |
| 3 | N | 3.856357 | 1.263054 | 1.448080 |
| 4 | C | 4.752281 | 1.005656 | 2.431044 |
| 5 | C | 5.060066 | 1.940835 | 3.433301 |
| 6 | H | 5.781867 | 1.711179 | 4.227531 |
| 7 | C | 4.437324 | 3.199844 | 3.360834 |
| 8 | H | 4.664284 | 3.961509 | 4.120101 |
| 9 | C | 3.560737 | 3.499517 | 2.299892 |
| 10 | H | 3.112550 | 4.497814 | 2.209098 |
| 11 | C | 3.289324 | 2.502496 | 1.346546 |
| 12 | N | 5.352674 | -0.250813 | 2.260154 |
| 13 | N | 5.030440 | -0.920930 | 1.098119 |
| 14 | C | 5.774416 | -2.041192 | 1.105694 |
| 15 | C | 6.581881 | -2.100978 | 2.271800 |
| 16 | C | 6.294865 | -0.945712 | 2.985134 |
| 17 | C | 2.464125 | 2.599662 | 0.145925 |
| 18 | N | 2.307943 | 1.442501 | -0.560659 |
| 19 | N | 1.541442 | 1.775566 | -1.637892 |
| 20 | C | 1.234685 | 3.120693 | -1.626236 |
| 21 | C | 1.812338 | 3.682679 | -0.498659 |
| 22 | N | -0.000014 | 0.000008 | -1.733140 |
| 23 | C | 0.799377 | 0.815879 | -2.423871 |
| 24 | C | 0.867432 | 0.850152 | -3.829962 |
| 25 | H | 1.566646 | 1.518391 | -4.351595 |
| 26 | C | -0.000009 | 0.000008 | -4.539279 |
| 27 | H | -0.000006 | 0.000007 | -5.638873 |
| 28 | C | -0.867453 | -0.850137 | -3.829967 |
| 29 | H | -1.566662 | -1.518379 | -4.351602 |
| 30 | C | -0.799403 | -0.815863 | -2.423876 |
| 31 | N | -1.541456 | -1.775557 | -1.637897 |
| 32 | N | -2.307954 | -1.442499 | -0.560663 |
| 33 | C | -2.464124 | -2.599658 | 0.145925 |
| 34 | C | -1.812331 | -3.682671 | -0.498659 |
| 35 | C | -1.234689 | -3.120682 | -1.626241 |
| 36 | N | -3.856356 | -1.263055 | 1.448081 |
| 37 | C | -3.289317 | -2.502494 | 1.346549 |
| 38 | C | -3.560721 | -3.499512 | 2.299901 |
| 39 | H | -3.112531 | -4.497808 | 2.209108 |
| 40 | C | -4.437304 | -3.199838 | 3.360846 |
| 41 | H | -4.664258 | -3.961500 | 4.120118 |
| 42 | C | -5.060051 | -1.940830 | 3.433312 |
| 43 | H | -5.781848 | -1.711174 | 4.227545 |
| 44 | C | -4.752274 | -1.005655 | 2.431048 |
| 45 | N | -5.352671 | 0.250812 | 2.260152 |

| | | | | |
|---|---|---|---|---|
| 46 | N | -5.030444 | 0.920920 | 1.098107 |
| 47 | C | -5.774425 | 2.041179 | 1.105684 |
| 48 | C | -6.581884 | 2.100971 | 2.271793 |
| 49 | C | -6.294861 | 0.945712 | 2.985132 |
| 50 | N | 3.856356 | -1.263054 | -1.448080 |
| 51 | C | 4.752279 | -1.005655 | -2.431043 |
| 52 | C | 5.060065 | -1.940834 | -3.433301 |
| 53 | H | 5.781865 | -1.711178 | -4.227532 |
| 54 | C | 4.437322 | -3.199843 | -3.360834 |
| 55 | H | 4.664283 | -3.961508 | -4.120102 |
| 56 | C | 3.560736 | -3.499516 | -2.299892 |
| 57 | H | 3.112549 | -4.497814 | -2.209098 |
| 58 | C | 3.289323 | -2.502496 | -1.346545 |
| 59 | N | 5.352673 | 0.250814 | -2.260154 |
| 60 | N | 5.030440 | 0.920930 | -1.098118 |
| 61 | C | 5.774417 | 2.041192 | -1.105693 |
| 62 | C | 6.581881 | 2.100978 | -2.271800 |
| 63 | C | 6.294863 | 0.945712 | -2.985134 |
| 64 | C | 2.464125 | -2.599662 | -0.145924 |
| 65 | N | 2.307943 | -1.442501 | 0.560660 |
| 66 | N | 1.541442 | -1.775566 | 1.637893 |
| 67 | C | 1.234686 | -3.120693 | 1.626237 |
| 68 | C | 1.812338 | -3.682679 | 0.498660 |
| 69 | N | -0.000015 | -0.000009 | 1.733140 |
| 70 | C | 0.799378 | -0.815880 | 2.423871 |
| 71 | C | 0.867433 | -0.850152 | 3.829963 |
| 72 | H | 1.566648 | -1.518391 | 4.351595 |
| 73 | C | -0.000008 | -0.000008 | 4.539279 |
| 74 | H | -0.000004 | -0.000007 | 5.638873 |
| 75 | C | -0.867452 | 0.850136 | 3.829968 |
| 76 | H | -1.566660 | 1.518379 | 4.351603 |
| 77 | C | -0.799403 | 0.815862 | 2.423876 |
| 78 | N | -1.541456 | 1.775556 | 1.637897 |
| 79 | N | -2.307954 | 1.442498 | 0.560663 |
| 80 | C | -2.464124 | 2.599658 | -0.145925 |
| 81 | C | -1.812331 | 3.682670 | 0.498659 |
| 82 | C | -1.234688 | 3.120681 | 1.626241 |
| 83 | N | -3.856356 | 1.263055 | -1.448082 |
| 84 | C | -3.289317 | 2.502493 | -1.346549 |
| 85 | C | -3.560721 | 3.499512 | -2.299901 |
| 86 | H | -3.112531 | 4.497808 | -2.209108 |
| 87 | C | -4.437304 | 3.199838 | -3.360846 |
| 88 | H | -4.664257 | 3.961501 | -4.120118 |
| 89 | C | -5.060051 | 1.940831 | -3.433312 |
| 90 | H | -5.781849 | 1.711175 | -4.227545 |
| 91 | C | -4.752274 | 1.005655 | -2.431049 |
| 92 | N | -5.352671 | -0.250812 | -2.260153 |
| 93 | N | -5.030443 | -0.920920 | -1.098109 |
| 94 | C | -5.774424 | -2.041179 | -1.105686 |
| 95 | C | -6.581884 | -2.100971 | -2.271795 |
| 96 | C | -6.294861 | -0.945711 | -2.985133 |
| 97 | H | 0.599740 | -3.555888 | 2.404482 |
| 98 | H | 1.779523 | -4.732853 | 0.194026 |
| 99 | H | 6.688070 | 0.570756 | -3.935758 |
| 100 | H | 5.713817 | 2.759760 | -0.281899 |
| 101 | H | 7.290693 | 2.887644 | -2.549672 |
| 102 | H | -1.779504 | 4.732843 | 0.194023 |
| 103 | H | -0.599741 | 3.555872 | 2.404487 |
| 104 | H | -6.688062 | -0.570759 | -3.935761 |
| 105 | H | -7.290696 | -2.887638 | -2.549665 |
| 106 | H | -5.713830 | -2.759744 | -0.281888 |
| 107 | H | -5.713831 | 2.759743 | 0.281886 |

| | | | | |
|---|---|---|---|---|
| 108 | H | -7.290696 | 2.887638 | 2.549663 |
| 109 | H | -6.688062 | 0.570759 | 3.935760 |
| 110 | H | -1.779505 | -4.732844 | -0.194023 |
| 111 | H | -0.599742 | -3.555873 | -2.404487 |
| 112 | H | 5.713816 | -2.759760 | 0.281900 |
| 113 | H | 7.290693 | -2.887644 | 2.549672 |
| 114 | H | 6.688072 | -0.570755 | 3.935758 |
| 115 | H | 0.599739 | 3.555887 | -2.404480 |
| 116 | H | 1.779522 | 4.732853 | -0.194025 |

$[Fe_2(\mu\text{-}L^2)_2]^{4+}$, mixed-spin

| | | | | |
|---|---|---|---|---|
| 1 | Fe | 3.750246 | 0.000700 | 0.001982 |
| 2 | Fe | -3.569326 | -0.001216 | -0.000747 |
| 3 | N | 3.939736 | 1.377487 | 1.338130 |
| 4 | C | 4.853223 | 1.212320 | 2.325091 |
| 5 | C | 5.165389 | 2.232161 | 3.238982 |
| 6 | H | 5.904785 | 2.080034 | 4.035642 |
| 7 | C | 4.521753 | 3.472140 | 3.072758 |
| 8 | H | 4.750993 | 4.297485 | 3.761619 |
| 9 | C | 3.620104 | 3.670783 | 2.008955 |
| 10 | H | 3.153246 | 4.651201 | 1.846001 |
| 11 | C | 3.350671 | 2.595917 | 1.143697 |
| 12 | N | 5.455667 | -0.053746 | 2.255703 |
| 13 | N | 5.113389 | -0.826082 | 1.164963 |
| 14 | C | 5.849089 | -1.947300 | 1.268274 |
| 15 | C | 6.669809 | -1.906239 | 2.425676 |
| 16 | C | 6.399843 | -0.687539 | 3.032150 |
| 17 | C | 2.502724 | 2.577928 | -0.044962 |
| 18 | N | 2.384279 | 1.366823 | -0.666781 |
| 19 | N | 1.579903 | 1.586214 | -1.740396 |
| 20 | C | 1.207838 | 2.911257 | -1.816522 |
| 21 | C | 1.785154 | 3.580024 | -0.746906 |
| 22 | N | 0.119652 | -0.265604 | -1.785620 |
| 23 | C | 0.924792 | 0.538073 | -2.488219 |
| 24 | C | 1.080274 | 0.483465 | -3.885261 |
| 25 | H | 1.778528 | 1.152853 | -4.406406 |
| 26 | C | 0.296105 | -0.450650 | -4.586186 |
| 27 | H | 0.357663 | -0.518910 | -5.681887 |
| 28 | C | -0.576143 | -1.287323 | -3.872225 |
| 29 | H | -1.224200 | -2.005753 | -4.392980 |
| 30 | C | -0.597549 | -1.162063 | -2.466199 |
| 31 | N | -1.385217 | -2.084528 | -1.690160 |
| 32 | N | -2.143490 | -1.725960 | -0.618208 |
| 33 | C | -2.569214 | -2.899693 | -0.077163 |
| 34 | C | -2.076886 | -4.013066 | -0.811380 |
| 35 | C | -1.332812 | -3.452798 | -1.837642 |
| 36 | N | -4.039003 | -1.642751 | 1.305359 |
| 37 | C | -3.494502 | -2.866545 | 1.065546 |
| 38 | C | -3.847219 | -3.987329 | 1.839635 |
| 39 | H | -3.401501 | -4.971392 | 1.644721 |
| 40 | C | -4.792652 | -3.823107 | 2.869302 |
| 41 | H | -5.092462 | -4.682104 | 3.486616 |
| 42 | C | -5.370854 | -2.565716 | 3.101355 |
| 43 | H | -6.128198 | -2.432546 | 3.883762 |
| 44 | C | -4.957551 | -1.498821 | 2.280562 |
| 45 | N | -5.484134 | -0.190968 | 2.378108 |
| 46 | N | -5.061509 | 0.743898 | 1.471009 |
| 47 | C | -5.724703 | 1.870534 | 1.782925 |
| 48 | C | -6.581152 | 1.672536 | 2.895826 |
| 49 | C | -6.406342 | 0.341735 | 3.251603 |
| 50 | N | 3.936787 | -1.376237 | -1.335869 |

| | | | | |
|---|---|---|---|---|
| 51 | C | 4.850541 | -1.211969 | -2.323231 |
| 52 | C | 5.160609 | -2.232200 | -3.237708 |
| 53 | H | 5.900176 | -2.081039 | -4.034416 |
| 54 | C | 4.514982 | -3.471116 | -3.072123 |
| 55 | H | 4.742729 | -4.296495 | -3.761425 |
| 56 | C | 3.613244 | -3.668646 | -2.008371 |
| 57 | H | 3.144758 | -4.648376 | -1.845866 |
| 58 | C | 3.345769 | -2.593790 | -1.142493 |
| 59 | N | 5.456493 | 0.052794 | -2.254221 |
| 60 | N | 5.117464 | 0.826624 | -1.163723 |
| 61 | C | 5.855269 | 1.946372 | -1.266688 |
| 62 | C | 6.675225 | 1.902857 | -2.424624 |
| 63 | C | 6.402071 | 0.684358 | -3.030869 |
| 64 | C | 2.498420 | -2.575324 | 0.045757 |
| 65 | N | 2.382076 | -1.364433 | 0.667827 |
| 66 | N | 1.577555 | -1.583312 | 1.741643 |
| 67 | C | 1.203995 | -2.907808 | 1.817853 |
| 68 | C | 1.779836 | -3.576805 | 0.747665 |
| 69 | N | 0.117033 | 0.268413 | 1.784977 |
| 70 | C | 0.922589 | -0.534208 | 2.488415 |
| 71 | C | 1.079385 | -0.477004 | 3.885134 |
| 72 | H | 1.777783 | -1.145714 | 4.406935 |
| 73 | C | 0.296332 | 0.458987 | 4.584849 |
| 74 | H | 0.358918 | 0.529448 | 5.680356 |
| 75 | C | -0.576126 | 1.294764 | 3.870039 |
| 76 | H | -1.223313 | 2.014553 | 4.389998 |
| 77 | C | -0.599081 | 1.166644 | 2.464295 |
| 78 | N | -1.387273 | 2.087599 | 1.687096 |
| 79 | N | -2.146168 | 1.727043 | 0.616188 |
| 80 | C | -2.574115 | 2.899729 | 0.074741 |
| 81 | C | -2.082484 | 4.014449 | 0.807471 |
| 82 | C | -1.336325 | 3.456083 | 1.833196 |
| 83 | N | -4.042779 | 1.639749 | -1.306412 |
| 84 | C | -3.500728 | 2.864666 | -1.066886 |
| 85 | C | -3.857006 | 3.984979 | -1.840050 |
| 86 | H | -3.413125 | 4.969922 | -1.645426 |
| 87 | C | -4.803559 | 3.819119 | -2.868404 |
| 88 | H | -5.106164 | 4.677704 | -3.484924 |
| 89 | C | -5.379366 | 2.560566 | -3.100058 |
| 90 | H | -6.137647 | 2.426087 | -3.881331 |
| 91 | C | -4.962474 | 1.494287 | -2.280310 |
| 92 | N | -5.486485 | 0.185389 | -2.377690 |
| 93 | N | -5.061854 | -0.748596 | -1.470643 |
| 94 | C | -5.722951 | -1.876547 | -1.782279 |
| 95 | C | -6.580086 | -1.680259 | -2.894956 |
| 96 | C | -6.407950 | -0.349139 | -3.250854 |
| 97 | H | 0.537148 | -3.254920 | 2.613716 |
| 98 | H | 1.701654 | -4.643276 | 0.517516 |
| 99 | H | 6.807013 | 0.225584 | -3.938821 |
| 100 | H | 5.779204 | 2.738426 | -0.514062 |
| 101 | H | 7.382646 | 2.664790 | -2.767452 |
| 102 | H | -2.262923 | 5.079088 | 0.630981 |
| 103 | H | -0.756367 | 3.921528 | 2.636678 |
| 104 | H | -6.870547 | 0.242680 | -4.046928 |
| 105 | H | -7.244233 | -2.408946 | -3.370906 |
| 106 | H | -5.564980 | -2.788195 | -1.195251 |
| 107 | H | -5.568656 | 2.782554 | 1.195950 |
| 108 | H | -7.246578 | 2.399918 | 3.371985 |
| 109 | H | -6.867616 | -0.251068 | 4.047714 |
| 110 | H | -2.255893 | -5.078088 | -0.635717 |
| 111 | H | -0.753403 | -3.916781 | -2.642370 |
| 112 | H | 5.771082 | -2.739201 | 0.515643 |

| | | | | |
|---|---|---|---|---|
| 113 | H | 7.375646 | -2.669946 | 2.767851 |
| 114 | H | 6.806250 | -0.229784 | 3.939970 |
| 115 | H | 0.540927 | 3.258974 | -2.612069 |
| 116 | H | 1.708409 | 4.646678 | -0.517117 |

$[Fe_2(\mu\text{-}L^2)_2]^{4+}$, high-spin

| | | | | |
|---|---|---|---|---|
| 1 | Fe | 3.708927 | -0.001986 | 0.000969 |
| 2 | Fe | -3.708929 | 0.001986 | 0.000969 |
| 3 | N | 4.161277 | 1.703520 | 1.231305 |
| 4 | C | 5.107889 | 1.625233 | 2.185750 |
| 5 | C | 5.517483 | 2.739370 | 2.943289 |
| 6 | H | 6.298890 | 2.662122 | 3.709446 |
| 7 | C | 4.899732 | 3.969764 | 2.670135 |
| 8 | H | 5.196424 | 4.864088 | 3.236598 |
| 9 | C | 3.916571 | 4.062075 | 1.667101 |
| 10 | H | 3.438062 | 5.024160 | 1.442545 |
| 11 | C | 3.571102 | 2.898007 | 0.956512 |
| 12 | N | 5.659390 | 0.331928 | 2.333042 |
| 13 | N | 5.220897 | -0.656345 | 1.491751 |
| 14 | C | 5.900479 | -1.758935 | 1.849807 |
| 15 | C | 6.784672 | -1.491741 | 2.926270 |
| 16 | C | 6.608606 | -0.143864 | 3.210067 |
| 17 | C | 2.601909 | 2.842527 | -0.148016 |
| 18 | N | 2.244331 | 1.625845 | -0.641630 |
| 19 | N | 1.419930 | 1.893144 | -1.688306 |
| 20 | C | 1.253178 | 3.246772 | -1.867285 |
| 21 | C | 1.992761 | 3.892153 | -0.886608 |
| 22 | N | 0.000011 | -0.000015 | -1.746430 |
| 23 | C | 0.720406 | 0.883438 | -2.442725 |
| 24 | C | 0.785389 | 0.921794 | -3.850712 |
| 25 | H | 1.429330 | 1.642866 | -4.372648 |
| 26 | C | 0.000009 | -0.000019 | -4.561839 |
| 27 | H | 0.000010 | -0.000020 | -5.661334 |
| 28 | C | -0.785371 | -0.921830 | -3.850714 |
| 29 | H | -1.429313 | -1.642902 | -4.372649 |
| 30 | C | -0.720388 | -0.883469 | -2.442727 |
| 31 | N | -1.419917 | -1.893172 | -1.688306 |
| 32 | N | -2.244321 | -1.625863 | -0.641634 |
| 33 | C | -2.601916 | -2.842544 | -0.148021 |
| 34 | C | -1.992774 | -3.892175 | -0.886612 |
| 35 | C | -1.253179 | -3.246802 | -1.867284 |
| 36 | N | -4.161278 | -1.703529 | 1.231310 |
| 37 | C | -3.571109 | -2.898020 | 0.956508 |
| 38 | C | -3.916582 | -4.062088 | 1.667095 |
| 39 | H | -3.438080 | -5.024174 | 1.442534 |
| 40 | C | -4.899737 | -3.969775 | 2.670133 |
| 41 | H | -5.196429 | -4.864100 | 3.236594 |
| 42 | C | -5.517481 | -2.739382 | 2.943297 |
| 43 | H | -6.298882 | -2.662134 | 3.709458 |
| 44 | C | -5.107887 | -1.625241 | 2.185760 |
| 45 | N | -5.659387 | -0.331937 | 2.333059 |
| 46 | N | -5.220907 | 0.656342 | 1.491771 |
| 47 | C | -5.900493 | 1.758930 | 1.849836 |
| 48 | C | -6.784675 | 1.491727 | 2.926301 |
| 49 | C | -6.608601 | 0.143849 | 3.210090 |
| 50 | N | 4.159950 | -1.706655 | -1.230561 |
| 51 | C | 5.106208 | -1.628659 | -2.185351 |
| 52 | C | 5.514449 | -2.742732 | -2.943697 |
| 53 | H | 6.295600 | -2.665774 | -3.710144 |
| 54 | C | 4.895562 | -3.972678 | -2.671098 |
| 55 | H | 5.191183 | -4.866930 | -3.238229 |

| | | | | |
|---|---|---|---|---|
| 56 | C | 3.912463 | -4.064631 | -1.667964 |
| 57 | H | 3.432940 | -5.026357 | -1.444022 |
| 58 | C | 3.568372 | -2.900692 | -0.956506 |
| 59 | N | 5.658495 | -0.335620 | -2.332207 |
| 60 | N | 5.219709 | 0.652847 | -1.491172 |
| 61 | C | 5.899833 | 1.755152 | -1.849066 |
| 62 | C | 6.784499 | 1.487588 | -2.925190 |
| 63 | C | 6.608170 | 0.139769 | -3.208972 |
| 64 | C | 2.599051 | -2.844643 | 0.147808 |
| 65 | N | 2.243389 | -1.627647 | 0.642419 |
| 66 | N | 1.418321 | -1.894274 | 1.688622 |
| 67 | C | 1.248960 | -3.247784 | 1.866172 |
| 68 | C | 1.987732 | -3.893667 | 0.885262 |
| 69 | N | 0.000012 | 0.000017 | 1.746768 |
| 70 | C | 0.719738 | -0.883944 | 2.443094 |
| 71 | C | 0.784750 | -0.922309 | 3.851084 |
| 72 | H | 1.428156 | -1.643863 | 4.373024 |
| 73 | C | 0.000004 | 0.000015 | 4.562230 |
| 74 | H | 0.000002 | 0.000013 | 5.661725 |
| 75 | C | -0.784740 | 0.922339 | 3.851087 |
| 76 | H | -1.428149 | 1.643891 | 4.373024 |
| 77 | C | -0.719720 | 0.883974 | 2.443096 |
| 78 | N | -1.418307 | 1.894302 | 1.688624 |
| 79 | N | -2.243378 | 1.627666 | 0.642424 |
| 80 | C | -2.599056 | 2.844660 | 0.147813 |
| 81 | C | -1.987744 | 3.893689 | 0.885267 |
| 82 | C | -1.248960 | 3.247814 | 1.866174 |
| 83 | N | -4.159951 | 1.706666 | -1.230565 |
| 84 | C | -3.568376 | 2.900705 | -0.956503 |
| 85 | C | -3.912469 | 4.064644 | -1.667960 |
| 86 | H | -3.432952 | 5.026371 | -1.444013 |
| 87 | C | -4.895561 | 3.972690 | -2.671099 |
| 88 | H | -5.191180 | 4.866941 | -3.238229 |
| 89 | C | -5.514441 | 2.742744 | -2.943706 |
| 90 | H | -6.295587 | 2.665786 | -3.710158 |
| 91 | C | -5.106204 | 1.628668 | -2.185361 |
| 92 | N | -5.658492 | 0.335630 | -2.332222 |
| 93 | N | -5.219720 | -0.652842 | -1.491189 |
| 94 | C | -5.899849 | -1.755145 | -1.849090 |
| 95 | C | -6.784503 | -1.487572 | -2.925216 |
| 96 | C | -6.608164 | -0.139753 | -3.208993 |
| 97 | H | 0.602548 | -3.640076 | 2.658041 |
| 98 | H | 2.086292 | -4.973784 | 0.742537 |
| 99 | H | 7.084527 | -0.500939 | -3.957616 |
| 100 | H | 5.736938 | 2.699987 | -1.318158 |
| 101 | H | 7.467829 | 2.182227 | -3.424177 |
| 102 | H | -2.086314 | 4.973805 | 0.742543 |
| 103 | H | -0.602549 | 3.640112 | 2.658039 |
| 104 | H | -7.084513 | 0.500959 | -3.957638 |
| 105 | H | -7.467835 | -2.182203 | -3.424211 |
| 106 | H | -5.736961 | -2.699981 | -1.318182 |
| 107 | H | -5.737557 | 2.703609 | 1.318677 |
| 108 | H | -7.467581 | 2.186560 | 3.425595 |
| 109 | H | -7.084800 | -0.496615 | 3.959042 |
| 110 | H | -2.093229 | -4.972223 | -0.744697 |
| 111 | H | -0.607718 | -3.639565 | -2.659689 |
| 112 | H | 5.737538 | -2.703611 | 1.318647 |
| 113 | H | 7.467578 | -2.186581 | 3.425557 |
| 114 | H | 7.084812 | 0.496597 | 3.959019 |
| 115 | H | 0.607717 | 3.639528 | -2.659694 |
| 116 | H | 2.093204 | 4.972202 | -0.744692 |

[$Fe_2$($\mu$-$L^3$)$_2$]$^{4+}$, low-spin

| | | | | |
|---|---|---|---|---|
| 1 | Fe | -0.182767 | 0.004188 | -4.259177 |
| 2 | Fe | 0.182733 | -0.033394 | 4.261510 |
| 3 | C | -2.331683 | -0.140481 | 1.517564 |
| 4 | C | -2.675692 | 0.243022 | -1.546747 |
| 5 | H | -2.428741 | -0.780093 | 2.413375 |
| 6 | H | -3.001428 | 0.775958 | -2.459229 |
| 7 | N | -1.745903 | 1.139321 | 1.977434 |
| 8 | N | -0.719660 | 1.163745 | 2.865117 |
| 9 | C | -0.284008 | 2.462743 | 2.894448 |
| 10 | C | -1.060127 | 3.267215 | 2.026982 |
| 11 | C | -1.979376 | 2.387850 | 1.461925 |
| 12 | H | -2.795213 | 2.559253 | 0.753737 |
| 13 | H | -0.985733 | 4.344735 | 1.854739 |
| 14 | C | 0.829692 | 2.731421 | 3.797597 |
| 15 | C | 1.471256 | 3.956491 | 4.052754 |
| 16 | C | 2.510342 | 3.990184 | 5.001754 |
| 17 | C | 2.879589 | 2.823847 | 5.696721 |
| 18 | C | 2.186595 | 1.641096 | 5.392882 |
| 19 | N | 1.215054 | 1.591923 | 4.448165 |
| 20 | H | 3.666069 | 2.846662 | 6.462006 |
| 21 | H | 3.025012 | 4.936327 | 5.221119 |
| 22 | H | 1.155542 | 4.870783 | 3.533512 |
| 23 | N | 2.358061 | 0.396325 | 6.018613 |
| 24 | N | 1.477057 | -0.596104 | 5.643973 |
| 25 | C | 1.806293 | -1.672780 | 6.379079 |
| 26 | C | 2.900966 | -1.381060 | 7.234624 |
| 27 | C | 3.229355 | -0.056241 | 6.983997 |
| 28 | H | 4.003261 | 0.588633 | 7.412596 |
| 29 | H | 3.385589 | -2.055569 | 7.947636 |
| 30 | H | 1.253615 | -2.612695 | 6.276646 |
| 31 | N | -0.835267 | -1.671040 | 4.426169 |
| 32 | C | -1.808981 | -1.738538 | 5.367256 |
| 33 | C | -2.478418 | -2.935025 | 5.669856 |
| 34 | C | -2.079137 | -4.094819 | 4.980052 |
| 35 | C | -1.033754 | -4.042180 | 4.038785 |
| 36 | C | -0.416937 | -2.804376 | 3.783588 |
| 37 | H | -0.692450 | -4.951461 | 3.526971 |
| 38 | H | -2.573947 | -5.051455 | 5.199633 |
| 39 | H | -3.267261 | -2.973904 | 6.432015 |
| 40 | N | -2.004289 | -0.497069 | 5.993815 |
| 41 | N | -1.132692 | 0.508664 | 5.631452 |
| 42 | C | -1.481172 | 1.577063 | 6.369468 |
| 43 | C | -2.579005 | 1.267290 | 7.214586 |
| 44 | C | -2.888494 | -0.060398 | 6.954884 |
| 45 | H | -0.938356 | 2.523794 | 6.276536 |
| 46 | H | -3.077337 | 1.931935 | 7.927418 |
| 47 | H | -3.656871 | -0.717223 | 7.375282 |
| 48 | C | 0.700203 | -2.512568 | 2.891225 |
| 49 | C | 1.495301 | -3.290532 | 2.016861 |
| 50 | N | 1.102714 | -1.202943 | 2.867073 |
| 51 | C | 2.385173 | -2.382139 | 1.448598 |
| 52 | N | 2.118196 | -1.143852 | 1.971974 |
| 53 | H | 1.447267 | -4.367717 | 1.833353 |
| 54 | H | 3.195380 | -2.523603 | 0.727329 |
| 55 | C | 2.672459 | 0.162665 | 1.546793 |
| 56 | C | 2.333971 | -0.184908 | -1.524279 |
| 57 | H | 2.424920 | -0.817003 | -2.425924 |
| 58 | H | 3.003393 | 0.688043 | 2.461634 |
| 59 | N | -2.147098 | -1.072819 | -1.976257 |
| 60 | N | -1.133152 | -1.151370 | -2.872362 |

| | | | | |
|---|---|---|---|---|
| 61 | C | -0.766848 | -2.471267 | -2.908424 |
| 62 | C | -1.582817 | -3.235104 | -2.040676 |
| 63 | C | -2.446879 | -2.307816 | -1.463118 |
| 64 | H | -3.260236 | -2.433845 | -0.742517 |
| 65 | H | -1.564163 | -4.314773 | -1.866692 |
| 66 | N | 1.779637 | 1.112737 | -1.973299 |
| 67 | N | 0.751283 | 1.169226 | -2.857290 |
| 68 | C | 0.349441 | 2.479180 | -2.877633 |
| 69 | C | 2.045783 | 2.351202 | -1.449643 |
| 70 | C | 1.148534 | 3.257733 | -2.007245 |
| 71 | H | 2.866941 | 2.496950 | -0.741770 |
| 72 | H | 1.102844 | 4.335751 | -1.828091 |
| 73 | N | -1.171583 | 1.657444 | -4.436933 |
| 74 | C | -0.757883 | 2.782101 | -3.777884 |
| 75 | C | -1.367731 | 4.024901 | -4.024987 |
| 76 | C | -2.140054 | 1.738053 | -5.382469 |
| 77 | C | 0.340651 | -2.786310 | -3.804769 |
| 78 | N | 0.789470 | -1.659811 | -4.438753 |
| 79 | C | 1.759173 | -1.746733 | -5.382220 |
| 80 | C | 2.395382 | -2.958722 | -5.694972 |
| 81 | C | 0.922446 | -4.038700 | -4.070885 |
| 82 | C | 1.965142 | -4.112543 | -5.013686 |
| 83 | C | -2.802266 | 2.940225 | -5.678741 |
| 84 | C | -2.404236 | 4.091819 | -4.975052 |
| 85 | H | 0.556289 | -4.942351 | -3.566301 |
| 86 | H | 2.433233 | -5.080643 | -5.241393 |
| 87 | H | 3.182238 | -3.013324 | -6.458182 |
| 88 | H | -3.586728 | 2.988730 | -6.444839 |
| 89 | H | -2.894054 | 5.052388 | -5.188510 |
| 90 | H | -1.029059 | 4.927130 | -3.499167 |
| 91 | N | -2.342337 | 0.502486 | -6.017118 |
| 92 | N | -1.488687 | -0.515454 | -5.646803 |
| 93 | C | -1.843903 | -1.577910 | -6.390692 |
| 94 | C | -2.927712 | -1.251390 | -7.247435 |
| 95 | C | -3.221884 | 0.079802 | -6.988450 |
| 96 | H | -3.427371 | -1.907981 | -7.966751 |
| 97 | H | -1.316417 | -2.532836 | -6.294265 |
| 98 | H | -3.977108 | 0.747563 | -7.415430 |
| 99 | N | 1.987953 | -0.506062 | -5.999003 |
| 100 | N | 1.146489 | 0.521291 | -5.625450 |
| 101 | C | 1.523966 | 1.585735 | -6.354997 |
| 102 | C | 2.610841 | 1.251991 | -7.205203 |
| 103 | C | 2.882348 | -0.086391 | -6.958146 |
| 104 | H | 3.630782 | -0.761030 | -7.386290 |
| 105 | H | 3.126603 | 1.908383 | -7.913255 |
| 106 | H | 1.008621 | 2.546757 | -6.252800 |
| 107 | H | -1.586869 | -0.629197 | 0.856652 |
| 108 | H | -1.817539 | 0.811762 | -1.134075 |
| 109 | H | 1.573620 | -0.664758 | -0.874779 |
| 110 | H | 1.825784 | 0.747343 | 1.132476 |
| 111 | C | -3.841422 | 0.161162 | -0.572407 |
| 112 | C | 3.840176 | 0.060792 | 0.577214 |
| 113 | C | 3.685658 | -0.071444 | -0.836100 |
| 114 | C | -3.684152 | 0.009899 | 0.838564 |
| 115 | C | 4.842268 | -0.114826 | -1.646332 |
| 116 | H | 4.730601 | -0.217308 | -2.736521 |
| 117 | C | 5.145427 | 0.129054 | 1.115220 |
| 118 | H | 5.273173 | 0.230484 | 2.203611 |
| 119 | H | 7.286928 | 0.138917 | 0.741308 |
| 120 | C | 6.284055 | 0.081459 | 0.295241 |
| 121 | H | 7.013582 | -0.074286 | -1.749772 |
| 122 | C | 6.131176 | -0.037330 | -1.095286 |

| | | | | |
|---|---|---|---|---|
| 123 | C | -5.146982 | 0.269794 | -1.103061 |
| 124 | H | -5.277271 | 0.387229 | -2.189575 |
| 125 | C | -4.837837 | -0.014774 | 1.653762 |
| 126 | H | -4.723872 | -0.132966 | 2.742152 |
| 127 | H | -7.285847 | 0.331204 | -0.719020 |
| 128 | C | -6.282815 | 0.242175 | -0.278169 |
| 129 | H | -7.007100 | 0.079234 | 1.768089 |
| 130 | C | -6.126980 | 0.101980 | 1.109902 |

$[Fe_2(\mu\text{-}L^3)_2]^{4+}$, mixed-spin

| | | | | |
|---|---|---|---|---|
| 1 | Fe | -0.204846 | 0.064143 | -4.495387 |
| 2 | Fe | 0.202857 | -0.054615 | 4.384803 |
| 3 | C | -2.094888 | 0.146899 | 1.239990 |
| 4 | C | -2.693187 | 0.406505 | -1.787468 |
| 5 | H | -2.044993 | -0.636727 | 2.018985 |
| 6 | H | -3.092789 | 0.897173 | -2.694789 |
| 7 | N | -1.647145 | 1.398497 | 1.889006 |
| 8 | N | -0.702724 | 1.381601 | 2.860550 |
| 9 | C | -0.432750 | 2.690360 | 3.134942 |
| 10 | C | -1.219853 | 3.542498 | 2.321137 |
| 11 | C | -1.983765 | 2.677552 | 1.540860 |
| 12 | H | -2.754636 | 2.875993 | 0.789693 |
| 13 | H | -1.256827 | 4.635703 | 2.316647 |
| 14 | C | 0.553230 | 2.992574 | 4.182146 |
| 15 | C | 0.984941 | 4.283626 | 4.537333 |
| 16 | C | 1.929926 | 4.414601 | 5.571571 |
| 17 | C | 2.429787 | 3.276114 | 6.223555 |
| 18 | C | 1.946043 | 2.023463 | 5.801694 |
| 19 | N | 1.038422 | 1.890136 | 4.815973 |
| 20 | H | 3.163448 | 3.372296 | 7.033564 |
| 21 | H | 2.279230 | 5.411430 | 5.876475 |
| 22 | H | 0.590849 | 5.171213 | 4.025814 |
| 23 | N | 2.355820 | 0.792806 | 6.366828 |
| 24 | N | 1.797667 | -0.360721 | 5.881081 |
| 25 | C | 2.373297 | -1.358162 | 6.574093 |
| 26 | C | 3.310820 | -0.857440 | 7.512319 |
| 27 | C | 3.273971 | 0.522257 | 7.356394 |
| 28 | H | 3.827558 | 1.313103 | 7.872381 |
| 29 | H | 3.929492 | -1.424902 | 8.214823 |
| 30 | H | 2.091557 | -2.398972 | 6.377405 |
| 31 | N | -0.626733 | -2.004641 | 4.807394 |
| 32 | C | -1.502635 | -2.156249 | 5.818530 |
| 33 | C | -1.967044 | -3.416590 | 6.238315 |
| 34 | C | -1.481810 | -4.543946 | 5.556093 |
| 35 | C | -0.569641 | -4.394226 | 4.495301 |
| 36 | C | -0.155277 | -3.096217 | 4.144584 |
| 37 | H | -0.185407 | -5.272554 | 3.960800 |
| 38 | H | -1.816155 | -5.546565 | 5.858719 |
| 39 | H | -2.674017 | -3.527222 | 7.069917 |
| 40 | N | -1.899130 | -0.934258 | 6.412353 |
| 41 | N | -1.356199 | 0.226377 | 5.927150 |
| 42 | C | -1.913434 | 1.213893 | 6.649088 |
| 43 | C | -2.823689 | 0.698996 | 7.606145 |
| 44 | C | -2.789385 | -0.678649 | 7.430675 |
| 45 | H | -1.638653 | 2.257671 | 6.458475 |
| 46 | H | -3.422820 | 1.255815 | 8.333638 |
| 47 | H | -3.327164 | -1.477498 | 7.951039 |
| 48 | C | 0.798400 | -2.771047 | 3.074905 |
| 49 | C | 1.556147 | -3.598852 | 2.210911 |
| 50 | N | 1.063710 | -1.454888 | 2.830140 |
| 51 | C | 2.297531 | -2.712102 | 1.431994 |

| | | | | |
|---|---|---|---|---|
| 52 | N | 1.975110 | -1.443754 | 1.828521 |
| 53 | H | 1.587904 | -4.691419 | 2.169353 |
| 54 | H | 3.039638 | -2.889495 | 0.647826 |
| 55 | C | 2.432721 | -0.161646 | 1.244819 |
| 56 | C | 2.345615 | -0.390163 | -1.842267 |
| 57 | H | 2.495232 | -0.944315 | -2.786849 |
| 58 | H | 2.639435 | 0.514586 | 2.096037 |
| 59 | N | -2.184243 | -0.926162 | -2.190644 |
| 60 | N | -1.202374 | -1.053193 | -3.116330 |
| 61 | C | -0.908777 | -2.391002 | -3.168924 |
| 62 | C | -1.734051 | -3.114555 | -2.276590 |
| 63 | C | -2.531901 | -2.146566 | -1.672144 |
| 64 | H | -3.326239 | -2.233993 | -0.925312 |
| 65 | H | -1.762960 | -4.193841 | -2.101741 |
| 66 | N | 1.849792 | 0.957813 | -2.210138 |
| 67 | N | 0.818611 | 1.115955 | -3.076780 |
| 68 | C | 0.504185 | 2.449614 | -3.044986 |
| 69 | C | 2.202510 | 2.155374 | -1.643786 |
| 70 | C | 1.361618 | 3.139685 | -2.155697 |
| 71 | H | 3.036531 | 2.219292 | -0.938921 |
| 72 | H | 1.388022 | 4.209893 | -1.931575 |
| 73 | N | -1.083662 | 1.781447 | -4.611267 |
| 74 | C | -0.593319 | 2.853523 | -3.917987 |
| 75 | C | -1.128379 | 4.139482 | -4.111308 |
| 76 | C | -2.059031 | 1.954461 | -5.536353 |
| 77 | C | 0.152545 | -2.759608 | -4.099356 |
| 78 | N | 0.650559 | -1.653682 | -4.732386 |
| 79 | C | 1.592597 | -1.784679 | -5.698326 |
| 80 | C | 2.147287 | -3.027969 | -6.041670 |
| 81 | C | 0.650656 | -4.040808 | -4.395914 |
| 82 | C | 1.663453 | -4.163699 | -5.365786 |
| 83 | C | -2.650131 | 3.204768 | -5.779178 |
| 84 | C | -2.171410 | 4.303955 | -5.042288 |
| 85 | H | 0.243177 | -4.927498 | -3.892907 |
| 86 | H | 2.066753 | -5.154755 | -5.617375 |
| 87 | H | 2.913226 | -3.118373 | -6.822446 |
| 88 | H | -3.441503 | 3.328316 | -6.529361 |
| 89 | H | -2.603998 | 5.299657 | -5.214107 |
| 90 | H | -0.726939 | 4.999267 | -3.559186 |
| 91 | N | -2.347785 | 0.755279 | -6.206647 |
| 92 | N | -1.561798 | -0.329086 | -5.874931 |
| 93 | C | -1.991835 | -1.341515 | -6.648476 |
| 94 | C | -3.057486 | -0.917038 | -7.484756 |
| 95 | C | -3.260325 | 0.422040 | -7.182571 |
| 96 | H | -3.604931 | -1.516264 | -8.219171 |
| 97 | H | -1.527341 | -2.331463 | -6.586897 |
| 98 | H | -3.972131 | 1.150535 | -7.584275 |
| 99 | N | 1.888428 | -0.548752 | -6.296135 |
| 100 | N | 1.132750 | 0.525308 | -5.873014 |
| 101 | C | 1.566870 | 1.580282 | -6.584504 |
| 102 | C | 2.605645 | 1.194281 | -7.471745 |
| 103 | C | 2.786740 | -0.166457 | -7.267071 |
| 104 | H | 3.474750 | -0.879627 | -7.732492 |
| 105 | H | 3.149582 | 1.831994 | -8.175720 |
| 106 | H | 1.124086 | 2.571682 | -6.441701 |
| 107 | H | -1.349784 | -0.125169 | 0.464692 |
| 108 | H | -1.818171 | 1.001938 | -1.458104 |
| 109 | H | 1.532040 | -0.905891 | -1.291814 |
| 110 | H | 1.579671 | 0.276853 | 0.689168 |
| 111 | C | -3.778603 | 0.344068 | -0.724238 |
| 112 | C | 3.677420 | -0.289331 | 0.379785 |
| 113 | C | 3.639584 | -0.369535 | -1.044081 |

| | | | | |
|---|---|---|---|---|
| 114 | C | -3.503282 | 0.237074 | 0.671087 |
| 115 | C | 4.858969 | -0.440671 | -1.755358 |
| 116 | H | 4.837917 | -0.496613 | -2.854261 |
| 117 | C | 4.931756 | -0.304940 | 1.031374 |
| 118 | H | 4.968023 | -0.244330 | 2.129871 |
| 119 | H | 7.095309 | -0.397231 | 0.841442 |
| 120 | C | 6.132718 | -0.388282 | 0.310219 |
| 121 | H | 7.029783 | -0.510972 | -1.670051 |
| 122 | C | 6.096276 | -0.451060 | -1.092675 |
| 123 | C | -5.126051 | 0.401399 | -1.149102 |
| 124 | H | -5.349316 | 0.483128 | -2.223689 |
| 125 | C | -4.583707 | 0.201448 | 1.580861 |
| 126 | H | -4.374690 | 0.120353 | 2.658669 |
| 127 | H | -7.225996 | 0.414298 | -0.590393 |
| 128 | C | -6.187934 | 0.365176 | -0.232062 |
| 129 | H | -6.737729 | 0.237768 | 1.870873 |
| 130 | C | -5.914786 | 0.267156 | 1.142634 |

$[Fe_2(\mu\text{-}L^3)_2]^{4+}$, high-spin

| | | | | |
|---|---|---|---|---|
| 1 | Fe | -0.222182 | 0.062413 | -4.594204 |
| 2 | Fe | 0.203793 | -0.065007 | 4.566186 |
| 3 | C | -2.082743 | -0.026014 | 1.417322 |
| 4 | C | -2.633459 | 0.179261 | -1.632592 |
| 5 | H | -2.022593 | -0.794910 | 2.210080 |
| 6 | H | -3.006157 | 0.730867 | -2.517668 |
| 7 | N | -1.704824 | 1.256295 | 2.054260 |
| 8 | N | -0.771980 | 1.302676 | 3.035797 |
| 9 | C | -0.581186 | 2.627596 | 3.301573 |
| 10 | C | -1.406342 | 3.425986 | 2.471395 |
| 11 | C | -2.109813 | 2.511164 | 1.690478 |
| 12 | H | -2.879778 | 2.659022 | 0.927123 |
| 13 | H | -1.505777 | 4.515941 | 2.454565 |
| 14 | C | 0.374604 | 2.993770 | 4.355932 |
| 15 | C | 0.726646 | 4.309706 | 4.707382 |
| 16 | C | 1.654744 | 4.501003 | 5.747095 |
| 17 | C | 2.217536 | 3.397124 | 6.407367 |
| 18 | C | 1.812326 | 2.116183 | 5.988428 |
| 19 | N | 0.920165 | 1.924931 | 4.998473 |
| 20 | H | 2.938708 | 3.539633 | 7.221392 |
| 21 | H | 1.942023 | 5.518138 | 6.049293 |
| 22 | H | 0.284718 | 5.169845 | 4.188042 |
| 23 | N | 2.292532 | 0.913966 | 6.559905 |
| 24 | N | 1.808849 | -0.272054 | 6.073467 |
| 25 | C | 2.442353 | -1.231519 | 6.769165 |
| 26 | C | 3.343645 | -0.673421 | 7.710514 |
| 27 | C | 3.221952 | 0.701538 | 7.553322 |
| 28 | H | 3.723875 | 1.525460 | 8.070562 |
| 29 | H | 3.994270 | -1.200842 | 8.415770 |
| 30 | H | 2.226703 | -2.287641 | 6.571932 |
| 31 | N | -0.499970 | -2.067886 | 4.966746 |
| 32 | C | -1.357636 | -2.289625 | 5.979783 |
| 33 | C | -1.738368 | -3.583139 | 6.382254 |
| 34 | C | -1.187458 | -4.666691 | 5.679166 |
| 35 | C | -0.294094 | -4.443697 | 4.615727 |
| 36 | C | 0.035657 | -3.116934 | 4.283995 |
| 37 | H | 0.140467 | -5.287650 | 4.064457 |
| 38 | H | -1.455853 | -5.693449 | 5.967694 |
| 39 | H | -2.431240 | -3.751258 | 7.216427 |
| 40 | N | -1.827411 | -1.104149 | 6.593364 |
| 41 | N | -1.373210 | 0.096159 | 6.113051 |
| 42 | C | -1.989323 | 1.035005 | 6.851310 |

| | | | | |
|---|---|---|---|---|
| 43 | C | -2.849350 | 0.448986 | 7.814152 |
| 44 | C | -2.720419 | -0.921732 | 7.625259 |
| 45 | H | -1.791643 | 2.097068 | 6.667549 |
| 46 | H | -3.477614 | 0.955909 | 8.553460 |
| 47 | H | -3.193872 | -1.760805 | 8.143613 |
| 48 | C | 0.957767 | -2.712930 | 3.213591 |
| 49 | C | 1.746041 | -3.472615 | 2.315392 |
| 50 | N | 1.148370 | -1.377432 | 3.003087 |
| 51 | C | 2.427502 | -2.525358 | 1.553621 |
| 52 | N | 2.042243 | -1.287534 | 1.989706 |
| 53 | H | 1.834562 | -4.559729 | 2.238731 |
| 54 | H | 3.167235 | -2.641582 | 0.755852 |
| 55 | C | 2.440793 | 0.034810 | 1.450902 |
| 56 | C | 2.256293 | -0.121255 | -1.639826 |
| 57 | H | 2.344721 | -0.770515 | -2.531930 |
| 58 | H | 2.662554 | 0.677121 | 2.324106 |
| 59 | N | -2.183523 | -1.151146 | -2.111484 |
| 60 | N | -1.232789 | -1.247398 | -3.071117 |
| 61 | C | -1.067460 | -2.583590 | -3.297510 |
| 62 | C | -1.929014 | -3.337499 | -2.464402 |
| 63 | C | -2.632754 | -2.385418 | -1.728987 |
| 64 | H | -3.425988 | -2.496589 | -0.983922 |
| 65 | H | -2.052450 | -4.423283 | -2.415724 |
| 66 | N | 1.839905 | 1.213375 | -2.133269 |
| 67 | N | 0.856045 | 1.331842 | -3.056658 |
| 68 | C | 0.693237 | 2.672749 | -3.253820 |
| 69 | C | 2.306483 | 2.437141 | -1.739642 |
| 70 | C | 1.586966 | 3.406531 | -2.435603 |
| 71 | H | 3.124599 | 2.528044 | -1.019146 |
| 72 | H | 1.721436 | 4.490222 | -2.374549 |
| 73 | N | -0.879567 | 2.088200 | -4.930496 |
| 74 | C | -0.292014 | 3.111791 | -4.252209 |
| 75 | C | -0.621576 | 4.450892 | -4.530326 |
| 76 | C | -1.789980 | 2.348121 | -5.887656 |
| 77 | C | -0.108719 | -2.992946 | -4.332214 |
| 78 | N | 0.443638 | -1.948134 | -5.008688 |
| 79 | C | 1.325751 | -2.177335 | -5.999737 |
| 80 | C | 1.714846 | -3.473728 | -6.385096 |
| 81 | C | 0.229734 | -4.322008 | -4.646698 |
| 82 | C | 1.147925 | -4.552617 | -5.687317 |
| 83 | C | -2.174947 | 3.656807 | -6.233628 |
| 84 | C | -1.570836 | 4.714170 | -5.534796 |
| 85 | H | -0.217195 | -5.161893 | -4.099118 |
| 86 | H | 1.423885 | -5.580805 | -5.961477 |
| 87 | H | 2.426455 | -3.647109 | -7.201821 |
| 88 | H | -2.911689 | 3.854967 | -7.021800 |
| 89 | H | -1.841352 | 5.751078 | -5.780021 |
| 90 | H | -0.144951 | 5.273997 | -3.982451 |
| 91 | N | -2.308373 | 1.186778 | -6.508149 |
| 92 | N | -1.848952 | -0.033435 | -6.085058 |
| 93 | C | -2.512125 | -0.941724 | -6.820546 |
| 94 | C | -3.408431 | -0.315877 | -7.724300 |
| 95 | C | -3.252139 | 1.046059 | -7.501017 |
| 96 | H | -4.077484 | -0.792169 | -8.448021 |
| 97 | H | -2.321228 | -2.011682 | -6.677994 |
| 98 | H | -3.737385 | 1.906571 | -7.972464 |
| 99 | N | 1.810392 | -0.995447 | -6.609665 |
| 100 | N | 1.354032 | 0.208160 | -6.140215 |
| 101 | C | 1.985652 | 1.142432 | -6.870954 |
| 102 | C | 2.857663 | 0.549924 | -7.819003 |
| 103 | C | 2.720273 | -0.819157 | -7.627747 |
| 104 | H | 3.197155 | -1.661997 | -8.137666 |

| | | | | |
|---|---|---|---|---|
| 105 | H | 3.498530 | 1.051926 | -8.550831 |
| 106 | H | 1.789652 | 2.206258 | -6.693125 |
| 107 | H | -1.309120 | -0.280074 | 0.664508 |
| 108 | H | -1.735476 | 0.721727 | -1.275018 |
| 109 | H | 1.428756 | -0.534085 | -1.028528 |
| 110 | H | 1.557251 | 0.474978 | 0.945389 |
| 111 | C | -3.731879 | 0.106943 | -0.583336 |
| 112 | C | 3.656950 | -0.028826 | 0.539891 |
| 113 | C | 3.573491 | -0.098145 | -0.881745 |
| 114 | C | -3.479518 | 0.005393 | 0.816087 |
| 115 | C | 4.770117 | -0.142566 | -1.633709 |
| 116 | H | 4.713626 | -0.194354 | -2.731221 |
| 117 | C | 4.932364 | -0.019774 | 1.149642 |
| 118 | H | 5.002427 | 0.031995 | 2.247201 |
| 119 | H | 7.088874 | -0.056712 | 0.887709 |
| 120 | C | 6.109791 | -0.068623 | 0.388732 |
| 121 | H | 6.942851 | -0.167335 | -1.621465 |
| 122 | C | 6.027915 | -0.128480 | -1.013038 |
| 123 | C | -5.073553 | 0.140735 | -1.031378 |
| 124 | H | -5.279290 | 0.223940 | -2.109752 |
| 125 | C | -4.575410 | -0.055225 | 1.706467 |
| 126 | H | -4.384218 | -0.130869 | 2.788415 |
| 127 | H | -7.182834 | 0.111463 | -0.510085 |
| 128 | C | -6.150408 | 0.079653 | -0.133983 |
| 129 | H | -6.734026 | -0.068151 | 1.958710 |
| 130 | C | -5.899372 | -0.018855 | 1.245056 |

[Cr($L^1$)]$^0$, low-spin

| | | | | |
|---|---|---|---|---|
| 1 | Cr | -0.0000006 | 0.0000003 | -0.4044694 |
| 2 | N | -1.9415970 | -0.1578734 | -0.7263176 |
| 3 | C | -2.8536238 | 0.7249909 | -0.1386556 |
| 4 | C | -4.2093496 | 0.7452008 | -0.5233691 |
| 5 | C | -4.6634102 | -0.1376153 | -1.5179837 |
| 6 | H | -5.7184986 | -0.1402802 | -1.8234371 |
| 7 | C | -3.7448489 | -1.0171566 | -2.1392316 |
| 8 | H | -4.0643407 | -1.7042749 | -2.9332408 |
| 9 | C | -2.4089592 | -0.9779232 | -1.7271791 |
| 10 | C | -2.1896490 | 1.5002459 | 0.8715537 |
| 11 | N | -0.8420424 | 1.2265990 | 0.9853901 |
| 12 | N | -0.4079366 | 1.9289231 | 2.0821154 |
| 13 | C | -1.4462830 | 2.6452591 | 2.6438122 |
| 14 | H | -1.2815972 | 3.2607328 | 3.5343853 |
| 15 | C | -2.5892721 | 2.4149249 | 1.8945454 |
| 16 | H | -3.5829794 | 2.8413150 | 2.0579625 |
| 17 | N | -1.3713585 | -1.7439287 | -2.2634340 |
| 18 | N | -0.1086345 | -1.4642335 | -1.7808690 |
| 19 | C | 0.7270988 | -2.3099138 | -2.4343875 |
| 20 | H | 1.8014042 | -2.2811829 | -2.2292352 |
| 21 | C | 0.0002901 | -3.1344279 | -3.3264379 |
| 22 | H | 0.3998925 | -3.9121636 | -3.9843248 |
| 23 | C | -1.3351971 | -2.7571214 | -3.2005229 |
| 24 | H | -2.2458704 | -3.1212105 | -3.6820190 |
| 25 | N | 1.9415965 | 0.1578744 | -0.7263180 |
| 26 | C | 2.4089591 | 0.9779236 | -1.7271788 |
| 27 | C | 3.7448466 | 1.0171584 | -2.1392308 |
| 28 | H | 4.0643392 | 1.7042764 | -2.9332394 |
| 29 | C | 4.6634063 | 0.1376170 | -1.5179818 |
| 30 | H | 5.7184948 | 0.1402822 | -1.8234346 |
| 31 | C | 4.2093474 | -0.7451995 | -0.5233675 |
| 32 | C | 2.8536233 | -0.7249889 | -0.1386556 |
| 33 | N | 1.3713592 | 1.7439288 | -2.2634349 |
| 34 | N | 0.1086349 | 1.4642328 | -1.7808705 |
| 35 | C | -0.7270992 | 2.3099116 | -2.4343902 |
| 36 | H | -1.8014051 | 2.2811808 | -2.2292397 |
| 37 | C | -0.0002879 | 3.1344264 | -3.3264418 |
| 38 | H | -0.3998907 | 3.9121613 | -3.9843296 |
| 39 | C | 1.3351993 | 2.7571205 | -3.2005245 |
| 40 | H | 2.2458728 | 3.1212093 | -3.6820198 |
| 41 | C | 2.1896494 | -1.5002443 | 0.8715527 |
| 42 | N | 0.8420431 | -1.2265988 | 0.9853886 |
| 43 | N | 0.4079374 | -1.9289232 | 2.0821132 |
| 44 | C | 1.4462838 | -2.6452584 | 2.6438093 |
| 45 | H | 1.2815990 | -3.2607331 | 3.5343819 |
| 46 | C | 2.5892726 | -2.4149227 | 1.8945428 |
| 47 | H | 3.5829797 | -2.8413127 | 2.0579601 |
| 48 | H | -4.8971209 | 1.4503556 | -0.0362816 |
| 49 | H | 4.8971190 | -1.4503537 | -0.0362806 |
| 50 | C | -0.8133727 | -1.4889143 | 2.7391114 |
| 51 | H | -1.4226952 | -1.0332247 | 1.9375486 |
| 52 | H | -1.3603697 | -2.3800270 | 3.1088057 |
| 53 | C | 0.8133735 | 1.4889134 | 2.7391130 |
| 54 | H | 1.3603707 | 2.3800256 | 3.1088081 |
| 55 | H | 1.4226955 | 1.0332245 | 1.9375496 |
| 56 | C | -0.5976402 | -0.4967398 | 3.9195082 |
| 57 | H | -0.4856625 | -1.0850280 | 4.8546134 |
| 58 | H | -1.5470994 | 0.0659512 | 4.0398199 |
| 59 | C | 0.5976407 | 0.4967378 | 3.9195087 |
| 60 | H | 1.5470997 | -0.0659536 | 4.0398195 |

| | | | | |
|---|---|---|---|---|
| 61 | H | 0.4856633 | 1.0850250 | 4.8546146 |

[Cr($L^1$)]$^0$, high-spin

| | | | | |
|---|---|---|---|---|
| 1 | Cr | 0.000084 | 0.000054 | -0.633025 |
| 2 | N | -2.038661 | -0.179885 | -0.807734 |
| 3 | C | -2.927308 | 0.740144 | -0.218084 |
| 4 | C | -4.269708 | 0.802529 | -0.624287 |
| 5 | C | -4.763302 | -0.097523 | -1.586407 |
| 6 | H | -5.814990 | -0.065989 | -1.898262 |
| 7 | C | -3.880224 | -1.063935 | -2.145958 |
| 8 | H | -4.236119 | -1.789680 | -2.888578 |
| 9 | C | -2.549210 | -1.051324 | -1.736062 |
| 10 | C | -2.325034 | 1.485735 | 0.866172 |
| 11 | N | -1.013779 | 1.202365 | 1.125984 |
| 12 | N | -0.689548 | 1.887190 | 2.253539 |
| 13 | C | -1.757129 | 2.619781 | 2.715003 |
| 14 | H | -1.676323 | 3.235772 | 3.617557 |
| 15 | C | -2.822374 | 2.405707 | 1.845805 |
| 16 | H | -3.823864 | 2.840040 | 1.918602 |
| 17 | N | -1.560015 | -1.906686 | -2.265138 |
| 18 | N | -0.272431 | -1.708014 | -1.826710 |
| 19 | C | 0.499540 | -2.566339 | -2.534018 |
| 20 | H | 1.578771 | -2.598316 | -2.348764 |
| 21 | C | -0.293268 | -3.329119 | -3.422629 |
| 22 | H | 0.039171 | -4.112078 | -4.110835 |
| 23 | C | -1.603261 | -2.888265 | -3.231470 |
| 24 | H | -2.544009 | -3.190002 | -3.699215 |
| 25 | N | 2.038806 | 0.180059 | -0.807662 |
| 26 | C | 2.549279 | 1.051665 | -1.735787 |
| 27 | C | 3.880453 | 1.063546 | -2.146709 |
| 28 | H | 4.236267 | 1.789074 | -2.889711 |
| 29 | C | 4.763656 | 0.096409 | -1.588333 |
| 30 | H | 5.815377 | 0.063960 | -1.901579 |
| 31 | C | 4.270017 | -0.803316 | -0.625777 |
| 32 | C | 2.927492 | -0.740126 | -0.218314 |
| 33 | N | 1.559998 | 1.907333 | -2.264405 |
| 34 | N | 0.272596 | 1.708040 | -1.826781 |
| 35 | C | -0.499206 | 2.565919 | -2.534654 |
| 36 | H | -1.578341 | 2.596948 | -2.350329 |
| 37 | C | 0.293394 | 3.329436 | -3.422374 |
| 38 | H | -0.038968 | 4.112317 | -4.110694 |
| 39 | C | 1.603183 | 2.889574 | -3.230082 |
| 40 | H | 2.543896 | 3.192189 | -3.696786 |
| 41 | C | 2.325303 | -1.485540 | 0.866251 |
| 42 | N | 1.014017 | -1.202195 | 1.126026 |
| 43 | N | 0.689766 | -1.886902 | 2.253687 |
| 44 | C | 1.757852 | -2.618758 | 2.715932 |
| 45 | H | 1.677228 | -3.234174 | 3.618953 |
| 46 | C | 2.822895 | -2.405089 | 1.846334 |
| 47 | H | 3.824481 | -2.839172 | 1.919280 |
| 48 | H | -4.936193 | 1.543367 | -0.159911 |
| 49 | H | 4.936573 | -1.544770 | -0.162169 |
| 50 | C | -0.583892 | -1.583356 | 2.892910 |
| 51 | H | -1.220929 | -1.194150 | 2.077164 |
| 52 | H | -1.035761 | -2.529924 | 3.253696 |
| 53 | C | 0.583500 | 1.582957 | 2.893425 |
| 54 | H | 1.035753 | 2.529332 | 3.254297 |
| 55 | H | 1.220697 | 1.193185 | 2.078147 |
| 56 | C | -0.523145 | -0.570648 | 4.071440 |
| 57 | H | -0.350883 | -1.134751 | 5.012588 |
| 58 | H | -1.542687 | -0.141600 | 4.169837 |

| | | | | |
|---|---|---|---|---|
| 59 | C | 0.521507 | 0.570557 | 4.072028 |
| 60 | H | 1.540887 | 0.141528 | 4.171649 |
| 61 | H | 0.348123 | 1.134912 | 5.012924 |

$[Cr_2(\mu\text{-}L^1)_2]^0$ conformation (a), low-spin

| | | | | |
|---|---|---|---|---|
| 1 | Cr | 0.000001 | 0.000001 | 4.230952 |
| 2 | Cr | 0.000001 | -0.000001 | -4.230952 |
| 3 | C | -2.635304 | -0.702687 | -1.857098 |
| 4 | C | -2.470816 | 0.308393 | -0.703891 |
| 5 | C | -2.470816 | -0.308393 | 0.703890 |
| 6 | C | -2.635304 | 0.702686 | 1.857098 |
| 7 | H | -2.712205 | -0.162112 | -2.818233 |
| 8 | H | -3.568648 | -1.286304 | -1.717436 |
| 9 | H | -3.319080 | 1.021879 | -0.798142 |
| 10 | H | -1.550182 | 0.902560 | -0.872557 |
| 11 | H | -1.550181 | -0.902560 | 0.872556 |
| 12 | H | -3.319078 | -1.021880 | 0.798142 |
| 13 | H | -3.568648 | 1.286303 | 1.717435 |
| 14 | H | -2.712205 | 0.162111 | 2.818233 |
| 15 | N | -1.551058 | -1.663878 | -2.021574 |
| 16 | N | -0.513686 | -1.427862 | -2.882227 |
| 17 | C | 0.275783 | -2.560463 | -2.834788 |
| 18 | C | -0.284321 | -3.507956 | -1.927597 |
| 19 | C | -1.433851 | -2.906671 | -1.438415 |
| 20 | H | -2.179076 | -3.257838 | -0.718482 |
| 21 | H | 0.098719 | -4.500278 | -1.675239 |
| 22 | C | 1.419210 | -2.523836 | -3.705991 |
| 23 | C | 2.405795 | -3.508533 | -3.910073 |
| 24 | C | 3.434852 | -3.284934 | -4.842887 |
| 25 | C | 3.453124 | -2.076089 | -5.581102 |
| 26 | C | 2.443061 | -1.138880 | -5.339519 |
| 27 | N | 1.445045 | -1.314079 | -4.406769 |
| 28 | H | 4.229843 | -1.878574 | -6.330939 |
| 29 | H | 4.210859 | -4.043519 | -5.011494 |
| 30 | H | 2.355679 | -4.447555 | -3.341262 |
| 31 | N | 2.296339 | 0.093599 | -5.984944 |
| 32 | N | 1.188852 | 0.833989 | -5.618155 |
| 33 | C | 1.252011 | 1.974791 | -6.348615 |
| 34 | C | 2.398989 | 1.969174 | -7.178778 |
| 35 | C | 3.050036 | 0.761898 | -6.929802 |
| 36 | H | 3.966717 | 0.330125 | -7.338573 |
| 37 | H | 2.715966 | 2.749564 | -7.877268 |
| 38 | H | 0.471613 | 2.733371 | -6.233789 |
| 39 | N | -1.445046 | 1.314081 | -4.406772 |
| 40 | C | -2.443060 | 1.138881 | -5.339522 |
| 41 | C | -3.453126 | 2.076097 | -5.581106 |
| 42 | C | -3.434858 | 3.284945 | -4.842892 |
| 43 | C | -2.405798 | 3.508538 | -3.910078 |
| 44 | C | -1.419209 | 2.523836 | -3.705994 |
| 45 | H | -2.355678 | 4.447561 | -3.341265 |
| 46 | H | -4.210864 | 4.043532 | -5.011500 |
| 47 | H | -4.229846 | 1.878579 | -6.330945 |
| 48 | N | -2.296339 | -0.093601 | -5.984947 |
| 49 | N | -1.188849 | -0.833995 | -5.618156 |
| 50 | C | -1.252010 | -1.974797 | -6.348615 |
| 51 | C | -2.398993 | -1.969177 | -7.178780 |
| 52 | C | -3.050039 | -0.761898 | -6.929806 |
| 53 | H | -0.471614 | -2.733379 | -6.233791 |
| 54 | H | -2.715970 | -2.749567 | -7.877270 |
| 55 | H | -3.966721 | -0.330122 | -7.338577 |
| 56 | C | -0.275779 | 2.560462 | -2.834788 |

| | | | | |
|---|---|---|---|---|
| 57 | C | 0.284324 | 3.507958 | -1.927595 |
| 58 | N | 0.513688 | 1.427861 | -2.882227 |
| 59 | C | 1.433854 | 2.906671 | -1.438413 |
| 60 | N | 1.551061 | 1.663878 | -2.021572 |
| 61 | H | -0.098718 | 4.500279 | -1.675238 |
| 62 | H | 2.179078 | 3.257838 | -0.718481 |
| 63 | C | 2.635306 | 0.702687 | -1.857097 |
| 64 | C | 2.470818 | -0.308393 | -0.703890 |
| 65 | C | 2.470819 | 0.308392 | 0.703891 |
| 66 | C | 2.635306 | -0.702687 | 1.857098 |
| 67 | H | 2.712206 | -0.162113 | 2.818232 |
| 68 | H | 3.568649 | -1.286304 | 1.717435 |
| 69 | H | 1.550185 | 0.902559 | 0.872557 |
| 70 | H | 3.319081 | 1.021877 | 0.798143 |
| 71 | H | 1.550184 | -0.902559 | -0.872557 |
| 72 | H | 3.319080 | -1.021878 | -0.798143 |
| 73 | H | 2.712207 | 0.162113 | -2.818231 |
| 74 | H | 3.568650 | 1.286303 | -1.717435 |
| 75 | N | -1.551059 | 1.663878 | 2.021573 |
| 76 | N | -0.513686 | 1.427862 | 2.882227 |
| 77 | C | 0.275782 | 2.560463 | 2.834787 |
| 78 | C | -0.284322 | 3.507956 | 1.927597 |
| 79 | C | -1.433851 | 2.906670 | 1.438414 |
| 80 | H | -2.179076 | 3.257838 | 0.718481 |
| 81 | H | 0.098718 | 4.500278 | 1.675239 |
| 82 | N | 1.551060 | -1.663878 | 2.021573 |
| 83 | N | 0.513688 | -1.427861 | 2.882227 |
| 84 | C | -0.275780 | -2.560462 | 2.834788 |
| 85 | C | 1.433853 | -2.906671 | 1.438414 |
| 86 | C | 0.284323 | -3.507957 | 1.927596 |
| 87 | H | 2.179077 | -3.257838 | 0.718481 |
| 88 | H | -0.098719 | -4.500279 | 1.675238 |
| 89 | N | -1.445046 | -1.314081 | 4.406772 |
| 90 | C | -1.419209 | -2.523836 | 3.705994 |
| 91 | C | -2.405798 | -3.508538 | 3.910078 |
| 92 | C | -2.443060 | -1.138881 | 5.339522 |
| 93 | C | 1.419210 | 2.523836 | 3.705991 |
| 94 | N | 1.445045 | 1.314079 | 4.406769 |
| 95 | C | 2.443060 | 1.138881 | 5.339520 |
| 96 | C | 3.453124 | 2.076090 | 5.581102 |
| 97 | C | 2.405795 | 3.508534 | 3.910073 |
| 98 | C | 3.434852 | 3.284935 | 4.842887 |
| 99 | C | -3.453126 | -2.076096 | 5.581106 |
| 100 | C | -3.434858 | -3.284944 | 4.842892 |
| 101 | H | 2.355679 | 4.447556 | 3.341262 |
| 102 | H | 4.210859 | 4.043520 | 5.011494 |
| 103 | H | 4.229843 | 1.878575 | 6.330939 |
| 104 | H | -4.229846 | -1.878578 | 6.330944 |
| 105 | H | -4.210864 | -4.043531 | 5.011500 |
| 106 | H | -2.355678 | -4.447561 | 3.341265 |
| 107 | N | -2.296339 | 0.093601 | 5.984947 |
| 108 | N | -1.188849 | 0.833994 | 5.618156 |
| 109 | C | -1.252009 | 1.974796 | 6.348616 |
| 110 | C | -2.398992 | 1.969177 | 7.178780 |
| 111 | C | -3.050039 | 0.761898 | 6.929806 |
| 112 | H | -2.715970 | 2.749567 | 7.877270 |
| 113 | H | -0.471613 | 2.733378 | 6.233791 |
| 114 | H | -3.966721 | 0.330123 | 7.338577 |
| 115 | N | 2.296339 | -0.093599 | 5.984944 |
| 116 | N | 1.188852 | -0.833989 | 5.618155 |
| 117 | C | 1.252011 | -1.974791 | 6.348615 |
| 118 | C | 2.398990 | -1.969174 | 7.178777 |

| | | | | |
|---|---|---|---|---|
| 119 | C | 3.050036 | -0.761897 | 6.929802 |
| 120 | H | 3.966718 | -0.330124 | 7.338574 |
| 121 | H | 2.715967 | -2.749564 | 7.877268 |
| 122 | H | 0.471613 | -2.733372 | 6.233789 |

$[Cr_2(\mu\text{-}L^1)_2]^0$ conformation (a), mixed-spin

| | | | | |
|---|---|---|---|---|
| 1 | Cr | -0.048482 | 0.036971 | 4.259359 |
| 2 | Cr | -0.016748 | -0.243799 | -4.447412 |
| 3 | C | -2.560941 | -1.400444 | -1.990204 |
| 4 | C | -2.453078 | -0.302152 | -0.916337 |
| 5 | C | -2.263130 | -0.833866 | 0.513706 |
| 6 | C | -2.540033 | 0.181061 | 1.639279 |
| 7 | H | -2.815775 | -0.945691 | -2.965931 |
| 8 | H | -3.374453 | -2.109081 | -1.732237 |
| 9 | H | -3.396335 | 0.283372 | -0.977049 |
| 10 | H | -1.636860 | 0.392316 | -1.198947 |
| 11 | H | -1.244453 | -1.251154 | 0.646012 |
| 12 | H | -2.964446 | -1.680006 | 0.686814 |
| 13 | H | -3.547582 | 0.629253 | 1.505946 |
| 14 | H | -2.527786 | -0.335390 | 2.615395 |
| 15 | N | -1.359141 | -2.201884 | -2.196291 |
| 16 | N | -0.358934 | -1.777163 | -3.022866 |
| 17 | C | 0.587681 | -2.778797 | -3.025652 |
| 18 | C | 0.169159 | -3.835723 | -2.162716 |
| 19 | C | -1.061998 | -3.431896 | -1.664029 |
| 20 | H | -1.755886 | -3.923257 | -0.974654 |
| 21 | H | 0.685555 | -4.776696 | -1.954502 |
| 22 | C | 1.710683 | -2.573635 | -3.902810 |
| 23 | C | 2.834698 | -3.395667 | -4.062396 |
| 24 | C | 3.850924 | -3.047181 | -4.973540 |
| 25 | C | 3.715267 | -1.846027 | -5.724416 |
| 26 | C | 2.582119 | -1.064459 | -5.533962 |
| 27 | N | 1.566819 | -1.384193 | -4.648676 |
| 28 | H | 4.490912 | -1.540366 | -6.439223 |
| 29 | H | 4.734806 | -3.684513 | -5.104019 |
| 30 | H | 2.910175 | -4.316083 | -3.465633 |
| 31 | N | 2.318731 | 0.144647 | -6.205781 |
| 32 | N | 1.179992 | 0.813331 | -5.832900 |
| 33 | C | 1.125348 | 1.923376 | -6.602597 |
| 34 | C | 2.237054 | 1.974935 | -7.474078 |
| 35 | C | 2.981611 | 0.824916 | -7.201729 |
| 36 | H | 3.906503 | 0.441000 | -7.639790 |
| 37 | H | 2.474416 | 2.748005 | -8.210999 |
| 38 | H | 0.291571 | 2.621407 | -6.476837 |
| 39 | N | -1.488010 | 1.333878 | -4.227178 |
| 40 | C | -2.673086 | 1.252496 | -4.941396 |
| 41 | C | -3.693059 | 2.202158 | -4.866267 |
| 42 | C | -3.520107 | 3.326445 | -4.024903 |
| 43 | C | -2.306058 | 3.439775 | -3.295873 |
| 44 | C | -1.322759 | 2.454212 | -3.409243 |
| 45 | H | -2.129400 | 4.301393 | -2.637075 |
| 46 | H | -4.305321 | 4.088770 | -3.941577 |
| 47 | H | -4.611288 | 2.086392 | -5.457367 |
| 48 | N | -2.758344 | 0.120592 | -5.781127 |
| 49 | N | -1.713719 | -0.766473 | -5.775422 |
| 50 | C | -2.046726 | -1.742272 | -6.652154 |
| 51 | C | -3.309791 | -1.489430 | -7.229167 |
| 52 | C | -3.744545 | -0.290716 | -6.655278 |
| 53 | H | -1.353244 | -2.573273 | -6.820853 |
| 54 | H | -3.843771 | -2.091177 | -7.970828 |
| 55 | H | -4.659708 | 0.286977 | -6.804194 |

| | | | | |
|---|---|---|---|---|
| 56 | C | -0.054009 | 2.520377 | -2.686576 |
| 57 | C | 0.576790 | 3.601977 | -1.996143 |
| 58 | N | 0.732653 | 1.413152 | -2.643112 |
| 59 | C | 1.785576 | 3.082634 | -1.548811 |
| 60 | N | 1.843385 | 1.773667 | -1.957328 |
| 61 | H | 0.215679 | 4.626927 | -1.872360 |
| 62 | H | 2.606278 | 3.541064 | -0.987208 |
| 63 | C | 2.913772 | 0.806758 | -1.726853 |
| 64 | C | 2.692658 | -0.157669 | -0.545172 |
| 65 | C | 2.576538 | 0.530340 | 0.825876 |
| 66 | C | 2.825709 | -0.377776 | 2.046088 |
| 67 | H | 2.785094 | 0.225788 | 2.970868 |
| 68 | H | 3.839559 | -0.825413 | 1.980831 |
| 69 | H | 1.589699 | 1.022566 | 0.931638 |
| 70 | H | 3.332184 | 1.343633 | 0.896661 |
| 71 | H | 1.800389 | -0.784007 | -0.749666 |
| 72 | H | 3.563075 | -0.850910 | -0.550741 |
| 73 | H | 3.030604 | 0.228256 | -2.663129 |
| 74 | H | 3.843123 | 1.391295 | -1.570535 |
| 75 | N | -1.583543 | 1.275621 | 1.766856 |
| 76 | N | -0.628438 | 1.269490 | 2.748624 |
| 77 | C | 0.015856 | 2.486230 | 2.649224 |
| 78 | C | -0.547130 | 3.252843 | 1.587381 |
| 79 | C | -1.552305 | 2.458116 | 1.058509 |
| 80 | H | -2.244291 | 2.633083 | 0.229213 |
| 81 | H | -0.255448 | 4.252265 | 1.255197 |
| 82 | N | 1.887618 | -1.480206 | 2.233029 |
| 83 | N | 0.791451 | -1.368784 | 3.046348 |
| 84 | C | 0.177087 | -2.606025 | 3.011833 |
| 85 | C | 1.972744 | -2.744766 | 1.691757 |
| 86 | C | 0.903379 | -3.491195 | 2.161482 |
| 87 | H | 2.794257 | -3.007867 | 1.018789 |
| 88 | H | 0.678062 | -4.536473 | 1.933478 |
| 89 | N | -1.274482 | -1.478088 | 4.430759 |
| 90 | C | -1.011238 | -2.705946 | 3.814447 |
| 91 | C | -1.850792 | -3.819912 | 4.009947 |
| 92 | C | -2.362961 | -1.410756 | 5.274081 |
| 93 | C | 1.058311 | 2.688551 | 3.618552 |
| 94 | N | 1.169505 | 1.561597 | 4.439251 |
| 95 | C | 2.085695 | 1.612555 | 5.466763 |
| 96 | C | 2.937735 | 2.698242 | 5.694424 |
| 97 | C | 1.888487 | 3.811673 | 3.798356 |
| 98 | C | 2.839306 | 3.819081 | 4.834495 |
| 99 | C | -3.233618 | -2.480654 | 5.503945 |
| 100 | C | -2.971970 | -3.710246 | 4.851784 |
| 101 | H | 1.776118 | 4.676896 | 3.130114 |
| 102 | H | 3.492115 | 4.688749 | 4.988948 |
| 103 | H | 3.655334 | 2.679416 | 6.524935 |
| 104 | H | -4.093099 | -2.367143 | 6.177354 |
| 105 | H | -3.635509 | -4.570121 | 5.015171 |
| 106 | H | -1.615259 | -4.766989 | 3.504618 |
| 107 | N | -2.470781 | -0.132157 | 5.829700 |
| 108 | N | -1.478528 | 0.760319 | 5.473176 |
| 109 | C | -1.786329 | 1.921166 | 6.103023 |
| 110 | C | -2.975799 | 1.776435 | 6.856636 |
| 111 | C | -3.395655 | 0.460558 | 6.666988 |
| 112 | H | -3.471489 | 2.537456 | 7.466962 |
| 113 | H | -1.137182 | 2.793100 | 5.978632 |
| 114 | H | -4.256742 | -0.093468 | 7.048318 |
| 115 | N | 2.045919 | 0.432431 | 6.216149 |
| 116 | N | 1.107219 | -0.498854 | 5.818405 |
| 117 | C | 1.251714 | -1.548616 | 6.664722 |

| | | | | |
|---|---|---|---|---|
| 118 | C | 2.281505 | -1.292534 | 7.601912 |
| 119 | C | 2.774410 | -0.024689 | 7.296836 |
| 120 | H | 3.565238 | 0.579980 | 7.746994 |
| 121 | H | 2.626597 | -1.950688 | 8.405149 |
| 122 | H | 0.609149 | -2.427551 | 6.554012 |

$[Cr_2(\mu\text{-}L^1)_2]^0$ conformation (a), high-spin

| | | | | |
|---|---|---|---|---|
| 1 | Cr | -0.052737 | 0.012352 | 4.234885 |
| 2 | Cr | 0.059538 | -0.145752 | -4.274911 |
| 3 | C | -2.606388 | -1.464310 | -1.994754 |
| 4 | C | -2.525385 | -0.380263 | -0.903013 |
| 5 | C | -2.353913 | -0.937017 | 0.519408 |
| 6 | C | -2.762201 | 0.021481 | 1.654639 |
| 7 | H | -2.836287 | -0.998267 | -2.972523 |
| 8 | H | -3.427413 | -2.173521 | -1.763762 |
| 9 | H | -3.473907 | 0.194532 | -0.969525 |
| 10 | H | -1.715496 | 0.331169 | -1.157571 |
| 11 | H | -1.311947 | -1.275166 | 0.689297 |
| 12 | H | -2.990705 | -1.840727 | 0.643523 |
| 13 | H | -3.803518 | 0.371583 | 1.497718 |
| 14 | H | -2.727697 | -0.520211 | 2.618313 |
| 15 | N | -1.400038 | -2.263135 | -2.180765 |
| 16 | N | -0.394115 | -1.826593 | -2.993432 |
| 17 | C | 0.557776 | -2.823607 | -3.000118 |
| 18 | C | 0.129997 | -3.892381 | -2.152742 |
| 19 | C | -1.106812 | -3.497073 | -1.660440 |
| 20 | H | -1.805556 | -4.000650 | -0.985544 |
| 21 | H | 0.645724 | -4.834333 | -1.947004 |
| 22 | C | 1.709051 | -2.633570 | -3.850461 |
| 23 | C | 2.798793 | -3.508921 | -4.020699 |
| 24 | C | 3.833864 | -3.184864 | -4.912044 |
| 25 | C | 3.772093 | -1.957764 | -5.652873 |
| 26 | C | 2.670410 | -1.135156 | -5.435319 |
| 27 | N | 1.665803 | -1.424715 | -4.568805 |
| 28 | H | 4.562024 | -1.687457 | -6.364099 |
| 29 | H | 4.684283 | -3.865260 | -5.051423 |
| 30 | H | 2.827479 | -4.450975 | -3.454534 |
| 31 | N | 2.473819 | 0.109035 | -6.096772 |
| 32 | N | 1.345597 | 0.819566 | -5.786413 |
| 33 | C | 1.398918 | 1.941824 | -6.527502 |
| 34 | C | 2.569188 | 1.961770 | -7.326529 |
| 35 | C | 3.235954 | 0.774924 | -7.027363 |
| 36 | H | 4.175179 | 0.360840 | -7.402340 |
| 37 | H | 2.890359 | 2.736048 | -8.029457 |
| 38 | H | 0.591468 | 2.676903 | -6.441213 |
| 39 | N | -1.407222 | 1.440909 | -4.209220 |
| 40 | C | -2.605785 | 1.368024 | -4.880037 |
| 41 | C | -3.662985 | 2.257783 | -4.706398 |
| 42 | C | -3.510015 | 3.330660 | -3.782393 |
| 43 | C | -2.296764 | 3.425630 | -3.071530 |
| 44 | C | -1.273510 | 2.489755 | -3.278111 |
| 45 | H | -2.144819 | 4.225676 | -2.333078 |
| 46 | H | -4.325935 | 4.045702 | -3.615770 |
| 47 | H | -4.596723 | 2.136428 | -5.269635 |
| 48 | N | -2.699510 | 0.299945 | -5.819248 |
| 49 | N | -1.745536 | -0.671876 | -5.813674 |
| 50 | C | -2.107785 | -1.546513 | -6.770977 |
| 51 | C | -3.304475 | -1.138403 | -7.410357 |
| 52 | C | -3.656869 | 0.054937 | -6.781161 |
| 53 | H | -1.483641 | -2.427396 | -6.962071 |
| 54 | H | -3.838659 | -1.632582 | -8.228008 |

| | | | | |
|---|---|---|---|---|
| 55 | H | -4.486584 | 0.743934 | -6.956067 |
| 56 | C | -0.011235 | 2.532464 | -2.554111 |
| 57 | C | 0.601524 | 3.574597 | -1.786151 |
| 58 | N | 0.799356 | 1.438043 | -2.578849 |
| 59 | C | 1.817271 | 3.046869 | -1.369341 |
| 60 | N | 1.904094 | 1.770171 | -1.863505 |
| 61 | H | 0.224106 | 4.582868 | -1.595251 |
| 62 | H | 2.625548 | 3.480286 | -0.771670 |
| 63 | C | 2.977427 | 0.800283 | -1.673996 |
| 64 | C | 2.754072 | -0.217811 | -0.537988 |
| 65 | C | 2.634051 | 0.413499 | 0.858669 |
| 66 | C | 2.872147 | -0.544512 | 2.040175 |
| 67 | H | 2.860938 | 0.032903 | 2.983544 |
| 68 | H | 3.868631 | -1.024187 | 1.946527 |
| 69 | H | 1.648692 | 0.904253 | 0.982727 |
| 70 | H | 3.393035 | 1.219149 | 0.967747 |
| 71 | H | 1.861681 | -0.834149 | -0.771475 |
| 72 | H | 3.624368 | -0.910708 | -0.569230 |
| 73 | H | 3.103618 | 0.265837 | -2.635330 |
| 74 | H | 3.903343 | 1.380914 | -1.485336 |
| 75 | N | -1.936054 | 1.210493 | 1.820898 |
| 76 | N | -0.792825 | 1.174448 | 2.573963 |
| 77 | C | -0.258797 | 2.445960 | 2.510300 |
| 78 | C | -1.066210 | 3.265081 | 1.664463 |
| 79 | C | -2.114502 | 2.449539 | 1.258894 |
| 80 | H | -2.973824 | 2.652426 | 0.611458 |
| 81 | H | -0.924974 | 4.322373 | 1.424555 |
| 82 | N | 1.902011 | -1.621965 | 2.209093 |
| 83 | N | 0.771187 | -1.447807 | 2.959048 |
| 84 | C | 0.125393 | -2.666515 | 2.964274 |
| 85 | C | 1.974609 | -2.906659 | 1.728303 |
| 86 | C | 0.863485 | -3.605071 | 2.181556 |
| 87 | H | 2.818192 | -3.224386 | 1.106648 |
| 88 | H | 0.629179 | -4.657150 | 1.996632 |
| 89 | N | -1.284190 | -1.505554 | 4.431130 |
| 90 | C | -1.067436 | -2.728302 | 3.767761 |
| 91 | C | -1.937080 | -3.812919 | 3.947074 |
| 92 | C | -2.348374 | -1.437690 | 5.314883 |
| 93 | C | 0.886901 | 2.690787 | 3.347014 |
| 94 | N | 1.119316 | 1.612782 | 4.225014 |
| 95 | C | 2.107435 | 1.754968 | 5.168474 |
| 96 | C | 2.941058 | 2.872332 | 5.264246 |
| 97 | C | 1.701390 | 3.835262 | 3.391319 |
| 98 | C | 2.737835 | 3.933901 | 4.341025 |
| 99 | C | -3.233113 | -2.487573 | 5.535701 |
| 100 | C | -3.033991 | -3.705029 | 4.827225 |
| 101 | H | 1.511678 | 4.656370 | 2.684606 |
| 102 | H | 3.376658 | 4.826175 | 4.379454 |
| 103 | H | 3.727175 | 2.925981 | 6.029286 |
| 104 | H | -4.067614 | -2.377555 | 6.241531 |
| 105 | H | -3.718727 | -4.549398 | 4.979523 |
| 106 | H | -1.744677 | -4.751192 | 3.405964 |
| 107 | N | -2.428409 | -0.163855 | 5.902644 |
| 108 | N | -1.478025 | 0.749633 | 5.497743 |
| 109 | C | -1.775993 | 1.910512 | 6.129755 |
| 110 | C | -2.920849 | 1.749266 | 6.939664 |
| 111 | C | -3.315946 | 0.417601 | 6.780402 |
| 112 | H | -3.401892 | 2.501596 | 7.572029 |
| 113 | H | -1.147252 | 2.790894 | 5.963359 |
| 114 | H | -4.140230 | -0.152017 | 7.216842 |
| 115 | N | 2.178918 | 0.620519 | 5.992557 |
| 116 | N | 1.278460 | -0.386659 | 5.707620 |

| | | | | |
|---|---|---|---|---|
| 117 | C | 1.529931 | -1.377611 | 6.597972 |
| 118 | C | 2.594778 | -1.012633 | 7.451974 |
| 119 | C | 2.990808 | 0.263865 | 7.046854 |
| 120 | H | 3.767575 | 0.935721 | 7.420782 |
| 121 | H | 3.025525 | -1.600652 | 8.267799 |
| 122 | H | 0.932436 | -2.294015 | 6.567431 |

$[Cr_2(\mu\text{-}L^1)_2]^0$ conformation (b), low-spin

| | | | | |
|---|---|---|---|---|
| 1 | Cr | -4.256409 | 0.031469 | 0.301274 |
| 2 | Cr | 4.242584 | -0.036752 | -0.314150 |
| 3 | N | 4.232063 | -0.762107 | -2.133955 |
| 4 | C | 5.076794 | -0.254822 | -3.097151 |
| 5 | C | 5.201268 | -0.791741 | -4.382829 |
| 6 | C | 4.435994 | -1.938228 | -4.706290 |
| 7 | C | 3.598468 | -2.505912 | -3.729130 |
| 8 | C | 3.514138 | -1.919541 | -2.451807 |
| 9 | N | 5.762683 | 0.860231 | -2.605185 |
| 10 | N | 5.518691 | 1.179179 | -1.282700 |
| 11 | C | 6.273359 | 2.276626 | -1.026370 |
| 12 | C | 6.995482 | 2.663463 | -2.181033 |
| 13 | C | 6.654545 | 1.747594 | -3.174915 |
| 14 | C | 2.758781 | -2.358711 | -1.309578 |
| 15 | N | 2.906669 | -1.570621 | -0.185454 |
| 16 | N | 2.121011 | -2.149596 | 0.776220 |
| 17 | C | 1.508485 | -3.284084 | 0.293402 |
| 18 | C | 1.882864 | -3.448357 | -1.031430 |
| 19 | C | 2.072157 | -1.626969 | 2.139009 |
| 20 | H | 2.700336 | -0.718449 | 2.123787 |
| 21 | H | 2.559257 | -2.356276 | 2.822165 |
| 22 | C | 0.654751 | -1.305702 | 2.650049 |
| 23 | H | 0.027248 | -2.222429 | 2.638328 |
| 24 | C | -0.050315 | -0.168571 | 1.896674 |
| 25 | H | -0.241124 | -0.462993 | 0.844708 |
| 26 | H | 0.619132 | 0.715618 | 1.863557 |
| 27 | C | -1.385368 | 0.240622 | 2.551850 |
| 28 | H | -2.150196 | -0.550937 | 2.441568 |
| 29 | H | -1.236937 | 0.419858 | 3.637827 |
| 30 | N | -2.960187 | 1.411103 | 1.046779 |
| 31 | N | -1.961552 | 1.451977 | 1.981984 |
| 32 | C | -1.526404 | 2.743433 | 2.182360 |
| 33 | C | -2.264264 | 3.582556 | 1.361290 |
| 34 | C | -3.157088 | 2.722058 | 0.656639 |
| 35 | N | -4.784601 | 1.673296 | -0.625652 |
| 36 | C | -4.202327 | 2.906163 | -0.313198 |
| 37 | C | -4.651246 | 4.101201 | -0.908187 |
| 38 | C | -5.722184 | 4.069233 | -1.819073 |
| 39 | C | -6.350871 | 2.833690 | -2.107846 |
| 40 | C | -5.863053 | 1.681096 | -1.483478 |
| 41 | N | -6.375921 | 0.389068 | -1.635353 |
| 42 | N | -5.761268 | -0.590699 | -0.881714 |
| 43 | C | -6.406527 | -1.744620 | -1.184173 |
| 44 | C | -7.433157 | -1.505403 | -2.129323 |
| 45 | C | -7.395228 | -0.138968 | -2.402898 |
| 46 | N | 4.605887 | 0.640343 | 1.491511 |
| 47 | C | 5.626209 | 0.105650 | 2.246675 |
| 48 | C | 5.987760 | 0.582103 | 3.510620 |
| 49 | C | 5.277879 | 1.687855 | 4.038516 |
| 50 | C | 4.250301 | 2.275209 | 3.278825 |
| 51 | C | 3.931227 | 1.749983 | 2.010904 |
| 52 | N | 6.227885 | -0.961341 | 1.573305 |
| 53 | N | 5.736360 | -1.227022 | 0.310559 |

| | | | | |
|---|---|---|---|---|
| 54 | C | 6.455779 | -2.281397 | -0.147959 |
| 55 | C | 7.404325 | -2.692623 | 0.819357 |
| 56 | C | 7.240658 | -1.837911 | 1.908252 |
| 57 | C | 2.945936 | 2.201728 | 1.067156 |
| 58 | N | 2.894664 | 1.467761 | -0.102916 |
| 59 | N | 1.942623 | 2.077349 | -0.878330 |
| 60 | C | 1.397277 | 3.161527 | -0.226120 |
| 61 | C | 2.006196 | 3.273309 | 1.014043 |
| 62 | C | 1.693362 | 1.648748 | -2.251677 |
| 63 | H | 2.622004 | 1.149828 | -2.584221 |
| 64 | H | 1.562327 | 2.565361 | -2.863961 |
| 65 | C | 0.501654 | 0.694401 | -2.457725 |
| 66 | H | 0.679351 | -0.222600 | -1.859573 |
| 67 | H | 0.542401 | 0.377335 | -3.523152 |
| 68 | C | -0.881824 | 1.292151 | -2.154970 |
| 69 | H | -0.995349 | 1.494198 | -1.071684 |
| 70 | H | -0.984637 | 2.275245 | -2.665323 |
| 71 | C | -2.079231 | 0.434746 | -2.617526 |
| 72 | H | -3.022845 | 0.976904 | -2.421259 |
| 73 | H | -2.015899 | 0.245436 | -3.709134 |
| 74 | N | -2.899704 | -1.023317 | -0.801616 |
| 75 | N | -2.197887 | -0.863325 | -1.966021 |
| 76 | C | -1.555619 | -2.030478 | -2.320424 |
| 77 | C | -1.846559 | -2.991727 | -1.366027 |
| 78 | C | -2.689443 | -2.335864 | -0.422089 |
| 79 | N | -4.114575 | -1.658377 | 1.281237 |
| 80 | C | -3.370930 | -2.731527 | 0.779949 |
| 81 | C | -3.362752 | -3.983621 | 1.425694 |
| 82 | C | -4.125676 | -4.173818 | 2.591857 |
| 83 | C | -4.915231 | -3.107066 | 3.086293 |
| 84 | C | -4.888077 | -1.889644 | 2.398388 |
| 85 | N | -5.621185 | -0.746330 | 2.729663 |
| 86 | N | -5.490517 | 0.320061 | 1.862240 |
| 87 | C | -6.274856 | 1.303490 | 2.369553 |
| 88 | C | -6.904896 | 0.869902 | 3.560940 |
| 89 | C | -6.473393 | -0.438872 | 3.772039 |
| 90 | H | -6.096176 | -2.680096 | -0.708579 |
| 91 | H | -8.120749 | -2.238544 | -2.561872 |
| 92 | H | -7.995709 | 0.490633 | -3.063836 |
| 93 | H | -2.187923 | 4.670893 | 1.288234 |
| 94 | H | -0.725858 | 2.955046 | 2.897440 |
| 95 | H | -0.940522 | -2.075835 | -3.224103 |
| 96 | H | -1.513318 | -4.032532 | -1.355644 |
| 97 | H | -6.338004 | 2.266689 | 1.853735 |
| 98 | H | -7.591506 | 1.440628 | 4.193573 |
| 99 | H | -6.694534 | -1.163343 | 4.559407 |
| 100 | H | 6.964275 | 1.653689 | -4.218563 |
| 101 | H | 7.683741 | 3.508200 | -2.281158 |
| 102 | H | 6.255986 | 2.726428 | -0.028994 |
| 103 | H | 1.568794 | -4.244192 | -1.712038 |
| 104 | H | 0.846462 | -3.877892 | 0.931005 |
| 105 | H | 6.250666 | -2.685031 | -1.144254 |
| 106 | H | 8.122773 | -3.513810 | 0.736599 |
| 107 | H | 7.749513 | -1.779723 | 2.873483 |
| 108 | H | 1.814463 | 4.033606 | 1.776051 |
| 109 | H | 0.615727 | 3.763687 | -0.698609 |
| 110 | H | 3.695668 | 3.145675 | 3.656285 |
| 111 | H | 5.540229 | 2.087718 | 5.027233 |
| 112 | H | 6.808846 | 0.115066 | 4.069636 |
| 113 | H | -4.162605 | 5.050101 | -0.646159 |
| 114 | H | -6.082237 | 4.992849 | -2.291837 |
| 115 | H | -7.205414 | 2.776660 | -2.794373 |

| | | | | |
|---|---|---|---|---|
| 116 | H | 3.015300 | -3.412101 | -3.945470 |
| 117 | H | 4.511849 | -2.386217 | -5.706171 |
| 118 | H | 5.884002 | -0.338481 | -5.113120 |
| 119 | H | -5.544380 | -3.230032 | 3.977294 |
| 120 | H | -4.127465 | -5.143654 | 3.107303 |
| 121 | H | -2.766633 | -4.804432 | 1.002635 |
| 122 | H | 0.763226 | -1.032464 | 3.722397 |

$[Cr_2(\mu\text{-}L^1)_2]^0$ conformation (b), mixed-spin

| | | | | |
|---|---|---|---|---|
| 1 | Cr | -4.177805 | 0.042940 | 0.286782 |
| 2 | Cr | 4.120520 | -0.002267 | -0.135965 |
| 3 | N | 4.137694 | -0.860435 | -2.151028 |
| 4 | C | 4.837692 | -0.381433 | -3.217825 |
| 5 | C | 4.825566 | -0.941565 | -4.492787 |
| 6 | C | 4.032071 | -2.114978 | -4.708684 |
| 7 | C | 3.318165 | -2.646388 | -3.629330 |
| 8 | C | 3.373231 | -2.031867 | -2.361762 |
| 9 | N | 5.623717 | 0.781692 | -2.941367 |
| 10 | N | 5.571841 | 1.330155 | -1.700875 |
| 11 | C | 6.396782 | 2.387858 | -1.733654 |
| 12 | C | 6.996074 | 2.531200 | -3.012613 |
| 13 | C | 6.478227 | 1.480884 | -3.765115 |
| 14 | C | 2.696198 | -2.533587 | -1.188682 |
| 15 | N | 2.701182 | -1.761537 | -0.057341 |
| 16 | N | 2.060354 | -2.491036 | 0.905889 |
| 17 | C | 1.642851 | -3.698554 | 0.410350 |
| 18 | C | 2.022449 | -3.769402 | -0.923463 |
| 19 | C | 1.939929 | -1.994593 | 2.272951 |
| 20 | H | 2.640745 | -1.140460 | 2.345477 |
| 21 | H | 2.307492 | -2.786266 | 2.959403 |
| 22 | C | 0.520900 | -1.572188 | 2.698670 |
| 23 | H | -0.177525 | -2.429299 | 2.584563 |
| 24 | C | -0.037216 | -0.339683 | 1.970609 |
| 25 | H | -0.201019 | -0.567046 | 0.898057 |
| 26 | H | 0.706298 | 0.483424 | 2.011277 |
| 27 | C | -1.362414 | 0.136705 | 2.597354 |
| 28 | H | -2.157249 | -0.621028 | 2.468936 |
| 29 | H | -1.226073 | 0.300683 | 3.687365 |
| 30 | N | -2.883803 | 1.382268 | 1.098847 |
| 31 | N | -1.879335 | 1.374447 | 2.028485 |
| 32 | C | -1.419592 | 2.650262 | 2.268081 |
| 33 | C | -2.147715 | 3.528628 | 1.479853 |
| 34 | C | -3.061216 | 2.708370 | 0.754453 |
| 35 | N | -4.693284 | 1.724582 | -0.571397 |
| 36 | C | -4.102127 | 2.939898 | -0.210167 |
| 37 | C | -4.539310 | 4.160015 | -0.761113 |
| 38 | C | -5.605178 | 4.170366 | -1.678835 |
| 39 | C | -6.237854 | 2.950952 | -2.022264 |
| 40 | C | -5.761555 | 1.771532 | -1.440433 |
| 41 | N | -6.275564 | 0.488784 | -1.654005 |
| 42 | N | -5.668999 | -0.525788 | -0.940433 |
| 43 | C | -6.311860 | -1.663646 | -1.302418 |
| 44 | C | -7.327413 | -1.380178 | -2.247282 |
| 45 | C | -7.285737 | -0.002489 | -2.457305 |
| 46 | N | 4.486269 | 0.760790 | 1.644078 |
| 47 | C | 5.478138 | 0.250079 | 2.462635 |
| 48 | C | 5.772005 | 0.752602 | 3.724427 |
| 49 | C | 5.024889 | 1.863204 | 4.208729 |
| 50 | C | 4.039375 | 2.431310 | 3.378080 |
| 51 | C | 3.787245 | 1.895318 | 2.107479 |
| 52 | N | 6.141873 | -0.834076 | 1.855406 |

| | | | | |
|---|---|---|---|---|
| 53 | N | 5.778219 | -1.132087 | 0.565934 |
| 54 | C | 6.527463 | -2.196562 | 0.201272 |
| 55 | C | 7.383158 | -2.584763 | 1.257364 |
| 56 | C | 7.113888 | -1.699161 | 2.303203 |
| 57 | C | 2.865344 | 2.385891 | 1.117143 |
| 58 | N | 2.840470 | 1.692870 | -0.073354 |
| 59 | N | 1.950020 | 2.348425 | -0.875792 |
| 60 | C | 1.412605 | 3.430989 | -0.224940 |
| 61 | C | 1.966656 | 3.492800 | 1.046213 |
| 62 | C | 1.716058 | 1.923900 | -2.253639 |
| 63 | H | 2.663442 | 1.475511 | -2.606137 |
| 64 | H | 1.537770 | 2.842464 | -2.850306 |
| 65 | C | 0.562748 | 0.922852 | -2.455695 |
| 66 | H | 0.778409 | 0.012796 | -1.859653 |
| 67 | H | 0.607997 | 0.609455 | -3.521850 |
| 68 | C | -0.835762 | 1.477290 | -2.136705 |
| 69 | H | -0.945482 | 1.664503 | -1.049876 |
| 70 | H | -0.965478 | 2.464573 | -2.632871 |
| 71 | C | -2.014685 | 0.600861 | -2.611631 |
| 72 | H | -2.969816 | 1.111409 | -2.389940 |
| 73 | H | -1.956748 | 0.449610 | -3.709639 |
| 74 | N | -2.801389 | -0.942801 | -0.850761 |
| 75 | N | -2.096760 | -0.720682 | -2.002949 |
| 76 | C | -1.416465 | -1.854900 | -2.392091 |
| 77 | C | -1.681629 | -2.855879 | -1.471832 |
| 78 | C | -2.553158 | -2.259908 | -0.514162 |
| 79 | N | -4.022457 | -1.687641 | 1.190423 |
| 80 | C | -3.238573 | -2.718829 | 0.663097 |
| 81 | C | -3.202178 | -3.992754 | 1.263698 |
| 82 | C | -3.978589 | -4.248154 | 2.408436 |
| 83 | C | -4.805315 | -3.223147 | 2.929622 |
| 84 | C | -4.801444 | -1.979977 | 2.288849 |
| 85 | N | -5.562112 | -0.866770 | 2.658414 |
| 86 | N | -5.431596 | 0.243389 | 1.847505 |
| 87 | C | -6.242716 | 1.187638 | 2.385951 |
| 88 | C | -6.889286 | 0.685738 | 3.541096 |
| 89 | C | -6.440259 | -0.624998 | 3.696528 |
| 90 | H | -6.007502 | -2.620088 | -0.866379 |
| 91 | H | -8.009980 | -2.092048 | -2.721314 |
| 92 | H | -7.877358 | 0.657616 | -3.096024 |
| 93 | H | -2.048623 | 4.616830 | 1.439532 |
| 94 | H | -0.611628 | 2.824118 | 2.984988 |
| 95 | H | -0.801877 | -1.853017 | -3.297200 |
| 96 | H | -1.304944 | -3.881291 | -1.489589 |
| 97 | H | -6.313026 | 2.173367 | 1.916182 |
| 98 | H | -7.598777 | 1.213125 | 4.185955 |
| 99 | H | -6.666028 | -1.391591 | 4.441521 |
| 100 | H | 6.657364 | 1.184026 | -4.801385 |
| 101 | H | 7.710891 | 3.289668 | -3.346834 |
| 102 | H | 6.524856 | 2.999808 | -0.833016 |
| 103 | H | 1.866653 | -4.610503 | -1.605060 |
| 104 | H | 1.115958 | -4.418233 | 1.046291 |
| 105 | H | 6.405987 | -2.623424 | -0.799503 |
| 106 | H | 8.106826 | -3.405295 | 1.264943 |
| 107 | H | 7.524011 | -1.624905 | 3.313560 |
| 108 | H | 1.765134 | 4.244344 | 1.814217 |
| 109 | H | 0.676430 | 4.072559 | -0.718723 |
| 110 | H | 3.468950 | 3.311102 | 3.709323 |
| 111 | H | 5.235760 | 2.281205 | 5.201349 |
| 112 | H | 6.574716 | 0.309621 | 4.328550 |
| 113 | H | -4.046945 | 5.095033 | -0.459148 |
| 114 | H | -5.957339 | 5.114196 | -2.116350 |

| | | | | |
|---|---|---|---|---|
| 115 | H | -7.086562 | 2.926516 | -2.718034 |
| 116 | H | 2.704229 | -3.549570 | -3.758102 |
| 117 | H | 3.993732 | -2.583661 | -5.700811 |
| 118 | H | 5.411323 | -0.505939 | -5.310432 |
| 119 | H | -5.440697 | -3.396434 | 3.807823 |
| 120 | H | -3.960442 | -5.236102 | 2.887981 |
| 121 | H | -2.573537 | -4.778540 | 0.821885 |
| 122 | H | 0.570163 | -1.365604 | 3.790784 |

$[Cr_2(\mu\text{-}L^1)_2]^0$ conformation (b), high-spin

| | | | | |
|---|---|---|---|---|
| 1 | Cr | -4.181525 | 0.014247 | 0.289791 |
| 2 | Cr | 4.171114 | 0.092565 | -0.252301 |
| 3 | N | 4.137505 | -0.962890 | -2.133910 |
| 4 | C | 4.748490 | -0.450921 | -3.256875 |
| 5 | C | 4.503335 | -0.887677 | -4.556963 |
| 6 | C | 3.577450 | -1.947945 | -4.759039 |
| 7 | C | 2.932723 | -2.491563 | -3.627850 |
| 8 | C | 3.207262 | -1.994308 | -2.346504 |
| 9 | N | 5.694099 | 0.579076 | -2.995220 |
| 10 | N | 5.678315 | 1.191999 | -1.778476 |
| 11 | C | 6.645530 | 2.126037 | -1.826969 |
| 12 | C | 7.300964 | 2.119524 | -3.083121 |
| 13 | C | 6.672430 | 1.109032 | -3.809641 |
| 14 | C | 2.558740 | -2.505878 | -1.145232 |
| 15 | N | 2.642819 | -1.778949 | 0.002589 |
| 16 | N | 1.989349 | -2.496886 | 0.952613 |
| 17 | C | 1.486787 | -3.665490 | 0.440291 |
| 18 | C | 1.826202 | -3.711057 | -0.906525 |
| 19 | C | 1.924272 | -2.020213 | 2.333144 |
| 20 | H | 2.665450 | -1.201718 | 2.403431 |
| 21 | H | 2.268271 | -2.841746 | 2.995789 |
| 22 | C | 0.536165 | -1.541105 | 2.794783 |
| 23 | H | -0.199679 | -2.366706 | 2.684984 |
| 24 | C | 0.014012 | -0.274879 | 2.097121 |
| 25 | H | -0.163846 | -0.469284 | 1.020285 |
| 26 | H | 0.778599 | 0.527827 | 2.154465 |
| 27 | C | -1.294558 | 0.215505 | 2.746578 |
| 28 | H | -2.087760 | -0.552949 | 2.655529 |
| 29 | H | -1.131840 | 0.402646 | 3.828940 |
| 30 | N | -2.693206 | 1.412514 | 1.117672 |
| 31 | N | -1.835213 | 1.443676 | 2.178676 |
| 32 | C | -1.533092 | 2.734470 | 2.532787 |
| 33 | C | -2.229246 | 3.587702 | 1.685595 |
| 34 | C | -2.964321 | 2.727152 | 0.815019 |
| 35 | N | -4.619802 | 1.752703 | -0.551160 |
| 36 | C | -3.963223 | 2.947714 | -0.203136 |
| 37 | C | -4.320266 | 4.168937 | -0.789819 |
| 38 | C | -5.371793 | 4.228284 | -1.729273 |
| 39 | C | -6.065788 | 3.034840 | -2.056972 |
| 40 | C | -5.667805 | 1.845826 | -1.449657 |
| 41 | N | -6.251027 | 0.590420 | -1.678426 |
| 42 | N | -5.656899 | -0.474734 | -1.035168 |
| 43 | C | -6.353851 | -1.576029 | -1.410262 |
| 44 | C | -7.391837 | -1.222078 | -2.299833 |
| 45 | C | -7.308572 | 0.164459 | -2.452040 |
| 46 | N | 4.452893 | 0.854578 | 1.671192 |
| 47 | C | 5.388129 | 0.375589 | 2.532209 |
| 48 | C | 5.601962 | 0.878896 | 3.812394 |
| 49 | C | 4.782775 | 1.977555 | 4.236491 |
| 50 | C | 3.827936 | 2.505100 | 3.354972 |
| 51 | C | 3.666347 | 1.952897 | 2.069298 |

| | | | | |
|---|---|---|---|---|
| 52 | N | 6.129982 | -0.709823 | 1.986121 |
| 53 | N | 5.836525 | -1.105554 | 0.710276 |
| 54 | C | 6.664284 | -2.132258 | 0.444618 |
| 55 | C | 7.503376 | -2.406363 | 1.554125 |
| 56 | C | 7.135817 | -1.477644 | 2.525534 |
| 57 | C | 2.757887 | 2.409334 | 1.044672 |
| 58 | N | 2.767431 | 1.743676 | -0.160727 |
| 59 | N | 1.866243 | 2.388791 | -0.960913 |
| 60 | C | 1.281694 | 3.434978 | -0.295094 |
| 61 | C | 1.817308 | 3.484905 | 0.985221 |
| 62 | C | 1.664610 | 1.969211 | -2.344214 |
| 63 | H | 2.632956 | 1.559934 | -2.689762 |
| 64 | H | 1.461111 | 2.884920 | -2.937266 |
| 65 | C | 0.546293 | 0.933320 | -2.571427 |
| 66 | H | 0.763588 | 0.029536 | -1.966275 |
| 67 | H | 0.626591 | 0.616548 | -3.634362 |
| 68 | C | -0.869369 | 1.459186 | -2.288139 |
| 69 | H | -1.016066 | 1.632353 | -1.204012 |
| 70 | H | -1.006009 | 2.447108 | -2.781239 |
| 71 | C | -2.014099 | 0.561494 | -2.795244 |
| 72 | H | -2.982605 | 1.060604 | -2.599189 |
| 73 | H | -1.925860 | 0.405869 | -3.889810 |
| 74 | N | -2.635234 | -0.945622 | -0.955358 |
| 75 | N | -2.083638 | -0.763016 | -2.190195 |
| 76 | C | -1.558765 | -1.934850 | -2.677630 |
| 77 | C | -1.783240 | -2.929832 | -1.735772 |
| 78 | C | -2.472687 | -2.281395 | -0.667934 |
| 79 | N | -3.902036 | -1.757588 | 1.131679 |
| 80 | C | -3.081781 | -2.744847 | 0.555361 |
| 81 | C | -2.955659 | -4.013896 | 1.136092 |
| 82 | C | -3.669531 | -4.334610 | 2.311302 |
| 83 | C | -4.527397 | -3.356970 | 2.878819 |
| 84 | C | -4.614987 | -2.110223 | 2.263759 |
| 85 | N | -5.414630 | -1.046810 | 2.707825 |
| 86 | N | -5.310042 | 0.125148 | 1.987520 |
| 87 | C | -6.140574 | 1.011542 | 2.591238 |
| 88 | C | -6.773011 | 0.414549 | 3.703883 |
| 89 | C | -6.297498 | -0.898293 | 3.755670 |
| 90 | H | -6.063232 | -2.556514 | -1.020160 |
| 91 | H | -8.120282 | -1.887494 | -2.772844 |
| 92 | H | -7.913966 | 0.869478 | -3.027472 |
| 93 | H | -2.249080 | 4.680591 | 1.723067 |
| 94 | H | -0.855962 | 2.941593 | 3.368044 |
| 95 | H | -1.071426 | -1.965726 | -3.657454 |
| 96 | H | -1.518189 | -3.986994 | -1.818186 |
| 97 | H | -6.230460 | 2.025491 | 2.189101 |
| 98 | H | -7.494089 | 0.873750 | 4.386900 |
| 99 | H | -6.522459 | -1.725833 | 4.433465 |
| 100 | H | 6.863646 | 0.715385 | -4.811235 |
| 101 | H | 8.129573 | 2.750664 | -3.418430 |
| 102 | H | 6.828277 | 2.759001 | -0.950828 |
| 103 | H | 1.607783 | -4.518010 | -1.611234 |
| 104 | H | 0.939198 | -4.374519 | 1.070683 |
| 105 | H | 6.615211 | -2.624747 | -0.532805 |
| 106 | H | 8.276245 | -3.176018 | 1.640922 |
| 107 | H | 7.507572 | -1.306186 | 3.539034 |
| 108 | H | 1.570814 | 4.208254 | 1.766989 |
| 109 | H | 0.524008 | 4.059220 | -0.779282 |
| 110 | H | 3.206867 | 3.360653 | 3.657410 |
| 111 | H | 4.917018 | 2.406018 | 5.238555 |
| 112 | H | 6.370810 | 0.460166 | 4.472806 |
| 113 | H | -3.775587 | 5.080354 | -0.503178 |

| | | | | |
|---|---|---|---|---|
| 114 | H | -5.657334 | 5.180012 | -2.195682 |
| 115 | H | -6.898645 | 3.044520 | -2.772843 |
| 116 | H | 2.199511 | -3.302874 | -3.740548 |
| 117 | H | 3.356198 | -2.311135 | -5.770980 |
| 118 | H | 5.012855 | -0.419115 | -5.408786 |
| 119 | H | -5.110697 | -3.575223 | 3.783596 |
| 120 | H | -3.576742 | -5.325138 | 2.774958 |
| 121 | H | -2.301614 | -4.756646 | 0.656793 |
| 122 | H | 0.618426 | -1.353155 | 3.888207 |

$[Cr_2(\mu\text{-}L^1)_2]^0$ conformation (c), low-spin

| | | | | |
|---|---|---|---|---|
| 1 | Cr | 0.149390 | 0.091734 | 4.165959 |
| 2 | Cr | -0.153820 | -0.082544 | -4.181420 |
| 3 | N | 2.038674 | -0.274527 | 4.532892 |
| 4 | C | 2.721791 | 0.444842 | 5.488181 |
| 5 | C | 4.046260 | 0.184447 | 5.854684 |
| 6 | H | 4.545766 | 0.797471 | 6.616020 |
| 7 | C | 4.714152 | -0.896725 | 5.230010 |
| 8 | H | 5.754642 | -1.127096 | 5.495615 |
| 9 | C | 4.026006 | -1.680585 | 4.286919 |
| 10 | H | 4.512692 | -2.541473 | 3.807590 |
| 11 | C | 2.692970 | -1.367720 | 3.957181 |
| 12 | N | 1.905310 | 1.446933 | 6.021803 |
| 13 | N | 0.608850 | 1.480562 | 5.545306 |
| 14 | C | 0.009244 | 2.512638 | 6.188409 |
| 15 | H | -1.038660 | 2.748049 | 5.978880 |
| 16 | C | 0.917830 | 3.140136 | 7.074640 |
| 17 | H | 0.724052 | 3.997161 | 7.726813 |
| 18 | C | 2.119737 | 2.444997 | 6.951983 |
| 19 | H | 3.089413 | 2.571508 | 7.439499 |
| 20 | C | 1.804900 | -2.050205 | 3.055572 |
| 21 | N | 0.549722 | -1.482660 | 2.938945 |
| 22 | N | -0.147855 | -2.307796 | 2.096143 |
| 23 | C | 0.634545 | -3.361158 | 1.677943 |
| 24 | H | 0.237032 | -4.112198 | 0.988594 |
| 25 | C | 1.883446 | -3.235630 | 2.265964 |
| 26 | H | 2.732281 | -3.917547 | 2.165362 |
| 27 | C | -1.485466 | -1.986618 | 1.609842 |
| 28 | H | -1.970286 | -1.361366 | 2.382165 |
| 29 | H | -2.049073 | -2.940989 | 1.546645 |
| 30 | C | -1.481243 | -1.279625 | 0.238873 |
| 31 | H | -1.025523 | -0.277770 | 0.365244 |
| 32 | H | -0.826980 | -1.845497 | -0.455936 |
| 33 | C | -2.881398 | -1.150359 | -0.378354 |
| 34 | H | -3.524647 | -0.511202 | 0.264718 |
| 35 | H | -3.375460 | -2.146386 | -0.400600 |
| 36 | C | -2.891647 | -0.624989 | -1.829010 |
| 37 | H | -2.289524 | -1.275234 | -2.489825 |
| 38 | H | -3.932417 | -0.627047 | -2.218250 |
| 39 | N | -1.232226 | 0.937727 | -2.780010 |
| 40 | N | -2.328278 | 0.710973 | -1.990591 |
| 41 | C | -2.758979 | 1.876545 | -1.398270 |
| 42 | H | -3.628072 | 1.878179 | -0.733290 |
| 43 | C | -1.915817 | 2.901708 | -1.798307 |
| 44 | H | -1.977592 | 3.955409 | -1.514969 |
| 45 | C | -0.961813 | 2.285872 | -2.659017 |
| 46 | N | 0.699840 | 1.683666 | -4.166416 |
| 47 | C | 0.164369 | 2.743512 | -3.428152 |
| 48 | C | 0.710711 | 4.039456 | -3.502393 |
| 49 | H | 0.268752 | 4.845670 | -2.900145 |
| 50 | C | 1.801467 | 4.290011 | -4.354838 |

| | | | | |
|---|---|---|---|---|
| 51 | H | 2.240108 | 5.294629 | -4.422007 |
| 52 | C | 2.315861 | 3.237444 | -5.150146 |
| 53 | H | 3.143624 | 3.406747 | -5.850787 |
| 54 | C | 1.729260 | 1.972835 | -5.035514 |
| 55 | N | 2.085155 | 0.835722 | -5.767224 |
| 56 | N | 1.313430 | -0.287653 | -5.543051 |
| 57 | C | 1.825500 | -1.250879 | -6.348271 |
| 58 | H | 1.376549 | -2.248929 | -6.351321 |
| 59 | C | 2.924134 | -0.748133 | -7.086318 |
| 60 | H | 3.537073 | -1.288010 | -7.814450 |
| 61 | C | 3.071985 | 0.583236 | -6.700166 |
| 62 | H | 3.779342 | 1.357843 | -7.005944 |
| 63 | N | -1.767708 | 0.492550 | 4.146562 |
| 64 | C | -2.624063 | -0.130088 | 5.028476 |
| 65 | C | -3.983735 | 0.179590 | 5.138659 |
| 66 | H | -4.623471 | -0.356551 | 5.851502 |
| 67 | C | -4.503068 | 1.214155 | 4.323150 |
| 68 | H | -5.566062 | 1.482819 | 4.387668 |
| 69 | C | -3.640340 | 1.908203 | 3.454974 |
| 70 | H | -4.010862 | 2.738384 | 2.837230 |
| 71 | C | -2.280529 | 1.547351 | 3.384339 |
| 72 | N | -1.938883 | -1.088511 | 5.781156 |
| 73 | N | -0.577821 | -1.165342 | 5.559224 |
| 74 | C | -0.125743 | -2.135563 | 6.392011 |
| 75 | H | 0.939401 | -2.386561 | 6.406224 |
| 76 | C | -1.193862 | -2.681572 | 7.143828 |
| 77 | H | -1.138724 | -3.476735 | 7.893515 |
| 78 | C | -2.339906 | -1.999730 | 6.737906 |
| 79 | H | -3.385959 | -2.083907 | 7.042039 |
| 80 | C | -1.230015 | 2.145003 | 2.603930 |
| 81 | N | 0.003820 | 1.540897 | 2.741767 |
| 82 | N | 0.858123 | 2.263422 | 1.952346 |
| 83 | C | 0.200699 | 3.307970 | 1.342108 |
| 84 | H | 0.733298 | 3.992175 | 0.674257 |
| 85 | C | -1.129943 | 3.265815 | 1.729229 |
| 86 | H | -1.925603 | 3.953545 | 1.431919 |
| 87 | C | 2.269585 | 1.913739 | 1.830146 |
| 88 | H | 2.415994 | 1.051145 | 2.506439 |
| 89 | H | 2.884293 | 2.753263 | 2.219535 |
| 90 | C | 2.703799 | 1.567467 | 0.392094 |
| 91 | H | 3.804821 | 1.414697 | 0.411355 |
| 92 | H | 2.531101 | 2.438012 | -0.277068 |
| 93 | C | 2.007142 | 0.321192 | -0.168965 |
| 94 | H | 0.910255 | 0.472028 | -0.158464 |
| 95 | H | 2.211209 | -0.538429 | 0.502450 |
| 96 | C | 2.442790 | -0.036376 | -1.602316 |
| 97 | H | 2.120391 | 0.735083 | -2.326416 |
| 98 | H | 3.549360 | -0.114509 | -1.660928 |
| 99 | N | 0.818243 | -1.356292 | -2.932456 |
| 100 | N | 1.880680 | -1.299721 | -2.068499 |
| 101 | C | 2.226596 | -2.557410 | -1.628648 |
| 102 | H | 3.052652 | -2.693759 | -0.924260 |
| 103 | C | 1.373130 | -3.475291 | -2.221648 |
| 104 | H | 1.390594 | -4.562730 | -2.107884 |
| 105 | C | 0.499086 | -2.697272 | -3.037334 |
| 106 | N | -1.016999 | -1.803533 | -4.547974 |
| 107 | C | -0.570635 | -2.987335 | -3.952489 |
| 108 | C | -1.144497 | -4.232552 | -4.275606 |
| 109 | H | -0.772050 | -5.141989 | -3.783751 |
| 110 | C | -2.178212 | -4.298846 | -5.226633 |
| 111 | H | -2.636385 | -5.262198 | -5.487961 |
| 112 | C | -2.613826 | -3.111640 | -5.863593 |

| | | | | |
|---|---|---|---|---|
| 113 | H | -3.402875 | -3.133026 | -6.626476 |
| 114 | C | -2.003022 | -1.905741 | -5.504146 |
| 115 | N | -2.291403 | -0.650097 | -6.046661 |
| 116 | N | -1.522661 | 0.396051 | -5.576108 |
| 117 | C | -1.970598 | 1.499942 | -6.224071 |
| 118 | H | -1.512357 | 2.472051 | -6.018881 |
| 119 | C | -3.026067 | 1.163177 | -7.105886 |
| 120 | H | -3.584128 | 1.839085 | -7.760909 |
| 121 | C | -3.213614 | -0.212042 | -6.976299 |
| 122 | H | -3.910531 | -0.902574 | -7.457111 |

$[Cr_2(\mu\text{-}L^1)_2]^0$ conformation (c), mixed-spin

| | | | | |
|---|---|---|---|---|
| 1 | Cr | 0.246832 | -0.035379 | 4.353996 |
| 2 | Cr | -0.213620 | -0.112276 | -4.179202 |
| 3 | N | 2.151248 | -0.369636 | 4.719630 |
| 4 | C | 2.842973 | 0.394702 | 5.643428 |
| 5 | C | 4.178579 | 0.185044 | 5.970116 |
| 6 | H | 4.679852 | 0.827230 | 6.706591 |
| 7 | C | 4.879305 | -0.874359 | 5.332540 |
| 8 | H | 5.934661 | -1.060874 | 5.569016 |
| 9 | C | 4.194409 | -1.674864 | 4.394895 |
| 10 | H | 4.708789 | -2.502907 | 3.886163 |
| 11 | C | 2.849614 | -1.422366 | 4.097667 |
| 12 | N | 2.026917 | 1.401322 | 6.191733 |
| 13 | N | 0.746459 | 1.487968 | 5.697795 |
| 14 | C | 0.155731 | 2.509879 | 6.361858 |
| 15 | H | -0.881764 | 2.775798 | 6.134594 |
| 16 | C | 1.058085 | 3.086244 | 7.283533 |
| 17 | H | 0.876265 | 3.926751 | 7.959874 |
| 18 | C | 2.243884 | 2.359112 | 7.157051 |
| 19 | H | 3.202973 | 2.439799 | 7.674741 |
| 20 | C | 2.004388 | -2.143533 | 3.177112 |
| 21 | N | 0.744213 | -1.625709 | 2.986548 |
| 22 | N | 0.097159 | -2.497189 | 2.161092 |
| 23 | C | 0.919373 | -3.534989 | 1.804120 |
| 24 | H | 0.565761 | -4.323255 | 1.131766 |
| 25 | C | 2.146146 | -3.351697 | 2.430427 |
| 26 | H | 3.014420 | -4.015158 | 2.388376 |
| 27 | C | -1.247594 | -2.221185 | 1.663559 |
| 28 | H | -1.750924 | -1.604451 | 2.432169 |
| 29 | H | -1.780849 | -3.192326 | 1.597788 |
| 30 | C | -1.266390 | -1.501931 | 0.303150 |
| 31 | H | -0.800945 | -0.505384 | 0.430235 |
| 32 | H | -0.639979 | -2.064380 | -0.419629 |
| 33 | C | -2.687878 | -1.342129 | -0.254228 |
| 34 | H | -3.305030 | -0.743995 | 0.450854 |
| 35 | H | -3.184953 | -2.334394 | -0.319837 |
| 36 | C | -2.744064 | -0.725945 | -1.664328 |
| 37 | H | -2.189443 | -1.347572 | -2.390670 |
| 38 | H | -3.799617 | -0.674676 | -2.009930 |
| 39 | N | -1.177877 | 0.878570 | -2.689936 |
| 40 | N | -2.142105 | 0.600548 | -1.758699 |
| 41 | C | -2.461035 | 1.724086 | -1.028965 |
| 42 | H | -3.197609 | 1.683228 | -0.220549 |
| 43 | C | -1.680488 | 2.773543 | -1.489210 |
| 44 | H | -1.671295 | 3.798535 | -1.111242 |
| 45 | C | -0.880051 | 2.217223 | -2.529446 |
| 46 | N | 0.606190 | 1.670942 | -4.228215 |
| 47 | C | 0.143764 | 2.708565 | -3.412568 |
| 48 | C | 0.673897 | 4.009665 | -3.512402 |
| 49 | H | 0.288273 | 4.799548 | -2.852760 |

| | | | | |
|---|---|---|---|---|
| 50 | C | 1.678143 | 4.283965 | -4.458543 |
| 51 | H | 2.100421 | 5.293872 | -4.548560 |
| 52 | C | 2.130854 | 3.248625 | -5.312249 |
| 53 | H | 2.897715 | 3.435385 | -6.074966 |
| 54 | C | 1.563924 | 1.977779 | -5.169196 |
| 55 | N | 1.878339 | 0.848441 | -5.931571 |
| 56 | N | 1.156261 | -0.292493 | -5.639849 |
| 57 | C | 1.621524 | -1.246615 | -6.483705 |
| 58 | H | 1.197137 | -2.254672 | -6.447673 |
| 59 | C | 2.641826 | -0.720821 | -7.312626 |
| 60 | H | 3.203831 | -1.248653 | -8.089118 |
| 61 | C | 2.789500 | 0.615168 | -6.942999 |
| 62 | H | 3.452061 | 1.403962 | -7.307404 |
| 63 | N | -1.803225 | 0.530166 | 3.929537 |
| 64 | C | -2.808247 | -0.050279 | 4.682379 |
| 65 | C | -4.114995 | 0.425032 | 4.756372 |
| 66 | H | -4.854782 | -0.078020 | 5.393051 |
| 67 | C | -4.456947 | 1.589122 | 4.019735 |
| 68 | H | -5.476594 | 1.993891 | 4.051816 |
| 69 | C | -3.431807 | 2.242005 | 3.291597 |
| 70 | H | -3.649590 | 3.167078 | 2.739147 |
| 71 | C | -2.130121 | 1.728201 | 3.274962 |
| 72 | N | -2.387360 | -1.195530 | 5.399836 |
| 73 | N | -1.043253 | -1.390427 | 5.559304 |
| 74 | C | -0.912799 | -2.533718 | 6.266153 |
| 75 | H | 0.090041 | -2.900563 | 6.511531 |
| 76 | C | -2.178287 | -3.081640 | 6.573781 |
| 77 | H | -2.398560 | -3.997151 | 7.131219 |
| 78 | C | -3.105561 | -2.208936 | 5.996778 |
| 79 | H | -4.196690 | -2.245782 | 5.943310 |
| 80 | C | -1.025350 | 2.397870 | 2.596277 |
| 81 | N | 0.172899 | 1.761789 | 2.493292 |
| 82 | N | 1.011423 | 2.648119 | 1.897093 |
| 83 | C | 0.379339 | 3.832065 | 1.610082 |
| 84 | H | 0.913671 | 4.661870 | 1.134839 |
| 85 | C | -0.935780 | 3.714140 | 2.041255 |
| 86 | H | -1.711784 | 4.483843 | 1.997591 |
| 87 | C | 2.399424 | 2.290852 | 1.612360 |
| 88 | H | 2.657342 | 1.478960 | 2.319419 |
| 89 | H | 3.028729 | 3.168274 | 1.866109 |
| 90 | C | 2.682701 | 1.861182 | 0.158410 |
| 91 | H | 3.787893 | 1.783284 | 0.057072 |
| 92 | H | 2.369260 | 2.671053 | -0.535275 |
| 93 | C | 2.030899 | 0.532913 | -0.253143 |
| 94 | H | 0.929005 | 0.614889 | -0.184745 |
| 95 | H | 2.329387 | -0.259990 | 0.465186 |
| 96 | C | 2.410174 | 0.105100 | -1.683006 |
| 97 | H | 2.014850 | 0.817385 | -2.430791 |
| 98 | H | 3.515368 | 0.073659 | -1.794930 |
| 99 | N | 0.838162 | -1.326424 | -2.944906 |
| 100 | N | 1.877188 | -1.199313 | -2.061261 |
| 101 | C | 2.265799 | -2.427695 | -1.577077 |
| 102 | H | 3.081192 | -2.510458 | -0.852122 |
| 103 | C | 1.461284 | -3.397319 | -2.157968 |
| 104 | H | 1.514197 | -4.478419 | -2.001765 |
| 105 | C | 0.570436 | -2.681095 | -3.011981 |
| 106 | N | -1.025882 | -1.870345 | -4.486654 |
| 107 | C | -0.503906 | -3.029220 | -3.901146 |
| 108 | C | -1.028839 | -4.301443 | -4.201968 |
| 109 | H | -0.596553 | -5.190138 | -3.721127 |
| 110 | C | -2.091580 | -4.419824 | -5.115255 |
| 111 | H | -2.512508 | -5.404863 | -5.357931 |

| | | | | |
|---|---|---|---|---|
| 112 | C | -2.607062 | -3.256969 | -5.738261 |
| 113 | H | -3.423509 | -3.319036 | -6.469352 |
| 114 | C | -2.040292 | -2.022026 | -5.406061 |
| 115 | N | -2.408435 | -0.782157 | -5.937597 |
| 116 | N | -1.674489 | 0.301836 | -5.495337 |
| 117 | C | -2.199084 | 1.381319 | -6.126583 |
| 118 | H | -1.780308 | 2.374448 | -5.938129 |
| 119 | C | -3.268909 | 0.992498 | -6.968235 |
| 120 | H | -3.884384 | 1.639498 | -7.600710 |
| 121 | C | -3.385032 | -0.389962 | -6.831882 |
| 122 | H | -4.066340 | -1.113282 | -7.286440 |

$[Cr_2(\mu\text{-}L^1)_2]^0$ conformation (c), high-spin

| | | | | |
|---|---|---|---|---|
| 1 | Cr | 0.246391 | 0.087306 | 4.317208 |
| 2 | Cr | -0.212358 | -0.126217 | -4.245576 |
| 3 | N | 2.209777 | -0.356050 | 4.774251 |
| 4 | C | 2.888194 | 0.401634 | 5.683059 |
| 5 | C | 4.223336 | 0.187889 | 6.016333 |
| 6 | H | 4.735533 | 0.813882 | 6.757802 |
| 7 | C | 4.905907 | -0.883487 | 5.361468 |
| 8 | H | 5.960785 | -1.082060 | 5.592312 |
| 9 | C | 4.217243 | -1.676936 | 4.421542 |
| 10 | H | 4.729747 | -2.504124 | 3.909932 |
| 11 | C | 2.869431 | -1.411022 | 4.133406 |
| 12 | N | 2.076033 | 1.416374 | 6.244660 |
| 13 | N | 0.784914 | 1.526459 | 5.778998 |
| 14 | C | 0.230467 | 2.552489 | 6.461981 |
| 15 | H | -0.806453 | 2.840242 | 6.258297 |
| 16 | C | 1.158496 | 3.107103 | 7.372807 |
| 17 | H | 1.003366 | 3.943165 | 8.061066 |
| 18 | C | 2.327671 | 2.361881 | 7.213114 |
| 19 | H | 3.299325 | 2.425375 | 7.709349 |
| 20 | C | 2.009658 | -2.125650 | 3.214059 |
| 21 | N | 0.745176 | -1.619264 | 3.012943 |
| 22 | N | 0.113266 | -2.498872 | 2.182794 |
| 23 | C | 0.945935 | -3.529351 | 1.832363 |
| 24 | H | 0.603298 | -4.323676 | 1.161113 |
| 25 | C | 2.166826 | -3.333119 | 2.467026 |
| 26 | H | 3.040431 | -3.990245 | 2.431555 |
| 27 | C | -1.233520 | -2.237976 | 1.682714 |
| 28 | H | -1.747812 | -1.630594 | 2.451902 |
| 29 | H | -1.754809 | -3.215365 | 1.610698 |
| 30 | C | -1.257489 | -1.511409 | 0.325881 |
| 31 | H | -0.796317 | -0.513639 | 0.459161 |
| 32 | H | -0.629623 | -2.066334 | -0.401701 |
| 33 | C | -2.680260 | -1.352821 | -0.229322 |
| 34 | H | -3.297987 | -0.757252 | 0.477414 |
| 35 | H | -3.176016 | -2.345561 | -0.296760 |
| 36 | C | -2.737380 | -0.735965 | -1.638378 |
| 37 | H | -2.183412 | -1.358605 | -2.365845 |
| 38 | H | -3.793114 | -0.684276 | -1.985071 |
| 39 | N | -1.224646 | 0.877200 | -2.718915 |
| 40 | N | -2.130197 | 0.589243 | -1.731147 |
| 41 | C | -2.415144 | 1.711610 | -0.997531 |
| 42 | H | -3.103228 | 1.673839 | -0.146679 |
| 43 | C | -1.668825 | 2.767936 | -1.505569 |
| 44 | H | -1.642472 | 3.790328 | -1.120932 |
| 45 | C | -0.913509 | 2.214454 | -2.582630 |
| 46 | N | 0.606864 | 1.667690 | -4.284223 |
| 47 | C | 0.096996 | 2.710020 | -3.479575 |
| 48 | C | 0.597737 | 4.018133 | -3.601945 |

| | | | | |
|---|---|---|---|---|
| 49 | H | 0.178990 | 4.812951 | -2.967784 |
| 50 | C | 1.613566 | 4.306704 | -4.532180 |
| 51 | H | 2.006299 | 5.326806 | -4.634357 |
| 52 | C | 2.111686 | 3.263180 | -5.364213 |
| 53 | H | 2.878476 | 3.460384 | -6.124450 |
| 54 | C | 1.574633 | 1.985024 | -5.203894 |
| 55 | N | 1.921466 | 0.855247 | -5.967865 |
| 56 | N | 1.204290 | -0.292177 | -5.710032 |
| 57 | C | 1.689500 | -1.235575 | -6.549698 |
| 58 | H | 1.268085 | -2.245678 | -6.526958 |
| 59 | C | 2.721242 | -0.697386 | -7.351888 |
| 60 | H | 3.304474 | -1.210792 | -8.122057 |
| 61 | C | 2.848509 | 0.637499 | -6.960241 |
| 62 | H | 3.512417 | 1.434643 | -7.304243 |
| 63 | N | -1.823995 | 0.547405 | 3.948351 |
| 64 | C | -2.817151 | -0.005307 | 4.720543 |
| 65 | C | -4.098884 | 0.513215 | 4.865467 |
| 66 | H | -4.824356 | 0.023648 | 5.527791 |
| 67 | C | -4.427047 | 1.713785 | 4.163960 |
| 68 | H | -5.428424 | 2.155558 | 4.247295 |
| 69 | C | -3.413887 | 2.341370 | 3.417428 |
| 70 | H | -3.618567 | 3.288669 | 2.898110 |
| 71 | C | -2.125835 | 1.783435 | 3.333390 |
| 72 | N | -2.438552 | -1.205603 | 5.388622 |
| 73 | N | -1.125003 | -1.409859 | 5.687395 |
| 74 | C | -1.077382 | -2.604341 | 6.304615 |
| 75 | H | -0.112238 | -2.996582 | 6.645538 |
| 76 | C | -2.367988 | -3.179151 | 6.414527 |
| 77 | H | -2.646171 | -4.138977 | 6.860415 |
| 78 | C | -3.220653 | -2.258512 | 5.805619 |
| 79 | H | -4.296159 | -2.284506 | 5.609670 |
| 80 | C | -1.026054 | 2.428713 | 2.635026 |
| 81 | N | 0.171792 | 1.781873 | 2.524575 |
| 82 | N | 1.015408 | 2.662372 | 1.914222 |
| 83 | C | 0.385197 | 3.845544 | 1.623146 |
| 84 | H | 0.919417 | 4.669026 | 1.135755 |
| 85 | C | -0.927070 | 3.739541 | 2.064426 |
| 86 | H | -1.697154 | 4.514214 | 2.015419 |
| 87 | C | 2.398740 | 2.300684 | 1.618916 |
| 88 | H | 2.667105 | 1.493627 | 2.327835 |
| 89 | H | 3.032065 | 3.178803 | 1.860706 |
| 90 | C | 2.669949 | 1.861304 | 0.165411 |
| 91 | H | 3.774697 | 1.796226 | 0.049924 |
| 92 | H | 2.336802 | 2.660582 | -0.531386 |
| 93 | C | 2.030594 | 0.521437 | -0.226358 |
| 94 | H | 0.928864 | 0.585728 | -0.137052 |
| 95 | H | 2.355769 | -0.263469 | 0.489725 |
| 96 | C | 2.387838 | 0.096095 | -1.661714 |
| 97 | H | 1.972151 | 0.803726 | -2.403064 |
| 98 | H | 3.490836 | 0.078945 | -1.794516 |
| 99 | N | 0.856833 | -1.340956 | -2.962811 |
| 100 | N | 1.856810 | -1.211644 | -2.031723 |
| 101 | C | 2.235483 | -2.441082 | -1.559715 |
| 102 | H | 3.018222 | -2.532684 | -0.800161 |
| 103 | C | 1.469576 | -3.412483 | -2.193507 |
| 104 | H | 1.529121 | -4.494463 | -2.045762 |
| 105 | C | 0.597766 | -2.695236 | -3.069501 |
| 106 | N | -1.032579 | -1.890542 | -4.544566 |
| 107 | C | -0.461617 | -3.049352 | -3.970862 |
| 108 | C | -0.958164 | -4.325901 | -4.296013 |
| 109 | H | -0.494125 | -5.213457 | -3.841694 |
| 110 | C | -2.030735 | -4.463113 | -5.193252 |

| | | | | |
|---|---|---|---|---|
| 111 | H | -2.422496 | -5.456112 | -5.449939 |
| 112 | C | -2.592828 | -3.297636 | -5.792801 |
| 113 | H | -3.407276 | -3.373392 | -6.524797 |
| 114 | C | -2.056332 | -2.058840 | -5.441714 |
| 115 | N | -2.459944 | -0.818368 | -5.975499 |
| 116 | N | -1.737964 | 0.280373 | -5.569620 |
| 117 | C | -2.292134 | 1.344689 | -6.191928 |
| 118 | H | -1.879818 | 2.344012 | -6.019663 |
| 119 | C | -3.374401 | 0.933703 | -7.003398 |
| 120 | H | -4.017480 | 1.561184 | -7.627578 |
| 121 | C | -3.457561 | -0.451777 | -6.846955 |
| 122 | H | -4.134871 | -1.191350 | -7.281819 |

$[Cr_2(\mu\text{-}L^2)_2]^0$, low-spin

| | | | | |
|---|---|---|---|---|
| 1 | Cr | 3.359932 | -0.002015 | -0.000296 |
| 2 | Cr | -3.359924 | 0.001997 | 0.000215 |
| 3 | N | 3.557361 | 1.306488 | 1.446737 |
| 4 | C | 4.437133 | 1.082037 | 2.480945 |
| 5 | C | 4.710553 | 2.014165 | 3.487286 |
| 6 | H | 5.409791 | 1.770990 | 4.297629 |
| 7 | C | 4.083098 | 3.281465 | 3.413690 |
| 8 | H | 4.274045 | 4.036056 | 4.188527 |
| 9 | C | 3.245341 | 3.570996 | 2.322461 |
| 10 | H | 2.781983 | 4.561856 | 2.214551 |
| 11 | C | 3.000523 | 2.583972 | 1.346903 |
| 12 | N | 5.037248 | -0.174062 | 2.349481 |
| 13 | N | 4.723838 | -0.866367 | 1.198004 |
| 14 | C | 5.426943 | -2.023945 | 1.261929 |
| 15 | C | 6.188373 | -2.075128 | 2.454468 |
| 16 | C | 5.925220 | -0.885249 | 3.131555 |
| 17 | C | 2.236070 | 2.691347 | 0.135829 |
| 18 | N | 2.109026 | 1.517224 | -0.569135 |
| 19 | N | 1.432213 | 1.851790 | -1.717838 |
| 20 | C | 1.151158 | 3.213146 | -1.747362 |
| 21 | C | 1.651207 | 3.774180 | -0.590260 |
| 22 | N | -0.000001 | 0.000190 | -1.807128 |
| 23 | C | 0.732111 | 0.880714 | -2.492535 |
| 24 | C | 0.792263 | 0.920159 | -3.903024 |
| 25 | H | 1.447670 | 1.635308 | -4.417662 |
| 26 | C | 0.000054 | 0.000109 | -4.608938 |
| 27 | H | 0.000083 | 0.000063 | -5.708507 |
| 28 | C | -0.792176 | -0.919899 | -3.902992 |
| 29 | H | -1.447536 | -1.635108 | -4.417609 |
| 30 | C | -0.732085 | -0.880372 | -2.492501 |
| 31 | N | -1.432058 | -1.851507 | -1.717771 |
| 32 | N | -2.108906 | -1.517067 | -0.569069 |
| 33 | C | -2.236052 | -2.691260 | 0.135848 |
| 34 | C | -1.651064 | -3.774029 | -0.590278 |
| 35 | C | -1.150898 | -3.212868 | -1.747290 |
| 36 | N | -3.557598 | -1.306558 | 1.446970 |
| 37 | C | -3.000712 | -2.584029 | 1.346968 |
| 38 | C | -3.245616 | -3.571187 | 2.322392 |
| 39 | H | -2.782222 | -4.562021 | 2.214404 |
| 40 | C | -4.083485 | -3.281795 | 3.413583 |
| 41 | H | -4.274479 | -4.036475 | 4.188324 |
| 42 | C | -4.710977 | -2.014511 | 3.487284 |
| 43 | H | -5.410274 | -1.771438 | 4.297607 |
| 44 | C | -4.437460 | -1.082276 | 2.481089 |
| 45 | N | -5.037418 | 0.173870 | 2.349715 |
| 46 | N | -4.723725 | 0.866239 | 1.198377 |
| 47 | C | -5.426698 | 2.023893 | 1.262411 |

| | | | | |
|---|---|---|---|---|
| 48 | C | -6.188322 | 2.075027 | 2.454814 |
| 49 | C | -5.925415 | 0.885041 | 3.131761 |
| 50 | N | 3.554101 | -1.311011 | -1.446922 |
| 51 | C | 4.432820 | -1.087957 | -2.482320 |
| 52 | C | 4.704142 | -2.020855 | -3.488486 |
| 53 | H | 5.402583 | -1.778743 | -4.299833 |
| 54 | C | 4.075630 | -3.287554 | -3.413385 |
| 55 | H | 4.264926 | -4.042737 | -4.188050 |
| 56 | C | 3.238989 | -3.575747 | -2.320935 |
| 57 | H | 2.774916 | -4.566149 | -2.211900 |
| 58 | C | 2.996241 | -2.587939 | -1.345626 |
| 59 | N | 5.034097 | 0.167701 | -2.352187 |
| 60 | N | 4.722694 | 0.860626 | -1.200555 |
| 61 | C | 5.426533 | 2.017682 | -1.265911 |
| 62 | C | 6.186401 | 2.067896 | -2.459491 |
| 63 | C | 5.921486 | 0.877959 | -3.135759 |
| 64 | C | 2.232890 | -2.693898 | -0.133571 |
| 65 | N | 2.107573 | -1.519275 | 0.571002 |
| 66 | N | 1.430906 | -1.852529 | 1.720170 |
| 67 | C | 1.148426 | -3.213624 | 1.750422 |
| 68 | C | 1.647311 | -3.775782 | 0.593371 |
| 69 | N | 0.000002 | 0.000223 | 1.809278 |
| 70 | C | 0.731728 | -0.880640 | 2.494673 |
| 71 | C | 0.791925 | -0.920057 | 3.905173 |
| 72 | H | 1.447103 | -1.635441 | 4.419776 |
| 73 | C | -0.000054 | 0.000146 | 4.611131 |
| 74 | H | -0.000082 | 0.000099 | 5.710702 |
| 75 | C | -0.792011 | 0.920389 | 3.905208 |
| 76 | H | -1.447236 | 1.635713 | 4.419831 |
| 77 | C | -0.731752 | 0.881051 | 2.494709 |
| 78 | N | -1.431067 | 1.852877 | 1.720239 |
| 79 | N | -2.107690 | 1.519492 | 0.571062 |
| 80 | C | -2.232925 | 2.694041 | -0.133563 |
| 81 | C | -1.647478 | 3.775994 | 0.593348 |
| 82 | C | -1.148701 | 3.213969 | 1.750492 |
| 83 | N | -3.553867 | 1.310987 | -1.446715 |
| 84 | C | -2.996067 | 2.587934 | -1.345572 |
| 85 | C | -3.238742 | 3.575610 | -2.321011 |
| 86 | H | -2.774717 | 4.566043 | -2.212048 |
| 87 | C | -4.075267 | 3.287271 | -3.413503 |
| 88 | H | -4.264525 | 4.042368 | -4.188260 |
| 89 | C | -4.703728 | 2.020548 | -3.488507 |
| 90 | H | -5.402106 | 1.778331 | -4.299878 |
| 91 | C | -4.432494 | 1.087754 | -2.482201 |
| 92 | N | -5.033908 | -0.167866 | -2.351987 |
| 93 | N | -4.722783 | -0.860737 | -1.200220 |
| 94 | C | -5.426740 | -2.017725 | -1.265481 |
| 95 | C | -6.186412 | -2.067984 | -2.459198 |
| 96 | C | -5.921265 | -0.878145 | -3.135594 |
| 97 | H | 0.546612 | -3.633759 | 2.561624 |
| 98 | H | 1.592525 | -4.826339 | 0.295841 |
| 99 | H | 6.287921 | 0.481499 | -4.085684 |
| 100 | H | 5.342476 | 2.748436 | -0.455966 |
| 101 | H | 6.852112 | 2.871671 | -2.788401 |
| 102 | H | -1.592691 | 4.826531 | 0.295749 |
| 103 | H | -0.546978 | 3.634190 | 2.561716 |
| 104 | H | -6.287509 | -0.481768 | -4.085625 |
| 105 | H | -6.852152 | -2.871729 | -2.788124 |
| 106 | H | -5.342885 | -2.748382 | -0.455430 |
| 107 | H | -5.340819 | 2.754388 | 0.452445 |
| 108 | H | -6.853882 | 2.879422 | 2.782511 |
| 109 | H | -6.293666 | 0.489086 | 4.081198 |

| | | | | |
|---|---|---|---|---|
| 110 | H | -1.597536 | -4.824522 | -0.292284 |
| 111 | H | -0.549006 | -3.633953 | -2.557941 |
| 112 | H | 5.341271 | -2.754335 | 0.451850 |
| 113 | H | 6.853970 | -2.879488 | 2.782172 |
| 114 | H | 6.293276 | -0.489383 | 4.081103 |
| 115 | H | 0.549353 | 3.634311 | -2.558036 |
| 116 | H | 1.597668 | 4.824653 | -0.292196 |

$[Cr_2(\mu\text{-}L^2)_2]^0$, mixed-spin

| | | | | |
|---|---|---|---|---|
| 1 | Cr | -0.031809 | 0.009906 | 3.246323 |
| 2 | Cr | -0.171399 | -0.149408 | -3.112286 |
| 3 | N | 1.242383 | 1.529112 | -3.409842 |
| 4 | C | 2.374829 | 1.448069 | -4.144196 |
| 5 | C | 3.160102 | 2.536683 | -4.532922 |
| 6 | H | 4.034350 | 2.396036 | -5.179210 |
| 7 | C | 2.736036 | 3.837735 | -4.121155 |
| 8 | H | 3.325200 | 4.723708 | -4.395327 |
| 9 | C | 1.553714 | 3.962784 | -3.396657 |
| 10 | H | 1.197621 | 4.951972 | -3.075653 |
| 11 | C | 0.798651 | 2.811211 | -3.049718 |
| 12 | N | 2.735754 | 0.129995 | -4.556904 |
| 13 | N | 1.760410 | -0.790660 | -4.767284 |
| 14 | C | 2.401283 | -1.897986 | -5.170880 |
| 15 | C | 3.806976 | -1.695816 | -5.223065 |
| 16 | C | 3.987678 | -0.376179 | -4.817288 |
| 17 | C | -0.436585 | 2.857382 | -2.312207 |
| 18 | N | -0.963951 | 1.689220 | -1.840377 |
| 19 | N | -2.143543 | 2.031616 | -1.232820 |
| 20 | C | -2.374859 | 3.397316 | -1.328669 |
| 21 | C | -1.306978 | 3.955078 | -2.003494 |
| 22 | N | -2.117714 | 0.097567 | 0.062873 |
| 23 | C | -2.846591 | 1.110862 | -0.430630 |
| 24 | C | -4.238663 | 1.253887 | -0.175841 |
| 25 | H | -4.822294 | 2.065304 | -0.630592 |
| 26 | C | -4.847786 | 0.305275 | 0.650866 |
| 27 | H | -5.921797 | 0.383005 | 0.873960 |
| 28 | C | -4.083168 | -0.745631 | 1.206333 |
| 29 | H | -4.515550 | -1.485528 | 1.891757 |
| 30 | C | -2.717034 | -0.780053 | 0.864278 |
| 31 | N | -1.859669 | -1.808310 | 1.376777 |
| 32 | N | -0.684063 | -1.484758 | 2.002604 |
| 33 | C | 0.081396 | -2.626464 | 1.971179 |
| 34 | C | -0.634368 | -3.680190 | 1.322195 |
| 35 | C | -1.846837 | -3.127049 | 0.954408 |
| 36 | N | 1.456781 | -1.270106 | 3.265163 |
| 37 | C | 1.347000 | -2.518295 | 2.645972 |
| 38 | C | 2.371794 | -3.479451 | 2.753452 |
| 39 | H | 2.252696 | -4.451212 | 2.253829 |
| 40 | C | 3.526950 | -3.189401 | 3.503086 |
| 41 | H | 4.338131 | -3.925181 | 3.588510 |
| 42 | C | 3.622914 | -1.948183 | 4.178727 |
| 43 | H | 4.490301 | -1.707812 | 4.807035 |
| 44 | C | 2.564885 | -1.042116 | 4.048091 |
| 45 | N | 2.460393 | 0.191214 | 4.699731 |
| 46 | N | 1.276472 | 0.874281 | 4.503547 |
| 47 | C | 1.386612 | 2.017449 | 5.225452 |
| 48 | C | 2.639226 | 2.069463 | 5.881570 |
| 49 | C | 3.307230 | 0.895469 | 5.533038 |
| 50 | N | -1.275406 | -1.727478 | -3.607203 |
| 51 | C | -2.308096 | -1.630953 | -4.509813 |
| 52 | C | -3.061294 | -2.712418 | -4.950354 |

| | | | | |
|---|---|---|---|---|
| 53 | H | -3.873843 | -2.570631 | -5.674179 |
| 54 | C | -2.728232 | -4.010266 | -4.467627 |
| 55 | H | -3.306537 | -4.885209 | -4.791440 |
| 56 | C | -1.627103 | -4.149620 | -3.609673 |
| 57 | H | -1.314281 | -5.142066 | -3.255470 |
| 58 | C | -0.902955 | -3.017231 | -3.194831 |
| 59 | N | -2.475432 | -0.309023 | -4.974661 |
| 60 | N | -1.527992 | 0.602436 | -4.591740 |
| 61 | C | -1.856506 | 1.759424 | -5.203952 |
| 62 | C | -3.025997 | 1.595875 | -5.981928 |
| 63 | C | -3.401290 | 0.262794 | -5.812260 |
| 64 | C | 0.269001 | -2.996163 | -2.369096 |
| 65 | N | 0.765109 | -1.754936 | -2.054452 |
| 66 | N | 1.903697 | -1.985259 | -1.325036 |
| 67 | C | 2.135957 | -3.342873 | -1.189915 |
| 68 | C | 1.118134 | -4.015622 | -1.838780 |
| 69 | N | 1.766553 | -0.011626 | -0.097977 |
| 70 | C | 2.561556 | -0.952596 | -0.612394 |
| 71 | C | 3.971124 | -0.953716 | -0.472623 |
| 72 | H | 4.588609 | -1.729913 | -0.943562 |
| 73 | C | 4.545050 | 0.095139 | 0.260400 |
| 74 | H | 5.635094 | 0.137984 | 0.398110 |
| 75 | C | 3.721192 | 1.085932 | 0.824968 |
| 76 | H | 4.128080 | 1.901464 | 1.436864 |
| 77 | C | 2.331296 | 0.967967 | 0.613166 |
| 78 | N | 1.434855 | 1.924396 | 1.170846 |
| 79 | N | 0.367024 | 1.541935 | 1.941213 |
| 80 | C | -0.420122 | 2.663629 | 2.056454 |
| 81 | C | 0.167290 | 3.756416 | 1.349115 |
| 82 | C | 1.336149 | 3.256604 | 0.806021 |
| 83 | N | -1.509350 | 1.271645 | 3.562651 |
| 84 | C | -1.548514 | 2.518611 | 2.936239 |
| 85 | C | -2.560534 | 3.454054 | 3.229216 |
| 86 | H | -2.562762 | 4.424085 | 2.712643 |
| 87 | C | -3.546675 | 3.138456 | 4.181002 |
| 88 | H | -4.347705 | 3.852980 | 4.414223 |
| 89 | C | -3.485588 | 1.897108 | 4.859846 |
| 90 | H | -4.219347 | 1.636559 | 5.633582 |
| 91 | C | -2.449945 | 1.015837 | 4.531872 |
| 92 | N | -2.204965 | -0.216544 | 5.144988 |
| 93 | N | -1.070518 | -0.880949 | 4.724905 |
| 94 | C | -1.022376 | -2.023482 | 5.452778 |
| 95 | C | -2.126325 | -2.094724 | 6.335834 |
| 96 | C | -2.866286 | -0.932116 | 6.123191 |
| 97 | H | 2.969809 | -3.702304 | -0.580424 |
| 98 | H | 0.990454 | -5.098914 | -1.914551 |
| 99 | H | -4.240373 | -0.311839 | -6.212456 |
| 100 | H | -1.243501 | 2.650836 | -5.041271 |
| 101 | H | -3.537120 | 2.350250 | -6.587021 |
| 102 | H | -0.219737 | 4.774171 | 1.253856 |
| 103 | H | 2.080129 | 3.704003 | 0.140699 |
| 104 | H | -3.784995 | -0.557303 | 6.580951 |
| 105 | H | -2.357147 | -2.894363 | 7.046228 |
| 106 | H | -0.202190 | -2.730059 | 5.295873 |
| 107 | H | 0.561351 | 2.736342 | 5.230309 |
| 108 | H | 3.012900 | 2.863063 | 6.535829 |
| 109 | H | 4.290264 | 0.506339 | 5.808254 |
| 110 | H | -0.301269 | -4.707043 | 1.151422 |
| 111 | H | -2.692016 | -3.534756 | 0.392006 |
| 112 | H | 1.824044 | -2.800488 | -5.403308 |
| 113 | H | 4.584430 | -2.411724 | -5.507493 |
| 114 | H | 4.888833 | 0.224025 | -4.663516 |

| | | | | |
|---|---|---|---|---|
| 115 | H | -3.247364 | 3.858271 | -0.857214 |
| 116 | H | -1.169405 | 5.013767 | -2.243070 |

$[Cr_2(\mu\text{-}L^2)_2]^0$, high-spin

| | | | | |
|---|---|---|---|---|
| 1 | Cr | 2.887823 | 0.123617 | 0.185760 |
| 2 | Cr | -2.887618 | -0.123582 | 0.185772 |
| 3 | N | 3.362886 | 1.625736 | 1.398223 |
| 4 | C | 4.285013 | 1.478619 | 2.408292 |
| 5 | C | 4.695391 | 2.510030 | 3.244063 |
| 6 | H | 5.437205 | 2.329407 | 4.032433 |
| 7 | C | 4.158452 | 3.811898 | 3.027147 |
| 8 | H | 4.457616 | 4.647421 | 3.673281 |
| 9 | C | 3.278718 | 4.009481 | 1.952401 |
| 10 | H | 2.884641 | 5.010705 | 1.727112 |
| 11 | C | 2.896049 | 2.927273 | 1.139156 |
| 12 | N | 4.798922 | 0.164613 | 2.463412 |
| 13 | N | 4.446713 | -0.676909 | 1.442609 |
| 14 | C | 5.083206 | -1.841782 | 1.682647 |
| 15 | C | 5.852170 | -1.753168 | 2.866985 |
| 16 | C | 5.646409 | -0.459074 | 3.347095 |
| 17 | C | 2.064983 | 2.970376 | -0.028004 |
| 18 | N | 1.822731 | 1.760239 | -0.632699 |
| 19 | N | 1.097231 | 2.046086 | -1.760651 |
| 20 | C | 0.895883 | 3.408986 | -1.885285 |
| 21 | C | 1.489614 | 4.026721 | -0.800209 |
| 22 | N | 0.000041 | -0.000221 | -1.860633 |
| 23 | C | 0.520932 | 1.021233 | -2.547113 |
| 24 | C | 0.538237 | 1.091657 | -3.961460 |
| 25 | H | 0.994771 | 1.943027 | -4.482787 |
| 26 | C | -0.000252 | -0.000111 | -4.663075 |
| 27 | H | -0.000369 | -0.000064 | -5.762879 |
| 28 | C | -0.538616 | -1.091929 | -3.961434 |
| 29 | H | -0.995299 | -1.943240 | -4.482723 |
| 30 | C | -0.521015 | -1.021602 | -2.547094 |
| 31 | N | -1.097274 | -2.046453 | -1.760590 |
| 32 | N | -1.822690 | -1.760557 | -0.632606 |
| 33 | C | -2.065037 | -2.970643 | -0.027880 |
| 34 | C | -1.489819 | -4.027030 | -0.800152 |
| 35 | C | -0.896023 | -3.409346 | -1.885216 |
| 36 | N | -3.362843 | -1.625995 | 1.398431 |
| 37 | C | -2.896034 | -2.927518 | 1.139297 |
| 38 | C | -3.278736 | -4.009698 | 1.952539 |
| 39 | H | -2.884716 | -5.010935 | 1.727242 |
| 40 | C | -4.158458 | -3.812076 | 3.027306 |
| 41 | H | -4.457643 | -4.647591 | 3.673432 |
| 42 | C | -4.695366 | -2.510196 | 3.244240 |
| 43 | H | -5.437168 | -2.329553 | 4.032611 |
| 44 | C | -4.284973 | -1.478782 | 2.408480 |
| 45 | N | -4.798855 | -0.164758 | 2.463563 |
| 46 | N | -4.446617 | 0.676809 | 1.442774 |
| 47 | C | -5.083069 | 1.841715 | 1.682818 |
| 48 | C | -5.852053 | 1.753081 | 2.867144 |
| 49 | C | -5.646350 | 0.458958 | 3.347230 |
| 50 | N | 3.145589 | -1.489282 | -1.303882 |
| 51 | C | 3.836835 | -1.367074 | -2.459679 |
| 52 | C | 4.113967 | -2.415507 | -3.340802 |
| 53 | H | 4.740920 | -2.257018 | -4.226089 |
| 54 | C | 3.619234 | -3.714220 | -3.001340 |
| 55 | H | 3.807796 | -4.567573 | -3.667433 |
| 56 | C | 2.926314 | -3.881457 | -1.803733 |
| 57 | H | 2.545947 | -4.870189 | -1.509646 |

| | | | | |
|---|---|---|---|---|
| 58 | C | 2.698682 | -2.774177 | -0.947345 |
| 59 | N | 4.324602 | -0.052621 | -2.733263 |
| 60 | N | 4.557878 | 0.797901 | -1.700250 |
| 61 | C | 5.022631 | 1.922803 | -2.264964 |
| 62 | C | 5.093657 | 1.803525 | -3.678988 |
| 63 | C | 4.630710 | 0.518042 | -3.947339 |
| 64 | C | 2.013971 | -2.848386 | 0.317578 |
| 65 | N | 1.683723 | -1.674186 | 0.930554 |
| 66 | N | 1.132821 | -2.014084 | 2.133779 |
| 67 | C | 1.103331 | -3.388144 | 2.294799 |
| 68 | C | 1.646958 | -3.954533 | 1.154912 |
| 69 | N | 0.000167 | 0.000243 | 2.257826 |
| 70 | C | 0.533945 | -1.018924 | 2.941390 |
| 71 | C | 0.556206 | -1.087420 | 4.357436 |
| 72 | H | 1.023900 | -1.928843 | 4.885459 |
| 73 | C | -0.000216 | 0.000038 | 5.056028 |
| 74 | H | -0.000368 | -0.000047 | 6.155892 |
| 75 | C | -0.556484 | 1.087582 | 4.357456 |
| 76 | H | -1.024378 | 1.928897 | 4.885480 |
| 77 | C | -0.533848 | 1.019278 | 2.941406 |
| 78 | N | -1.132791 | 2.014399 | 2.133825 |
| 79 | N | -1.683620 | 1.674494 | 0.930573 |
| 80 | C | -2.013953 | 2.848663 | 0.317577 |
| 81 | C | -1.647017 | 3.954831 | 1.154936 |
| 82 | C | -1.103387 | 3.388463 | 2.294850 |
| 83 | N | -3.145476 | 1.489504 | -1.303962 |
| 84 | C | -2.698652 | 2.774419 | -0.947367 |
| 85 | C | -2.926375 | 3.881710 | -1.803742 |
| 86 | H | -2.546091 | 4.870469 | -1.509627 |
| 87 | C | -3.619276 | 3.714454 | -3.001366 |
| 88 | H | -3.807907 | 4.567813 | -3.667432 |
| 89 | C | -4.113895 | 2.415710 | -3.340871 |
| 90 | H | -4.740819 | 2.257206 | -4.226179 |
| 91 | C | -3.836700 | 1.367264 | -2.459773 |
| 92 | N | -4.324366 | 0.052788 | -2.733390 |
| 93 | N | -4.557640 | -0.797786 | -1.700371 |
| 94 | C | -5.022329 | -1.922706 | -2.265116 |
| 95 | C | -5.093276 | -1.803378 | -3.679185 |
| 96 | C | -4.630359 | -0.517871 | -3.947505 |
| 97 | H | 0.651485 | -3.840276 | 3.182828 |
| 98 | H | 1.770031 | -5.022845 | 0.953280 |
| 99 | H | 4.465728 | -0.015083 | -4.887683 |
| 100 | H | 5.283654 | 2.776655 | -1.628715 |
| 101 | H | 5.423772 | 2.551001 | -4.407236 |
| 102 | H | -1.770153 | 5.023142 | 0.953309 |
| 103 | H | -0.651603 | 3.840613 | 3.182906 |
| 104 | H | -4.465336 | 0.015268 | -4.887836 |
| 105 | H | -5.423327 | -2.550864 | -4.407451 |
| 106 | H | -5.283361 | -2.776588 | -1.628912 |
| 107 | H | -4.943116 | 2.684442 | 0.998634 |
| 108 | H | -6.474142 | 2.530012 | 3.321076 |
| 109 | H | -6.024437 | -0.054337 | 4.234933 |
| 110 | H | -1.502129 | -5.097958 | -0.580510 |
| 111 | H | -0.286577 | -3.806181 | -2.702161 |
| 112 | H | 4.943300 | -2.684494 | 0.998446 |
| 113 | H | 6.474273 | -2.530091 | 3.320906 |
| 114 | H | 6.024458 | 0.054216 | 4.234819 |
| 115 | H | 0.286380 | 3.805776 | -2.702216 |
| 116 | H | 1.501797 | 5.097650 | -0.580537 |

$[Cr_2(\mu\text{-}L^3)_2]^0$, low-spin

| | | | | |
|---|---|---|---|---|
| 1 | Cr | -0.339160 | -0.110053 | -3.810273 |
| 2 | Cr | 0.341956 | -0.148427 | 3.809598 |
| 3 | C | -1.751266 | 0.587132 | 0.932172 |
| 4 | C | -3.250252 | 0.206761 | -1.700795 |
| 5 | H | -1.478613 | -0.329806 | 1.494697 |
| 6 | H | -3.986837 | 0.196997 | -2.532113 |
| 7 | N | -1.114861 | 1.681465 | 1.658621 |
| 8 | N | -0.192804 | 1.431738 | 2.642527 |
| 9 | C | 0.341964 | 2.663591 | 2.967729 |
| 10 | C | -0.255644 | 3.683335 | 2.167413 |
| 11 | C | -1.163403 | 3.023763 | 1.353921 |
| 12 | H | -1.837813 | 3.392799 | 0.575661 |
| 13 | H | -0.053333 | 4.757655 | 2.192666 |
| 14 | C | 1.322576 | 2.622237 | 4.017892 |
| 15 | C | 2.069880 | 3.682548 | 4.567664 |
| 16 | C | 2.976973 | 3.428071 | 5.610700 |
| 17 | C | 3.109067 | 2.109643 | 6.109522 |
| 18 | C | 2.337950 | 1.099583 | 5.525887 |
| 19 | N | 1.473001 | 1.308756 | 4.475019 |
| 20 | H | 3.788509 | 1.883876 | 6.941652 |
| 21 | H | 3.569060 | 4.242765 | 6.048824 |
| 22 | H | 1.931025 | 4.699357 | 4.174843 |
| 23 | N | 2.325347 | -0.243717 | 5.912590 |
| 24 | N | 1.427590 | -1.050456 | 5.241342 |
| 25 | C | 1.584120 | -2.290079 | 5.769605 |
| 26 | C | 2.583262 | -2.278737 | 6.772658 |
| 27 | C | 3.042821 | -0.965125 | 6.845782 |
| 28 | H | 3.803953 | -0.491736 | 7.470725 |
| 29 | H | 2.929076 | -3.125308 | 7.373505 |
| 30 | H | 0.974034 | -3.120745 | 5.402088 |
| 31 | N | -0.828727 | -1.711078 | 3.555317 |
| 32 | C | -1.971084 | -1.855077 | 4.309573 |
| 33 | C | -2.781283 | -2.995631 | 4.281436 |
| 34 | C | -2.383235 | -4.077088 | 3.459607 |
| 35 | C | -1.189515 | -3.981430 | 2.721458 |
| 36 | C | -0.421766 | -2.802575 | 2.782672 |
| 37 | H | -0.843370 | -4.818641 | 2.099589 |
| 38 | H | -2.994314 | -4.988733 | 3.417145 |
| 39 | H | -3.690233 | -3.049489 | 4.894116 |
| 40 | N | -2.182802 | -0.720224 | 5.098626 |
| 41 | N | -1.192537 | 0.242283 | 5.041873 |
| 42 | C | -1.593920 | 1.231302 | 5.880711 |
| 43 | C | -2.839603 | 0.906161 | 6.469527 |
| 44 | C | -3.196185 | -0.341017 | 5.957571 |
| 45 | H | -0.967034 | 2.119137 | 6.010055 |
| 46 | H | -3.411302 | 1.505209 | 7.184892 |
| 47 | H | -4.067561 | -0.977708 | 6.127653 |
| 48 | C | 0.822639 | -2.501579 | 2.127817 |
| 49 | C | 1.705902 | -3.191612 | 1.246352 |
| 50 | N | 1.312279 | -1.238279 | 2.393349 |
| 51 | C | 2.731094 | -2.296324 | 0.979971 |
| 52 | N | 2.467048 | -1.132207 | 1.667834 |
| 53 | H | 1.596389 | -4.200286 | 0.840079 |
| 54 | H | 3.615003 | -2.381998 | 0.341144 |
| 55 | C | 3.246664 | 0.104968 | 1.696757 |
| 56 | C | 1.751771 | 0.556822 | -0.922276 |
| 57 | H | 1.450767 | -0.348470 | -1.489737 |
| 58 | H | 3.983100 | 0.066396 | 2.527329 |
| 59 | N | -2.505725 | -1.051993 | -1.683476 |
| 60 | N | -1.350021 | -1.182020 | -2.403944 |
| 61 | C | -0.895730 | -2.460073 | -2.146254 |
| 62 | C | -1.800361 | -3.133654 | -1.274076 |

| | | | | |
|---|---|---|---|---|
| 63 | C | -2.803045 | -2.214034 | -1.005530 |
| 64 | H | -3.691775 | -2.281203 | -0.371193 |
| 65 | H | -1.720109 | -4.148711 | -0.876936 |
| 66 | N | 1.141301 | 1.671749 | -1.638943 |
| 67 | N | 0.222125 | 1.450512 | -2.631987 |
| 68 | C | -0.285793 | 2.695648 | -2.949200 |
| 69 | C | 1.214503 | 3.009828 | -1.320766 |
| 70 | C | 0.326403 | 3.695400 | -2.134525 |
| 71 | H | 1.888939 | 3.357880 | -0.532901 |
| 72 | H | 0.145124 | 4.773665 | -2.149941 |
| 73 | N | -1.435475 | 1.375597 | -4.473260 |
| 74 | C | -1.261749 | 2.682140 | -4.004657 |
| 75 | C | -1.986808 | 3.760773 | -4.548576 |
| 76 | C | -2.298807 | 1.191601 | -5.529837 |
| 77 | C | 0.342530 | -2.791291 | -2.797745 |
| 78 | N | 0.785351 | -1.705643 | -3.558730 |
| 79 | C | 1.927239 | -1.877299 | -4.308287 |
| 80 | C | 2.702029 | -3.042302 | -4.287024 |
| 81 | C | 1.073614 | -3.993433 | -2.743756 |
| 82 | C | 2.267179 | -4.118863 | -3.477454 |
| 83 | C | -3.048780 | 2.220631 | -6.107996 |
| 84 | C | -2.894221 | 3.532096 | -5.597322 |
| 85 | H | 0.698369 | -4.825242 | -2.131674 |
| 86 | H | 2.849890 | -5.049158 | -3.441179 |
| 87 | H | 3.611773 | -3.118535 | -4.896211 |
| 88 | H | -3.728962 | 2.014303 | -6.944511 |
| 89 | H | -3.469199 | 4.361461 | -6.030707 |
| 90 | H | -1.830568 | 4.771486 | -4.146641 |
| 91 | N | -2.308313 | -0.148182 | -5.928820 |
| 92 | N | -1.430745 | -0.977647 | -5.258678 |
| 93 | C | -1.604089 | -2.208657 | -5.801826 |
| 94 | C | -2.593061 | -2.168760 | -6.814145 |
| 95 | C | -3.028938 | -0.846612 | -6.876935 |
| 96 | H | -2.947724 | -3.002457 | -7.427701 |
| 97 | H | -1.011983 | -3.053880 | -5.438043 |
| 98 | H | -3.775547 | -0.353519 | -7.504221 |
| 99 | N | 2.178843 | -0.741458 | -5.083930 |
| 100 | N | 1.216979 | 0.249484 | -5.025094 |
| 101 | C | 1.655471 | 1.235123 | -5.849186 |
| 102 | C | 2.896369 | 0.879623 | -6.430396 |
| 103 | C | 3.211229 | -0.383153 | -5.929171 |
| 104 | H | 4.065188 | -1.043594 | -6.096874 |
| 105 | H | 3.492235 | 1.469425 | -7.133640 |
| 106 | H | 1.056690 | 2.142768 | -5.974690 |
| 107 | H | -1.286905 | 0.496989 | -0.068805 |
| 108 | H | -2.506630 | 0.988002 | -1.952583 |
| 109 | H | 1.290722 | 0.473141 | 0.080819 |
| 110 | H | 2.525131 | 0.904829 | 1.955606 |
| 111 | C | -3.962942 | 0.512866 | -0.391557 |
| 112 | C | 3.965081 | 0.403920 | 0.389321 |
| 113 | C | 3.269174 | 0.651893 | -0.827908 |
| 114 | C | -3.264997 | 0.724893 | 0.831333 |
| 115 | C | 4.008605 | 0.961983 | -1.990196 |
| 116 | H | 3.468391 | 1.162095 | -2.927380 |
| 117 | C | 5.376616 | 0.466945 | 0.391935 |
| 118 | H | 5.914056 | 0.272683 | 1.332860 |
| 119 | H | 7.198769 | 0.822659 | -0.738045 |
| 120 | C | 6.100249 | 0.776909 | -0.770082 |
| 121 | H | 5.960290 | 1.266828 | -2.889768 |
| 122 | C | 5.410294 | 1.026186 | -1.968065 |
| 123 | C | -5.372126 | 0.617005 | -0.397869 |
| 124 | H | -5.911478 | 0.450045 | -1.342941 |

| | | | | |
|---|---|---|---|---|
| 125 | C | -3.999934 | 1.040075 | 1.995216 |
| 126 | H | -3.457512 | 1.211585 | 2.936857 |
| 127 | H | -7.187636 | 1.010940 | 0.730094 |
| 128 | C | -6.091095 | 0.932723 | 0.765498 |
| 129 | H | -5.945391 | 1.388926 | 2.892360 |
| 130 | C | -5.398938 | 1.145341 | 1.969332 |

$[Cr_2(\mu\text{-}L^3)_2]^0$, mixed-spin

| | | | | |
|---|---|---|---|---|
| 1 | Cr | -0.337824 | -0.103121 | -3.717171 |
| 2 | Cr | 0.335164 | -0.390055 | 3.914774 |
| 3 | C | -1.904083 | 1.020579 | 0.605173 |
| 4 | C | -3.438343 | 0.272650 | -1.853682 |
| 5 | H | -1.538429 | 0.034668 | 0.955970 |
| 6 | H | -4.181634 | 0.122001 | -2.665620 |
| 7 | N | -1.282496 | 2.002763 | 1.487654 |
| 8 | N | -0.295980 | 1.613703 | 2.337838 |
| 9 | C | 0.163150 | 2.755503 | 2.919391 |
| 10 | C | -0.542577 | 3.890040 | 2.401738 |
| 11 | C | -1.447502 | 3.365560 | 1.488314 |
| 12 | H | -2.193955 | 3.847046 | 0.848662 |
| 13 | H | -0.422088 | 4.943320 | 2.670606 |
| 14 | C | 1.192421 | 2.662208 | 3.946293 |
| 15 | C | 1.796055 | 3.790471 | 4.515695 |
| 16 | C | 2.778926 | 3.654858 | 5.520923 |
| 17 | C | 3.112763 | 2.343890 | 5.954193 |
| 18 | C | 2.488210 | 1.261711 | 5.341531 |
| 19 | N | 1.558803 | 1.358845 | 4.324602 |
| 20 | H | 3.826958 | 2.184480 | 6.772477 |
| 21 | H | 3.248201 | 4.534341 | 5.980023 |
| 22 | H | 1.497352 | 4.788811 | 4.166792 |
| 23 | N | 2.750777 | -0.070325 | 5.747058 |
| 24 | N | 1.856339 | -1.035462 | 5.381254 |
| 25 | C | 2.320401 | -2.192068 | 5.902030 |
| 26 | C | 3.521352 | -1.974347 | 6.614429 |
| 27 | C | 3.778346 | -0.606220 | 6.490484 |
| 28 | H | 4.608098 | 0.008780 | 6.848479 |
| 29 | H | 4.134122 | -2.711895 | 7.141526 |
| 30 | H | 1.768052 | -3.122462 | 5.730071 |
| 31 | N | -0.781596 | -1.982918 | 3.586606 |
| 32 | C | -2.003150 | -2.127662 | 4.218836 |
| 33 | C | -2.793918 | -3.267909 | 4.113040 |
| 34 | C | -2.326660 | -4.350520 | 3.322460 |
| 35 | C | -1.084977 | -4.225852 | 2.663533 |
| 36 | C | -0.332722 | -3.052265 | 2.790896 |
| 37 | H | -0.699875 | -5.043612 | 2.037622 |
| 38 | H | -2.927142 | -5.264207 | 3.225842 |
| 39 | H | -3.759632 | -3.326121 | 4.632397 |
| 40 | N | -2.333288 | -0.987043 | 4.971713 |
| 41 | N | -1.436523 | 0.056850 | 4.931252 |
| 42 | C | -1.953099 | 1.024034 | 5.729411 |
| 43 | C | -3.188158 | 0.607721 | 6.274371 |
| 44 | C | -3.407615 | -0.679201 | 5.777365 |
| 45 | H | -1.411370 | 1.966476 | 5.858828 |
| 46 | H | -3.842644 | 1.168323 | 6.948490 |
| 47 | H | -4.222672 | -1.389399 | 5.936170 |
| 48 | C | 0.927958 | -2.750857 | 2.153439 |
| 49 | C | 1.837056 | -3.481892 | 1.331520 |
| 50 | N | 1.403844 | -1.478511 | 2.350787 |
| 51 | C | 2.867525 | -2.593490 | 1.048502 |
| 52 | N | 2.579271 | -1.404119 | 1.668594 |
| 53 | H | 1.750218 | -4.513953 | 0.981982 |

| | | | | |
|---|---|---|---|---|
| 54 | H | 3.770601 | -2.707106 | 0.440851 |
| 55 | C | 3.380207 | -0.177879 | 1.708391 |
| 56 | C | 1.722565 | 0.460942 | -0.773381 |
| 57 | H | 1.367725 | -0.433297 | -1.326074 |
| 58 | H | 4.174968 | -0.285759 | 2.476997 |
| 59 | N | -2.642402 | -0.942832 | -1.679796 |
| 60 | N | -1.458215 | -1.115705 | -2.344048 |
| 61 | C | -1.031183 | -2.384920 | -2.008260 |
| 62 | C | -1.969867 | -3.004093 | -1.131939 |
| 63 | C | -2.973350 | -2.065358 | -0.951178 |
| 64 | H | -3.886047 | -2.092537 | -0.348680 |
| 65 | H | -1.910942 | -3.995485 | -0.675772 |
| 66 | N | 1.097854 | 1.596331 | -1.444443 |
| 67 | N | 0.236951 | 1.422070 | -2.496704 |
| 68 | C | -0.238921 | 2.683858 | -2.797184 |
| 69 | C | 1.164992 | 2.920381 | -1.072851 |
| 70 | C | 0.331262 | 3.645624 | -1.909867 |
| 71 | H | 1.794798 | 3.231143 | -0.233966 |
| 72 | H | 0.154481 | 4.724351 | -1.888853 |
| 73 | N | -1.350755 | 1.429083 | -4.403684 |
| 74 | C | -1.165063 | 2.719763 | -3.896667 |
| 75 | C | -1.841489 | 3.827414 | -4.444098 |
| 76 | C | -2.178740 | 1.289201 | -5.494454 |
| 77 | C | 0.215057 | -2.774632 | -2.610032 |
| 78 | N | 0.711676 | -1.731985 | -3.396076 |
| 79 | C | 1.874516 | -1.961952 | -4.096564 |
| 80 | C | 2.605777 | -3.151631 | -4.017118 |
| 81 | C | 0.902992 | -3.997401 | -2.494108 |
| 82 | C | 2.109625 | -4.188120 | -3.190671 |
| 83 | C | -2.879322 | 2.349278 | -6.078622 |
| 84 | C | -2.710799 | 3.645406 | -5.533867 |
| 85 | H | 0.481896 | -4.792513 | -1.863812 |
| 86 | H | 2.657619 | -5.136745 | -3.111418 |
| 87 | H | 3.531452 | -3.277496 | -4.593470 |
| 88 | H | -3.533550 | 2.176986 | -6.943002 |
| 89 | H | -3.246386 | 4.498979 | -5.970711 |
| 90 | H | -1.677094 | 4.824762 | -4.013081 |
| 91 | N | -2.213852 | -0.043675 | -5.916125 |
| 92 | N | -1.399922 | -0.913677 | -5.217855 |
| 93 | C | -1.589592 | -2.130070 | -5.788223 |
| 94 | C | -2.523935 | -2.039902 | -6.848016 |
| 95 | C | -2.909653 | -0.701963 | -6.910642 |
| 96 | H | -2.876891 | -2.851529 | -7.491426 |
| 97 | H | -1.046155 | -3.000516 | -5.408019 |
| 98 | H | -3.605622 | -0.172956 | -7.566248 |
| 99 | N | 2.199858 | -0.853271 | -4.883838 |
| 100 | N | 1.282220 | 0.180459 | -4.871811 |
| 101 | C | 1.799806 | 1.137675 | -5.683686 |
| 102 | C | 3.045987 | 0.720933 | -6.210077 |
| 103 | C | 3.281958 | -0.550150 | -5.686627 |
| 104 | H | 4.112227 | -1.249614 | -5.808886 |
| 105 | H | 3.696204 | 1.276631 | -6.892645 |
| 106 | H | 1.248454 | 2.069061 | -5.845171 |
| 107 | H | -1.514062 | 1.155752 | -0.421903 |
| 108 | H | -2.730919 | 1.044239 | -2.214836 |
| 109 | H | 1.322278 | 0.392481 | 0.256351 |
| 110 | H | 2.695352 | 0.611364 | 2.079465 |
| 111 | C | -4.147258 | 0.694298 | -0.576045 |
| 112 | C | 4.015160 | 0.188753 | 0.375647 |
| 113 | C | 3.246885 | 0.520545 | -0.776205 |
| 114 | C | -3.426019 | 1.052725 | 0.597926 |
| 115 | C | 3.917613 | 0.888320 | -1.962789 |

| | | | | |
|---|---|---|---|---|
| 116 | H | 3.325369 | 1.152575 | -2.850760 |
| 117 | C | 5.425144 | 0.231893 | 0.292150 |
| 118 | H | 6.016677 | -0.021092 | 1.185643 |
| 119 | H | 7.178677 | 0.627146 | -0.930135 |
| 120 | C | 6.079922 | 0.598495 | -0.893855 |
| 121 | H | 5.812920 | 1.216096 | -2.967412 |
| 122 | C | 5.319047 | 0.928561 | -2.027555 |
| 123 | C | -5.558270 | 0.713573 | -0.545529 |
| 124 | H | -6.113608 | 0.438007 | -1.455362 |
| 125 | C | -4.141222 | 1.396262 | 1.763988 |
| 126 | H | -3.584123 | 1.659217 | 2.674719 |
| 127 | H | -7.359715 | 1.086914 | 0.613569 |
| 128 | C | -6.259884 | 1.074875 | 0.616103 |
| 129 | H | -6.079509 | 1.679654 | 2.701192 |
| 130 | C | -5.545848 | 1.409642 | 1.777978 |

$[Cr_2(\mu\text{-}L^3)_2]^0$, high-spin

| | | | | |
|---|---|---|---|---|
| 1 | Cr | -0.279095 | -0.242034 | -3.906149 |
| 2 | Cr | 0.307987 | -0.132066 | 3.754797 |
| 3 | C | -1.680094 | 0.486544 | 0.744439 |
| 4 | C | -3.358826 | -0.154334 | -1.713797 |
| 5 | H | -1.328929 | -0.416660 | 1.286210 |
| 6 | H | -4.159191 | -0.278500 | -2.474233 |
| 7 | N | -1.030761 | 1.607237 | 1.415210 |
| 8 | N | -0.225567 | 1.418653 | 2.511343 |
| 9 | C | 0.271500 | 2.669649 | 2.834571 |
| 10 | C | -0.239059 | 3.639277 | 1.919043 |
| 11 | C | -1.050314 | 2.929734 | 1.045227 |
| 12 | H | -1.633608 | 3.254696 | 0.178033 |
| 13 | H | -0.033573 | 4.712930 | 1.897815 |
| 14 | C | 1.181504 | 2.686923 | 3.944263 |
| 15 | C | 1.884201 | 3.775929 | 4.485616 |
| 16 | C | 2.779022 | 3.578067 | 5.553983 |
| 17 | C | 2.938710 | 2.268318 | 6.090577 |
| 18 | C | 2.211727 | 1.227180 | 5.524639 |
| 19 | N | 1.362962 | 1.384405 | 4.448299 |
| 20 | H | 3.608700 | 2.088859 | 6.942100 |
| 21 | H | 3.339920 | 4.419455 | 5.980949 |
| 22 | H | 1.731088 | 4.778821 | 4.061797 |
| 23 | N | 2.205627 | -0.103647 | 5.987979 |
| 24 | N | 1.384672 | -0.982152 | 5.318177 |
| 25 | C | 1.490653 | -2.164201 | 5.966286 |
| 26 | C | 2.382426 | -2.053481 | 7.056012 |
| 27 | C | 2.825399 | -0.727317 | 7.046506 |
| 28 | H | 3.511762 | -0.187685 | 7.703980 |
| 29 | H | 2.677377 | -2.837051 | 7.760142 |
| 30 | H | 0.929030 | -3.031069 | 5.603153 |
| 31 | N | -0.783816 | -1.742760 | 3.408342 |
| 32 | C | -1.976610 | -1.948539 | 4.072332 |
| 33 | C | -2.725518 | -3.114515 | 3.964910 |
| 34 | C | -2.218628 | -4.175130 | 3.161274 |
| 35 | C | -0.973541 | -4.014910 | 2.522604 |
| 36 | C | -0.264555 | -2.811027 | 2.653728 |
| 37 | H | -0.544362 | -4.826912 | 1.918689 |
| 38 | H | -2.785283 | -5.110223 | 3.063569 |
| 39 | H | -3.674810 | -3.223833 | 4.506191 |
| 40 | N | -2.297863 | -0.848987 | 4.890397 |
| 41 | N | -1.374660 | 0.173973 | 4.930948 |
| 42 | C | -1.857586 | 1.080160 | 5.813752 |
| 43 | C | -3.094645 | 0.648689 | 6.339472 |
| 44 | C | -3.354521 | -0.584565 | 5.731423 |

| | | | | |
|---|---|---|---|---|
| 45 | H | -1.292860 | 1.997040 | 6.011162 |
| 46 | H | -3.729094 | 1.167134 | 7.064429 |
| 47 | H | -4.188429 | -1.283531 | 5.832811 |
| 48 | C | 0.992958 | -2.445848 | 2.066605 |
| 49 | C | 1.915766 | -3.072321 | 1.177219 |
| 50 | N | 1.440885 | -1.175528 | 2.383839 |
| 51 | C | 2.919559 | -2.138190 | 0.968609 |
| 52 | N | 2.611794 | -1.007979 | 1.690470 |
| 53 | H | 1.843512 | -4.064757 | 0.725161 |
| 54 | H | 3.817894 | -2.177323 | 0.344926 |
| 55 | C | 3.424761 | 0.194428 | 1.871890 |
| 56 | C | 1.964625 | 0.959574 | -0.632703 |
| 57 | H | 1.584133 | -0.012536 | -1.005443 |
| 58 | H | 4.144444 | 0.043836 | 2.705714 |
| 59 | N | -2.541733 | -1.369518 | -1.673678 |
| 60 | N | -1.381022 | -1.433220 | -2.382529 |
| 61 | C | -0.896020 | -2.705493 | -2.205172 |
| 62 | C | -1.784064 | -3.444500 | -1.367394 |
| 63 | C | -2.814000 | -2.563654 | -1.057911 |
| 64 | H | -3.706976 | -2.687533 | -0.437415 |
| 65 | H | -1.687244 | -4.479957 | -1.030429 |
| 66 | N | 1.400764 | 1.970990 | -1.519582 |
| 67 | N | 0.404146 | 1.632179 | -2.386873 |
| 68 | C | -0.005614 | 2.803643 | -2.957434 |
| 69 | C | 1.612536 | 3.326742 | -1.496377 |
| 70 | C | 0.739186 | 3.900025 | -2.410252 |
| 71 | H | 2.364979 | 3.771240 | -0.837131 |
| 72 | H | 0.657301 | 4.962624 | -2.656084 |
| 73 | N | -1.518528 | 1.489449 | -4.317795 |
| 74 | C | -1.026747 | 2.770655 | -3.989070 |
| 75 | C | -1.520485 | 3.927629 | -4.619854 |
| 76 | C | -2.445531 | 1.439834 | -5.328240 |
| 77 | C | 0.351986 | -3.028539 | -2.868209 |
| 78 | N | 0.772696 | -2.026265 | -3.745802 |
| 79 | C | 1.967932 | -2.167090 | -4.388724 |
| 80 | C | 2.786904 | -3.284801 | -4.250833 |
| 81 | C | 1.123855 | -4.185237 | -2.691600 |
| 82 | C | 2.344922 | -4.328527 | -3.384861 |
| 83 | C | -2.960323 | 2.542667 | -6.000643 |
| 84 | C | -2.499906 | 3.840578 | -5.621961 |
| 85 | H | 0.778215 | -4.970260 | -2.003988 |
| 86 | H | 2.962838 | -5.225971 | -3.249212 |
| 87 | H | 3.740567 | -3.363187 | -4.788179 |
| 88 | H | -3.679037 | 2.407615 | -6.819119 |
| 89 | H | -2.877941 | 4.738594 | -6.127536 |
| 90 | H | -1.126544 | 4.907955 | -4.316151 |
| 91 | N | -2.872672 | 0.122690 | -5.665163 |
| 92 | N | -2.012980 | -0.914572 | -5.476121 |
| 93 | C | -2.681621 | -2.014684 | -5.867519 |
| 94 | C | -3.984858 | -1.689055 | -6.319794 |
| 95 | C | -4.079813 | -0.305253 | -6.166570 |
| 96 | H | -4.760190 | -2.365521 | -6.692817 |
| 97 | H | -2.191246 | -2.992836 | -5.800965 |
| 98 | H | -4.904410 | 0.392043 | -6.339212 |
| 99 | N | 2.266365 | -1.056041 | -5.214293 |
| 100 | N | 1.424734 | 0.031593 | -5.144752 |
| 101 | C | 1.923077 | 0.941619 | -6.012540 |
| 102 | C | 3.085181 | 0.445254 | -6.646093 |
| 103 | C | 3.278730 | -0.833544 | -6.120449 |
| 104 | H | 4.034902 | -1.595018 | -6.327161 |
| 105 | H | 3.703797 | 0.945804 | -7.396832 |
| 106 | H | 1.418969 | 1.906918 | -6.130530 |

| | | | | |
|---|---|---|---|---|
| 107 | H | -1.292827 | 0.425207 | -0.290601 |
| 108 | H | -2.685876 | 0.639860 | -2.098244 |
| 109 | H | 1.547007 | 1.091826 | 0.384580 |
| 110 | H | 2.721501 | 0.982725 | 2.207322 |
| 111 | C | -3.983623 | 0.219509 | -0.379385 |
| 112 | C | 4.171868 | 0.587134 | 0.607731 |
| 113 | C | 3.486148 | 0.949750 | -0.585403 |
| 114 | C | -3.203207 | 0.558059 | 0.763264 |
| 115 | C | 4.235510 | 1.262811 | -1.739253 |
| 116 | H | 3.702717 | 1.529118 | -2.663929 |
| 117 | C | 5.583396 | 0.572240 | 0.608356 |
| 118 | H | 6.111631 | 0.293624 | 1.533267 |
| 119 | H | 7.418813 | 0.889503 | -0.513586 |
| 120 | C | 6.319295 | 0.904008 | -0.540862 |
| 121 | H | 6.200775 | 1.489262 | -2.635975 |
| 122 | C | 5.639789 | 1.242226 | -1.722567 |
| 123 | C | -5.392433 | 0.269568 | -0.284359 |
| 124 | H | -5.992345 | 0.009579 | -1.170298 |
| 125 | C | -3.862374 | 0.945631 | 1.949940 |
| 126 | H | -3.259586 | 1.218414 | 2.827808 |
| 127 | H | -7.134073 | 0.685946 | 0.948714 |
| 128 | C | -6.035643 | 0.652337 | 0.902985 |
| 129 | H | -5.747575 | 1.297451 | 2.965888 |
| 130 | C | -5.262764 | 0.994248 | 2.026170 |

## References


[1] C. Bartual-Murgui, C. Codina, O. Roubeau, G. Aromí, *Chem. – Eur. J.* **2016**, *22*, 12767–12776.

[2] G. M. Sheldrick, *Acta Cryst. Sect. C: Struct. Chem.* **2015**, *71*, 3–8.

[3] L. J. Barbour, *J. Appl. Cryst.* **2020**, *53*, 1141–1146.

[4] O. V. Dolomanov, L. J. Bourhis, R. J. Gildea, J. A. K. Howard, H. Puschmann, *J. Appl. Cryst.* **2009**. *42*, 339–341.

[5] C. Foces-Foces, I. Alkorta, J. Elguero, *Acta Cryst. Sect. B: Struct. Sci.* **2000**, *56*, 1018–1028.

[6] P. Guionneau, M. Marchivie, G. Bravic, J.-F. Létard, D. Chasseau, *Top. Curr. Chem.* **2004**, *234*, 97–128.

[7] I. Capel Berdiell, R. Kulmaczewski, M. A. Halcrow, *Inorg. Chem.* **2017**, *56*, 8817–8828.

[8] J. K. McCusker, A. L. Rheingold, D. N. Hendrickson, *Inorg. Chem.* **1996**, *35*, 2100–2112.

[9] J. M. Holland, J. A. McAllister, C. A. Kilner, M. Thornton-Pett, A. J. Bridgeman, M. A. Halcrow, *J. Chem. Soc. Dalton Trans.* **2002**, 548–554.

[10] S. Vela, J. J. Novoa, J. Ribas-Arino, *Phys. Chem. Chem. Phys.* **2014**, *16*, 27012–27024.

[11] M. A. Halcrow, *Coord. Chem. Rev.* **2009**, *253*, 2493–2514.

[12] L. J. Kershaw Cook, R. Mohammed, G. Sherborne, T.D. Roberts, S. Alvarez, M. A. Halcrow, *Coord. Chem. Rev.* **2015**, *289–290*, 2–12.

[13] G. A. Craig, O. Roubeau, G. Aromí, *Coord. Chem. Rev.* **2014**, *269*, 13–31.

[14] A. J. Gordon, R. A. Ford, *The Chemists Companion – A Handbook of Practical Data, Techniques and References*, John Wiley, Chichester, UK, **1972**. p. 109.
